# Access to 2-Alkenyl-furans via a Cascade of Pd-Catalyzed Cyclization/Coupling Followed by Oxidative Aromatization with DDQ

**DOI:** 10.1021/acs.joc.4c00149

**Published:** 2024-05-03

**Authors:** Bartosz Bisek, Wojciech Chaładaj

**Affiliations:** Institute of Organic Chemistry, Polish Academy of Sciences, Kasprzaka 44/52, 01-224 Warsaw, Poland

## Abstract

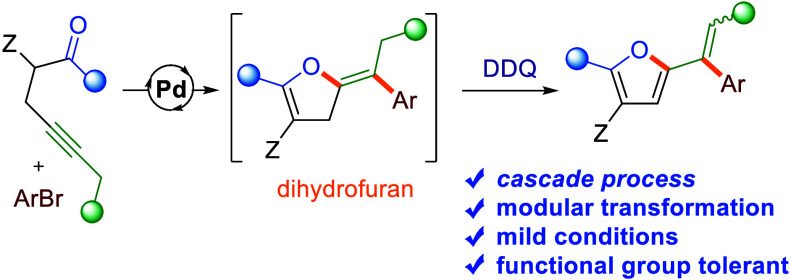

An unprecedented DDQ-mediated oxidative aromatization of 2-bezylidene-dihydrofurans yielding 2-alkenyl-furans is disclosed. Integration of this transformation with a prior Pd-catalyzed reaction of α-propargylic-β-ketoesters and (hetero)aryl halides into a one-pot cascade process opens a direct modular route to highly substituted 2-vinyl-furans. Experimental and computational studies reveal that the crucial step of the oxidative-aromatization involves facile hydride transfer from the dihydrofuran ring to the O-center of DDQ.

Due to their biological relevance, efficient and selective routes to diversely substituted furans are of the utmost importance. In particular, 2-alkenylfurans constitute a group of compounds that have attracted attention as useful building blocks^1^ and biologically relevant compounds.^2^ Among the few recently reported protocols,^3^ intramolecular keto-ene-yne cyclizations have gained the most recognition and become the method of choice for their synthesis.^4−7^ Exact methods on this subject vary, yet the core strategy remains the same and relies on the exploitation of a carbenoid moiety formed in a cascade 5-exo-dig cyclization/cross-coupling reaction. Vicente et al. proposed zinc as a metal candidate for forming the carbenoid in the reaction cycle;^4^ however, the usage of palladium,^5^ silver,^6^ and other metals^7^ have been reported as well (Scheme 1a). Reported protocols are limited in regard to ene-ynes and often suffer from the high catalyst loading required to ensure conversion.

**Scheme 1:**
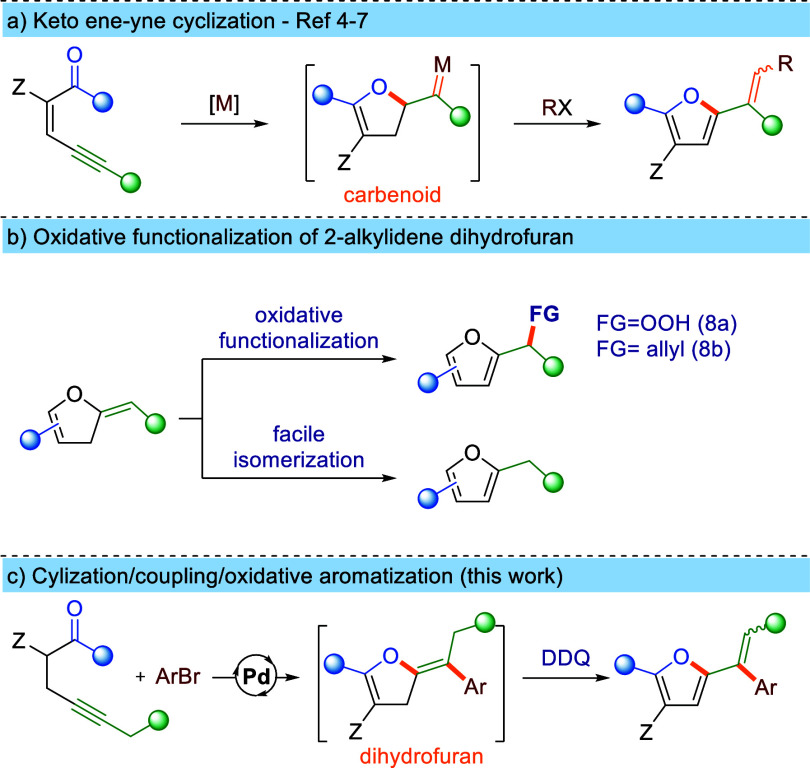
Previous Methodologies and Aim of This Work

We hypothesized that 2-vinyl furans could also be accessed by oxidative aromatization of 2-alkylidene dihydrofurans; however, no such transformation has been reported to date. Furthermore, only a few examples of aromatization-driven oxidative functionalizations of dihydrofurans bearing an exocyclic alkene motif have been disclosed.^8^ These typically suffer from narrow scope, often restricted to simple substituted alkylidene dihydrofurans. Underdevelopment of this strategy stems mainly from poor availability of more elaborated derivatives of alkylidene dihydrofurans and their high tendency to isomerize to the corresponding furans, particularly under acidic or basic conditions (Scheme 1b). Recently we proposed a Pd-catalyzed tandem cyclization/coupling strategy enabling access to highly substituted 2-benzylidenedihydrofurans.^9^ We envisioned that integration of this strategy with a further oxidative-aromatization step into a one-pot cascade transformation could provide an elegant and straightforward route to 2-alkenyl-3,5-furans. Avoiding direct synthesis of the dihydrofuran is beneficial, as it not only shortens the synthetic sequence through elimination of isolation/purification steps but also minimizes the risk of undesirable isomerization. Herein we report a new strategy for the modular synthesis of highly substituted 2-alkenylfurans via the one-pot cascade of Pd-catalyzed cyclization/coupling of propargyl dicarbonyl compounds with aryl bromides followed by oxidative aromatization with DDQ.

Notably, the latter component of the sequential transformation, i.e. oxidative aromatization, is unprecedented, even for separately prepared 2-benzylidenedihydrofurans.

To test our hypothesis that 2-benzylidenedihydrofuran can be oxidatively transformed into the corresponding 2-vinylfuran, compound **1** was chosen as the benchmark substrate (Table 1). Initial tests of various oxidants revealed that DDQ is capable of promoting the reaction with a high efficiency. Preliminary investigations also revealed that the reaction can operate in a wide range of solvents, of which dichloroethane performed the best, enabling almost quantitative formation of **2** in less than 30 min. Furthermore, we observed that using excess DDQ substantially decreased the outcome of the process, presumably due to reactivity of the resulting vinylfuran **2** toward DDQ.^11a^

**Table 1:**
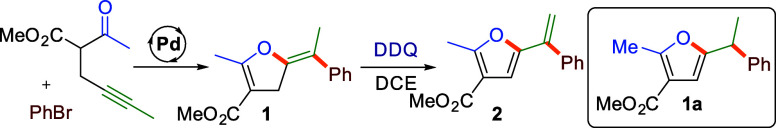
Evaluation of Reaction Conditions

Entry	Variable	Yield **2**
1^a^	None	92%
2^a^	Furan **1a** as substrate	4%^c^
3^a^	Toluene as a solvent	77%
4^a^	THF as a solvent	63%
5^a^	MeCN as a solvent	64%
6^a^	DMF as a solvent	71%
7^a^	1.5 equiv of DDQ	29%
		
8^b^	None	89%
9^b^	Toluene as solvent	84%
10^b^	No cosolvent	74%

a Reaction conditions: 1 (0.40 mmol), DDQ (0.44 mmol), DCE (1.0 mL), 0.5 h, rt.

b Reaction conditions: methyl 2-acetylhex-4-ynoate (0.10 mmol), PhBr (0.11 mmol), XPhos Pd G3 (0.5 mol %), K_2_CO_3_ (0.11 mmol) DMF (0.5 mL), 24 h, rt, after that adding DDQ (0.11 mmol), DCE (0.5 mL), 0.5 h rt.

c 44% of starting material recovered (methyl 2-methyl-5-(1-phenylethyl)furan-3-carboxylate, **1a**).

Interestingly, furan **1a**, independently obtained via isomerization of **1**, did not provide **2** under the reaction conditions, although some consumption of starting material was observed. Encouraged by the preliminary results, we decided to design a one-pot transformation merging a Pd-catalyzed tandem cyclization/coupling with further oxidative-aromatization (Table 1, entries 8–10). To our delight, treatment of the in situ obtained **1** with DDQ provides a similar if not better yield of the desired vinylfuran **2**, compared to the reaction of independently prepared and isolated **1** performed in DMF. Use of polar aprotic solvent (DMF) is essential for the first step of the cascade, i.e., Pd-catalyzed cyclization/coupling. Although it cannot be completely replaced, the use of less polar cosolvents at the stage of DDQ oxidation brought further improvement of the overall efficiency; combination of DMF with dichloroethane enabled isolation of the desired vinylfuran **2** in 89% yield, in a sequential process starting from methyl-2-acetylhex-4-ynoate and bromobenzene.

With satisfactory conditions in hand for the model reaction, we proceeded to explore the scope of the established protocol providing access to highly substituted 2-alkenyl-furans (Table 2). First, various aryl and heteroaryl bromides in combination with methyl 2-acetylhex-4-ynoate were surveyed under the optimized conditions. Thus, incorporation of electron rich (**3**-**4**, **9**-**11**), neutral (**2**, **7**) and mildly deficient (**5**-**6**, **8**) aryl groups into 2-alkenyl-furans was achieved with good yields. Importantly, unprotected alcohols (**11**), amides (**4**), aryl chlorides (**5**, **8**) and heterocyclic motifs (**14**-**16**) were compatible with the reaction conditions. Strongly electron deficient aryl bromides (e.g., NO_2_ or CN substituted) are not competent reaction partners, due to very fast isomerization of the resulting dihydrofuran to furan. Various activated ketones, including ketoesters (**17**-**22**), diketones (**23**-**25**) and ketosulfones (**26**-**27**) also gave rise to the expected products with satisfactory yields and enabled variation of the substitution pattern in positions 4 and 5 of the furan backbone. Exchange of the methyl-cap at the terminus of alkyne moiety with a longer alkyl homologues (**21**, **22**) or tethered alkene motif (**28**) is also well tolerated; however, formation of a mixture of *E*/*Z* isomers is observed. In contrast, substrates bearing secondary alkyl substituents (^*i*^Pr) at the alkyne terminus are not compatible with the reaction conditions. Noticeably, the reaction is well scalable; vinyfurane **2** was isolated in comparable yields for reactions run at 0.4 and 2 mmol scales (75% and 73%, respectively)

**Table 2:**
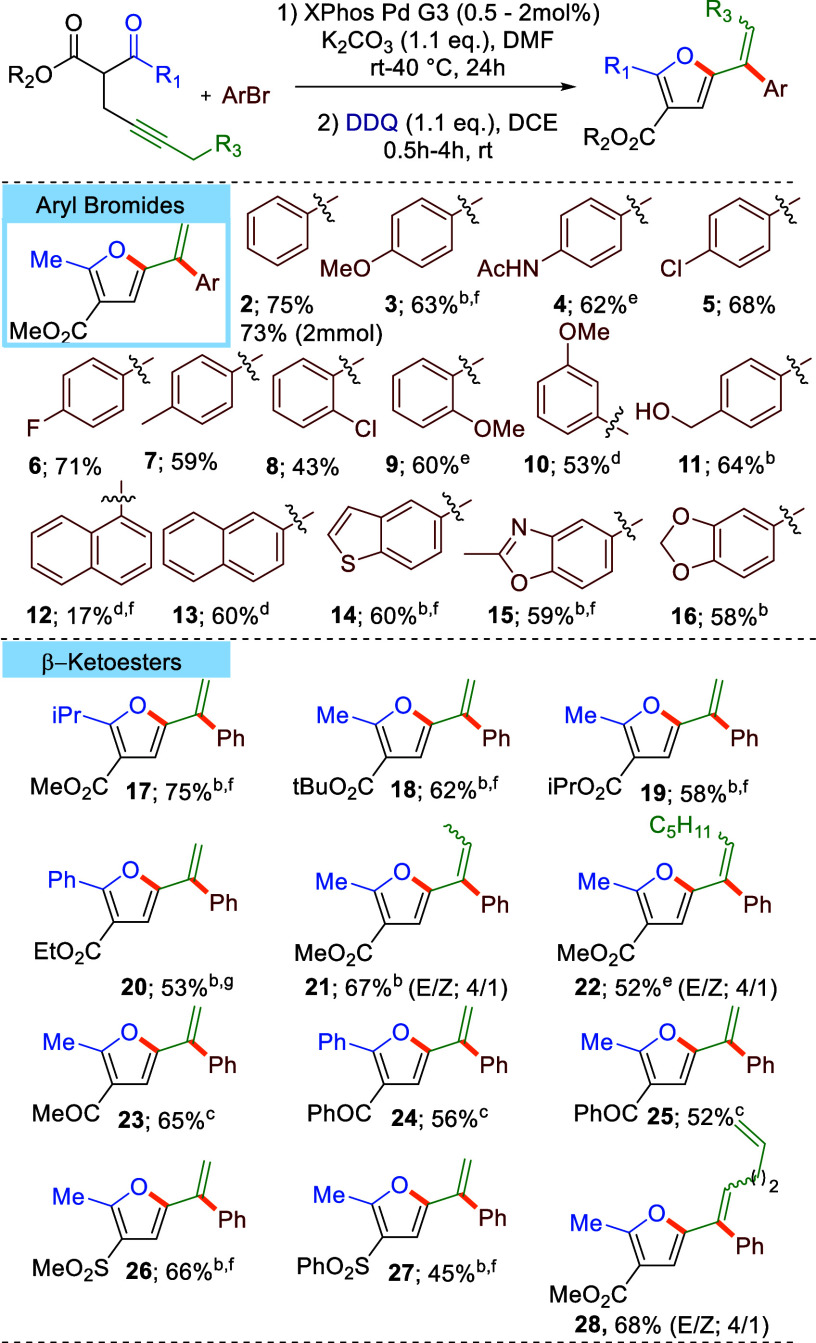
Scope of Synthesized Products

a Reaction conditions: dicarbonyl compound (0.40 mmol), PhBr (0.44 mmol), XPhos Pd G3 (1.69 mg, 2.00 μmol), K_2_CO_3_ (0.44 mmol) DMF (1 mL), 24 h, rt, after that adding DDQ (0.44 mmol), DCE (1 mL), 0.5 h rt.

b 40 °C in the first stage.

c XPhos Pd G3 (3.39 mg, 4.00 μmol).

d 40 °C in the first stage and XPhos Pd G3 (3.39 mg, 4.00 μmol).

e 40 °C in the first stage and XPhos Pd G3 (6.78 mg, 8.00 μmol).

f Stirred for 4 h in the second stage,

g 2 mL of DCE added at second stage.

DDQ is one of the most widely used reagents for a variety of oxidative transformations, including dehydrogenations.^10^ Although exploited for years, the mechanistic aspects of DDQ-driven transformations are still an elusive feature of its reactivity, with Single Electron Transfer (SET) followed by Hydrogen Atom Transfer (HAT)^10c,11^ frequently proposed alongside other reports of the Hydride Transfer (HT),^10c,12^ and Addition–Elimination^11b,13^ pathways, depending on the nature of the substrate and reaction’s environment. To understand the reaction mechanism of the DDQ-driven oxidative-aromatization of 2-benzylidene-dihydrofurans in this study, we performed a set of experimental and theoretical investigations. First, to probe the potentially radical nature of the process, a radical-clock experiment involving compound **29** was designed and performed (Scheme 2a). Only compound **28** formed, with no traces of compound **30**, resulting from cyclization of a potential radical on the tethered olefin, observed. Furthermore, if the reaction of **1** was performed in methanol, then methyl ether **31** was isolated as the main product (Scheme 2b). Formation of **31** can be ascribed to trapping of the benzyl cation intermediate with solvent. These two control experiments point toward ionic (hydride transfer), rather than a radical, mechanism.

**Scheme 2:**
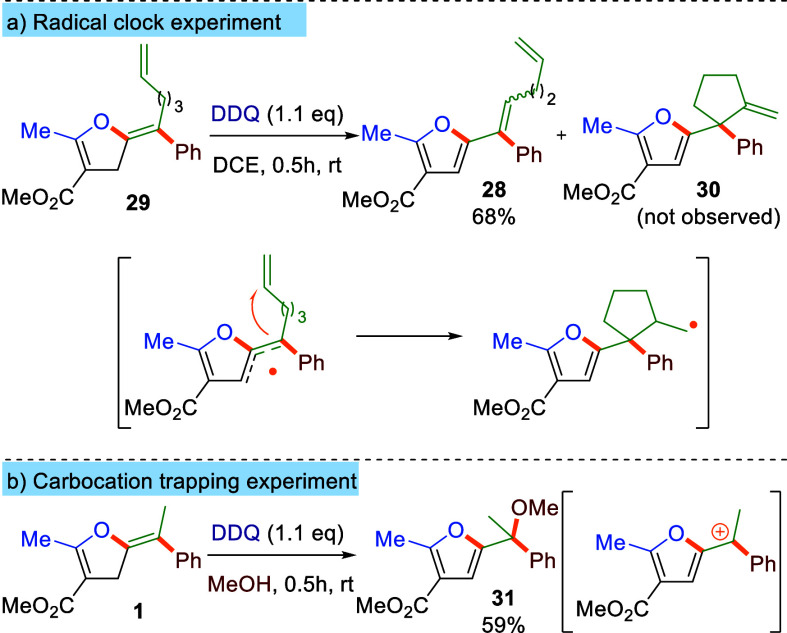
Radical/Carbocation Scavenging Experiments

We also considered that 2-benzylidene-dihydrofuran may act as a π-nucleophile, as proposed by Mayr for reaction of DDQ with silyl enol ethers and other related electron-rich olefins. However, in the model reaction of **1** we did not observe formation of putative O- or C- adducts with DDQ, neither by NMR nor by UV–vis spectroscopy.

To determine from which position hydride transfer occurs, the reactivity of **1** was compared in competition experiments with its deuterated analogues **32** and **33** (Scheme 3). Kinetic isotope effects of 1.3 and 2.1 were found for compounds deuterated on the exocyclic olefin fragment (**32**) and the furan ring (**33**), respectively. The latter value, fitting within the primary KIE for early TS, points toward hydride transfer from the heterocyclic ring leading to formation of a benzylic carbocation intermediate in the rate determining step. This is in line with secondary KIE observed for **32** and the control experiment presented in Scheme 2.

**Scheme 3:**
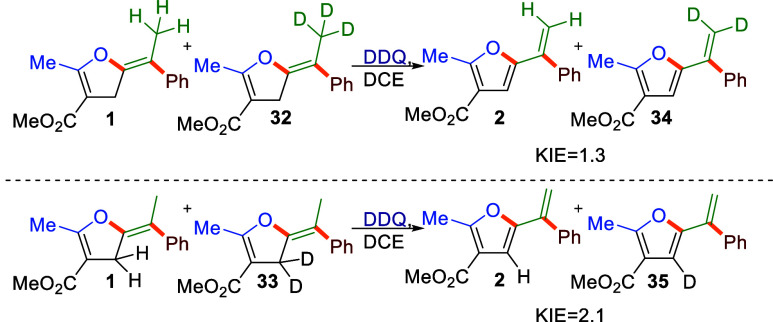
Kinetic Isotope Effect Measurements

Finally, various mechanistic scenarios were also investigated computationally. Various plausible reaction scenarios were considered and are depicted in Figure 1. The most favorable path (depicted in black) involves a rate determining hydride transfer from the substrate to DDQ, which is consistent with control experiments and literature accounts on mechanistic aspects of related benzylic and allylic oxidations with DDQ.^12^ Both hydride transfer in the first step and the overall dehydration process are highly exergonic (Δ*G* = −56.1 and −137.5 kJ/mol, respectively). This contrasts with the hypothetical radical hydrogen transfer which was calculated to be highly endergonic (Δ*G* = 121.1 kJ/mol). In the preferred path, the initial formation of charge-transfer complex **IM1** between **1** and DDQ is followed by O-attack of DDQ on the methylene moiety in the heterocyclic ring of **1**. This exergonic step (Δ*G* = −56.1 kJ/mol), proceeding through a transition state **TS1**, is associated with a small barrier (Δ*G*^‡^ = 61.8 kJ/mol). Then, facile deprotonation of benzylic carbocation by DDQH^–^ (**TS2**, Δ*G*^‡^ = 12.1 kJ/mol) results in charge-transfer complex **IM3** of vinylfuran with DDQH_2_, which spontaneously dissociates. The alternative path involving hydride transfer to the C-center of DDQ through a transition state **TS3** is less favorable (Δ*G*^‡^ = 79.5 kJ/mol). It could be also considered that that the hydride transfer occurs first from an allylic position in the side chain via **TS4**; however, it is even less accessible (Δ*G*^‡^ = 102.8 kJ/mol). Recently, Mayr postulated addition of DDQ to electron rich olefins (e.g., silyl enol ethers), which can trigger further reactivity of the adduct via inner-sphere processes.^13b^ Such a manifold is, however, hardly reachable for **1**, featuring high barriers of 94.7 and 123.2 kJ/mol for **TS5** and **TS6**, corresponding to C- and O-attack of DDQ, respectively.

**Figure 1:**
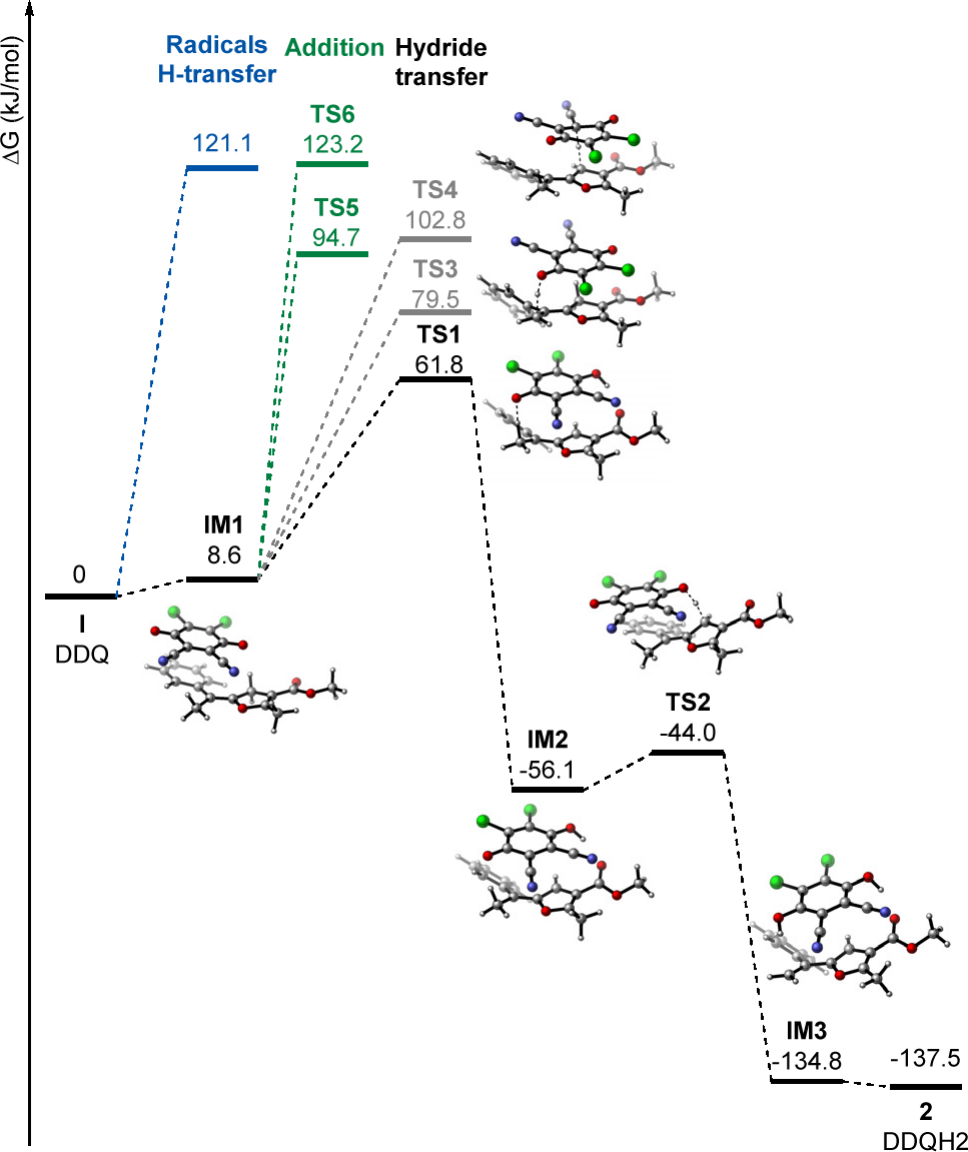
Gibbs free-energy profile for plausible mechanistic scenarios for oxidation of 1 with DDQ. Calculated at SMD(DCE)/M06-2X/6-311++G(d,p)//M06-2X/6-31G(d).

In conclusion, a one-pot cascade transformation merging the Pd-catalyzed reaction of α-propargylic-β-ketoesters and (hetero)aryl halides with the unprecedented oxidative aromatization of the resulting 2-benzylidene-dihydrofuran with DDQ is established. A modular approach enables efficient construction of highly substituted 2-vinyl furans directly from simple acyclic building blocks—propargyl-substituted activated carbonyl compounds and aryl halides. Mechanistic studies, including DFT calculations, of the oxidative transformation point toward a pathway involving hydride transfer from the dihydrofuran ring to the O-center of DDQ as the key step.

## Experimental Section

### General Reaction Procedure for One-Pot Synthesis of 2-Alkenyl-3,5-furans

In a glovebox, to a 4 mL glass screw-capped vial containing XPhos Pd G3 (1.69 mg, 2.0 μmol) and the following reagents were added K_2_CO_3_ (60.8 mg, 0.44 mmol), aryl bromide (0.44 mmol), dicarbonyl compound (0.4 mmol), and DMF (1 mL). Then, a magnetic stirring bar was added and the vial was sealed with a cap containing a PTFE septum. The reaction mixture was stirred at room temperature for 24 h. After that time, the vial was opened at air atmosphere, DCE (1 mL) was added, and the mixture was stirred for a while to evenly mix the solvents. Next, DDQ (100.0 mg, 0.44 mmol) was added and the vial was again sealed. The reaction mixture was stirred at room temperature for a given time. Then, a mixture was quenched with saturated NaHCO_3_ solution (20 mL) and water (10 mL), extracted with DCM (3 × 20 mL), dried (Na_2_SO_4_), and concentrated, and crude product was purified by column chromatography on silica gel.

### Methyl 2-Methyl-5-(1-phenylvinyl)furan-3-carboxylate (2)

Prepared in a reaction of methyl 2-acetylhex-4-ynoate (67.3 mg, 0.40 mmol) with bromobenzene (69.9 mg, 0.44 mmol) under general conditions, with a second stage lasting for 0.5 h. The title compound was isolated as a yellowish oil (72.5 mg, 0.30 mmol, 75%) after chromatography on silica gel (15 g column, hexane:ethyl acetate 98:2); ^1^H NMR (400 MHz, CDCl_3_) δ 7.46–7.40 (m, 2H), 7.39–7.34 (m, 3H), 6.42 (s, 1H), 5.76–5.71 (m, 1H), 5.24 (d, *J* = 1.0 Hz, 1H), 3.80 (s, 3H), 2.64 (s, 3H); ^13^C{^1^H} NMR (101 MHz, CDCl_3_) δ 164.3, 159.3, 151.8, 139.0, 138.6, 128.3, 128.2, 128.1, 114.8, 112.2, 109.6, 51.2, 13.8; IR (CH_2_Cl_2_): 1718 (C = O), 1231 (C–O), 1093 (C–O) cm^–1^ HRMS (EI) *m*/*z*: [M]^+^ Calcd for C_15_H_14_O_3_: 242.0943, Found 242.0951

## Data Availability

The data underlying this study are available in the published article and its Supporting Information.

## Supporting Information Available

Evaluation of reaction conditions, details of optimization and control experiments, experimental procedures, characterization of all compounds, copies of ^1^H and ^13^C{^1^H} NMR spectra of isolated compounds, and details of computational studies (PDF)
