# Supplementary material for: Access to 2-Alkenyl-furans via a Cascade of Pd-Catalyzed Cyclization/Coupling Followed by Oxidative Aromatization with DDQ
Source: J Org Chem. 2024 May 3;89(10):7275–9. doi: 10.1021/acs.joc.4c00149 (PMC11110045; doi:10.1021/acs.joc.4c00149)
Supplement: Supplementary file 1 — jo4c00149_si_001.pdf [file jo4c00149_si_001.pdf]

Supporting information

Access to 2-alkenyl-furans via a cascade of Pd-catalyzed cyclization/coupling followed by oxidative aromatization with DDQ.

Bartosz Bisek, Wojciech Chaładaj*

Institute of Organic Chemistry, Polish Academy of Sciences, Kasprzaka 44/52, 01-224 Warsaw, Poland
**wojciech.chaladaj@icho.edu.pl*

Table of Contents

Table of Contents S1
General Information S2
Materials S2
Evaluation of reaction conditions for Pd-catalyzed 5-exo-dig cyclization/coupling of internal alkynes with further oxidative-aromatization S3
Control experiments S5
Reaction procedures S12
Analytical data of isolated products S15
Computational Studies S26
Copies of ^{1}H and $^{13}C\{^{1}H\}$ NMR spectra of isolated compounds S42

General Information

All manipulations were performed in a nitrogen-filled glovebox or under an argon atmosphere using Schlenk techniques, unless otherwise noted. Reactions that required heating were put in an aluminum heating block of proper size. Flash chromatography was performed using Merck silica gel 60 (230-400 mesh). TLC analysis of reaction mixtures was performed on Merck silica gel 60 F254 TLC plates and visualized with cerium molybdate stain (Hanessian's stain). ^{1}H, ^{13}C{1H} spectra were recorded with a Bruker AV 400 or Varian Agilent 500/600 spectrometer. UV-VIS spectra were recorded on an Agilent Cary 60 UV-VIS. ^{1}H and ^{13}C chemical shifts are given in ppm relative to TMS. The solvent signals were used as references ($CDCl_3$ δ_H = 7.26 ppm, δ_C = 77.0 ppm) and the chemical shift converted to the TMS scale. Coupling constants (*J*) are reported in Hz, and the following abbreviations were used to denote multiplets: s = singlet, d = doublet, t = triplet, q = quartet, quint = quintet, m = multiplet (denotes complex pattern), dd = doublet of doublets, dt = doublet of triplets and br = broad signal. Infrared spectra were recorded with a Jasco FTIR-6200 spectometer. Electron ionization high-resolution mass spectra (EI-HR) were recorded with an Autospec Premier (Waters Inc) mass spectrometer using the narrow-range high-voltage scan technique with low-boiling perfluorokerosene (PFK) as internal standard. Samples were introduced by using a heated direct insertion probe. Electrospray ionization high-resolution mass spectra (ESI-HR) were recorded with MALDISynapt G2-S HDMS (Waters Inc) mass spectrometer equipped with an electrospray ion source and q-TOF type mass analyzer. ESI-MS spectra were recorded in the positive ion mode (the source parameters: capillary voltage 3.15 kV, sampling cone 25 V, source temperature 120 °C, desolvation temperature 150 °C).

Materials

Unless otherwise noted, all commercially available compounds, including aryl bromides (ABCR, Acros, Fluorochem, TCI, Sigma-Aldrich, Strem) were used as received. Dry solvents were acquired by the use of a solvent purification system (SPS). Buchwald-type 3rd-generation palladacyclic precatalysts (Ligand Pd G3) were prepared following literature procedure[1], and showed similar reactivity to the commercial samples (Sigma-Aldrich). The internal acetylenic β-dicarbonyl used for the synthesis of **2 - 27** were synthesized by a previously reported procedure[3].

Evaluation of reaction conditions for Pd-catalyzed 5-exo-dig cyclization/coupling of internal alkynes with further oxidative-aromatization

General procedure for evaluation of reaction conditions: In a glovebox, to a 4-mL screw-capped vial containing catalyst following reagents ware added: methyl 2-acetylhex-4-ynoate (16.8 mg, 0.1 mmol, 1 equiv), bromobenzene (17.3 mg, 0.11 mmol, 1.1 eq), base (0.11 mmol, 1.1 eq), DMF (0.5 mL). Then, magnetic stirring bar was placed and the vial was sealed with a cap containing Teflon seal. The reaction mixture was stirred at room temperature for 24 h. Vial was opened at air atmosphere. Next, co-solvent (0.5 mL) was added, followed by oxidant. Sample was stirred for given time. The mixture was diluted with DCM (1 mL) quenched with sat. aquas $NaHCO_3$ (1 mL) and mezytylene (15 µl) was added as an internal standard.

1. Oxidative-aromatization optimalization

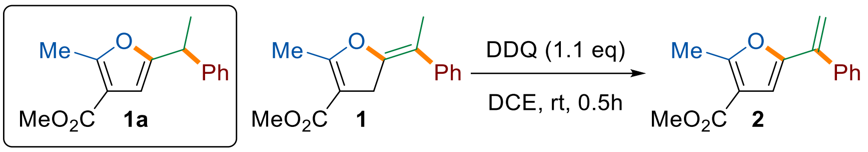

Entry	Variable[a]	Yield 2[b]
**1**	**DCE (1 mL)**	**92%**
2	Furan **1a** as substrate	4%
3	1.5 eq DDQ	29%
4	Toluene (1 mL)	77%
5	THF (1 mL)	63%
6	MeCN (1 mL)	64%
7	DMF (1 mL)	71%

[a]Conditions: **1** (0.4 mmol, 1 eq), DDQ (0.44 mmol, 1.1 eq), DCE (1 mL), rt, 0.5h; [b]isolated yield.

2. Effect of the oxidant

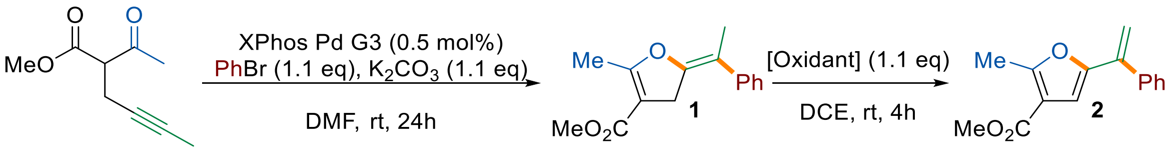

Entry	Oxidant[a]	Yield[b]
**1**	**DDQ**	**88%**
2	Chloranil	63%
3	PBQ	14%
4	ACQ	0%
5	Selectfluor	18%
6	PIFA	10%
7	PhI(OAc)2	26%

[a]Conditions: XPhos Pd G3 (0.5 mol%), methyl 2-acetylhex-4-ynoate (0.1 mmol, 1 eq), bromobenzene (0.11 mmol, 1.1 eq), K_2CO_3 (0.11 mmol, 1.1 eq), DMF (0.5 mL), rt, 24h, in next stage oxidant (0.11 mmol, 1.1 eq), + DCE (0.5 mL), rt, 4h; [b]determined by GC with mezytylene as an internal standard.

3. Effect of co-solvent

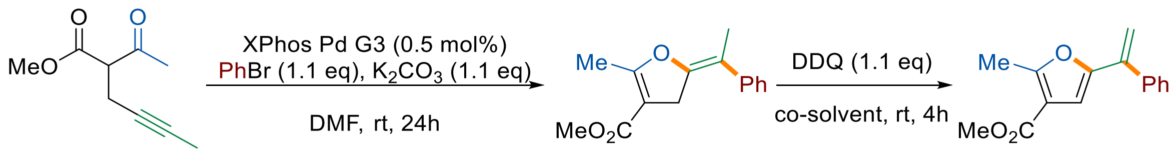

Entry	Co-solvent[a]	Yield[b]
**1**	**DCE (0.5 mL)**	**88%**
2	DCE (1.0 mL)	84%
3	DCE (0.25 mL)	83%
4	DMF (0.5 mL)	74%
5	Toluene (0.5 mL)	84%
6	DCM (0.5 ml)	75%
7	MeCN (0.5 mL)	72%
8	THF (0.5 mL)	78%
9	None	74%

[a]Conditions: XPhos Pd G3 (0.5 mol%), methyl 2-acetylhex-4-ynoate (0.1 mmol, 1 eq), bromobenzene (0.11 mmol, 1.1 eq), K_2CO_3 (0.11 mmol, 1.1 eq), DMF (0.5 mL), rt, 24h, in next stage DDQ (0.11 mmol, 1.1 eq), + co-solvent, rt, 4h; [b]determined by GC with mezytylene as an internal standard.

4. Effect of the reaction time

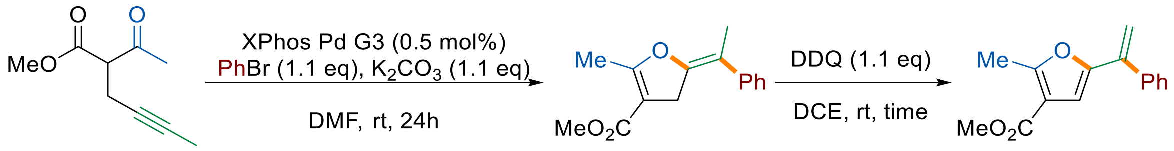

Entry	Time[a]	Yield[b]
1	0.25h	84%
**2**	**0.5h**	**89%**
3	4h	88%

[a]Conditions: XPhos Pd G3 (0.5 mol%), methyl 2-acetylhex-4-ynoate (0.10 mmol, 1 eq), bromobenzene (0.11 mmol, 1.1 eq), K_2CO_3 (0.11 mmol, 1.1 eq), DMF (0.5 mL), rt, 24h, in next stage DDQ (0.11 mmol, 1.1 eq), + DCE (0.5 mL), rt; [b]determined by GC with mezytylene as an internal standard.

Control experiments

1. Reaction with TEMPO

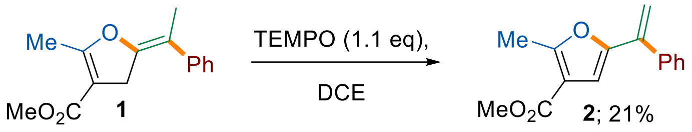

To the mixture of **1** (0.1 mmol, 1.0 eq) in DCE (0.5 mL), TEMPO (0.11 mmol, 0.11 eq) was added in one portion. Mixture was stirred at rt. for 4h after which reaction was quenched with $NaHCO_3$ (1 mL) and water (1 mL). DCM (2 mL) and mezytylene (15 µl) were added. Yield was determined via GC with mezytylene as internal standard.

2. Radical clock experiment

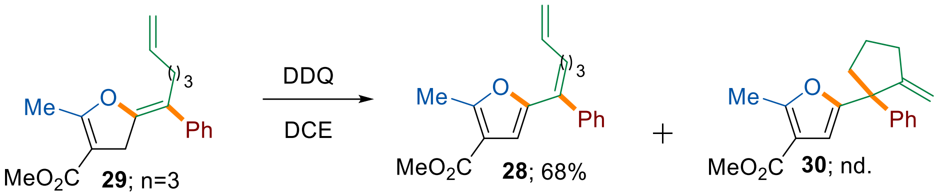

In a glovebox, to a 4-mL glass screw-capped vial containing DDQ (100.0 mg, 0.44 mmol) 2-benzylidene-dihydrofuran **29** (0.4 mmol) was added followed by DCE (1.0 mL). Then, magnetic stirring bar was placed and the vial was sealed with a cap containing a PTFE septum. The reaction mixture was stirred at room temperature for 0.5h. Then, mixture was quenched with saturated $NaHCO_3$ solution (20 ml) and water (5 ml), extracted with DCM (3x20 ml), dried (Na_2SO_4), concentrated and crude product was purified by column chromatography on silica gel.

2a. Radical clock experiment - benchmark reaction

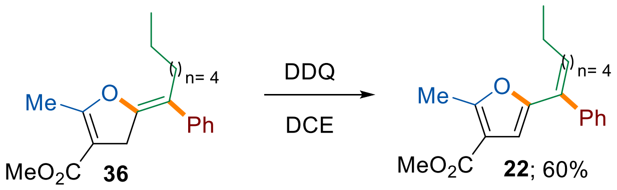

In a glovebox, to a 4-mL glass screw-capped vial containing DDQ (100.0 mg, 0.44 mmol) 2-benzylidene-dihydrofuran **36** (0.4 mmol) was added followed by DCE (1.0 mL). Then, magnetic stirring bar was placed and the vial was sealed with a cap containing a PTFE septum. The reaction mixture was stirred at room temperature for 0.5h. Then, mixture was quenched with saturated $NaHCO_3$ solution (20 ml) and water (5 ml), extracted with DCM (3x20 ml), dried (Na_2SO_4), concentrated and crude product was purified by column chromatography on silica gel.

3. Carbocation scavenger experiment

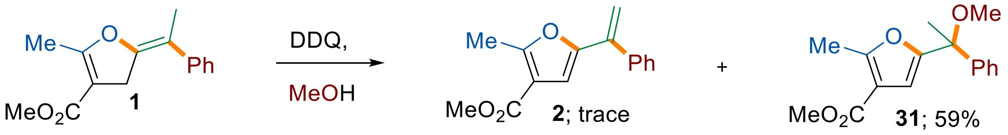

In a glovebox, to a 4-mL glass screw-capped vial containing DDQ (100.0 mg, 0.44 mmol) 2-benzylidene-dihydrofuran **1** (0.4 mmol) was added followed by MeOH (1.0 mL). Then, magnetic stirring bar was placed and the vial was sealed with a cap containing a PTFE septum. The reaction mixture was stirred at room temperature for 0.5h. Then, mixture was quenched with saturated $NaHCO_3$ solution (20 ml) and water (5 ml), extracted with DCM (3x20 ml), dried (Na_2SO_4), concentrated and crude product was purified by column chromatography on silica gel.

4. Reaction monitoring on UV-VIS

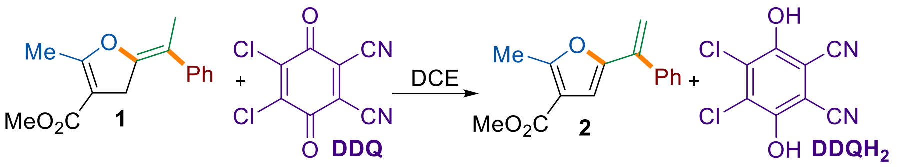

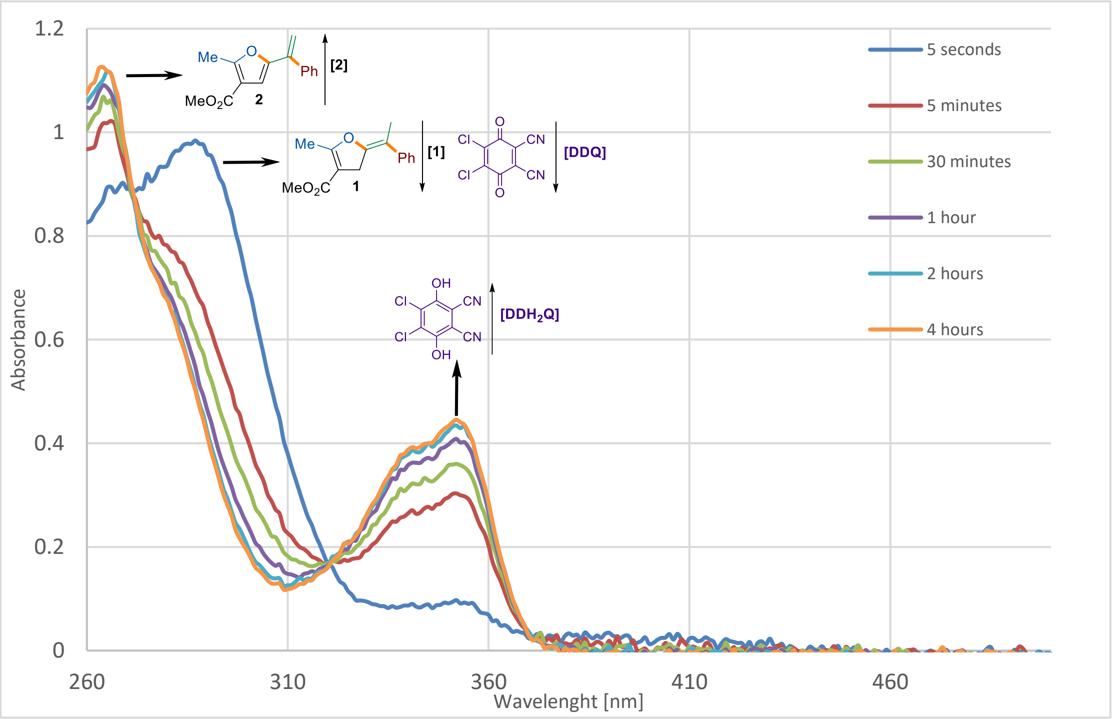

To a 2.4-mL glass measuring cuvette containing DCE, 2-benzylidene-dihydrofuran **1** ($6x10^{-5}$ mmol, 1.0 eq) was added from standard solution (**1,** 10^{-2} mmol in DCE) followed by DDQ ($6x10^{-5}$ mmol, 1.0 eq) added from standard solution (DDQ, 10^{-2} mmol in DCE). The reaction mixture was monitored on UV-VIS apparatus at room temperature for 4h.

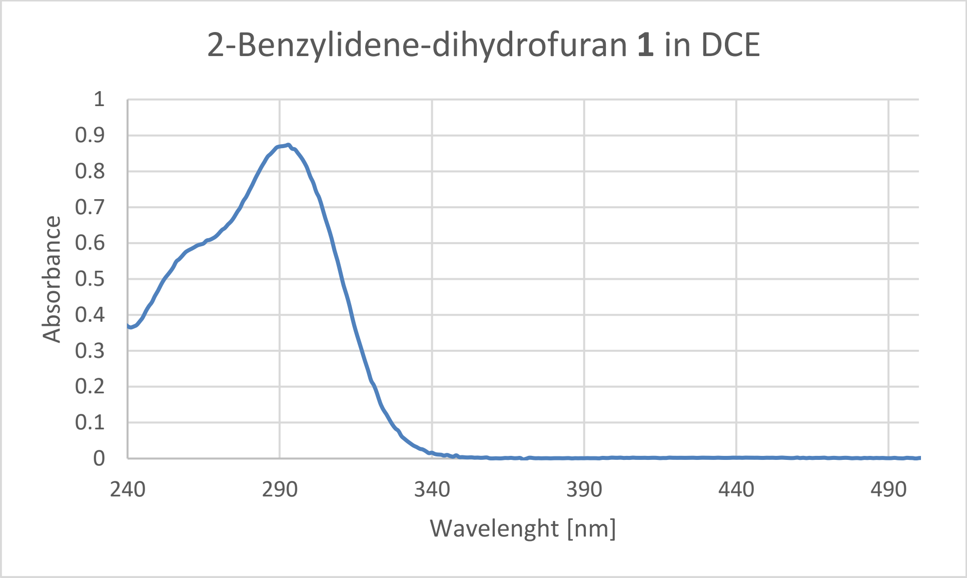

1 (6x10^{-5} mmol), DCE (2.4 mL)

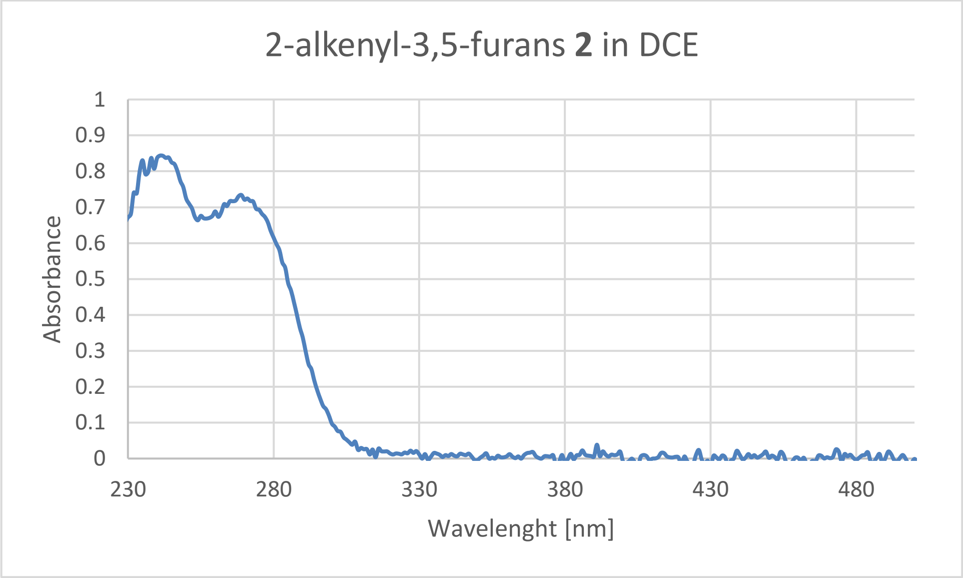

2 (6x10^{-5} mmol), DCE (2.4 mL)

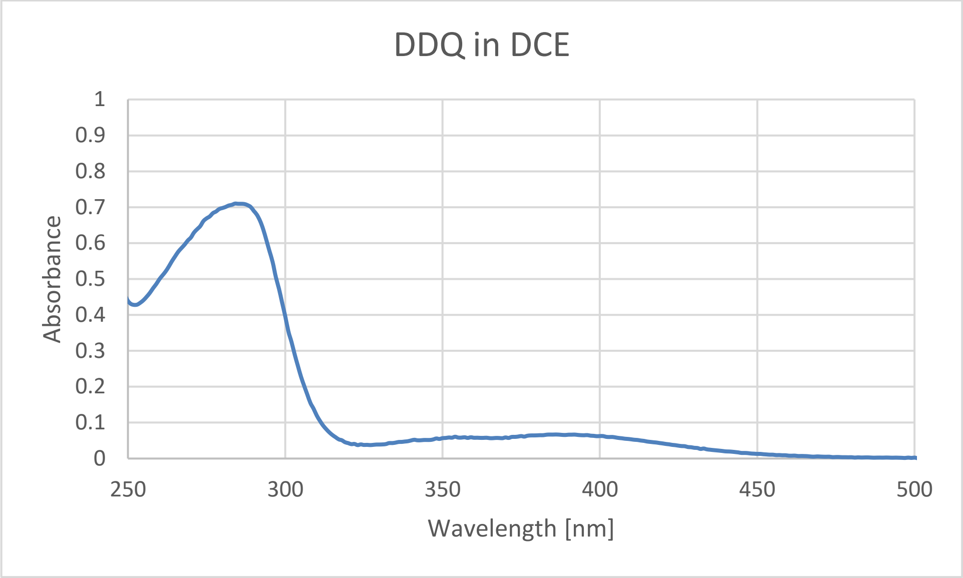

DDQ (6x10^{-5} mmol), DCE (2.4 mL)

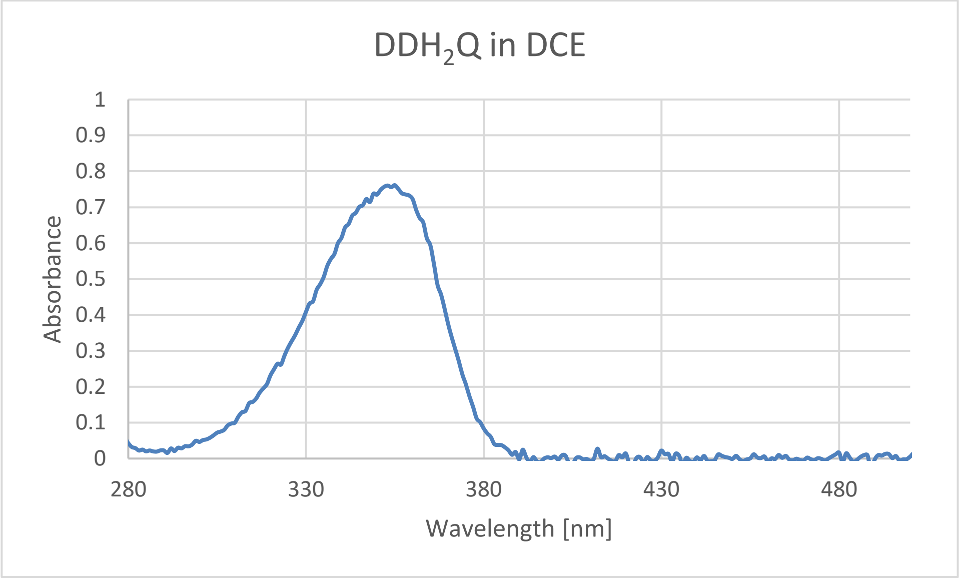

DDQH$_2$ (6x10^{-5} mmol); prepared from solution in DMF (10^{-2} mmol), DCE (2.4 mL)

5. KIE measurements

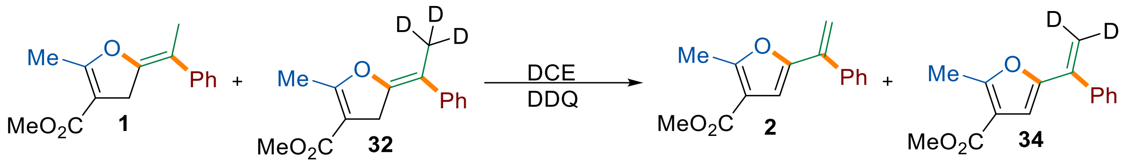

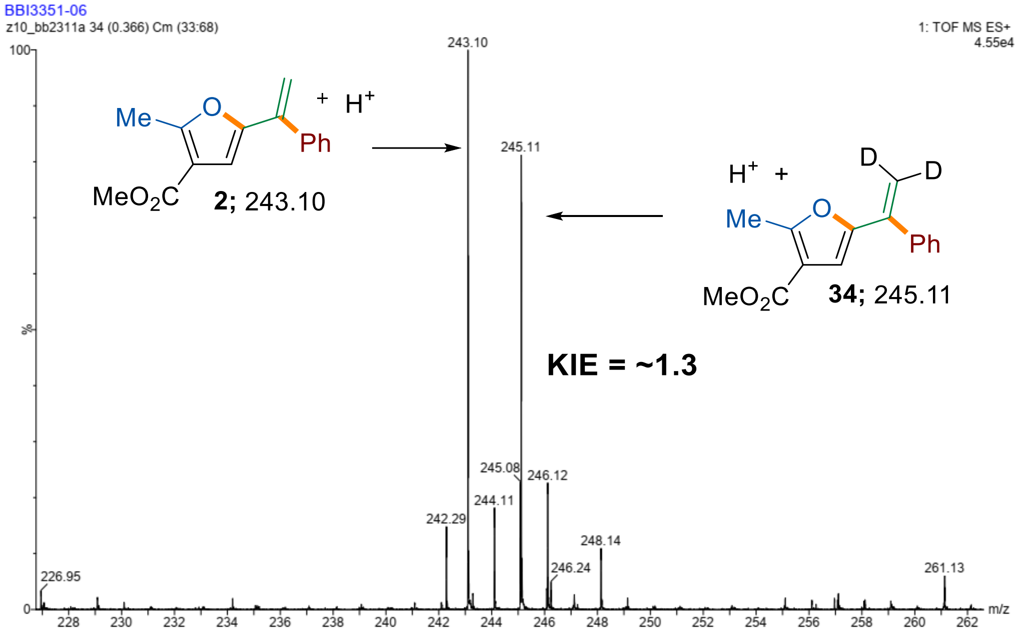

To the equimolar mixture of **1** (0.4 mmol, 1.0 eq) and **32** (0.4 mmol, 1.0 eq) in DCE (1.0 mL), DDQ (0.04 mmol, 0.1 eq) was added in one portion. Mixture was stirred at rt. for 0.5h after which reaction was quenched with $NaHCO_3$ (1 mL) and water (10 mL). DCM (15 mL) was added, phases were separated and water phase was extracted with DCM (2x15 mL). The combined organic phases were dried over Na_2SO_4, filtered, and evaporated. The mixture of 2-alkenyl-3,5-furans was purified by column chromatography (15 g of silica gel; 99:1 Hex:Oct). KIE were estimated by known method with ESI/MS from the ratios of peak intensities[2] and ratios of peak areas with assumption that spraying and ionization of deuterated and non-deuterated compounds are similar.

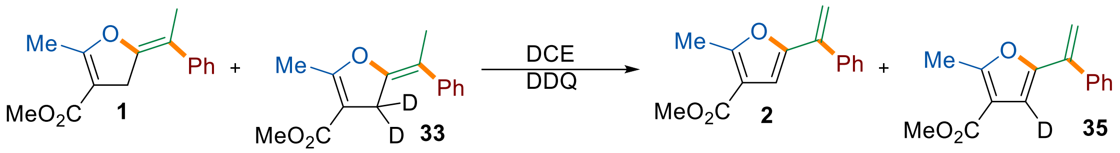

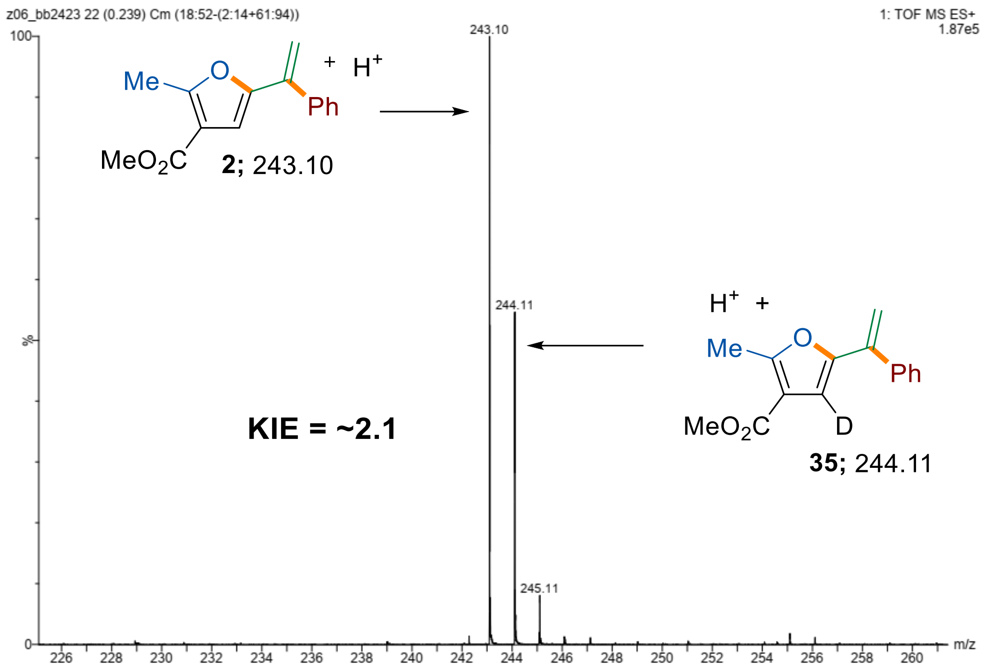

To the equimolar mixture of **1** (0.4 mmol, 1.0 eq) and **33** (0.4 mmol, 1.0 eq) in DCE (1.0 mL), DDQ (0.04 mmol, 0.1 eq) was added in one portion. Mixture was stirred at rt. for 0.5h after which reaction was quenched with $NaHCO_3$ (1 mL) and water (10 mL). DCM (15 mL) was added, phases were separated and water phase was extracted with DCM (2x15 mL). The combined organic phases were dried over Na_2SO_4, filtered, and evaporated. The mixture of 2-alkenyl-3,5-furans was purified by column chromatography (15 g of silica gel; 99:1 Hex:Oct). KIE were estimated by known method with ESI/MS from the ratios of peak intensities[2] and ratios of peak areas with assumption that spraying and ionization of deuterated and non-deuterated compounds are similar. During KIE calculations for compounds 2/35 isotopic distribution was taken into account, as predictive model suggests 16.2% of M=244.11 arises from compound 2.

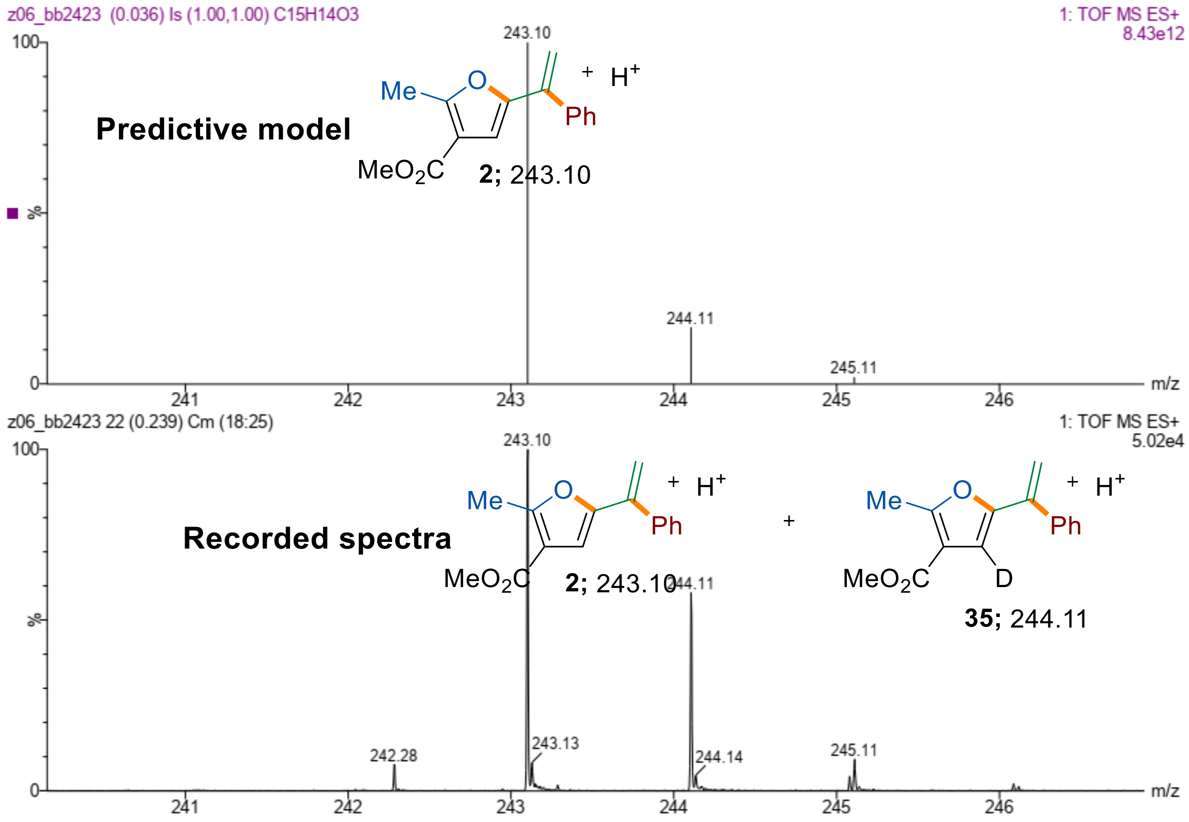

The KIE result obtained from the peak intensities ratio of **2**/**35** was compared with the results acquired from the area ratio of **2**/**35**, giving similar results.

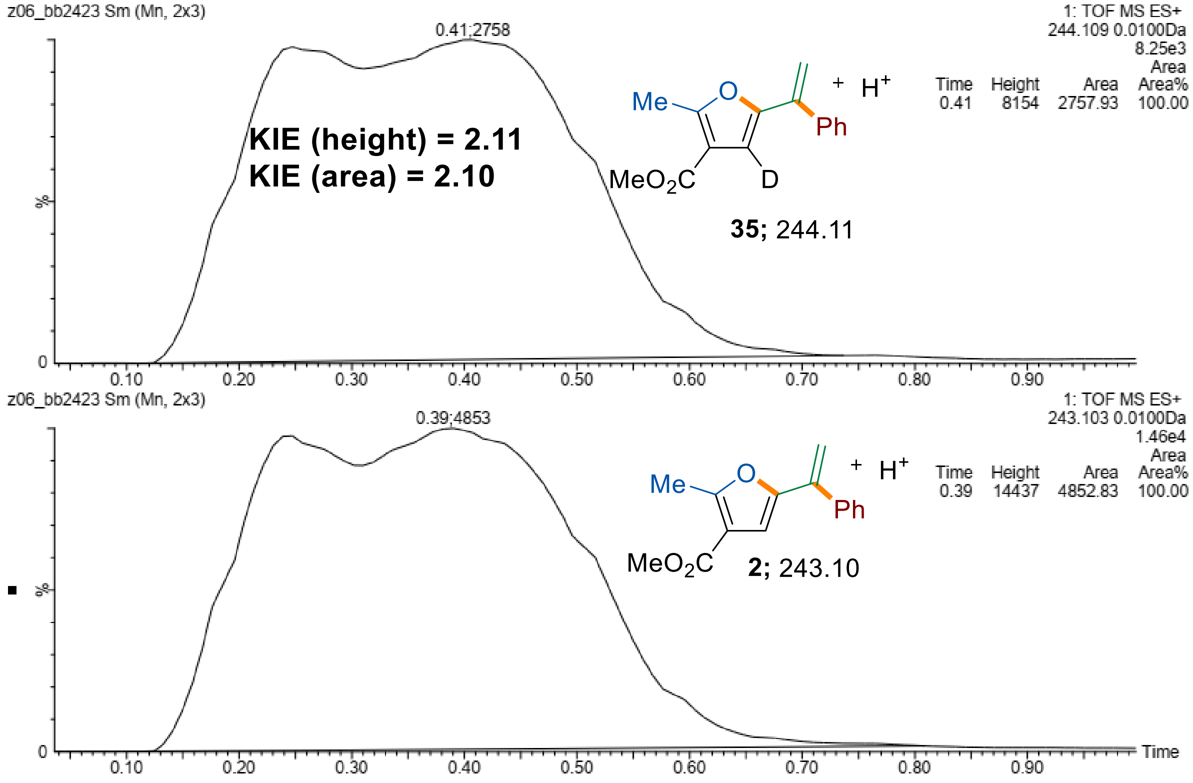

Reaction procedures

One-pot synthesis of 2-alkenyl-3,5-furans from internal alkynes

Conditions A: In a glovebox, to a 4-mL glass screw-capped vial containing XPhos Pd G3 (1.69 mg, 2.0 μmol) and the following reagents were added: K_2CO_3 (60.8 mg, 0.44 mmol), aryl bromide (0.44 mmol), dicarbonyl compound (0.4 mmol), DMF (1 ml). Then, magnetic stirring bar was placed and the vial was sealed with a cap containing a PTFE septum. The reaction mixture was stirred at room temperature for 24 h. After that time, the vial was opened at air atmosphere, DCE (1 ml) was added and the mixture stirred for a while to evenly mix the solvents. Next, DDQ (100.0 mg, 0.44 mmol) was added and the vial was again sealed. The reaction mixture was stirred at room temperature for a given time. Then, mixture was quenched with saturated $NaHCO_3$ solution (20 ml) and water (10 ml), extracted with DCM (3x20 ml), dried (Na_2SO_4), concentrated and crude product was purified by column chromatography on silica gel.

Procedure for reaction run at gram scale for compound 2: In a glovebox to a 25-mL, vacuum dried schlenk flask containing XPhos Pd G3 (8.46 mg, 10 μmol) following reagents were added: K_2CO_3 (0.3 g, 2.2 mmol), bromobenzene (0.34 g, 2.2 mmol), methyl 2-acetylhex-4-ynoate (0.34 g, 2.0 mmol), DMF (55 ml) and the reaction mixture was stirred at room temperature for 24 h. After that time, the vial was opened at air atmosphere, DCE (5 ml) was added and the mixture stirred for a while to evenly mix the solvents. Next, DDQ (0.45 g, 2.0 mmol) was added and the vial was again sealed. The reaction mixture was stirred at room temperature for a 2 hours. The mixture was quenched with NH_4Cl solution (20 ml) and water (10 ml), extracted with DCM (3x30 ml), dried (Na_2SO_4), concentrated and crude product was purified by column chromatography on silica gel.using hexane/ethyl acetate 98:2 as an eluent. Compound **2** was isolated as yellowish oil (0.35 g, 1.45 mmol, 73 %).

Synthesis of 2-benzylidene-dihydrofurans: 28, 36

Conditions B: In a glovebox, to a 4-mL glass screw-capped vial containing XPhos Pd G3 (6.77 mg, 8.0 μmol) and following reagents were added: K_2CO_3 (121.6 mg, 0.88 mmol), aryl bromide (0.88 mmol), dicarbonyl compound (0.80 mmol), DMF (2 ml). Then, magnetic stirring bar was placed and the vial was sealed with a cap containing a PTFE septum. The reaction mixture was stirred at room temperature for 24 h. Then, mixture was quenched with saturated NH_4Cl solution (15 ml) and water (5 ml), extracted with MTBE (3x15 ml), dried (Na_2SO_4), concentrated and crude product was purified by column chromatography on silica gel.

Synthesis of 2-alkenyl-3,5-furans: 22, 29

Conditions C: In a glovebox, to a 4-mL glass screw-capped vial containing DDQ (100.0 mg, 0.44 mmol) 2-benzylidene-dihydrofuran (0.4 mmol) was added followed by DCE (1.0 mL). Then, magnetic stirring bar was placed and the vial was sealed with a cap containing a PTFE septum. The reaction mixture was stirred at room temperature for 0.5h. Then, mixture was quenched with saturated $NaHCO_3$ solution (20 ml) and water (5 ml), extracted with DCM (3x20 ml), dried (Na_2SO_4), concentrated and crude product was purified by column chromatography on silica gel.

Synthesis of internal acetylenic β-dicarbonyl compounds

The internal acetylenic β-dicarbonyl used for the synthesis of **2 - 27** were synthesised by a previously reported procedure[3]

Conditions D: A 50-mL Schlenk tube containing 60% NaH (408.0 mg, 10.2 mmol) and stirring bar was evacuated and backfilled with argon three times. Then THF (30 ml) was added and mixture was cooled down to 0 °C. Then dicarbonyl compound (10.0 mmol) was slowly added. Resulting mixture

The mixture was stirred for approximately 30 minutes, until the hydrogen bubbles were not visible. Then alkyne bromide (10.2 mmol) was added. The reaction mixture was heated to 60°C, stirred for 24h, and then cooled toroom temperature. The mixture was quenched with NH4Cl solution (30 ml) and water (10 ml) , extracted with MTBE (3x30 ml), dried (Na2SO4), concentrated and crude product was purified by column chromatography on silica gel.

Conditions E: A 50-mL Schlenk tube containing K2CO3 (373.0 mg, 2.7 mmol) and stirring bar was evacuated and backfilled with argon three times. Then, acetone (25 ml) was added, followed by dicarbonyl compound (5.0 mmol). Resulting mixture was stirred for 10 minutes. Then alkyne bromide (2.5 mmol) was added. Reaction was heated to 60°C, stirred for 24h, then cooled to room temperature.

The mixture was filtered through celite and concentrated. Crude product was purified by column chromatography on silica gel.

Synthesis of substrates used in transformations yielding 22, 29

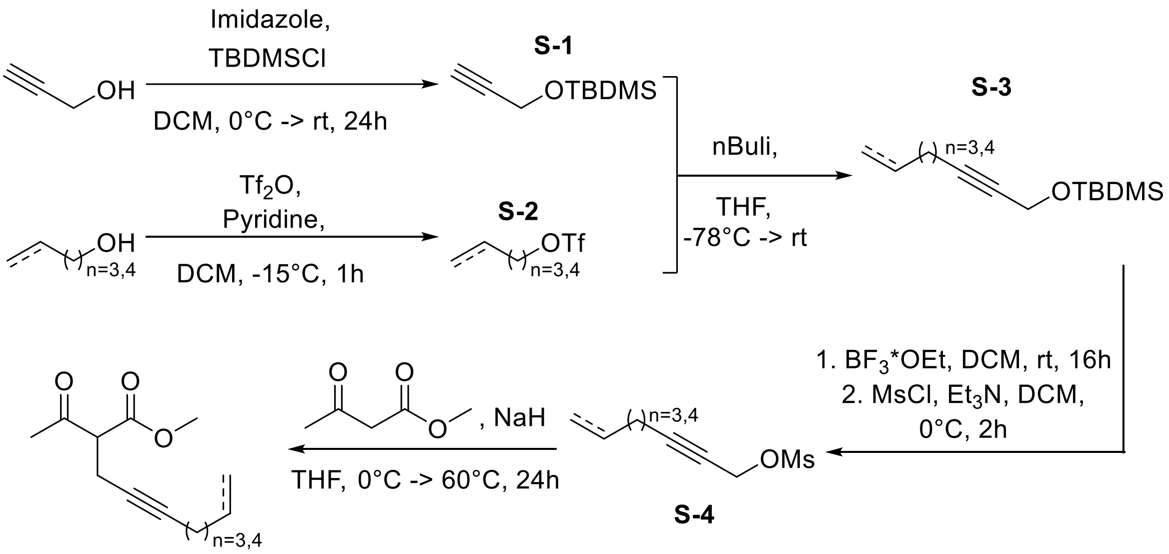

Conditions F:

To 250 mL round bottom flask containing DCM (100 mL) and stirring bar, Imidazole was added (60.0 mmol). Then mixture was cooled to 0°C followed by addition of propargyl alcohol (40.0 mmol) and TBDMSCl (48.0 mmol). The reaction mixture was allowed to warm to room temperature and stirred for approximately 24h. The mixture was quenched with $NaHCO_3$ (10 mL) and water (100 mL). Phases were separated, the water phase was extracted with 3x30 mL of DCM. The combined organic phase was dried over Na_2SO_4, filtered and concentrated. **S-1** was distilled from this mixture (20 mbar, 52°C).

S-2 were prepared according to literature procedure[4]. To 500 mL round bottom flask containing DCM (100 mL) and stirring bar, (un)saturated alcohol (24.0 mmol) and pyridine was added, followed by slow addition of triflate anhydride in -15°C. Mixture was stirred at this temperature for approx. 1h, after which hexane was added into it (200 mL) and mixture was filtered through celite pad, followed by mixture concentration on rotary evaporator at room temperature for approx. 1h, yielding **S-2**. During this time another 100 mL round bottom flask was prepared by adding dry THF (40 mL), **S-1** (20.0 mmol), followed by slow addition of nBuLi 2.5M in hexanes (22.0 mmol) at -78°C. Mixture was stirred

at -78°C during **S-2** concentration to ensure full deprotonation. Next, crude **S-2** (approx.. 24.0 mmol) was slowly added at -78°C to the mixture containing deprotonated **S-1**. The reaction mixture was allowed to slowly warm up to rt. and left overnight. After that time, the mixture was quenched with NH_4Cl_{aq} (20 mL) and water (10 mL). Phases were separated, the water phase was extracted with MTBE 3x20 mL. The combined organic phases were dried over Na_2SO_4. The resulting **S-3** was isolated as yellowish oil after chromatography on silica gel (250 g column, Hexane:Ethyl Acetate 99:1).

To 100 mL round bottom flask, containing dry DCM (60 mL) and stirring bar **S-3** (11.0 mmol) was added followed by addition of BF_3*OEt (22.0 mmol). Mixture was stirred for approx. 16h at room temperature. After this time, water (20 mL) was added to the mixture. Organic phase was isolated, water phase was extracted with DCM 3x20 mL. Combined organic phases were dried over Na_2SO_4 and filtered. The mixture was concentrated in a rotary evaporator to approx. 60 mL of DCM, which was cooled to 0°C and treated with Et_3N (16.5 mmol). After 10 minutes, MsCl (17.6 mmol) was slowly added to this mixture at 0°C. The reaction mixture was stirred at this temperature for approx. 2h. The reaction was quenched with the addition of 7 mL of 1M HCl, followed by the addition of $NaHCO_3$ solution (30 mL). Organic phase was separated. Water phase was extracted with DCM 3x20 mL. Combined organic phases were dried over Na_2SO_4, filtered through a wet (DCM) silica plug that was additionally rinsed with DCM (300 mL) and dried over rotary evaporator yielding **S-4**, which was used without further purification for keto-ester synthesis within **Conditions D**.

Synthesis of deuterated keto-esters

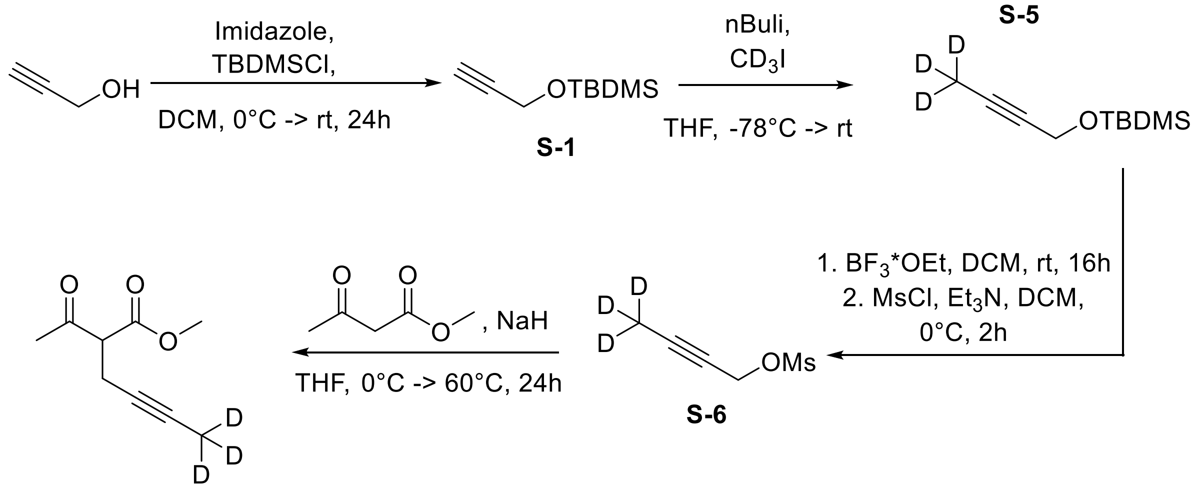

methyl 2-acetylhex-4-ynoate-6,6,6-*d*$_3$

methyl 2-acetylhex-4-ynoate-6,6,6-d3 was synthesized in a multi-step transformation.

To 250 mL round bottom flask containing DCM (100 mL) and stirring bar, imidazole was added (60.0 mmol). Then the mixture was cooled to 0°C followed by the addition of propargyl alcohol (40.0 mmol) and TBDMSCl (48.0 mmol). The reaction mixture was allowed to warm up to room temperature and stirred for approx. 24h. The mixture was quenched with $NaHCO_3$ (10 mL) and water (100 mL). Phases were separated, the water phase was extracted with DCM 3x30 mL. The combined organic phase was dried over Na_2SO_4, filtered and concentrated. **S-1** was distilled from this mixture (20 mbar, 52°C).

To 100 mL round bottom flask containing THF (40 mL), **S-1** (20.0 mmol) was added, followed by slow addition of nBuLi 2.5M in hexanes (22.0 mmol) at -78°C. Mixture was stirred at -78°C for approx. 1h. After this time, CD_3I (30.0 mmol) was carefully added to the reaction mixture. The mixture was allowed to warm up to rt and stirred overnight. The mixture was quenched with $NaHCO_3$ (5 mL) and water (30 mL). The phases were separated. The water phase was extracted with MTBE 3x20 mL, dried over

Na_2SO_4, evaporated at 300 mbar 40°C. **S-5** was isolated as colorless oil (16.6 mmol, 83%) by distillation (10 mbar, 65°C).

To 100 mL round bottom flask, containing dry DCM (40 mL) and stirring bar **S-5** (8.0 mmol) was added followed by addition of BF_3*OEt (16.0 mmol). The mixture was stirred for approximately 16h at room temperature. After this time, water (16 mL) was added to the mixture. The organic phase was isolated, the water phase extracted with DCM 2x10 mL. Combined organic phases were dried over Na_2SO_4 and filtered. The next stage of transformation was carried out with the mixture obtained with approx. 70 ml of DCM within. The reaction mixture was cooled to 0°C, followed by the addition of Et_3N (12.0 mmol). After 10 minutes MsCl (12.8 mmol) was slowly added at 0°C. Reaction mixture was stirred at that temperature for approximately 2 hours. The reaction was stopped with the addition of 4 ml of 1M HCl, followed by the addition of $NaHCO_{3aq}$ (20 mL). The organic phase was separated. The water phase was extracted with 3x20 ml of DCM. Combined organic phases were dried over Na_2SO_4, filtered and concentrated. Crude **S-6** (6.3 mmol, 79%) was used without further purification for **methyl 2-acetylhex-4-ynoate-6,6,6-*d₃*** synthesis within **Conditions D**.

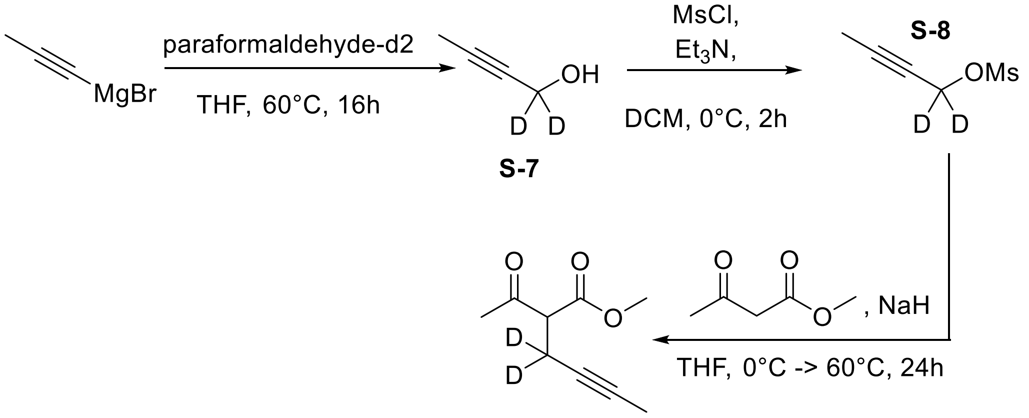

methyl 2-acetylhex-4-ynoate-3,3-*d*$_2$

methyl 2-acetylhex-4-ynoate-3,3-d2 was synthesized in a multi-step transformation.

To a dry two-neck round bottom flask with stirring bar propynylmagnesium bromide 0.5M in THF (10 mmol) was added under an atmosphere of argon, followed by the addition of paraformaldehyde-d2 (8.0 mmol). The mixture was heated to 60 ° C and stirred at this temperature for 16 h. The reaction was then quenched with saturated NH_4Cl (10 mL) and water (10 mL) extracted with DCM 3x15 mL. Combined organic phases were dried over Na_2SO_4 and filtered. The resulting solution containing **S-7** was used in the next step without further purification. To this mixture, containing a stirring bar, Et_3N (9.6 mmol) was added at 0°C, followed by slow addition of MsCl (10.4 mmol) at the same temperature. The mixture was stirred at 0 ° C for 2 h. The reaction was stopped with the addition of 4 ml of 1M HCl, followed by the addition of $NaHCO_{3aq}$ (20 mL). The organic phase was separated. The water phase was extracted with 3x20 ml of DCM. Combined organic phases were dried on Na_2SO_4, filtered, and concentrated. Crude **S-8** (7.4 mmol, 92%) was used for the synthesis of **methyl 2-acetylhex-4-ynoate-3,3-*d₃*** within **Conditions D**

Analytical data of isolated products

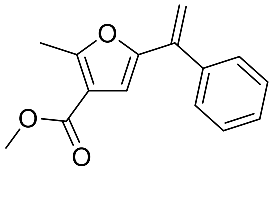

(2) methyl 2-methyl-5-(1-phenylvinyl)furan-3-carboxylate Prepared in one-pot reaction of methyl 2-acetylhex-4-ynoate (67.3 mg, 0.40 mmol) with bromobenzene (69.9 mg, 0.44 mmol) under **conditions A** (72.5 mg, 0.30 mmol, 75%), with a second stage lasting for 0.5h. The title compound was isolated as yellowish oil after chromatography on silica gel (15 g column, Hexane:Ethyl Acetate 98:2); ^{1}H NMR (400 MHz, $CDCl_3$) δ 7.46 – 7.40 (m,

2H), 7.39 – 7.34 (m, 3H), 6.42 (s, 1H), 5.76 – 5.71 (m, 1H), 5.24 (d, *J* = 1.0 Hz, 1H), 3.80 (s, 3H), 2.64 (s, 3H); $^{13}C\{^1H\}$ NMR (101 MHz, $CDCl_3$) δ 164.3, 159.3, 151.8, 139.0, 138.6, 128.3, 128.2, 128.1, 114.8, 112.2, 109.6, 51.2, 13.8; IR (CH_2Cl_2): 1718 (C=O), 1231 (C-O), 1093 (C-O) cm^{-1} HRMS (EI) m/z: $[M]^+$ Calcd for $C_{15}H_{14}O_3$: 242.0943, Found 242.0951

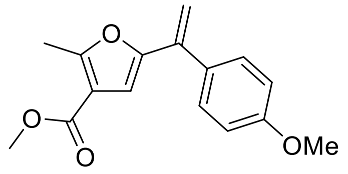

(3) methyl 5-(1-(4-methoxyphenyl)vinyl)-2-methylfuran-3-carboxylate Prepared in one-pot reaction of methyl 2-acetylhex-4-ynoate (67.3 mg, 0.40 mmol) with 1-bromo-4-methoxybenzene (83.0 mg, 0.44 mmol) under modified **conditions A** (68.7 mg, 0.25 mmol, 63%), under 40°C on first stage, with second stage lasting for 4h. The title compound was isolated as off white oill after chromatography on silica gel (15 g column, Hexane:Ethyl Acetate 98:2); ^1H NMR (400 MHz, $CDCl_3$) δ 7.39 – 7.33 (m, 2H), 6.94 – 6.87 (m, 2H), 6.42 (s, 1H), 5.66 (brs, 1H), 5.19 (brs, 1H), 3.84 (s, 3H), 3.79 (s, 3H), 2.63 (s, 3H); $^{13}C\{^1H\}$ NMR (101 MHz, $CDCl_3$) δ 164.3, 159.6, 159.3, 152.1, 138.0, 131.4, 129.3, 114.8, 113.7, 111.4, 109.4, 55.3, 51.2, 13.8; IR (CH_2Cl_2): 1718 (C=O), 1233 (C-O), 1092 (C-O) cm^{-1} HRMS (EI) m/z: $[M]^+$ Calcd for $C_{16}H_{16}O_4$: 272.1049, Found 272.1048

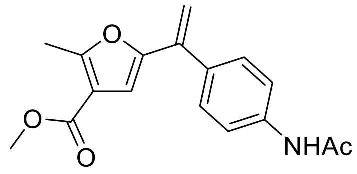

(4) methyl 5-(1-(4-acetamidophenyl)vinyl)-2-methylfuran-3-carboxylate Prepared in one-pot reaction of methyl 2-acetylhex-4-ynoate (67.3 mg, 0.40 mmol) with *N*-(4-bromophenyl)acetamide (94.2 mg, 0.44 mmol) under modified **conditions A** (74.3 mg, 0.25 mmol, 62%), under 40°C on first stage and 2mol% Xphos Pd G3, with second stage lasting for 0.5h. The title compound was isolated as off white solid after chromatography on silica gel (15 g column, Hexane:Ethyl Acetate 98:2); ^1H NMR (500 MHz, $CDCl_3$) δ 7.74 (brs, 1H), 7.52 (d, *J* = 8.3 Hz, 2H), 7.36 (d, *J* = 8.3 Hz, 2H), 6.39 (s, 1H), 5.68 (s, 1H), 5.19 (s, 1H), 3.78 (s, 3H), 2.61 (s, 3H), 2.18 (s, 3H); $^{13}C\{^1H\}$ NMR (101 MHz, $CDCl_3$) δ 168.5, 164.4, 159.4, 151.7, 138.0, 137.9, 134.8, 128.8, 119.7, 114.7, 111.9, 109.5, 51.3, 24.5, 13.8; IR (CH_2Cl_2): 3305 (N-H), 1717 (C=O), 1233 (C-O), 1093 (C-O) cm^{-1} HRMS (EI) m/z: $[M]^+$ Calcd for $C_{17}H_{17}NO_4$: 299.1158, Found 299.1165

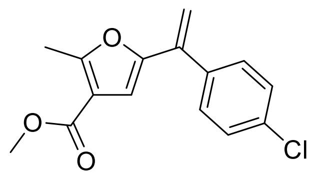

(5) methyl 5-(1-(4-chlorophenyl)vinyl)-2-methylfuran-3-carboxylate Prepared in one-pot reaction of methyl 2-acetylhex-4-ynoate (67.3 mg, 0.40 mmol) with 1-bromo-4-chlorobenzene (85.0 mg, 0.44 mmol) under **conditions A** (75.0 mg, 0.27 mmol, 68%), with second stage lasting for 0.5h. The title compound was isolated as colorless oil after chromatography on silica gel (15 g column, Hexane:Ethyl Acetate 98:2); ^1H NMR (400 MHz, $CDCl_3$) δ 7.35 (s, 4H), 6.38 (s, 1H), 5.73 (s, 1H), 5.22 (s, 1H), 3.79 (s, 3H), 2.63 (s, 3H); $^{13}C\{^1H\}$ NMR (101 MHz, $CDCl_3$) δ 164.2, 159.5, 151.4, 137.5, 137.4, 134.1, 129.5, 128.5, 114.9, 112.6, 109.6, 51.3, 13.8.; IR (CH_2Cl_2): 1719 (C=O), 1231 (C-O), 1092 (C-O) cm^{-1} HRMS (EI) m/z: $[M]^+$ Calcd for $C_{15}H_{13}O_3Cl$: 276.0553, Found 276.0554

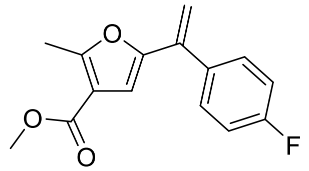

(6) methyl 5-(1-(4-fluorophenyl)vinyl)-2-methylfuran-3-carboxylate Prepared in one-pot reaction of methyl 2-acetylhex-4-ynoate (67.3 mg, 0.40 mmol) with 1-bromo-4-fluorobenzene (77.0 mg, 0.44 mmol) under **conditions A** (73.6 mg, 0.28 mmol, 71%), with second stage lasting for 0.5h. The title compound was isolated as colorless oil after chromatography on silica gel (15 g column, Hexane:Ethyl Acetate 98:2); ^1H NMR (400 MHz, $CDCl_3$) δ 7.43 – 7.35 (m, 2H), 7.10 – 7.01 (m, 2H), 6.37 (s, 1H), 5.72 (s, 1H), 5.19 (s, 1H), 3.82 – 3.76 (m, 3H), 2.65 – 2.60 (m, 3H); $^{13}C\{^1H\}$ NMR (101 MHz, $CDCl_3$) δ 164.2, 162.7 (d, *J* = 248.2 Hz), 159.5, 151.7, 137.6, 135.0 (d, *J* = 3.4 Hz), 129.9 (d, *J* = 8.3 Hz), 115.2 (d, *J* = 21.6 Hz), 114.8, 112.3, 109.6, 51.3, 13.8; IR (CH_2Cl_2): 1719 (C=O), 1232 (C-O), 1092 (C-O) cm^{-1} HRMS (EI) m/z: $[M]^+$ Calcd for $C_{15}H_{13}O_3F$: 260.0849, Found 260.0843

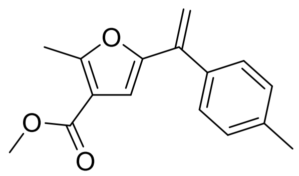

(7) methyl 2-methyl-5-(1-(p-tolyl)vinyl)furan-3-carboxylate Prepared in one-pot reaction of methyl 2-acetylhex-4-ynoate (67.3 mg, 0.40 mmol) with 1-bromo-4-methylbenzene (75.3 mg, 0.44 mmol) under **conditions A** (60.8 mg, 0.24 mmol, 59%), with second stage lasting for 0.5h. The title compound was isolated as transparent oil after chromatography on silica gel (15 g column, Hexane:Ethyl Acetate 98:2); ^{1}H NMR (500 MHz, $CDCl_3$) δ 7.33 (d, *J* = 8.0 Hz, 2H), 7.19 (d, *J* = 8.3 Hz, 2H), 6.43 (s, 1H), 5.71 (s, 1H), 5.22 (s, 1H), 3.80 (s, 3H), 2.64 (s, 3H), 2.39 (s, 3H); $^{13}C\{^{1}H\}$ NMR (101 MHz, $CDCl_3$) δ 164.3, 159.3, 152.0, 138.4, 138.0, 136.1, 129.0, 128.1, 114.7, 111.8, 109.5, 51.2, 21.2, 13.8; IR (CH_2Cl_2): 1719 (C=O), 1232 (C-O), 1092 (C-O) cm^{-1} HRMS (EI) m/z: $[M]^+$ Calcd for $C_{16}H_{16}O_3$: 256.1099, Found 256.1097

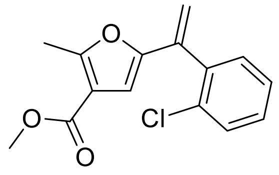

(8) methyl 5-(1-(2-chlorophenyl)vinyl)-2-methylfuran-3-carboxylate Prepared in one-pot reaction of methyl 2-acetylhex-4-ynoate (67.3 mg, 0.40 mmol) with 1-bromo-2-chlorobenzene (84.2 mg, 0.44 mmol) under modified **conditions A** (47.5 mg, 0.17 mmol, 43%), with 2 mol% XPhos Pd G3 and under 40°C on first stage, with second stage lasting for 0.5h. The title compound was isolated as colorless oil after chromatography on silica gel (15 g column, Hexane:Ethyl Acetate 98:2); ^{1}H NMR (500 MHz, $CDCl_3$) δ 7.45 – 7.40 (m, 1H), 7.33 – 7.27 (m, 3H), 6.09 (s, 1H), 5.92 (s, 1H), 5.15 (s, 1H), 3.76 (s, 3H), 2.63 (s, 3H); $^{13}C\{^{1}H\}$ NMR (101 MHz, $CDCl_3$) δ 164.2, 159.4, 151.0, 137.6, 135.9, 133.2, 131.2, 129.7, 129.3, 126.7, 114.9, 113.6, 109.3, 51.2, 13.9; IR (CH_2Cl_2): 1720 (C=O), 1232 (C-O), 1100 (C-O) cm^{-1} HRMS (EI) m/z: $[M]^+$ Calcd for $C_{15}H_{13}O_3Cl$: 276.0553, Found 276.0551

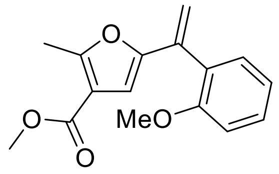

(9) methyl 5-(1-(2-methoxyphenyl)vinyl)-2-methylfuran-3-carboxylate Prepared in one-pot reaction of methyl 2-acetylhex-4-ynoate (67.3 mg, 0.40 mmol) with 1-bromo-2- methoxybenzene (82.3 mg, 0.44 mmol) under modified **conditions A** (65.5 mg, 0.24 mmol, 60%), with 2 mol% XPhos Pd G3 and under 40°C on first stage, with second stage lasting for 0.5h. The title compound was isolated as white solid after chromatography on silica gel (15 g column, Hexane:Ethyl Acetate 98:2); ^{1}H NMR (400 MHz, $CDCl_3$) δ 7.34 (td, *J* = 8.1, 1.8 Hz, 1H), 7.22 (dd, *J* = 7.4, 1.8 Hz, 1H), 7.01 – 6.91 (m, 2H), 6.13 (s, 1H), 5.86 (d, *J* = 1.5 Hz, 1H), 5.15 (d, *J* = 1.5 Hz, 1H), 3.76 (s, 6H), 2.61 (s, 3H); $^{13}C\{^{1}H\}$ NMR (101 MHz, $CDCl_3$) δ 164.5, 158.9, 157.0, 152.0, 135.4, 130.9, 129.4, 128.1, 120.5, 114.8, 113.0, 111.1, 108.6, 55.6, 51.2, 13.9; IR (CH_2Cl_2): 1718 (C=O), 1235 (C-O), 1088 (C-O) cm^{-1} HRMS (EI) m/z: $[M]^+$ Calcd for $C_{16}H_{16}O_4$: 272.1049, Found 272.1045

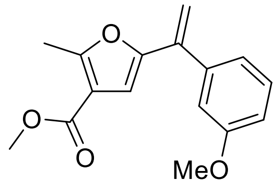

(10) methyl 5-(1-(3-methoxyphenyl)vinyl)-2-methylfuran-3-carboxylate Prepared in one-pot reaction of methyl 2-acetylhex-4-ynoate (67.3 mg, 0.40 mmol) with 1-bromo-3-methoxybenzene (84.2 mg, 0.44 mmol) under modified **conditions A** (54.2 mg, 0.21 mmol, 53%), with 1 mol% XPhos Pd G3 and under 40°C on first stage, with second stage lasting for 0.5h. The title compound was isolated as white solid after chromatography on silica gel (15 g column, Hexane:Ethyl Acetate 98:2); ^{1}H NMR (500 MHz, $CDCl_3$) δ 7.29 (t, *J* = 7.9 Hz, 1H), 7.03 – 7.00 (m, 1H), 6.98 – 6.96 (m, 1H), 6.91 (dd, *J* = 8.2, 1.8 Hz, 1H), 6.43 (s, 1H), 5.73 (s, 1H), 5.25 (s, 1H), 3.83 (s, 3H), 3.79 (s, 3H), 2.63 (s, 3H); $^{13}C\{^{1}H\}$ NMR (101 MHz, $CDCl_3$) δ 164.3, 159.5, 159.4, 151.7, 140.4, 138.5, 129.3, 120.7, 114.8, 114.0, 113.6, 112.2, 109.6, 55.3, 13.9; IR (CH_2Cl_2): 1719 (C=O), 1233 (C-O), 1097 (C-O) cm^{-1} HRMS (EI) m/z: $[M]^+$ Calcd for $C_{16}H_{16}O_4$: 272.1049, Found 272.1055

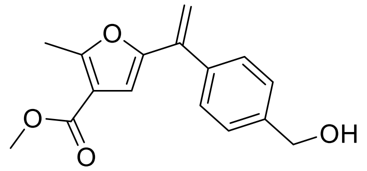

(11) methyl 5-(1-(4-(hydroxymethyl)phenyl)vinyl)-2-methylfuran-3-carboxylate Prepared in one-pot reaction of methyl 2-acetylhex-4-ynoate (67.3 mg, 0.40 mmol) with (4-bromophenyl)methanol (82.3 mg, 0.44 mmol) under modified **conditions A** (69.6 mg, 0.26 mmol, 64%), under 40°C on first stage, with second stage lasting for 0.5h. The title compound was isolated as pale-green oil after chromatography on silica gel (15 g column, Hexane:Ethyl Acetate 8:2); ^{1}H NMR (400 MHz, $CDCl_3$) δ 7.44 – 7.34 (m, 4H), 6.40 (s, 1H), 5.73 (s, 1H), 5.23 (s, 1H), 4.71 (s, 2H), 3.78 (s, 3H), 2.62 (s, 3H); $^{13}C\{^{1}H\}$ NMR (101 MHz,

$CDCl_3$) δ 164.3, 159.4, 151.8, 140.9, 138.3, 138.3, 128.4, 126.9, 114.8, 112.2, 109.5, 64.9, 51.3, 13.8; IR (CH_2Cl_2): 3418 (O-H), 1717 (C=O), 1233 (C-O), 1092 (C-O) cm^{-1} HRMS (EI) m/z: $[M]^+$ Calcd for $C_{16}H_{16}O_4$: 272.1049, Found 272.1053

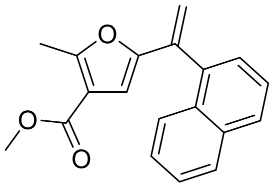

(12) methyl 2-methyl-5-(1-(naphthalen-1-yl)vinyl)furan-3-carboxylate Prepared in one-pot reaction of methyl 2-acetylhex-4-ynoate (67.3 mg, 0.40 mmol) with 1-bromonaphthalene (91.1 mg, 0.44 mmol) under modified **conditions A** (19.4 mg, 0.07 mmol, 17%), under 40°C on first stage, with second stage lasting for 4h. The title compound was isolated as pale-green oil after chromatography on silica gel (15 g column, Hexane:Dioxane 98:2); ^1H NMR (400 MHz, $CDCl_3$) δ 7.94 – 7.83 (m, 3H), 7.52 – 7.39 (m, 5H), 6.07 (d, J = 1.1 Hz, 1H), 5.97 (s, 1H), 5.26 (d, J = 1.2 Hz, 1H), 3.70 (s, 3H), 2.65 (s, 3H); $^{13}C\{^1H\}$ NMR (101 MHz, $CDCl_3$) δ 164.2, 159.3, 152.4, 136.7, 136.6, 133.6, 131.8, 128.3, 128.2, 126.9, 126.0, 125.8, 125.8, 125.3, 114.9, 113.8, 109.7, 51.2, 13.9; IR (CH_2Cl_2): 1719 (C=O), 1234 (C-O), 1079 (C-O) cm^{-1} HRMS (EI) m/z: $[M]^+$ Calcd for $C_{19}H_{16}O_3$: 292.1099, Found 292.1095

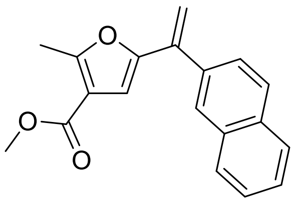

(13) methyl 2-methyl-5-(1-(naphthalen-2-yl)vinyl)furan-3-carboxylate Prepared in one-pot reaction of methyl 2-acetylhex-4-ynoate (67.3 mg, 0.40 mmol) with 2-bromonaphthalene (91.1 mg, 0.44 mmol) under modified **conditions A** (70.6 mg, 0.24 mmol, 60%), with 1mol% Xphos Pd G3 and 40°C on first stage, with second stage lasting for 0.5h. The title compound was isolated as yellowish oil after chromatography on silica gel (15 g column, Hexane:Ethyl Acetate 98:2; ^1H NMR (400 MHz, $CDCl_3$) δ 7.92 (br s, 1H), 7.90 – 7.83 (m, 3H), 7.58 – 7.48 (m, 3H), 6.47 (s, 1H), 5.84 (s, 1H), 5.37 (s, 1H), 3.79 (s, 3H), 2.67 (s, 3H); $^{13}C\{^1H\}$ NMR (101 MHz, $CDCl_3$) δ 164.3, 159.4, 151.9, 138.6, 136.4, 133.2, 133.1, 128.1, 127.9, 127.6, 127.2, 126.4, 126.3, 126.2, 114.9, 112.7, 109.7, 51.2, 13.9; IR (CH_2Cl_2): 1718 (C=O), 1231 (C-O), 1090 (C-O) cm^{-1} HRMS (EI) m/z: $[M]^+$ Calcd for $C_{19}H_{16}O_3$: 292.1099, Found 292.1103

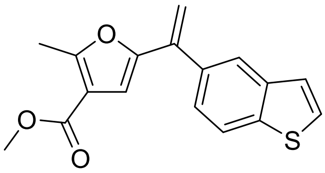

(14) methyl 5-(1-(benzo[b]thiophen-5-yl)vinyl)-2-methylfuran-3-carboxylate Prepared in one-pot reaction of methyl 2-acetylhex-4-ynoate (67.3 mg, 0.40 mmol) with 5-bromobenzo[b]thiophene (93.8 mg, 0.44 mmol) under modified **conditions A** (71.8 mg, 0.24 mmol, 60%), under 40°C on first stage, with second stage lasting for 4h. The title compound was isolated as pale-green oil after chromatography on silica gel (15 g column, Hexane:Ethyl Acetate 95:5); ^1H NMR (400 MHz, $CDCl_3$) δ 7.91 – 7.80 (m, 2H), 7.50 – 7.38 (m, 2H), 7.38 – 7.30 (m, 1H), 6.44 (s, 1H), 5.80 (s, 1H), 5.30 (s, 1H), 3.79 (s, 3H), 2.66 (s, 3H); $^{13}C\{^1H\}$ NMR (101 MHz, $CDCl_3$) δ 164.3, 159.4, 152.0, 139.6, 139.5, 138.6, 135.3, 127.0, 124.7, 123.9, 123.1, 122.2, 114.8, 112.4, 109.7, 51.2, 13.9; IR (CH_2Cl_2): 1717 (C=O), 1232 (C-O), 1091 (C-O) cm^{-1} HRMS (EI) m/z: $[M]^+$ Calcd for $C_{17}H_{14}O_3S$: 298.0664, Found 298.0665

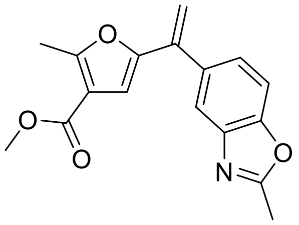

(15) methyl 2-methyl-5-(1-(2-methylbenzo[d]oxazol-5-yl)vinyl)furan-3-carboxylate Prepared in one-pot reaction of methyl 2-acetylhex-4-ynoate (67.3 mg, 0.40 mmol) with 5-bromo-2-methylbenzo[d]oxazole (93.3 mg, 0.44 mmol) under modified **conditions A** (70.7 mg, 0.24 mmol, 59%), under 40°C on first stage, with second stage lasting for 4h. The title compound was isolated as orange oil after chromatography on silica gel (15 g column, Hexane:Ethyl Acetate 8:2); ^1H NMR (400 MHz, $CDCl_3$) δ 7.69 (d, J = 1.4 Hz, 1H), 7.43 (d, J = 8.4 Hz, 1H), 7.34 (dd, J = 8.4, 1.7 Hz, 1H), 6.37 (s, 1H), 5.74 (brs, 1H), 5.23 (brs, 1H), 3.76 (s, 3H), 2.63 (s, 3H), 2.62 (s, 3H); $^{13}C\{^1H\}$ NMR (101 MHz, $CDCl_3$) δ 164.5, 164.2, 159.4, 151.8, 150.9, 141.6, 138.2, 135.4, 124.9, 119.2, 114.8, 112.5, 109.8, 109.7, 51.2, 14.5, 13.8; IR (CH_2Cl_2): 1719 (C=O), 1231 (C-O), 1083 (C-O) cm^{-1} HRMS (EI) m/z: $[M]^+$ Calcd for $C_{17}H_{15}NO_4$: 297.1001, Found 297.1001

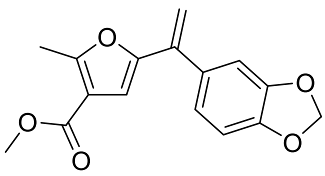

(16) methyl 5-(1-(benzo[d][1,3]dioxol-5-yl)vinyl)-2-methylfuran-3-carboxylate Prepared in one-pot reaction of methyl 2-acetylhex-4-ynoate (67.3 mg, 0.40 mmol) with 5-bromobenzo[d][1,3]dioxole (88.5 mg, 0.44 mmol) under modified **conditions A** (65.9 mg, 0.23 mmol, 58%), under 40°C on first stage, with second stage lasting for 0.5h. The title compound was isolated as white solid after chromatography on silica gel (15 g column, Hexane:Ethyl Acetate 95:5); ^{1}H NMR (400 MHz, $CDCl_3$) δ 6.94 – 6.88 (m, 2H), 6.82 – 6.78 (m, 1H), 6.43 (s, 1H), 5.98 (s, 2H), 5.65 (brs, 1H), 5.18 (brs, 1H), 3.80 (s, 3H), 2.62 (s, 3H); ^{13}C{^{1}H} NMR (101 MHz, $CDCl_3$) δ 164.3, 159.3, 151.9, 147.6, 147.5, 138.1, 133.0, 121.8, 114.8, 111.7, 109.5, 108.7, 108.1, 101.1, 51.2, 13.8; IR (CH_2Cl_2): 1717 (C=O), 1235 (C-O), 1080 (C-O) cm^{-1} HRMS (EI) m/z: [M]$^+$ Calcd for $C_{16}H_{14}O_5$: 286.0841, Found 286.0832

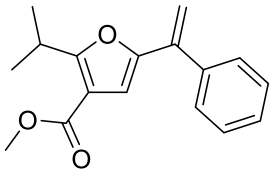

(17) methyl 2-isopropyl-5-(1-phenylvinyl)furan-3-carboxylate Prepared in one-pot reaction of methyl 2-isobutyrylhex-4-ynoate (78.5 mg, 0.40 mmol) with bromobenzene (69.1 mg, 0.44 mmol) under modified **conditions A** (81.4 mg, 0.30 mmol, 75%), under 40°C on first stage, with second stage lasting for 4h. The title compound was isolated as transparent oil after chromatography on silica gel (15 g column, Hexane:Ethyl Acetate 98:2; ^{1}H NMR (600 MHz, $CDCl_3$) δ 7.45 – 7.42 (m, 2H), 7.40 – 7.35 (m, 3H), 6.40 (s, 1H), 5.74 (s, 1H), 5.25 (s, 1H), 3.82 – 3.78 (m, 4H), 1.34 (d, *J* = 7.0 Hz, 6H); ^{13}C{^{1}H} NMR (101 MHz, $CDCl_3$) δ 167.4, 164.2, 151.5, 139.0, 138.7, 128.3, 128.2, 128.1, 112.8, 112.1, 109.5, 51.2, 27.4, 20.8; IR (CH_2Cl_2): 1719 (C=O), 1231 (C-O), 1065 (C-O) cm^{-1} HRMS (EI) m/z: [M]$^+$ Calcd for $C_{17}H_{18}O_3$: 270.1256, Found 270.1255

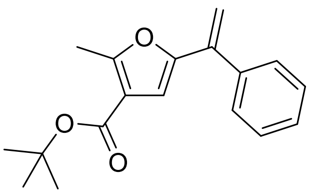

(18) tert-butyl 2-methyl-5-(1-phenylvinyl)furan-3-carboxylate Prepared in one-pot reaction of tert-butyl 2-acetylhex-4-ynoate (84.1 mg, 0.40 mmol) with bromobenzene (69.1 mg, 0.44 mmol) under modified **conditions A** (71.0 mg, 0.25 mmol, 62%), under 40°C on first stage, with second stage lasting for 4h. The title compound was isolated as beige solid after chromatography on silica gel (15 g column, Hexane:Ethyl Acetate 98:2); ^{1}H NMR (400 MHz, $CDCl_3$) δ 7.47 – 7.41 (m, 2H), 7.41 – 7.35 (m, 3H), 6.40 (s, 1H), 5.74 (s, 1H), 5.24 (s, 1H), 2.62 (s, 3H), 1.55 (s, 9H); ^{13}C{^{1}H} NMR (101 MHz, $CDCl_3$) δ 163.3, 158.5, 151.4, 139.1, 138.7, 128.2, 128.2, 116.5, 112.0, 109.9, 80.5, 28.3, 13.9; IR (CH_2Cl_2): 1710 (C=O), 1234 (C-O), 1092 (C-O) cm^{-1} HRMS (EI) m/z: [M]$^+$ Calcd for $C_{18}H_{20}O_3$: 284.1412, Found 284.1419

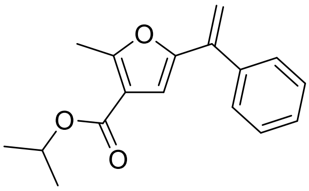

(19) isopropyl 2-methyl-5-(1-phenylvinyl)furan-3-carboxylate Prepared in one-pot reaction of isopropyl 2-acetylhex-4-ynoate (78.5 mg, 0.40 mmol) with bromobenzene (69.1 mg, 0.44 mmol) under modified **conditions A** (62.8 mg, 0.23 mmol, 58%), under 40°C on first stage, with second stage lasting for 4h. The title compound was isolated as transparent oil after chromatography on silica gel (15 g column, Hexane:Ethyl Acetate 98:2); ^{1}H NMR (400 MHz, $CDCl_3$) δ 7.47 – 7.41 (m, 2H), 7.41 – 7.35 (m, 3H), 6.41 (s, 1H), 5.73 (brs, 1H), 5.23 (brs, 1H), 5.16 (hept, *J* = 6.3 Hz, 1H), 2.64 (s, 3H), 1.31 (d, *J* = 6.3 Hz, 6H); ^{13}C{^{1}H} NMR (101 MHz, $CDCl_3$) δ 163.5, 159.0, 151.7, 139.1, 138.7, 128.3, 128.2, 128.1, 115.5, 112.2, 109.6, 67.5, 22.0, 13.9; IR (CH_2Cl_2): 1712 (C=O), 1231 (C-O), 1092 (C-O) cm^{-1} HRMS (ESI) m/z: [M+Na]$^+$ Calcd for $C_{17}H_{18}O_3Na$: 293.1154, Found 293.1153

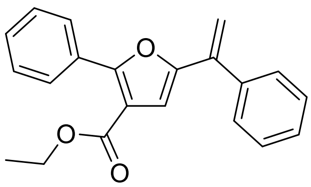

(20) ethyl 2-phenyl-5-(1-phenylvinyl)furan-3-carboxylate Prepared in one-pot reaction of ethyl 3-oxo-3-phenylpropanoate (97.7 mg, 0.40 mmol) with bromobenzene (69.1 mg, 0.44 mmol) under modified **conditions A** (67.0 mg, 0.21 mmol, 53%), under 40°C on first stage, with second stage lasting for 0.5h with 2 mL of DCE being added. The title compound was isolated as white solid after chromatography on silica gel (15 g column, Hexane:Ethyl Acetate 99:1); ^{1}H NMR (500 MHz, $CDCl_3$) δ 8.07 (d, J = 6.9 Hz, 2H), 7.53 – 7.38 (m, 8H), 6.64 (s, 1H), 5.89 (s, 1H), 5.35 (s, 1H), 4.29 (q, J = 7.1 Hz, 2H), 1.32 (t, J = 7.1 Hz, 3H); ^{13}C{^{1}H} NMR (101 MHz, $CDCl_3$) δ 152.3, 138.9, 138.5, 129.4, 128.5, 128.4, 128.3, 128.2, 128.1, 113.3, 111.9,

60.5, 14.2; IR (CH_2Cl_2): 1719 (C=O), 1231 (C-O), 1083 (C-O) cm^{-1} HRMS (EI) m/z: [M]$^+$ Calcd for $C_{21}H_{18}O_3$: 318.1264, Found 318.1256

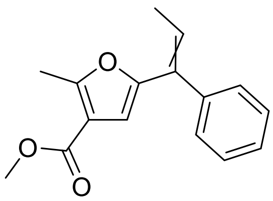

(21) methyl 2-methyl-5-(1-phenylprop-1-en-1-yl)furan-3-carboxylate Prepared in one-pot reaction of methyl 2-acetylhept-4-ynoate (72.9 mg, 0.40 mmol) with bromobenzene (69.1 mg, 0.44 mmol) under modified **conditions A** (69.2 mg, 67%), under 40°C on first stage, with second stage lasting for 0.5h. The title compound was isolated in mixture of isomers (E/Z 4/1), as yellowish oil after chromatography on silica gel (15 g column, Hexane:Ethyl Acetate 98:1); **Isomer E** ^{1}H NMR (400 MHz, $CDCl_3$) δ 7.43 – 7.21 (m, 5H), 6.36 (q, *J* = 7.2 Hz, 1H), 5.96 (s, 1H), 3.75 (s, 3H), 2.60 (s, 3H), 1.69 (d, *J* = 7.2 Hz, 3H); ^{13}C{^{1}H} NMR (101 MHz, $CDCl_3$) δ 164.5, 158.5, 153.4, 136.8, 132.0, 129.7, 128.3, 127.5, 121.9, 114.6, 107.3, 51.1, 14.5, 13.8; **Isomer Z** ^{1}H NMR (400 MHz, $CDCl_3$) δ 7.43 – 7.21 (m, 5H), 6.45 (s, 1H), 5.92 (q, *J* = 7.3 Hz, 1H), 3.81 (s, 3H), 2.59 (s, 3H), 2.06 (d, *J* = 7.3 Hz, 3H); ^{13}C{^{1}H} NMR (101 MHz, $CDCl_3$) δ 164.5, 158.5, 153.4, 136.8, 132.0, 129.7, 128.3, 127.5, 121.9, 114.6, 107.3, 51.1, 14.5, 13.8; IR (CH_2Cl_2): 1719 (C=O), 1233 (C-O), 1089 (C-O) cm^{-1} HRMS (EI) m/z: [M]$^+$ Calcd for $C_{16}H_{16}O_3$: 256.1099, Found 256.1105

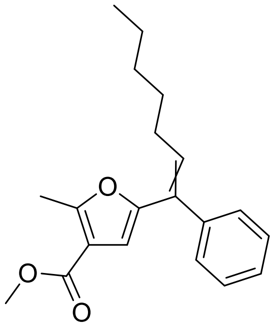

(22) methyl 2-methyl-5-(1-phenylhept-1-en-1-yl)furan-3-carboxylate Prepared in reaction of methyl 2-acetylundec-4-ynoate (95.4 mg, 0.40 mmol) with bromobenzene (69.1 mg, 0.44 mmol) under modified **conditions A** (65.0 mg, 0.21 mmol, 52%) under 40°C on first stage and 2 mol% Xphos Pd G3, with second stage lasting for 0.5h. The title compound was isolated in mixture of E/Z isomers (E/Z 4/1) as transparent oil after chromatography on silica gel (15 g column, Hexane:Ethyl Acetate 98:2); **Isomer E:** ^{1}H NMR (500 MHz, $CDCl_3$) δ 7.40 – 7.28 (m, 3H), 7.25 – 7.20 (m, 2H), 6.28 (t, *J* = 7.7 Hz, 1H), 5.94 (s, 1H), 3.75 (s, 3H), 2.61 (s, 3H), 2.03 (q, *J* = 7.6 Hz, 2H), 1.45 – 1.38 (m, 2H), 1.28 – 1.20 (m, 4H), 0.86 (t, *J* = 7.0 Hz, 3H); ^{13}C{^{1}H} NMR (101 MHz, $CDCl_3$) δ 164.5, 158.5, 153.4, 137.0, 131.0, 129.6, 128.2, 127.7, 127.4, 114.6, 107.5, 51.1, 31.4, 29.4, 28.7, 22.5, 14.0, 13.8; **Isomer Z:** ^{1}H NMR (500 MHz, $CDCl_3$) δ 7.40 – 7.28 (m, 3H), 7.25 – 7.20 (m, 2H), 6.44 (s, 1H), 5.84 (t, *J* = 7.4 Hz, 1H), 3.82 (s, 3H), 2.59 (s, 3H), 2.46 (q, *J* = 7.5 Hz, 2H), 1.53 (p, *J* = 7.3 Hz, 2H), 1.37 – 1.33 (m, 4H), 0.93 – 0.89 (m, 3H); ^{13}C{^{1}H} NMR (126 MHz, $CDCl_3$) δ 164.5, 158.6, 151.2, 141.1, 134.0, 130.8, 128.1, 128.0, 127.3, 114.1, 110.9, 51.3, 31.6, 29.8, 29.5, 22.5, 14.0, 13.9; IR (CH_2Cl_2): 1720 (C=O), 1232 (C-O), 1089 (C-O) cm^{-1} HRMS (EI) m/z: [M]$^+$ Calcd for $C_{20}H_{24}O_3$: 312.1725, Found 312.1736

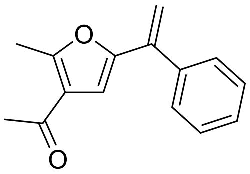

(23) 1-(2-methyl-5-(1-phenylvinyl)furan-3-yl)ethan-1-one Prepared in one-pot reaction of 3-(but-2-yn-1-yl)pentane-2,4-dione (60.9 mg, 0.40 mmol) with bromobenzene (69.1 mg, 0.44 mmol) under modified **conditions A** (59.0 mg, 0.26 mmol, 65%), with 1 mol% XPhos Pd G3 and under 40°C on first stage, with second stage lasting for 0.5h. The title compound was isolated as colorless oil after chromatography on silica gel (15 g column, Hexane:Ethyl Acetate 95:5); ^{1}H NMR (400 MHz, $CDCl_3$) δ 7.45 – 7.41 (m, 2H), 7.41 – 7.36 (m, 3H), 6.37 (s, 1H), 5.76 (s, 1H), 5.25 (s, 1H), 2.65 (s, 3H), 2.35 (s, 3H); ^{13}C{^{1}H} NMR (101 MHz, $CDCl_3$) δ 194.0, 158.5, 151.7, 139.0, 138.5, 128.3, 128.2, 122.9, 112.5, 109.2, 29.0, 14.5; IR (CH_2Cl_2): 1677 (C=O), cm^{-1} HRMS (EI) m/z: [M]$^+$ Calcd for $C_{15}H_{14}O_2$: 226.0994, Found 226.0991

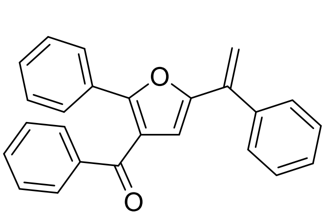

(24) phenyl(2-phenyl-5-(1-phenylvinyl)furan-3-yl)methanone Prepared in one-pot reaction of 2-(but-2-yn-1-yl)-1,3-diphenylpropane-1,3-dione (110.5 mg, 0.40 mmol) with bromobenzene (69.1 mg, 0.44 mmol) under modified **conditions A** (78.4 mg, 0.22 mmol, 56%), with 1 mol% XPhos Pd G3 and under 40°C on first stage, with second stage lasting for 0.5h. The title compound was isolated as yellow oil after chromatography on silica gel (15 g column, Hexane:Ethyl Acetate 95:5); ^{1}H NMR (400 MHz, $CDCl_3$) δ 7.90 – 7.83 (m, 2H), 7.80 – 7.73 (m, 2H), 7.55 – 7.47 (m, 3H), 7.44 – 7.30 (m, 8H), 6.51 (s, 1H), 5.97 (s, 1H), 5.40 (s, 1H); ^{13}C{^{1}H} NMR (101 MHz, $CDCl_3$) δ 191.6, 152.4, 138.8, 138.5,

137.8, 132.8, 129.7, 129.6, 129.1, 128.4, 128.3, 128.3, 128.2, 127.5, 122.5, 113.5, 112.5; IR (CH_2Cl_2): 1667 (C=O) cm^{-1} HRMS (EI) m/z: $[M]^+$ Calcd for $C_{25}H_{18}O_2$: 350.1307, Found 350.1309

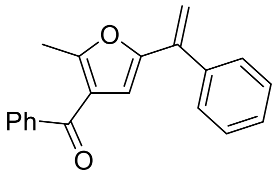

(25) 1-(2-methyl-5-(1-phenylvinyl)furan-3-yl)ethan-1-one Prepared in one-pot reaction of 2-(but-2-yn-1-yl)-1-phenylbutane-1,3-dione (85.7 mg, 0.40 mmol) with bromobenzene (69.1 mg, 0.44 mmol) under modified **conditions A** with 1mol% Xphos Pd G3 (60.4 mg, 0.21 mmol, 52%), with second stage lasting for 0.5h. The title compound was isolated as yellow-green oil after chromatography on silica gel (15 g column, Hexane:Ethyl Acetate 95:5); ^{1}H NMR (500 MHz, $CDCl_3$) δ 7.81 – 7.76 (m, 2H), 7.53 (t, J = 7.4 Hz, 1H), 7.48 – 7.42 (m, 4H), 7.40 – 7.35 (m, 3H), 6.38 (s, 1H), 5.81 (s, 1H), 5.29 (s, 1H), 2.60 (s, 3H); $^{13}C\{^1H\}$ NMR (126 MHz, $CDCl_3$) δ 191.1, 159.5, 151.6, 138.9, 138.5, 132.2, 128.9, 128.3, 128.2, 128.2, 122.1, 112.7, 110.5, 14.4; IR (CH_2Cl_2): 1651 (C=O) cm^{-1} HRMS (EI) m/z: $[M]^+$ Calcd for $C_{20}H_{16}O_2$: 288.1150, Found 288.1144

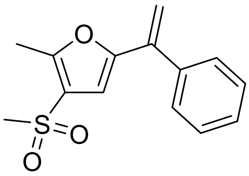

(26) 2-methyl-3-(methylsulfonyl)-5-(1-phenylvinyl)furan Prepared in one-pot reaction of 3-(methylsulfonyl)hept-5-yn-2-one (75.3 mg, 0.40 mmol) with bromobenzene (69.1 mg, 0.44 mmol) under **conditions A** (69.5 mg, 0.26 mmol, 66%), with second stage lasting for 4h. The title compound was isolated as white solid after chromatography on silica gel (15 g column, Hexane:Ethyl Acetate 9:1); ^{1}H NMR (400 MHz, $CDCl_3$) δ 7.42 – 7.36 (m, 5H), 6.37 (s, 1H), 5.79 (s, 1H), 5.32 (s, 1H), 3.03 (s, 3H), 2.64 (s, 3H); $^{13}C\{^1H\}$ NMR (101 MHz, $CDCl_3$) δ 156.5, 152.8, 138.3, 138.0, 128.4, 128.4, 128.1, 123.4, 113.7, 107.9, 45.0, 12.9; IR (CH_2Cl_2): 1309 (S=O), 1147 (S=O) cm^{-1} HRMS (ESI) m/z: $[M+Na]^+$ Calcd for $C_{14}H_{14}O_3NaS$: 285.0561, Found 285.0563

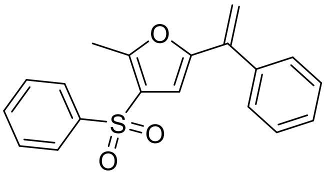

(27) 2-methyl-3-(phenylsulfonyl)-5-(1-phenylvinyl)furan Prepared in one-pot reaction of 3-(phenylsulfonyl)hept-5-yn-2-one (100.2 mg, 0.40 mmol) with bromobenzene (69.1 mg, 0.44 mmol) under **conditions A** (57.9 mg, 0.18 mmol, 45%), with second stage lasting for 4h. The title compound was isolated as yellow oil after chromatography on silica gel (15 g column, Hexane:Ethyl Acetate 9:1); ^{1}H NMR (400 MHz, $CDCl_3$) δ 7.92 – 7.88 (m, 2H), 7.60 – 7.54 (m, 1H), 7.53 – 7.47 (m, 2H), 7.37 (s, 5H), 6.36 (s, 1H), 5.74 (s, 1H), 5.27 (s, 1H), 2.64 (s, 3H); $^{13}C\{^1H\}$ NMR (101 MHz, $CDCl_3$) δ 156.4, 152.7, 142.4, 138.3, 138.0, 133.1, 129.2, 128.4, 128.4, 128.1, 126.8, 124.3, 113.6, 108.2, 13.1; IR (CH_2Cl_2): 1319 (S=O), 1153 (S=O) cm^{-1} HRMS (ESI) m/z: $[M+Na]^+$ Calcd for $C_{19}H_{16}O_3NaS$: 347.0718, Found 347.0717

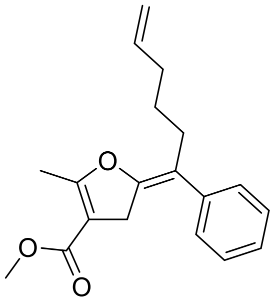

(28) methyl (E)-2-methyl-5-(1-phenylhex-5-en-1-ylidene)-4,5-dihydrofuran-3-carboxylate Prepared in reaction of methyl 2-acetylhept-6-enoate (177.8 mg, 0.80 mmol) with bromobenzene (138.17 mg, 0.88 mmol) under **conditions B** (227.0 mg, 0.76 mmol, 95%). The title compound was isolated as yellow oil after chromatography on silica gel (15 g column, Hexane:Ethyl Acetate 98:2); ^{1}H NMR (600 MHz, $CDCl_3$) δ 7.35 – 7.31 (m, 2H), 7.24 – 7.20 (m, 3H), 5.81 – 5.73 (m, 1H), 4.96 (dq, J = 17.1, 1.6 Hz, 1H), 4.93 – 4.89 (m, 1H), 3.67 (s, 3H), 3.51 – 3.48 (m, 2H), 2.55 – 2.50 (m, 2H), 2.31 (t, J = 1.8 Hz, 3H), 2.05 (q, J = 6.9 Hz, 2H), 1.43 (p, J = 7.6 Hz, 2H); $^{13}C\{^1H\}$ NMR (151 MHz, $CDCl_3$) δ 165.9, 165.3, 149.8, 139.7, 138.7, 128.5, 128.1, 126.5, 115.9, 114.4, 103.5, 50.9, 33.4, 33.2, 29.8, 27.3, 13.7; IR (CH_2Cl_2): 1703 (C=O), 1224 (C-O), 1071 (C-O) cm^{-1} HRMS (EI) m/z: $[M]^+$ Calcd for $C_{19}H_{22}O_3$: 298.1569, Found 298.1560

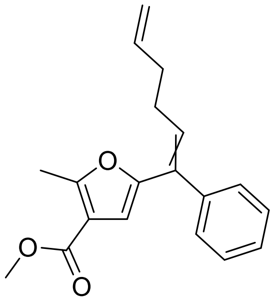

(29a/b) methyl 2-methyl-5-(1-phenylhexa-1,5-dien-1-yl)furan-3-carboxylate Prepared in reaction of methyl (E)-2-methyl-5-(1-phenylhex-5-en-1-ylidene)-4,5-dihydrofuran-3-carboxylate (119.4 mg, 0.40 mmol) with DDQ (100.0 mg, 0.44 mmol) under **conditions C** (80.5 mg, 0.27 mmol, 68%). The title compound was isolated in mixture of E/Z isomers (E/Z 4/1) as transparent oil after chromatography on silica gel (15 g column, Hexane:Ethyl Acetate 98:2); **Isomer E:** ^{1}H NMR (500 MHz, $CDCl_3$) δ 7.41 – 7.28 (m, 3H), 7.25 – 7.21 (m, 2H), 6.30 – 6.24 (m, 1H), 5.96 (s, 1H), 5.83 – 5.73 (m, 1H), 5.04 – 4.94 (m, 1H), 3.75 (s, 3H), 2.61 (s, 3H), 2.20 – 2.12 (m, 4H); ^{13}C{^{1}H} NMR (126 MHz, $CDCl_3$) δ 164.5, 158.6, 153.2, 137.9, 136.9, 131.5, 129.6, 128.3, 127.5, 126.5, 115.0, 114.6, 107.8, 51.2, 33.8, 28.2, 13.8; **Isomer Z:** ^{1}H NMR (500 MHz, $CDCl_3$) δ 7.41 – 7.27 (m, 3H), 7.25 – 7.20 (m, 2H), 6.45 (s, 1H), 5.93 – 5.83 (m, 1H), 5.09 (dd, *J* = 17.1, 1.8 Hz, 1H), 5.04 – 4.93 (m, 1H), 3.81 (s, 3H), 2.60 (s, 3H), 2.21 – 2.10 (m, 4H); ^{13}C{^{1}H} NMR (126 MHz, $CDCl_3$) δ 164.5, 158.7, 151.2, 141.0, 138.0, 132.6, 131.2, 128.1, 128.1, 127.4, 115.1, 114.2, 111.1, 51.3, 33.8, 29.1, 13.9; IR (CH_2Cl_2): 1720 (C=O), 1233 (C-O), 1069 (C-O) cm^{-1} HRMS (EI) m/z: [M]$^+$ Calcd for $C_{19}H_{20}O_3$: 296.1412, Found 296.1410

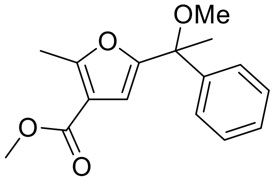

(31) methyl 5-(1-methoxy-1-phenylethyl)-2-methylfuran-3-carboxylate Prepared in reaction of methyl (E)-2-methyl-5-(1-phenylethylidene)-4,5-dihydrofuran-3-carboxylate (97.2 mg, 0.40 mmol) with DDQ (100.0 mg, 0.44 mmol) under modified **conditions C** (64.7 mg, 0.24 mmol, 59%) with MeOH (1.0 mL) used as solvent. The title compound was isolated as transparent oil after chromatography on silica gel (15 g column, Hexane:Ethyl Acetate 98:2); ^{1}H NMR (500 MHz, $CDCl_3$) δ 7.39 – 7.31 (m, 4H), 7.29 – 7.25 (m, 1H), 6.54 (s, 1H), 3.81 (s, 3H), 3.19 (s, 3H), 2.54 (s, 3H), 1.79 (s, 3H); ^{13}C{^{1}H} NMR (126 MHz, $CDCl_3$) δ 164.4, 159.5, 154.4, 143.6, 128.1, 127.3, 126.0, 113.4, 110.0, 51.3, 51.3, 25.0, 13.9; IR (CH_2Cl_2): 1719 (C=O), 1231 (C-O), 1105 (C-O) cm^{-1} HRMS (EI) m/z: [M]$^+$ Calcd for $C_{16}H_{18}O_4$: 274.1205, Found 274.1216

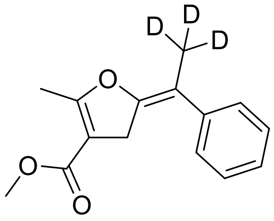

(32) methyl (E)-2-methyl-5-(1-phenylethylidene-2,2,2-d3)-4,5-dihydrofuran-3-carboxylate Prepared in reaction of **methyl 2-acetylhex-4-ynoate-6,6,6-*d$_3$*** (137.0 mg, 0.80 mmol) with bromobenzene (138.2 mg, 0.88 mmol) under **conditions B** (139.7 mg, 0.56 mmol, 71%). The title compound was isolated as off-white solid after chromatography on silica gel (15 g column, Hexane:Ethyl Acetate 98:2); ^{1}H NMR (500 MHz, $CDCl_3$) δ 7.34 – 7.32 (m, 2H), 7.30 – 7.27 (m, 2H), 7.24 – 7.19 (m, 1H), 3.70 (s, 3H), 3.61 (brs, 2H), 2.33 (t, *J* = 1.8 Hz, 3H); ^{13}C{^{1}H} NMR (126 MHz, $CDCl_3$) δ 165.8, 165.3, 149.8, 140.6, 128.4, 127.2, 126.3, 110.7, 103.6, 51.0, 33.4, 13.7; IR (CH_2Cl_2): 1710 (C=O), 1225 (C-O), 1072 (C-O) cm^{-1} HRMS (EI) m/z: [M]$^+$ Calcd for $C_{15}H_{13}D_3O_3$: 247.1288, Found 247.1287

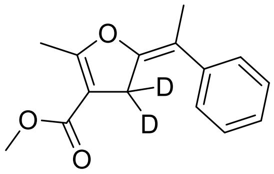

(33) methyl (E)-2-methyl-5-(1-phenylethylidene)-4,5-dihydrofuran-3-carboxylate-4,4-d2 Prepared in reaction of **methyl 2-acetylhex-4-ynoate-3,3-d2** (136.2 mg, 0.80 mmol) with bromobenzene (138.2 mg, 0.88 mmol) under **conditions B** (157.6 mg, 0.64 mmol, 80%). The title compound was isolated as off-white oil after chromatography on silica gel (15 g column, Hexane:Ethyl Acetate 98:2); ^{1}H NMR (400 MHz, $CDCl_3$) δ 7.37 – 7.31 (m, 2H), 7.31 – 7.27 (m, 2H), 7.24 – 7.19 (m, 1H), 3.70 (s, 3H), 2.33 (s, 3H), 2.08 (s, 3H); ^{13}C{^{1}H} NMR (101 MHz, $CDCl_3$) δ 165.8, 165.3, 149.8, 140.6, 127.3, 126.3, 50.9, 13.7; IR (CH_2Cl_2): 1709 (C=O), 1260 (C-O), 1080 (C-O) cm^{-1} HRMS (EI) m/z: [M]$^+$ Calcd for $C_{15}H_{14}D_2O_3$: 246.1225, Found 246.1234

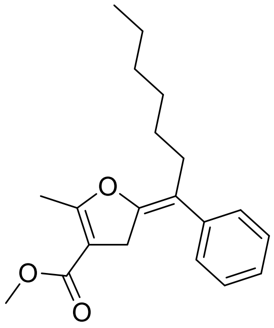

(36) methyl (E)-2-methyl-5-(1-phenylheptylidene)-4,5-dihydrofuran-3-carboxylate Prepared in reaction of methyl 2-acetylundec-4-ynoate (190.7 mg, 0.80 mmol) with bromobenzene (138.17 mg, 0.88 mmol) under **conditions B** (221.0 mg, 0.70 mmol, 88%). The title compound was isolated as brown oil after chromatography on silica gel (15 g column, Hexane:Ethyl Acetate 98:2); ^{1}H NMR (500 MHz, $CDCl_3$) δ 7.36 – 7.31 (m, 2H), 7.24 – 7.20 (m, 3H), 3.68 (s, 3H), 3.52 – 3.49 (m, 2H), 2.51 (t, *J* = 7.2 Hz, 2H), 2.33 – 2.30 (m, 3H), 1.36 – 1.28 (m, 4H), 1.28 – 1.20 (m, 4H), 0.86 (t, *J* = 6.9 Hz, 3H); ^{13}C{^{1}H} (126 MHz, $CDCl_3$) δ 166.0, 165.4, 149.5, 139.8, 128.4, 128.1, 126.4, 116.3, 103.4, 50.9, 33.1, 31.6, 30.3, 29.0, 28.0, 22.6, 14.1, 13.7; IR (CH_2Cl_2): 1719 (C=O), 1234 (C-O), 1092 (C-O) cm^{-1} HRMS (EI), m/z: [M]$^{+}$ Calcd for $C_{20}H_{26}O_3$: 314.1882, Found 314.1887

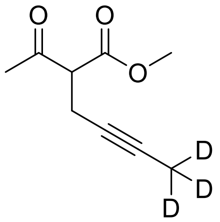

methyl 2-acetylhex-4-ynoate-6,6,6-d3 Prepared in reaction of methyl acetoacetate (696.7 mg, 6.00 mmol) with **but-2-yn-1-yl-4,4,4-d3 methanesulfonate** (925.3 mg, 6.12 mmol) under **conditions D** (491.9 mg, 2.9 mmol, 48%). The title compound was isolated as yellow oil after chromatography on silica gel (100 g column, Hexane:Ethyl Acetate 95:5); ^{1}H NMR (500 MHz, $CDCl_3$) δ 3.73 (s, 3H), 3.62 (t, *J* = 7.6 Hz, 1H), 2.69 – 2.58 (m, 2H), 2.26 (s, 3H); ^{13}C{^{1}H} NMR (126 MHz, $CDCl_3$) δ 201.6, 168.9, 77.7 (m), 74.9, 58.5, 52.5, 29.4, 17.9; IR (CH_2Cl_2): 2126 (C≡C) 1710 (C=O), 1225 (C-O), 1072 (C-O) cm^{-1}; HRMS (APCI), m/z: [M+H]$^{+}$ Calcd for $C_9H_{10}D_3O_3$: 172.1053, Found 172.1054

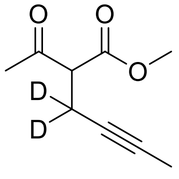

methyl 2-acetylhex-4-ynoate-3,3-d2 Prepared in reaction of methyl acetoacetate (812.8 mg, 7.00 mmol) with **But-2-yn-1-yl-1,1-*d*$_2$ methanesulfonate** (1072.0 mg, 7.1 mmol) under **conditions D** (347.7 mg, 2.0 mmol, 29%). The title compound was isolated as yellow oil after chromatography on silica gel (250 g column, Hexane:Ethyl Acetate 95:5); ^{1}H NMR (400 MHz, $CDCl_3$) δ 3.73 (s, 3H), 3.62 (s, 1H), 2.27 (s, 3H), 1.72 (s, 3H); ^{13}C{^{1}H} NMR (101 MHz, $CDCl_3$) δ 201.6, 168.9, 74.8, 58.4, 52.5, 29.4, 3.3; IR (CH_2Cl_2): 2116 (C≡C) 1718 (C=O), 1220 (C-O), 1152 (C-O) cm^{-1}; HRMS (APCI), m/z: [M+H]$^{+}$ Calcd for $C_9H_{11}D_2O_3$: 171.0991, Found 171.0990

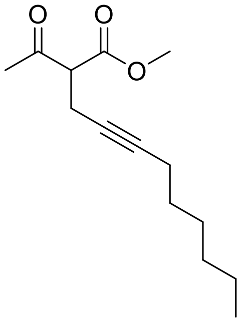

methyl 2-acetylundec-4-ynoate Prepared in reaction of methyl acetoacetate (232.2 mg, 2.00 mmol) with **non-2-yn-1-yl methanesulfonate** (441.2 mg, 2.04 mmol) under **conditions D** (181.0 mg, 0.76 mmol, 38%). The title compound was isolated as yellow oil after chromatography on silica gel (30 g column, Hexane:Ethyl Acetate 95:5 ^{1}H NMR (600 MHz, $CDCl_3$) δ 3.74 (s, 3H), 3.65 (t, *J* = 7.6 Hz, 1H), 2.72 – 2.63 (m, 2H), 2.28 (s, 3H), 2.09 (tt, *J* = 7.1, 2.4 Hz, 2H), 1.45 – 1.40 (m, 2H), 1.35 – 1.21 (m, 6H), 0.88 (t, *J* = 7.1 Hz, 3H); ^{13}C{^{1}H} NMR (151 MHz, $CDCl_3$) δ 201.7, 168.9, 82.7, 75.7, 58.7, 52.6, 31.3, 29.5, 28.8, 28.4, 22.5, 18.6, 18.0, 14.0; HRMS (EI), m/z: [M]$^{+}$ Calcd for $C_{14}H_{22}O_3$: 238.1569, Found 238.1566

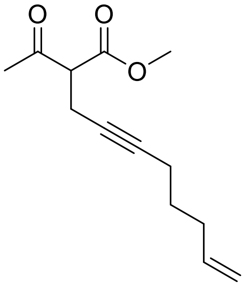

methyl 2-acetyldec-9-en-4-ynoate Prepared in reaction of methyl acetoacetate (928.9 mg, 8.00 mmol) with **oct-7-en-2-yn-1-yl methanesulfonate** (1650.5 mg, 8.16 mmol) under **conditions D** (686.5 mg, 3.1 mmol, 39%). The title compound was isolated as yellow oil after chromatography on silica gel (250 g column, Hexane:Ethyl Acetate 95:5 ^{1}H NMR (500 MHz, $CDCl_3$) δ 5.80 – 5.70 (m, 1H), 5.00 (dq, *J* = 17.2, 1.5 Hz, 1H), 4.97 – 4.93 (m, 1H), 3.73 (s, 3H), 3.64 (t, *J* = 7.6 Hz, 1H), 2.72 – 2.62 (m, 2H), 2.27 (s, 3H), 2.13 – 2.05 (m, 4H), 1.52 (p, *J* = 7.2 Hz, 2H); ^{13}C{^{1}H} NMR (126 MHz, $CDCl_3$) δ 201.5, 168.9, 137.8, 115.0, 82.2, 76.1, 58.7, 52.5, 32.6, 29.4, 27.9, 18.0, 18.0; HRMS (APCI), m/z: [M+H]$^{+}$ Calcd for $C_{13}H_{19}O_3$: 223.1334, Found 223.1331

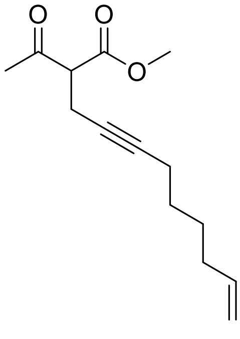

methyl 2-acetylundec-10-en-4-ynoate Prepared in reaction of methyl acetoacetate (928.9 mg, 8.00 mmol) with **non-8-en-2-yn-1-yl methanesulfonate** (1765.0 mg, 8.16 mmol) under **conditions D** (1189.0 mg, 5.0 mmol, 63%). The title compound was isolated as yellow oil after chromatography on silica gel (250 g column, Hexane:Ethyl Acetate 95:5 ^{1}H NMR (400 MHz, $CDCl_3$) δ 5.83 -5.70 (m, 1H), 4.98 (dq, *J* = 17.1, 1.6 Hz, 1H), 4.95 – 4.91 (m, 1H), 3.73 (s, 3H), 3.63 (t, *J* = 7.6 Hz, 1H), 2.72 – 2.60 (m, 2H), 2.26 (s, 3H), 2.13 – 2.06 (m, 2H), 2.06 – 1.98 (m, 2H), 1.48 – 1.39 (m, 4H); $^{13}C\{^1H\}$ NMR (101 MHz, $CDCl_3$) δ 201.5, 168.9, 138.5, 114.5, 82.4, 75.9, 58.7, 52.5, 33.2, 29.4, 28.2, 27.9, 18.4, 18.0; HRMS (APCI), m/z: $[M+H]^+$ Calcd for $C_{14}H_{21}O_3$: 237.1491, Found 237.1493

methyl 2-acetylhex-4-ynoate Prepared in concordance with previously reported reaction[3] of methyl 3-oxobutanoate (2.32 g, 20.0 mmol) with 1-bromobut-2-yne (2.71 g, 20.4 mmol) under **conditions D** (2.06 g, 12.3 mmol 61%). The title compound was isolated as yellowish oil after chromatography on silica gel (250 g column, Hexane:Ethyl Acetate 9:1) ^{1}H NMR (400 MHz, $CDCl_3$) δ 3.73 (s, 3H), 3.62 (t, *J* = 7.5 Hz, 1H), 2.66-2.60 (m, 2H), 2.26 (s, 3H), 1.71 (t, *J* = 2.5 Hz, 3H); $^{13}C\{^1H\}$ NMR (101 MHz, $CDCl_3$) δ 201.2, 168.9, 77.8, 74.8, 58.6, 52.51, 29.4, 17.9, 3.3;

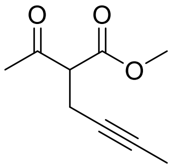

isopropyl 2-acetylhex-4-ynoate Prepared in concordance with previously reported reaction[3] of isopropyl 3-oxobutanoate (1.44 g, 10.0 mmol) with 1-bromobut-2-yne (1.36 g, 10.2 mmol) under **conditions D** (0.83 g, 4.56 mmol 46%). The title compound was isolated as yellowish oil after chromatography on silica gel (200 g column, Hexane:MTBE 98:2 → 95:5) ^{1}H NMR (400 MHz, $CDCl_3$) δ 5.04 (sept, *J* = 6.3 Hz, 1H), 3.54 (t, *J* = 7.6 Hz, 1H), 2.67-2.55 (m, 2H), 2.23 (s, 3H), 1.70 (t, *J* = 2.6 Hz, 3H), 1.23 (d, *J* = 3.6 Hz, 3H), 1.22 (d, *J* = 3.6 Hz, 3H); $^{13}C\{^1H\}$ NMR (101 MHz, $CDCl_3$) δ 201.6, 168.0, 77.6, 75.0, 69.2, 59.0, 29.2, 21.6, 21.4, 17.8, 3.3;

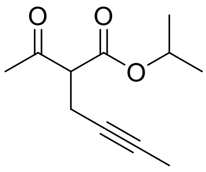

tert-butyl 2-acetylhex-4-ynoate Prepared in concordance with previously reported reaction[3] of tert-butyl 3-oxobutanoate (1.44 g, 10.0 mmol) with 1-bromobut-2-yne (1.36 g, 10.2 mmol) under **conditions D** (0.85 g, 4.03 mmol 40%). The title compound was isolated as yellowish oil after chromatography on silica gel (200 g column, Hexane:MTBE 98:2 → 95:5) ^{1}H NMR (400 MHz, $CDCl_3$) δ 3.49 (t, *J* = 7.6 Hz, 1H), 2.60-2.54 (m, 2H), 2.24 (s, 3H), 1.71 (t, *J* = 2.6 Hz, 3H), 1.44 (s, 9H); $^{13}C\{^1H\}$ NMR (101 MHz, $CDCl_3$) δ 202.0, 167.6, 82.2, 77.4, 75.2, 59.8, 29.2, 27.8, 17.8, 3.3;

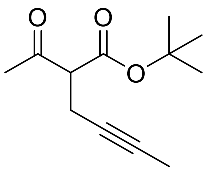

ethyl 3-oxo-3-phenylpropanoate Prepared in concordance with previously reported reaction[3] of ethyl 3-oxo-3-phenylpropanoate (1.92 g, 10.0 mmol) with 1-bromobut-2-yne (1.36 g, 10.2 mmol) under **conditions D** (1.77 g, 7.23 mmol 72%). The title compound was isolated as yellowish oil after chromatography on silica gel (250 g column, Hexane:Dioxane 9:1) followed by second chromatography of impure fraction (100g column, Hex:Dioxane 9:1) ^{1}H NMR (400 MHz, $CDCl_3$) δ 8.03-7.99 (m, 2H), 7.60-7.54 (m, 1H), 7.50-7.43 (m, 2H), 4.50 (t, J = 7.4 Hz, 1H), 4.14 (q, J = 7.1 Hz, 2H), 3.19 (s, 1H), 2.90-2.72 (m, 2H), 1.68 (t, J = 2.6 Hz, 3H), 1.16 (t, J = 7.2 Hz, 3H); $^{13}C\{^1H\}$ NMR (101 MHz, $CDCl_3$) δ 193.8, 168.6, 136.08, 133.5, 128.7, 128.6, 77.8, 75.2, 61.5, 53.7, 18.7, 13.9, 3.3;

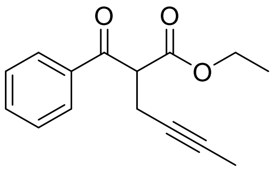

ethyl 2-isobutyrylhex-4-ynoate Prepared in concordance with previously reported reaction[3] of ethyl 4-methyl-3-oxopentanoate (1.43 g, 9.95 mmol) with 1-bromobut-2-yne (1.38 g, 10.4 mmol) under **conditions D** (1.28 g, 6.52 mmol 66%). The title compound was isolated as colorless oil after chromatography on silica gel (200 g column, Hexane:MTBE 99.5:0.5 → 98:2) ^{1}H NMR (400 MHz, $CDCl_3$) δ 3.83 (dd, *J* = 8.0, 7.2 Hz, 1H), 3.70 (s, 3H), 2.83 (sept, *J* = 6.9 Hz, 1H), 2.68–2.58 (m, 2H), 1.70 (t, *J* = 2.6 Hz, 3H), 1.12 (d, *J* = 2.6 Hz, 3H), 1.10 (d, *J* = 2.4 Hz, 3H); $^{13}C\{^1H\}$ NMR (101 MHz, $CDCl_3$) δ 207.7, 168.9, 77.6, 75.2, 55.8, 52.4, 41.1, 18.2, 17.9, 17.7, 3.3;

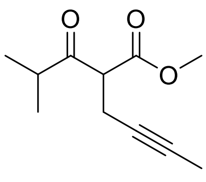

methyl 2-acetylhept-4-ynoate Prepared in concordance with previously reported reaction[3] of methyl 3-oxobutanoate (1.16 g, 10.0 mmol) with 1-bromopent-2-yne (1.50 g, 10.2 mmol) under **conditions D** (1.20 g, 6.6 mmol 66%). The title compound was isolated as colorless oil after chromatography on silica gel (200 g column, Hexane:Dioxane 98:2 → 95:5) ^{1}H NMR (400 MHz, $CDCl_3$) δ 3.71 (s, 3H), 3.62 (t, *J* = 7.6 Hz, 1H), 2.67-2.61 (m, 2H), 2.25 (s, 3H), 2.12-2.03 (m, 2H), 1.04 (t, *J* = 7.5 Hz, 3H); ^{13}C{^{1}H} NMR (101 MHz, $CDCl_3$) δ 201.6, 168.9, 83.9, 75.0, 58.6, 52.5, 29.4, 17.9, 13.9, 12.2;

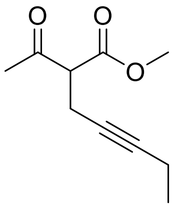

3-(but-2-yn-1-yl)pentane-2,4-dione Prepared in concordance with previously reported reaction[3] of pentane-2,4-dione (4.50 g, 45.0 mmol) with 1-bromobut-2-yne (1.20 g, 9.0 mmol) under **conditions E** (1.14 g, 7.5 mmol 83%). The title compound was isolated as yellow oil after chromatography on silica gel (250 g column, Hexane:Diethyl Ether 98:2 → 9:1) ^{1}H NMR (400 MHz, $CDCl_3$) δ 3.01 (q, *J* = 2.6 Hz, 2H), 2.18 (s, 6H), 1.74 (t, *J* = 2.6 Hz, 3H), Minor tautomer signals: 3.76 (t, *J* = 7.6 Hz, 1H), 2.61 (dq, *J* = 7.6, 2.6 Hz, 2H), 2.20 (s, 6H), 1.71 (t, *J* = 2.6 Hz, 3H); ^{13}C{^{1}H} NMR (101 MHz, $CDCl_3$) δ 202.8, 190.7, 107.4, 76.4, 75.9, 74.8, 67.3, 29.1, 23.0, 17.8, 17.5, 3.3, 3.3.

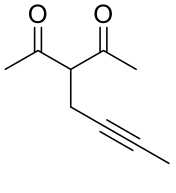

2-(but-2-yn-1-yl)-1-phenylbutane-1,3-dione Prepared in concordance with previously reported reaction[3] of 1-phenylbutane-1,3-dione (0.81 g, 5.0 mmol) with 1-bromobut-2-yne (0.33 g, 2.5 mmol) under **conditions E** (0.43 g, 2.0 mmol 81%). The title compound was isolated as yellow solid after chromatography on silica gel (100 g column, Hexane:Ethyl Acetate 98:2 → 9:1) ^{1}H NMR (400 MHz, $CDCl_3$) δ 8.03 – 7.98 (m, 2H), 7.62 – 7.56 (m, 1H), 7.51 – 7.45 (m, 2H), 4.63 (dd, *J* = 8.2, 6.6 Hz, 1H), 2.87 (m, 1H), 2.76 – 2.67 (m, 1H), 2.18 (s, 3H), 1.68 (t, J = 2.6 Hz, 3H):; ^{13}C{^{1}H} NMR (101 MHz, $CDCl_3$) δ 202.5, 195.1, 136.2, 133.8, 128.8, 128.8, 78.3, 75.1, 62.1, 28.1, 18.7, 3.3;

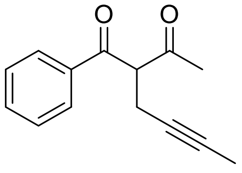

2-(but-2-yn-1-yl)-1,3-diphenylpropane-1,3-dione Prepared in concordance with previously reported reaction[3] of 1,3-diphenylpropane-1,3-dione (1.13 g, 5.0 mmol) with 1-bromobut-2-yne (0.36 g, 2.7 mmol) under **conditions E** (0.67 g, 2.4 mmol 90%). The title compound was isolated as yellow solid after chromatography on silica gel (100 g column, Hexane:Dioxane 98:2 → 9:1) ^{1}H NMR (400 MHz, $CDCl_3$) δ 8.01 – 7.94 (m, 4H), 7.59 – 7.52 (m, 2H), 7.44 (t, *J* = 7.7 Hz, 1H), 5.45 (t, *J* = 7.0 Hz, 1H), 2.94 (dq, *J* = 7.3, 2.5 Hz, 2H), 1.62 (t, *J* = 2.6 Hz, 3H); ^{13}C{^{1}H} NMR (101 MHz, $CDCl_3$) δ 194.9, 136.0, 133.5, 128.8, 128.7, 75.9, 56.0, 19.4, 3.3;

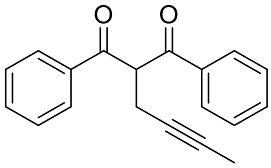

3-(phenylsulfonyl)hept-5-yn-2-one Prepared in concordance with previously reported reaction[3] of 1-(phenylsulfonyl)propan-2-one (1.99 g, 10.0 mmol) with 1-bromobut-2-yne (1.35 g, 10.2 mmol) under **conditions D** (1.33 g, 5.3 mmol 53%). The title compound was isolated as white solid after chromatography on silica gel (150 g column, Hexane:Ethyl Acetate 9:1 → 8:2) ^{1}H NMR (400 MHz, $CDCl_3$) δ 7.82 – 7.77 (m, 2H), 7.71 – 7.65 (m, 1H), 7.59 – 7.51 (m, 2H), 4.21 (dd, *J* = 10.0, 5.1 Hz, 1H), 2.80 – 2.66 (m, 2H), 2.42 (s, 3H), 1.64 (t, *J* = 2.5 Hz, 3H); ^{13}C{^{1}H} NMR (101 MHz, $CDCl_3$) δ 198.6, 136.5, 134.5, 129.2, 129.1, 79.1, 74.0, 72.4, 31.4, 17.4, 3.3;

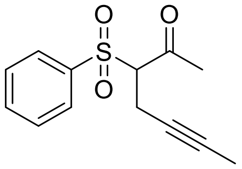

3-(methylsulfonyl)hept-5-yn-2-one Prepared in concordance with previously reported reaction[3] of 1-(methylsulfonyl)propan-2-one (1.37 g, 10.1 mmol) with 1-bromobut-2-yne (1.33 g, 10.0 mmol) under **conditions D** (0.58 g, 3.1 mmol 31%). The title compound was isolated as white solid after chromatography on silica gel (150 g column, Hexane:Ethyl Acetate 9:1 → 8:2) ^{1}H NMR (400 MHz, $CDCl_3$) δ 4.05 (dd, *J* = 8.2, 6.8 Hz, 1H), 2.94 – 2.86 (m, 5H), 2.47 (s, 3H), 1.75 (t, *J* = 2.6 Hz, 3H); ^{13}C{^{1}H} NMR (101 MHz, $CDCl_3$) δ 200.5, 79.6, 72.7, 72.3, 38.6, 31.5, 17.4, 3.3.

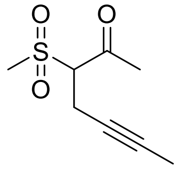

Computational Studies

General.

All calculations were performed using Gaussian 09 package.[5] Structures of minima and transition states were optimized at M06-2X/6-31G(d) level of theory.[6] Frequency calculations were performed at the same level of theory to confirm the nature of stationary points and provide corrections to thermodynamic functions. Single point energies were computed at M06-2X/6-311++g(d,p) level of theory with solvation (dichloroethane or DMF) according to SMD model[7] Molecular structures were visualized in CYLview.[8]

Gibbs free energy of computed structures in various solvents.

Reported structures were calculated at SMD(solvent)/M06-2X/6-311++G(d,p)//M06-2X/6-31G(d) level of theory. Reported values of Gibbs free energy of intermediates and transition states given relative to starting materials (DDQ and 2).

**Structure**	G(DCE) (kJ/mol)	G(DMF) (kJ/mol)
**IM1**	8.6	8.4
**TS1**	61.8	60.8
**IM2**	-56.1	-54.7
**TS2**	-44.0	-41.3
**IM3**	-134.8	-127.4
**2 + DDQH2**	-137.5	-126.7
**TS3**	79.5	77.5
**TS4**	102.8	102.4
**TS5**	94.7	90.9
**TS6**	123.2	121.9
**Radicals (H transfer)**	121.1	125.4

Optimized geometries, energies and corrections to thermodynamic functions.

DDQ

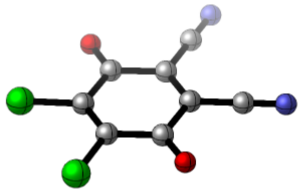

E (M06-2X/6-31G(d)) = -1484.83009956
E (SMD(dichloroethane)/M06-2X /6-311++g(d,p)//M06-2X/6-31G(d)) = -1485.06457250
E (SMD(DMF)/M06-2X /6-311++g(d,p)//M06-2X/6-31G(d)) = -1485.06757436

Zero-point correction= 0.064277 (Hartree/Particle)
Thermal correction to Energy= 0.076612
Thermal correction to Enthalpy= 0.077556
Thermal correction to Gibbs Free Energy= 0.024056

Charge = 0 Multiplicity = 1

O	-0.13514400	-2.65929100	0.00041200
C	-0.11641300	-1.45570600	0.00006900
C	-1.39969500	-0.67369100	-0.00020200
C	1.16017600	-0.67321300	-0.00005100

C	-1.39978600	0.67361500	0.00008900
C	-2.60873700	-1.44227600	-0.00031400
C	1.16011600	0.67326200	-0.00016600
Cl	2.58692600	-1.60749400	0.00025600
C	-0.11650900	1.45563100	-0.00000500
C	-2.60879900	1.44217500	0.00047900
N	-3.58663000	-2.05885800	-0.00071600
Cl	2.58684100	1.60758900	-0.00032400
O	-0.13527000	2.65925000	0.00004000
N	-3.58663300	2.05884800	0.00045100

methyl (E)-2-methyl-5-(1-phenylethylidene)-4,5-dihydrofuran-3-carboxylate

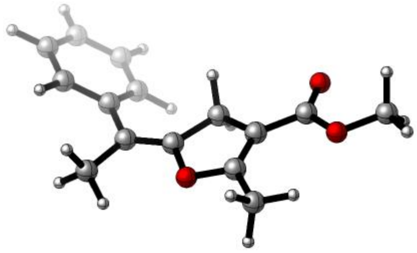

E (M06-2X/6-31G(d)) = -806.550616063
E (SMD(dichloroethane)/M06-2X /6-311++g(d,p)//M06-2X/6-31G(d)) = -806.793025560
E (SMD(DMF)/M06-2X /6-311++g(d,p)//M06-2X/6-31G(d)) = -806.792159507

Zero-point correction=	0.281969 (Hartree/Particle)
Thermal correction to Energy=	0.299317
Thermal correction to Enthalpy=	0.300261
Thermal correction to Gibbs Free Energy=	0.235655

Charge = 0 Multiplicity = 1

C	-1.94140800	1.28784100	-0.10219700
C	-1.88523500	-0.05480900	-0.04435400
C	-0.45432500	-0.48882600	-0.23378200
O	-0.71027200	1.85242700	-0.25954400
C	0.25734500	0.84515900	-0.24364600
C	1.54892600	1.18930000	-0.15910200
C	-2.95112700	-1.04538700	0.08020100
O	-4.18143100	-0.51367800	0.24858200
O	-2.75455700	-2.23966800	0.03862400
C	-5.22815800	-1.47463100	0.36834700
H	-5.28579200	-2.09153700	-0.53103700
H	-5.05407800	-2.12335900	1.22944200
H	-6.14394700	-0.89991700	0.49921500
C	2.62350100	0.17434300	-0.06682700
C	3.76166600	0.44273000	0.70890000
C	2.56688600	-1.05276000	-0.74123200
C	4.78464500	-0.49027100	0.83131200
H	3.84096900	1.39045000	1.23276100
C	3.59100700	-1.98659700	-0.62089300
H	1.72993300	-1.26818300	-1.39577700
C	4.70276700	-1.71342100	0.17037400
H	5.64958100	-0.26036300	1.44625300
H	3.52234300	-2.92750000	-1.15844400

H	5.50180400	-2.44208700	0.26400100
C	-3.04704000	2.27832400	-0.03389200
H	-4.00799500	1.77873500	0.06020600
H	-2.88879700	2.94191500	0.82194500
H	-3.03436100	2.89736200	-0.93621800
H	-0.35077500	-1.04082700	-1.17588400
C	1.94200900	2.64533500	-0.07680900
H	1.14650700	3.28870000	-0.45349300
H	2.14786200	2.94493900	0.95776600
H	2.84988100	2.82911700	-0.65890800
H	-0.08181000	-1.14807500	0.55846800

IM1

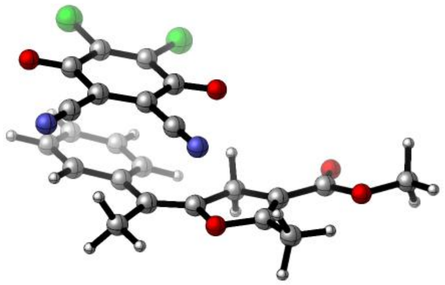

E (M06-2X/6-31G(d)) = -2291.41038536
E (SMD(dichloroethane)/M06-2X /6-311++g(d,p)//M06-2X/6-31G(d)) = -2291.88016709
E (SMD(DMF)/M06-2X /6-311++g(d,p)//M06-2X/6-31G(d)) = -2291.88235372

Zero-point correction=	0.348302 (Hartree/Particle)
Thermal correction to Energy=	0.379025
Thermal correction to Enthalpy=	0.379970
Thermal correction to Gibbs Free Energy=	0.285540

Charge = 0 Multiplicity = 1

C	3.53518400	1.14180100	0.41520700
C	3.46013600	-0.19876900	0.44107600
C	2.15649300	-0.59890800	1.07269600
O	2.41971400	1.72091500	0.95976100
C	1.49773100	0.74353800	1.27769100
C	0.26140300	1.13919300	1.65846300
C	4.41232100	-1.21709000	0.00065500
O	5.52034400	-0.71433400	-0.57897500
O	4.22365400	-2.40395700	0.14687200
C	6.45727400	-1.69737400	-1.01615200
H	6.80639900	-2.29427200	-0.17084900
H	5.99723800	-2.36098400	-1.75121000
H	7.28145600	-1.14242800	-1.46158600
C	-0.85254500	0.20713900	1.93053100
C	-2.16522900	0.71615600	1.98153500
C	-0.69831800	-1.18049300	2.10509700
C	-3.26449500	-0.11708600	2.17039200
H	-2.33784000	1.78279000	1.87653600
C	-1.79892300	-2.01145900	2.29295000
H	0.28518000	-1.62877200	2.12779000
C	-3.08887800	-1.48970500	2.32147700

H	-4.26188400	0.31273900	2.18651700
H	-1.63974400	-3.07777400	2.42111300
H	-3.94472900	-2.14200000	2.46079800
C	4.53046400	2.12437500	-0.07932000
H	5.41037800	1.61747200	-0.46809800
H	4.06451900	2.72702400	-0.86562900
H	4.81495100	2.79883500	0.73403300
H	2.31812300	-1.12396800	2.02411600
C	-0.01353100	2.61939300	1.79127000
H	0.90374300	3.19965200	1.71232500
H	-0.69892500	2.98550100	1.01682900
H	-0.48142300	2.83229300	2.75794400
H	1.57524700	-1.27199100	0.43153700
O	0.62381400	-0.77034700	-1.51595300
C	-0.48951600	-0.34962000	-1.31396800
C	-0.74771400	1.12663400	-1.27973400
C	-1.66242500	-1.25782700	-1.13246000
C	-1.99760700	1.61039100	-1.12128200
C	0.39759700	1.96782000	-1.46448600
C	-2.91153100	-0.77434900	-1.00332200
Cl	-1.30330600	-2.92750200	-1.17372800
C	-3.17951200	0.69849700	-0.96792900
C	-2.28271500	3.01503800	-1.10533800
N	1.32839300	2.63684200	-1.62061800
Cl	-4.29350900	-1.77124500	-0.90006700
O	-4.29049200	1.14996300	-0.84853900
N	-2.50797700	4.14928400	-1.08413500

TS1

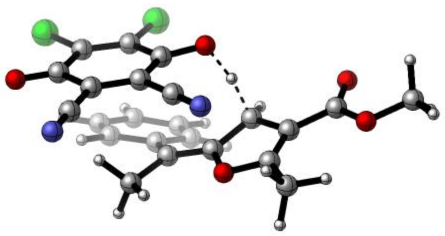

E (M06-2X/6-31G(d)) = -2291.37830856
E (SMD(dichloroethane)/M06-2X /6-311++g(d,p)//M06-2X/6-31G(d)) = -2291.85538071
E (SMD(DMF)/M06-2X /6-311++g(d,p)//M06-2X/6-31G(d)) = -2291.85788875

Zero-point correction=	0.342639 (Hartree/Particle)
Thermal correction to Energy=	0.372531
Thermal correction to Enthalpy=	0.373475
Thermal correction to Gibbs Free Energy=	0.281009

Charge = 0 Multiplicity = 1

C	3.19334600	0.65160600	1.03758500
C	3.25177900	-0.42430400	0.21060300
C	1.91729500	-1.01128300	0.16336900
O	1.95043200	0.75396300	1.58626200
C	1.16539100	-0.28435800	1.15515600
C	-0.15259100	-0.33034300	1.58026900
C	4.36069400	-0.95807700	-0.59381200

O	5.49706000	-0.25594000	-0.46569800
O	4.25048400	-1.93706700	-1.29352200
C	6.59101900	-0.74702300	-1.24379400
H	6.83512000	-1.76994300	-0.95042400
H	6.33560700	-0.73154100	-2.30498500
H	7.42308900	-0.07651100	-1.03711500
C	-0.98959200	-1.53095900	1.43209000
C	-2.38988500	-1.41584000	1.49606000
C	-0.44261400	-2.81963700	1.29593700
C	-3.20672900	-2.53114400	1.35482000
H	-2.85411700	-0.44633900	1.64586800
C	-1.26011700	-3.93307800	1.16514400
H	0.63066000	-2.95843200	1.34850700
C	-2.64666800	-3.79263000	1.17603200
H	-4.28434600	-2.40900400	1.38737200
H	-0.81186900	-4.91679900	1.06938000
H	-3.28489200	-4.66358400	1.06779400
C	4.14624800	1.70830000	1.44974600
H	5.14591600	1.47486900	1.09024300
H	3.81581200	2.66344100	1.02768400
H	4.14889100	1.79787200	2.53977000
H	1.77054600	-2.05755300	-0.08025200
C	-0.70396000	0.80944600	2.37943700
H	0.02710600	1.60453700	2.52515800
H	-1.57145200	1.25039700	1.86372700
H	-1.06645900	0.45908100	3.35108900
H	1.34012700	-0.48160000	-0.89555500
O	0.62426300	0.12488100	-1.74010000
C	-0.44946900	0.59480100	-1.24864800
C	-0.47691900	1.92014200	-0.66783600
C	-1.71564800	-0.11614500	-1.36865800
C	-1.64375100	2.51491000	-0.26994600
C	0.78716100	2.58607400	-0.53325400
C	-2.88957900	0.46366000	-1.01201100
Cl	-1.62605300	-1.71560800	-1.97684900
C	-2.94816100	1.82544000	-0.41700600
C	-1.65751600	3.81668800	0.32164700
N	1.81342200	3.10548100	-0.41245800
Cl	-4.40011300	-0.32355300	-1.19398700
O	-3.98873700	2.33388600	-0.05222600
N	-1.65076100	4.86474600	0.81255100

IM2

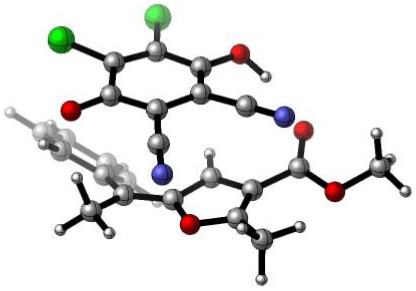

E (M06-2X/6-31G(d)) = -2291.42388955

E (SMD(dichloroethane)/M06-2X /6-311++g(d,p)//M06-2X/6-31G(d)) = -2291.90625143
E (SMD(DMF)/M06-2X /6-311++g(d,p)//M06-2X/6-31G(d)) = -2291.90792012

Zero-point correction=	0.347977 (Hartree/Particle)
Thermal correction to Energy=	0.377980
Thermal correction to Enthalpy=	0.378924
Thermal correction to Gibbs Free Energy=	0.287004

Charge = 0 Multiplicity = 1

C	-2.66791400	-1.59285500	0.47185600
C	-2.43157500	-0.38908000	1.12960800
C	-1.05644100	-0.33942900	1.40311200
O	-1.53429200	-2.27910100	0.38001000
C	-0.51414400	-1.53997700	0.93724000
C	0.79072300	-1.99380600	0.74359200
C	-3.33910200	0.76780800	1.24721400
O	-4.62193200	0.43633400	1.22507800
O	-2.93306900	1.91040200	1.32514800
C	-5.52965600	1.54134700	1.09915300
H	-5.41512900	2.21884400	1.94619500
H	-5.32255200	2.06987700	0.16675400
H	-6.52350500	1.09940100	1.08450400
C	1.90083700	-1.36072000	1.44243600
C	3.17009500	-1.34339300	0.83640600
C	1.74337800	-0.81242800	2.73014000
C	4.24112200	-0.75155400	1.49054400
H	3.28967500	-1.72480000	-0.17507000
C	2.82530600	-0.24143500	3.38255100
H	0.78605500	-0.88939300	3.23520000
C	4.07357100	-0.20383800	2.76103100
H	5.20673900	-0.70574700	0.99824800
H	2.70028600	0.16428100	4.38093500
H	4.91778100	0.24893600	3.27122000
C	-3.84775300	-2.18871400	-0.19167300
H	-4.16378300	-3.09308700	0.33883500
H	-4.66616100	-1.47268600	-0.22038900
H	-3.55383300	-2.47119300	-1.20912300
H	-0.51696500	0.49394200	1.83279100
C	1.05422800	-3.12168500	-0.17413400
H	0.14650500	-3.65006900	-0.46183400
H	1.50164200	-2.69765000	-1.10798900
H	1.79543000	-3.80282900	0.25114100
H	-1.63697600	2.57179100	0.10618800
O	-0.75329600	2.83255600	-0.22172300
C	-0.12123000	1.80136300	-0.80641400
C	-0.75889700	0.75968000	-1.48022300
C	1.30194400	1.81885500	-0.81179900
C	-0.03258200	-0.33993700	-1.99838400
C	-2.18432600	0.80929600	-1.60333800
C	2.02229600	0.81285000	-1.41302300
Cl	2.08039600	3.15711100	-0.04663700
C	1.40143800	-0.38816600	-1.97378700
C	-0.74885700	-1.47493700	-2.47829100
N	-3.34205300	0.85320900	-1.65690100
Cl	3.73671700	0.89148000	-1.51775300

O	2.07573200	-1.36928400	-2.35356300
N	-1.38023900	-2.39726400	-2.79245300

TS2

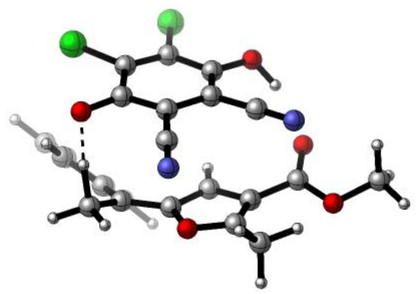

E (M06-2X/6-31G(d)) = -2291.42165140
E (SMD(dichloroethane)/M06-2X /6-311++g(d,p)//M06-2X/6-31G(d)) = -2291.90027905
E (SMD(DMF)/M06-2X /6-311++g(d,p)//M06-2X/6-31G(d)) = -2291.90137627

Zero-point correction=	0.344839 (Hartree/Particle)
Thermal correction to Energy=	0.373948
Thermal correction to Enthalpy=	0.374892
Thermal correction to Gibbs Free Energy=	0.285606

Charge = 0 Multiplicity = 1

C	2.53725400	-1.76998100	-0.00297000
C	2.28750100	-0.83755900	-0.99777400
C	0.89943600	-0.86817700	-1.24751000
O	1.39591500	-2.37432700	0.33212400
C	0.37404900	-1.83688700	-0.40998800
C	-0.94819800	-2.17834900	-0.04031600
C	3.19592700	0.20688400	-1.50191700
O	4.47722600	-0.12201700	-1.41783400
O	2.79616600	1.27648500	-1.92104600
C	5.39715500	0.94832100	-1.67752600
H	5.24943300	1.33279400	-2.68753500
H	5.23762000	1.74298000	-0.94626300
H	6.38747100	0.51186900	-1.56716900
C	-2.05184300	-1.80288800	-0.92319300
C	-3.29499900	-1.46843700	-0.36146100
C	-1.90382300	-1.80617600	-2.32167600
C	-4.35576200	-1.11610700	-1.18460100
H	-3.40174500	-1.42526500	0.71920100
C	-2.97672600	-1.47439100	-3.13652900
H	-0.96148700	-2.11504500	-2.76256400
C	-4.20130600	-1.12266000	-2.56945400

H	-5.30187900	-0.82421600	-0.74093200
H	-2.86109900	-1.49569400	-4.21512200
H	-5.03612300	-0.85449100	-3.20915300
C	3.74022100	-2.15886100	0.76789300
H	4.08242300	-3.15345400	0.46271700
H	4.53758800	-1.43637800	0.60322600
H	3.47483700	-2.19157600	1.82948700
H	0.34979600	-0.21335100	-1.91006000
C	-1.21042400	-2.79026000	1.22796300
H	-0.34167300	-3.21633700	1.72708400
H	-1.53691000	-1.85211600	1.93088500
H	-2.09128300	-3.43222300	1.24117700
H	1.73192000	2.48329500	-0.93297200
O	0.92415100	2.91234300	-0.58556500
C	0.27925700	2.09085300	0.25986100
C	0.92236800	1.24315000	1.16238000
C	-1.13604100	2.14285600	0.29522200
C	0.18919700	0.34635300	1.97306800
C	2.35373500	1.26892000	1.21895500
C	-1.85918000	1.31441800	1.13365900
Cl	-1.91729300	3.26586900	-0.75530600
C	-1.23148500	0.31253700	1.96131500
C	0.88697400	-0.61337500	2.76949700
N	3.51285000	1.28006400	1.22462600
Cl	-3.57397100	1.43067300	1.22221700
O	-1.90844700	-0.56508500	2.59638900
N	1.48612100	-1.40896500	3.36374200

IM3

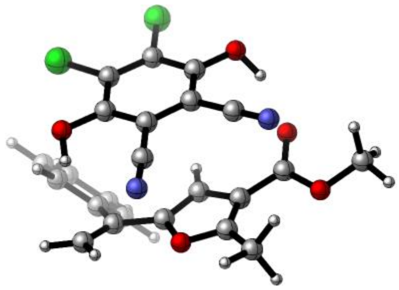

E (M06-2X/6-31G(d)) = -2291.46120245
E (SMD(dichloroethane)/M06-2X /6-311++g(d,p)//M06-2X/6-31G(d)) = -2291.93642533
E (SMD(DMF)/M06-2X /6-311++g(d,p)//M06-2X/6-31G(d)) = -2291.93569874

Zero-point correction=	0.348790 (Hartree/Particle)
Thermal correction to Energy=	0.379136
Thermal correction to Enthalpy=	0.380081
Thermal correction to Gibbs Free Energy=	0.287145

Charge = 0 Multiplicity = 1

C	2.43623400	-1.94200000	0.06670900
C	2.12849800	-1.03532600	-0.91760100
C	0.70839000	-1.07608500	-1.09272600
O	1.30282900	-2.53803500	0.47968800
C	0.24848300	-2.00767500	-0.21271700

C	-1.09131300	-2.40128500	0.21544500
C	3.03437700	-0.04544600	-1.50058400
O	4.31449200	-0.39441500	-1.41731100
O	2.66295800	1.01533400	-1.97804000
C	5.25216600	0.62302400	-1.78658900
H	5.08725200	0.92953400	-2.82064800
H	5.14072600	1.48128800	-1.12099700
H	6.23408200	0.16950400	-1.66756000
C	-2.21806500	-2.01671600	-0.67435200
C	-3.38931000	-1.47777100	-0.13347500
C	-2.13465400	-2.20697400	-2.05957100
C	-4.45787600	-1.14213100	-0.95943700
H	-3.44625900	-1.29294800	0.93544500
C	-3.20583100	-1.87605900	-2.88188100
H	-1.23163600	-2.63253300	-2.48741100
C	-4.37027800	-1.34160500	-2.33411700
H	-5.35503400	-0.71077300	-0.52613300
H	-3.13181700	-2.03753300	-3.95279100
H	-5.20305200	-1.07653700	-2.97791800
C	3.69307500	-2.32949400	0.75844000
H	4.28466500	-3.01717100	0.14633200
H	4.29933500	-1.44028100	0.94671400
H	3.44677100	-2.81092800	1.70658200
H	0.11976100	-0.46039800	-1.75821100
C	-1.27956300	-3.04071000	1.38237300
H	-0.44357900	-3.29900900	2.02531600
H	-1.45253900	-0.89776700	2.83375000
H	-2.27426100	-3.35491200	1.68189500
H	1.91980000	2.45673300	-1.17016300
O	1.22801200	3.01195200	-0.74679300
C	0.50996100	2.26011700	0.09696400
C	1.09002300	1.37934400	1.01105200
C	-0.89482800	2.40200200	0.10772400
C	0.28499000	0.55985700	1.82997200
C	2.51838800	1.30581700	1.10158200
C	-1.68844800	1.63147200	0.95227300
Cl	-1.59604100	3.54226600	-0.96747400
C	-1.10573200	0.66343000	1.79223600
C	0.86319600	-0.42553000	2.69305500
N	3.67351300	1.25294600	1.16782900
Cl	-3.39555500	1.82469600	0.99445500
O	-1.92040600	-0.09201000	2.54922200
N	1.26485100	-1.25705400	3.39279300

2

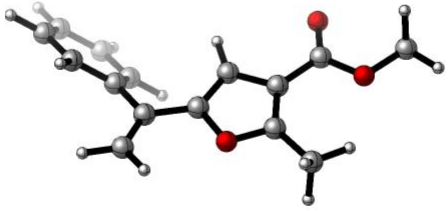

E (M06-2X/6-31G(d)) = -805.349760940

E (SMD(dichloroethane)/M06-2X /6-311++g(d,p)//M06-2X/6-31G(d)) = -805.588869413
E (SMD(DMF)/M06-2X /6-311++g(d,p)//M06-2X/6-31G(d)) = -805.587884154

Zero-point correction=	0.259049 (Hartree/Particle)
Thermal correction to Energy=	0.275572
Thermal correction to Enthalpy=	0.276516
Thermal correction to Gibbs Free Energy=	0.213215

Charge = 0 Multiplicity = 1

C	1.96166100	1.32054900	0.15586600
C	1.82442000	-0.02635500	-0.05601700
C	0.41813700	-0.28621900	-0.14727100
O	0.73784000	1.88813600	0.19931700
C	-0.20475600	0.91106300	0.01440000
C	-1.60486500	1.32896600	-0.01483000
C	2.84435600	-1.07786000	-0.17779700
O	4.10509900	-0.61047500	-0.07515600
O	2.58943600	-2.24580800	-0.35291000
C	5.11745600	-1.60901000	-0.19024900
H	5.00420400	-2.35878800	0.59559300
H	5.05527600	-2.10396500	-1.16166100
H	6.06522700	-1.08292700	-0.08648100
C	-2.63403800	0.25484200	0.01778900
C	-3.73260100	0.31109300	-0.84598600
C	-2.54463200	-0.80657300	0.92598800
C	-4.72309700	-0.66459700	-0.79883800
H	-3.79369500	1.11964900	-1.56869800
C	-3.53544000	-1.78177700	0.97313100
H	-1.70107800	-0.85569900	1.60812500
C	-4.62717800	-1.71437200	0.11089700
H	-5.56619800	-0.60960700	-1.48066800
H	-3.45576500	-2.59471800	1.68824200
H	-5.39747300	-2.47849000	0.14545700
C	3.10980200	2.24501000	0.34749900
H	4.04564900	1.69179500	0.30458700
H	3.10885400	3.01513500	-0.43027100
H	3.03070300	2.74970600	1.31548200
H	-0.03885500	-1.24809400	-0.32546800
C	-1.94274100	2.62420000	-0.07842800
H	-1.18989200	3.40287600	-0.12639700
H	-2.98485300	2.92454600	-0.06476600

DDQH$_2$

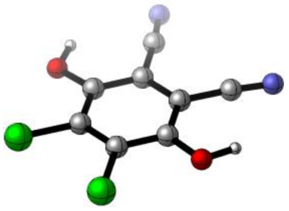

E (M06-2X/6-31G(d)) = -1486.07792416
E (SMD(dichloroethane)/M06-2X /6-311++g(d,p)//M06-2X/6-31G(d)) = -1486.32365032
E (SMD(DMF)/M06-2X /6-311++g(d,p)//M06-2X/6-31G(d)) = -1486.32273189

Zero-point correction= 0.088494 (Hartree/Particle)
Thermal correction to Energy= 0.101046
Thermal correction to Enthalpy= 0.101990
Thermal correction to Gibbs Free Energy= 0.049048

Charge = 0 Multiplicity = 1

O	0.06480100	2.75335200	0.00006900
C	0.13195200	1.41418100	0.00001400
C	1.33143400	0.70512900	-0.00011100
C	-1.08122800	0.69510000	-0.00008300
C	1.33132500	-0.70558200	0.00005600
C	2.54576600	1.46219200	0.00016300
C	-1.08139200	-0.69493200	-0.00003700
Cl	-2.54742400	1.58627400	-0.00030300
C	0.13163600	-1.41403000	-0.00001400
C	2.54570900	-1.46251200	-0.00010400
N	3.46703700	2.16411000	0.00027400
Cl	-2.54768700	-1.58585400	0.00020300
O	0.06399900	-2.75384800	0.00057700
N	3.46719200	-2.16414000	-0.00045700
H	0.95748500	-3.13814700	-0.00267200
H	0.95818300	3.13790100	0.00118300

TS3

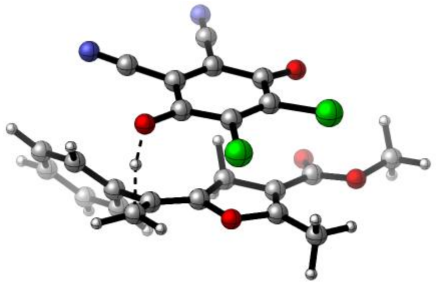

E (M06-2X/6-31G(d)) = -2291.35963173
E (SMD(dichloroethane)/M06-2X /6-311++g(d,p)//M06-2X/6-31G(d)) = -2291.84086751
E (SMD(DMF)/M06-2X /6-311++g(d,p)//M06-2X/6-31G(d)) = -2291.84324940

Zero-point correction= 0.342130 (Hartree/Particle)
Thermal correction to Energy= 0.371609
Thermal correction to Enthalpy= 0.372553
Thermal correction to Gibbs Free Energy= 0.282141

Charge = 0 Multiplicity = 1

C	-1.76978300	-0.00147300	-1.89290000
C	-1.54718000	-1.13844600	-1.21886700
C	-0.08392700	-1.24338700	-0.91863700
O	-0.57893400	0.69072800	-2.09211300
C	0.44118000	0.00857000	-1.53238200
C	1.74305100	0.50923400	-1.65002600

C	-2.47573400	-2.16623000	-0.71780700
O	-3.76446000	-1.82206500	-0.83594000
O	-2.08623200	-3.20084000	-0.23314600
C	-4.67392300	-2.72826800	-0.20351700
H	-4.59151300	-3.72192300	-0.64769900
H	-4.44767600	-2.78611800	0.86303200
H	-5.66560000	-2.31086900	-0.36794800
C	2.90698000	-0.33934400	-1.28708600
C	3.95869000	0.20159300	-0.54146900
C	3.01174800	-1.65716900	-1.75079700
C	5.07160100	-0.57121700	-0.22723700
H	3.88824200	1.21904500	-0.16792500
C	4.13045100	-2.42339100	-1.44826500
H	2.23505700	-2.07123200	-2.38705000
C	5.15912600	-1.88463900	-0.67751400
H	5.85847700	-0.14659300	0.38717400
H	4.20115500	-3.44088300	-1.81889400
H	6.02608300	-2.48852000	-0.42991700
C	-2.95646100	0.65639100	-2.48855400
H	-3.85340500	0.09243500	-2.23924300
H	-3.04462500	1.67595400	-2.10252600
H	-2.83932100	0.71098200	-3.57576100
H	0.36790800	-2.16599600	-1.29574500
C	1.94193400	1.85363100	-2.00661800
H	1.11177200	2.41152400	-2.43417500
H	1.85038500	2.39768500	-0.68408000
H	2.94072600	2.16237900	-2.30326400
H	0.12034400	-1.26362800	0.17149100
O	1.57801100	2.61725700	0.42542500
C	0.70793100	1.76495900	0.85874900
C	1.08062500	0.69478800	1.73561300
C	-0.71117300	1.96641900	0.60910400
C	0.14790300	-0.19722800	2.22095400
C	2.44827400	0.62334300	2.16813500
C	-1.65365900	1.12766500	1.11131800
Cl	-1.12235300	3.32316200	-0.36658600
C	-1.28570400	-0.08614700	1.87549000
C	0.51922500	-1.26006600	3.10334000
N	3.54020200	0.58004800	2.54652300
Cl	-3.33379400	1.34874800	0.84909300
O	-2.10505300	-0.93310300	2.18245400
N	0.82876900	-2.11862800	3.81494700

TS4

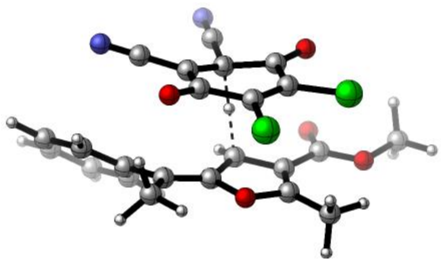

E (M06-2X/6-31G(d)) = -2291.36810968

E (SMD(dichloroethane)/M06-2X /6-311++g(d,p)//M06-2X/6-31G(d)) = -2291.84789059
E (SMD(DMF)/M06-2X /6-311++g(d,p)//M06-2X/6-31G(d)) = -2291.85080408

Zero-point correction=	0.342963 (Hartree/Particle)
Thermal correction to Energy=	0.373046
Thermal correction to Enthalpy=	0.373990
Thermal correction to Gibbs Free Energy=	0.280260

Charge = 0 Multiplicity = 1

C	-1.67592400	-0.34171600	-1.82760600
C	-1.43152300	-1.41983700	-1.02691100
C	-0.02324300	-1.39063400	-0.69301200
O	-0.53504100	0.34665300	-2.05982400
C	0.51822600	-0.29491000	-1.43512500
C	1.77456200	0.28034100	-1.48351900
C	-2.33969900	-2.40754200	-0.41740600
O	-3.62871400	-2.19559900	-0.71279200
O	-1.94017700	-3.29571500	0.29525500
C	-4.54026700	-3.06105500	-0.02738700
H	-4.33966700	-4.10250400	-0.28489700
H	-4.43394400	-2.92792500	1.05103900
H	-5.53306200	-2.76357500	-0.35948200
C	2.97435300	-0.45886500	-1.07254700
C	4.10126200	0.24753400	-0.62239600
C	3.06475800	-1.85789900	-1.19664200
C	5.26139900	-0.42644800	-0.25940400
H	4.05545800	1.32625700	-0.51646900
C	4.22769200	-2.52460600	-0.84430200
H	2.24210600	-2.41595600	-1.63090400
C	5.32661300	-1.81129100	-0.36346200
H	6.10701400	0.13630600	0.12087400
H	4.28408300	-3.60222200	-0.95820600
H	6.23282300	-2.33671200	-0.07983700
C	-2.88452600	0.19844500	-2.49633200
H	-3.77050100	-0.33625100	-2.16042200
H	-2.98381300	1.26359000	-2.26704000
H	-2.77801400	0.09408900	-3.58120700
H	0.51841400	-2.27371700	-0.37221000
C	1.94567800	1.63087600	-2.10292100
H	1.03853300	1.96559200	-2.60608500
H	2.17351000	2.37349100	-1.32114500
H	2.78143200	1.62060700	-2.80740000
H	0.07577600	-0.80598000	0.61031400
O	1.26132800	3.11743200	0.44303000
C	0.55600500	2.16970000	0.75469800
C	1.03309900	0.99302200	1.44337100
C	-0.92145700	2.19579500	0.42629300
C	0.16255800	-0.10586200	1.72305100
C	2.38508200	0.99099200	1.87001500
C	-1.79356400	1.30749800	0.93861300
Cl	-1.41622900	3.47877000	-0.59210600
C	-1.33348700	0.14316700	1.75078300
C	0.60778100	-1.10758000	2.67272800
N	3.49371600	0.99705700	2.21240600
Cl	-3.49054600	1.39009600	0.69041600

O	-2.10035300	-0.62412900	2.27423500
N	0.98005800	-1.92130400	3.40317900

TS5

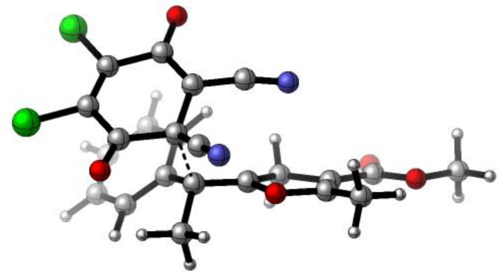

E (M06-2X/6-31G(d)) = -2291.36284884
E (SMD(dichloroethane)/M06-2X /6-311++g(d,p)//M06-2X/6-31G(d)) = -2291.84945796
E (SMD(DMF)/M06-2X /6-311++g(d,p)//M06-2X/6-31G(d)) = -2291.85312539

Zero-point correction=	0.347806 (Hartree/Particle)
Thermal correction to Energy=	0.377130
Thermal correction to Enthalpy=	0.378075
Thermal correction to Gibbs Free Energy=	0.287660

Charge = 0 Multiplicity = 1

C	-3.50793800	1.04649300	-0.26624900
C	-3.58941100	-0.28479800	-0.36072600
C	-2.23679400	-0.83153100	-0.68303700
O	-2.18910900	1.44329500	-0.56378200
C	-1.41387200	0.41175700	-0.72302400
C	-0.00992000	0.66195400	-1.06567200
C	-4.73160000	-1.18786800	-0.12558500
O	-5.85549200	-0.53981500	0.19698300
O	-4.63659400	-2.38656800	-0.22740100
C	-6.97953500	-1.38752300	0.45988300
H	-7.21754900	-1.98106600	-0.42471200
H	-6.75663400	-2.05606000	1.29317600
H	-7.79943800	-0.71729800	0.70959800
C	0.77351000	-0.59138100	-1.39825600
C	1.67264900	-0.59345800	-2.47164700
C	0.74110000	-1.71580300	-0.56059800
C	2.51823300	-1.67678700	-2.69225600
H	1.74068500	0.26052600	-3.13548100
C	1.59138300	-2.79549900	-0.77877800
H	0.10038700	-1.74118600	0.31279700

C	2.48725500	-2.77826800	-1.84319500
H	3.21476600	-1.64683800	-3.52380700
H	1.56326600	-3.63808100	-0.09560300
H	3.16032700	-3.61375100	-2.00527600
C	-4.38339100	2.17064800	0.12428200
H	-5.38903700	1.80491000	0.32056000
H	-3.96942600	2.62790100	1.02941500
H	-4.40460400	2.92989500	-0.66260600
H	-2.17040700	-1.33531400	-1.66064900
C	0.00012000	1.74150000	-2.17233500
H	-0.58874000	2.60691300	-1.86980800
H	1.01528500	2.08281300	-2.36757900
H	-0.41854200	1.32507000	-3.09384500
H	-1.90651500	-1.55770100	0.06160700
O	2.49738900	2.54580100	-0.77604500
C	2.22391600	1.59457400	-0.08372400
C	0.77130700	1.35750700	0.37662200
C	3.24439100	0.59924600	0.32406100
C	0.64701100	0.49961600	1.55811200
C	0.11211900	2.65656500	0.55845700
C	2.99629000	-0.30471900	1.28866700
Cl	4.75636000	0.73340000	-0.48746900
C	1.65365500	-0.41214400	1.99660900
C	-0.67490600	0.32521900	2.00378300
N	-0.45241500	3.65240200	0.71770800
Cl	4.17220100	-1.43899200	1.78925200
O	1.50323300	-1.26623800	2.85738600
N	-1.82590500	0.23912900	2.18120800

TS6

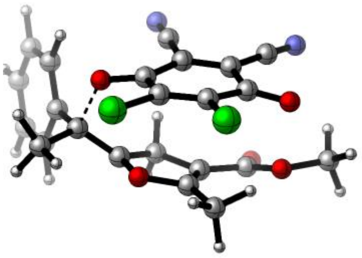

E (M06-2X/6-31G(d)) = -2291.35748489
E (SMD(dichloroethane)/M06-2X /6-311++g(d,p)//M06-2X/6-31G(d)) = -2291.83633762
E (SMD(DMF)/M06-2X /6-311++g(d,p)//M06-2X/6-31G(d)) = -2291.83886553

Zero-point correction=	0.346000 (Hartree/Particle)
Thermal correction to Energy=	0.375637
Thermal correction to Enthalpy=	0.376581
Thermal correction to Gibbs Free Energy=	0.285287

Charge = 0 Multiplicity = 1

C	-0.93564300	-0.09379400	-1.84574300
C	-0.50388800	-1.30456100	-1.46386000
C	0.95810100	-1.22424300	-1.15348000
O	0.13227600	0.82166400	-1.74940700

C	1.21627600	0.22702500	-1.30994000
C	2.17493100	1.04792800	-0.62412800
C	-1.28206300	-2.55034600	-1.26587300
O	-2.58415000	-2.29326100	-1.17933900
O	-0.76000700	-3.63077700	-1.14846100
C	-3.42719800	-3.40075700	-0.82737300
H	-3.53935200	-4.06485100	-1.68731200
H	-2.99271600	-3.94258800	0.01314200
H	-4.37666200	-2.95134500	-0.54459300
C	3.42238600	0.30340600	-0.22095000
C	3.87224200	0.25100400	1.09715200
C	4.16977600	-0.31151100	-1.23153700
C	5.04967300	-0.42922400	1.39857300
H	3.29396200	0.71846600	1.88393000
C	5.34538200	-0.98775100	-0.92581900
H	3.83195700	-0.25517300	-2.26512700
C	5.78594500	-1.04903000	0.39416900
H	5.38349100	-0.48102200	2.42959100
H	5.91399100	-1.46610000	-1.71675300
H	6.69960600	-1.58145800	0.63796300
C	-2.21831800	0.48459700	-2.31943100
H	-3.04785900	0.05917200	-1.74883600
H	-2.20514200	1.56869000	-2.18798000
H	-2.36978600	0.25958500	-3.37993500
H	1.56707300	-1.83160700	-1.84204600
C	2.43342000	2.43258700	-1.17842600
H	1.52226800	2.89262200	-1.56055000
H	2.83823300	3.04902100	-0.37259100
H	3.17946500	2.36704400	-1.97604900
H	1.24476200	-1.57573600	-0.15167000
O	1.34397000	1.38784600	0.80689300
C	0.07675300	1.09876800	1.03011600
C	-0.27737900	-0.16395500	1.57241100
C	-0.99608700	1.97320800	0.64337400
C	-1.58108000	-0.62848900	1.54365800
C	0.77834700	-0.98149700	2.10863700
C	-2.30110700	1.56214900	0.67060300
Cl	-0.57289300	3.56887800	0.13190100
C	-2.67657400	0.17474700	0.98569800
C	-1.90971400	-1.94776900	1.98050800
N	1.63836400	-1.63213800	2.52922200
Cl	-3.59872600	2.60239300	0.22620300
O	-3.80115800	-0.27499000	0.80492000
N	-2.16955100	-3.03288000	2.29280200

Copies of ^{1}H and $^{13}C\{^{1}H\}$ NMR spectra of isolated compounds

^{1}H NMR (400 MHz, $CDCl_3$) of compound **2**

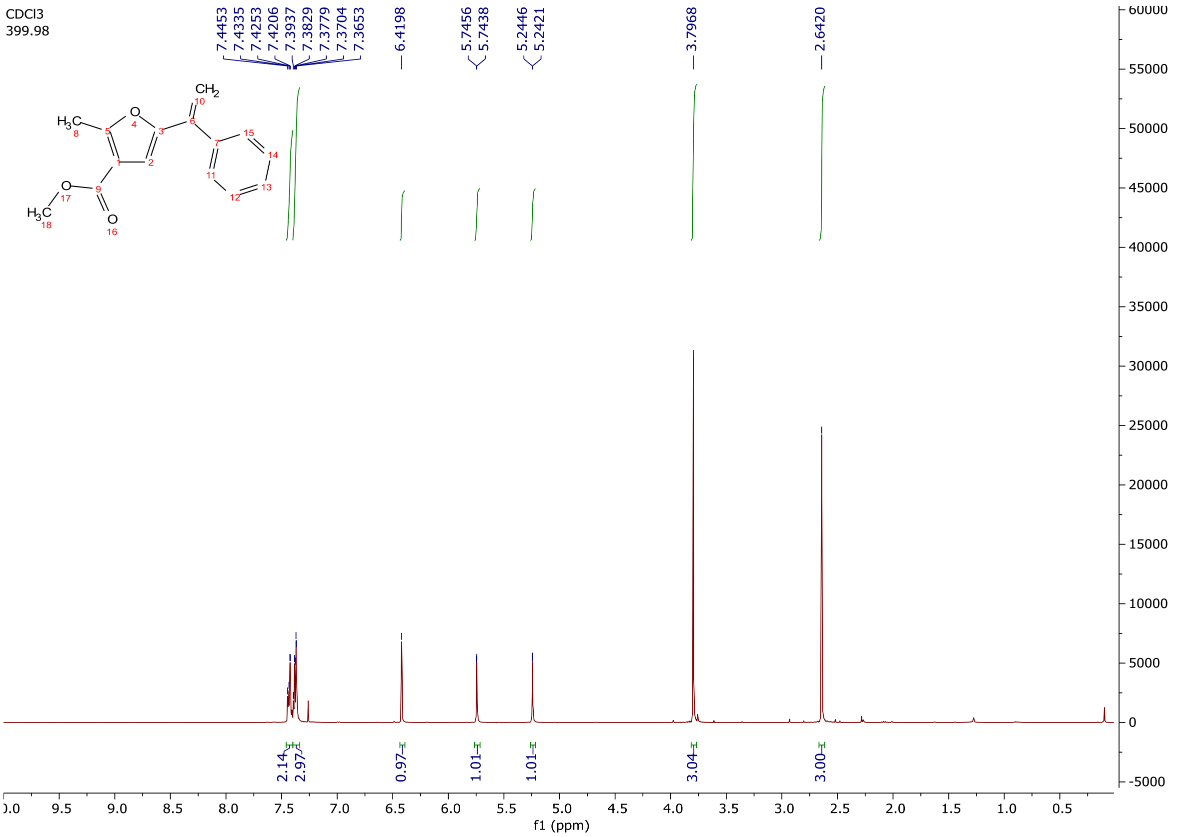

$^{13}C\{^{1}H\}$ NMR (101 MHz, $CDCl_3$) of compound **2**

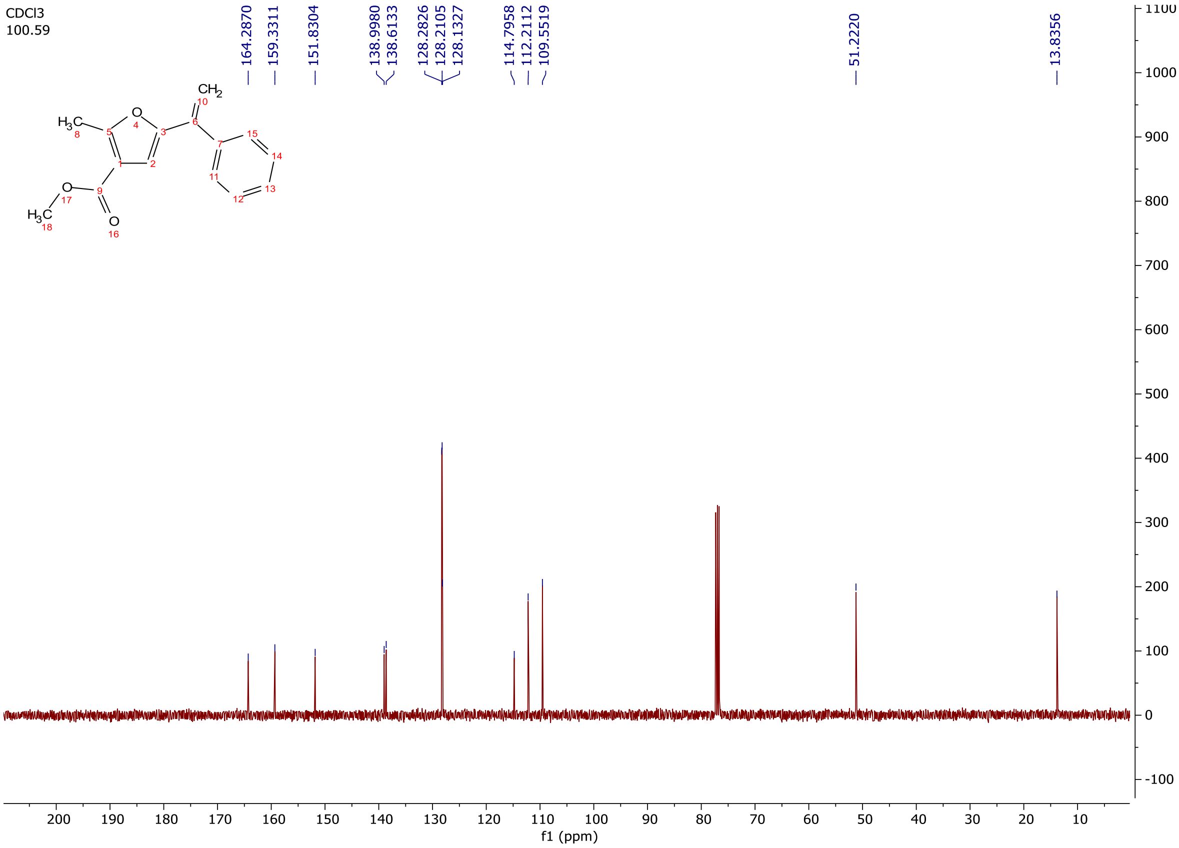

^{1}H NMR (400 MHz, $CDCl_3$) of compound **3**

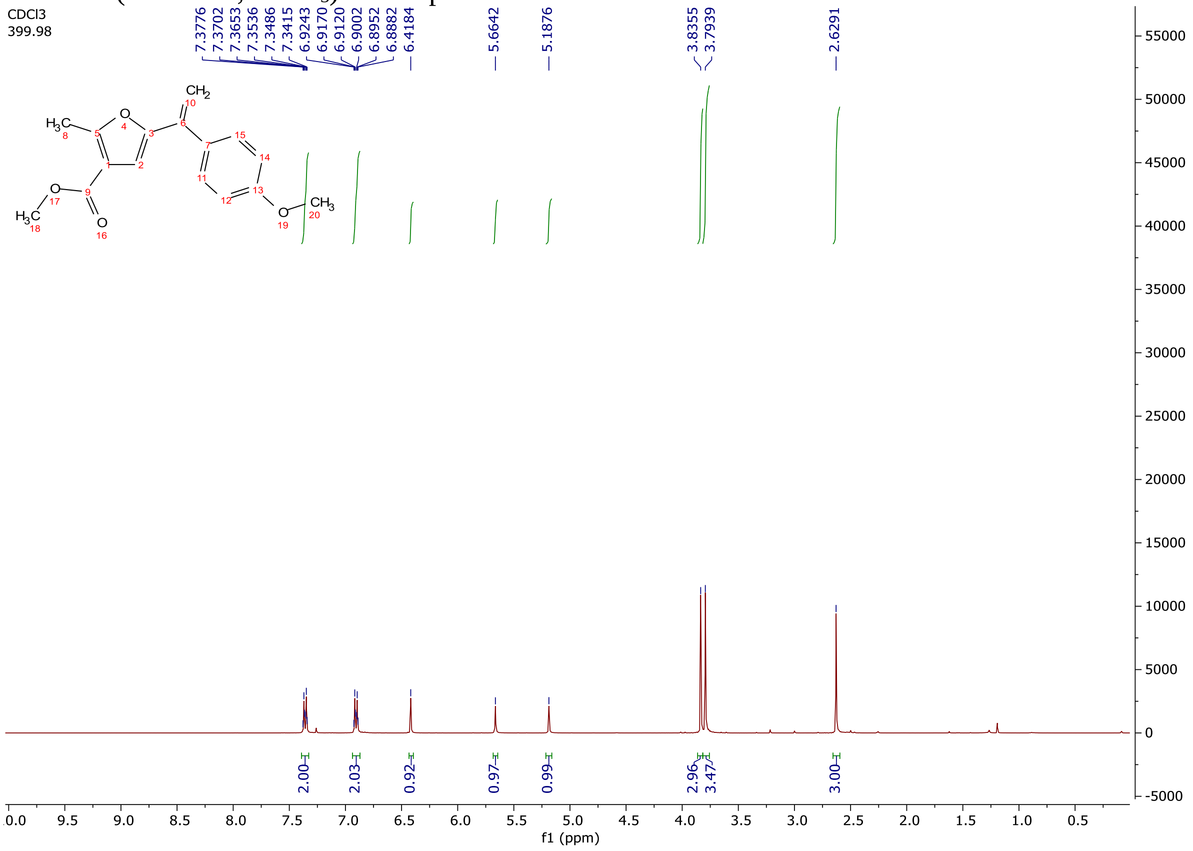

$^{13}C\{^{1}H\}$ NMR (101 MHz, $CDCl_3$) of compound **3**

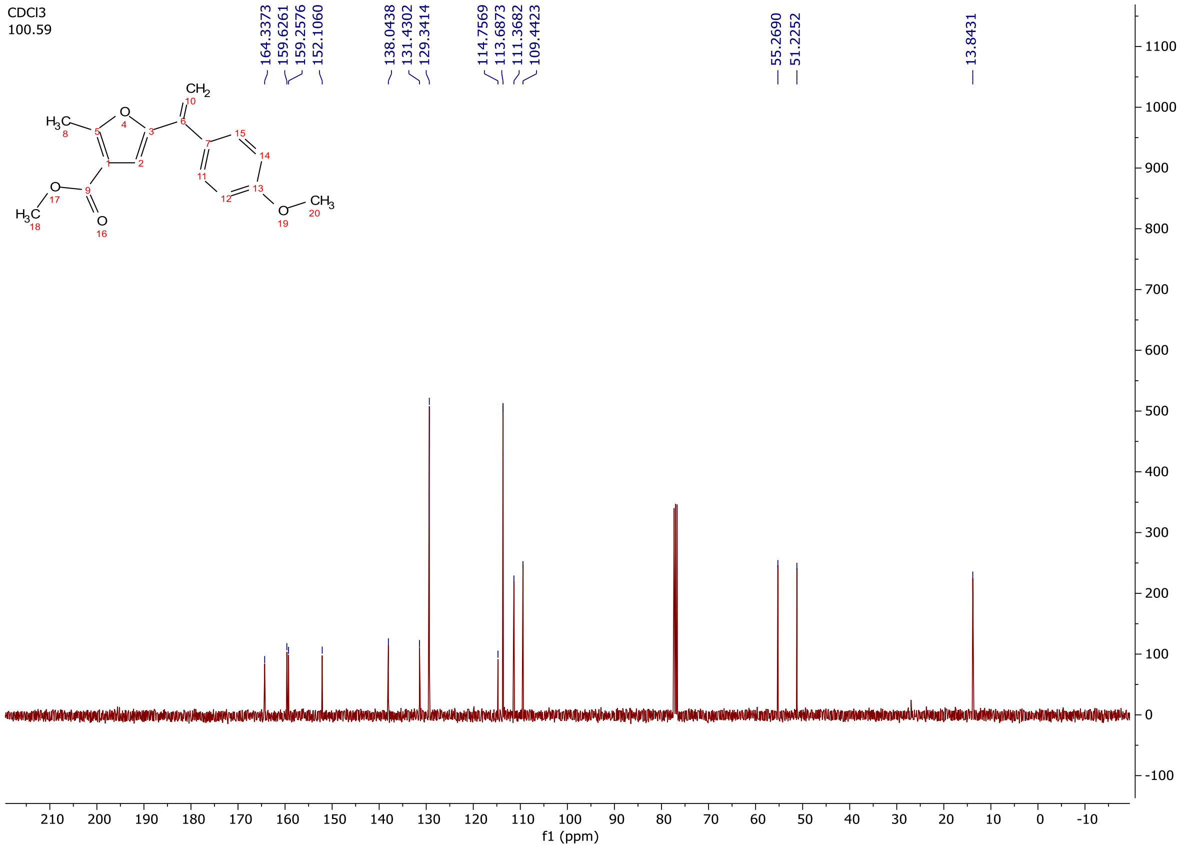

^{1}H NMR (500 MHz, $CDCl_3$) of compound **4**

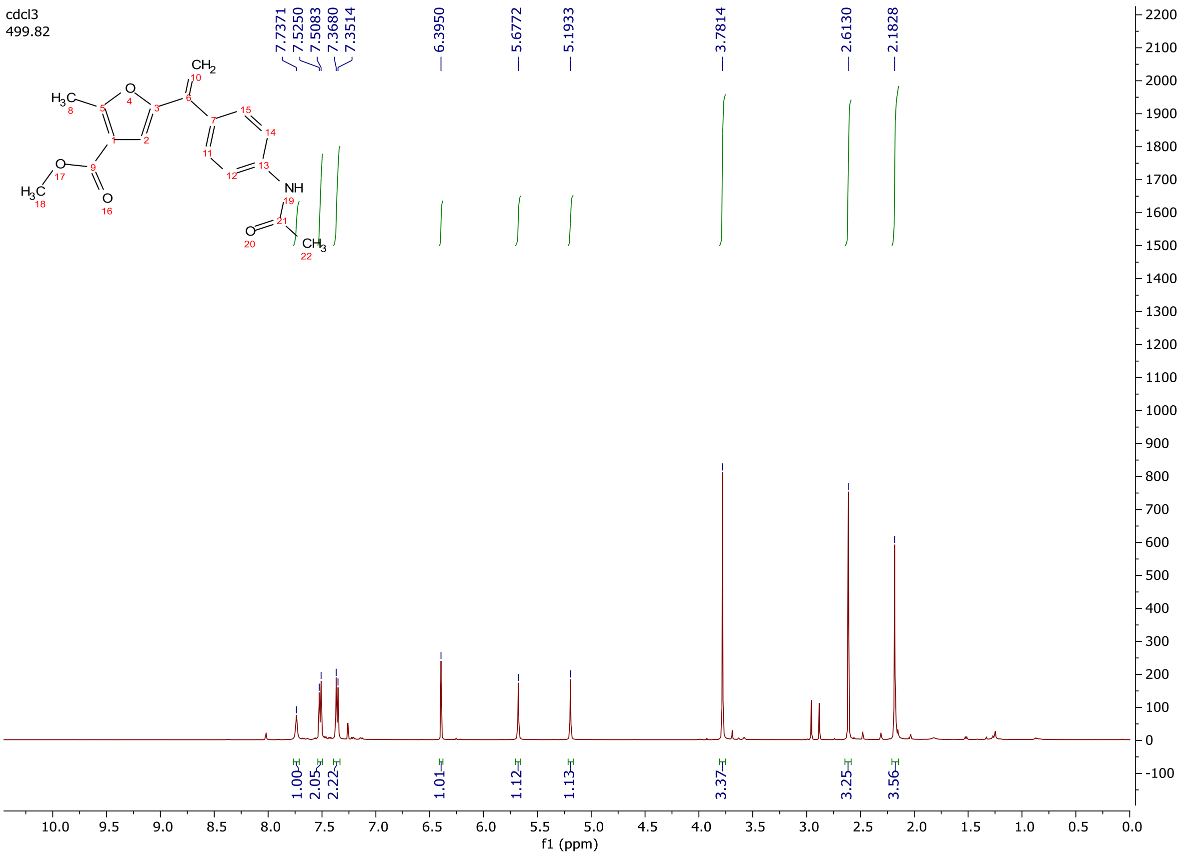

$^{13}C\{^{1}H\}$ NMR (126 MHz, $CDCl_3$) of compound **4**

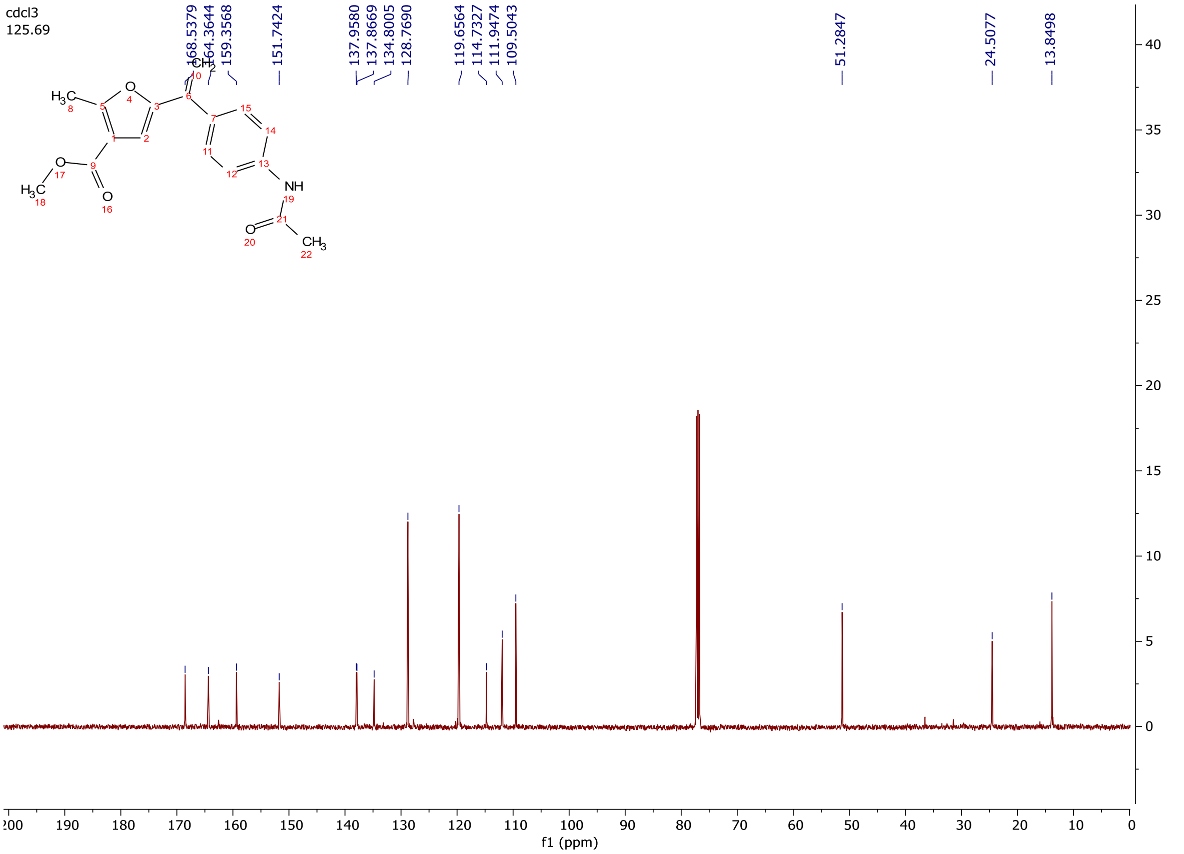

^{1}H NMR (400 MHz, $CDCl_3$) of compound **5**

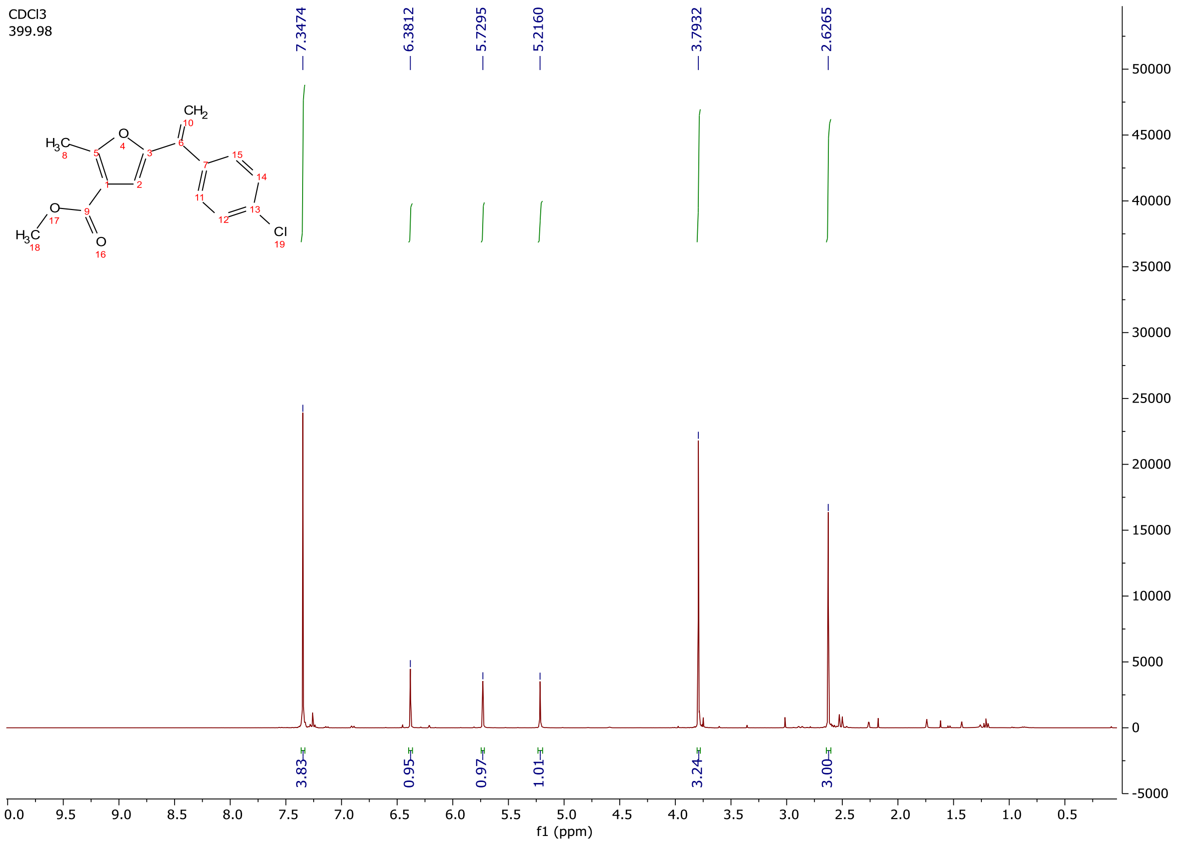

$^{13}C\{^{1}H\}$ NMR (101 MHz, $CDCl_3$) of compound **5**

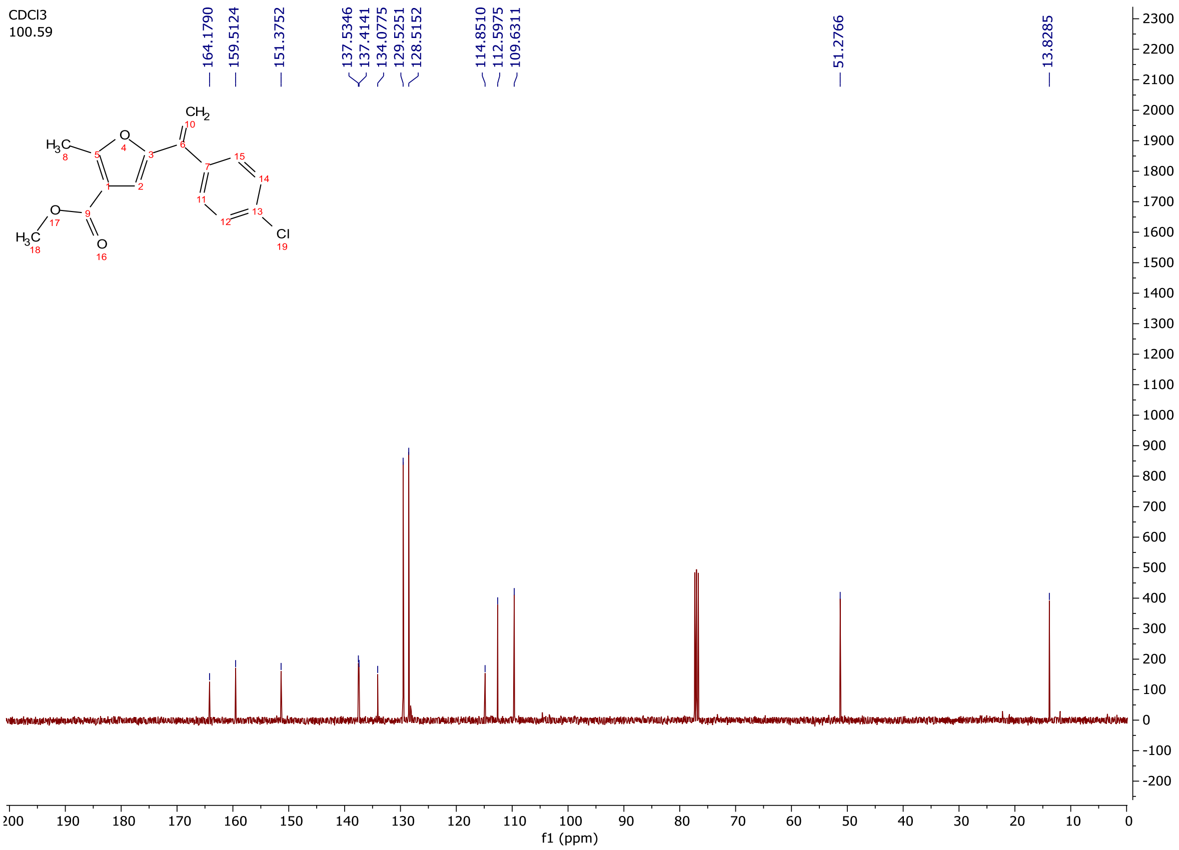

^{1}H NMR (400 MHz, $CDCl_3$) of compound **6**

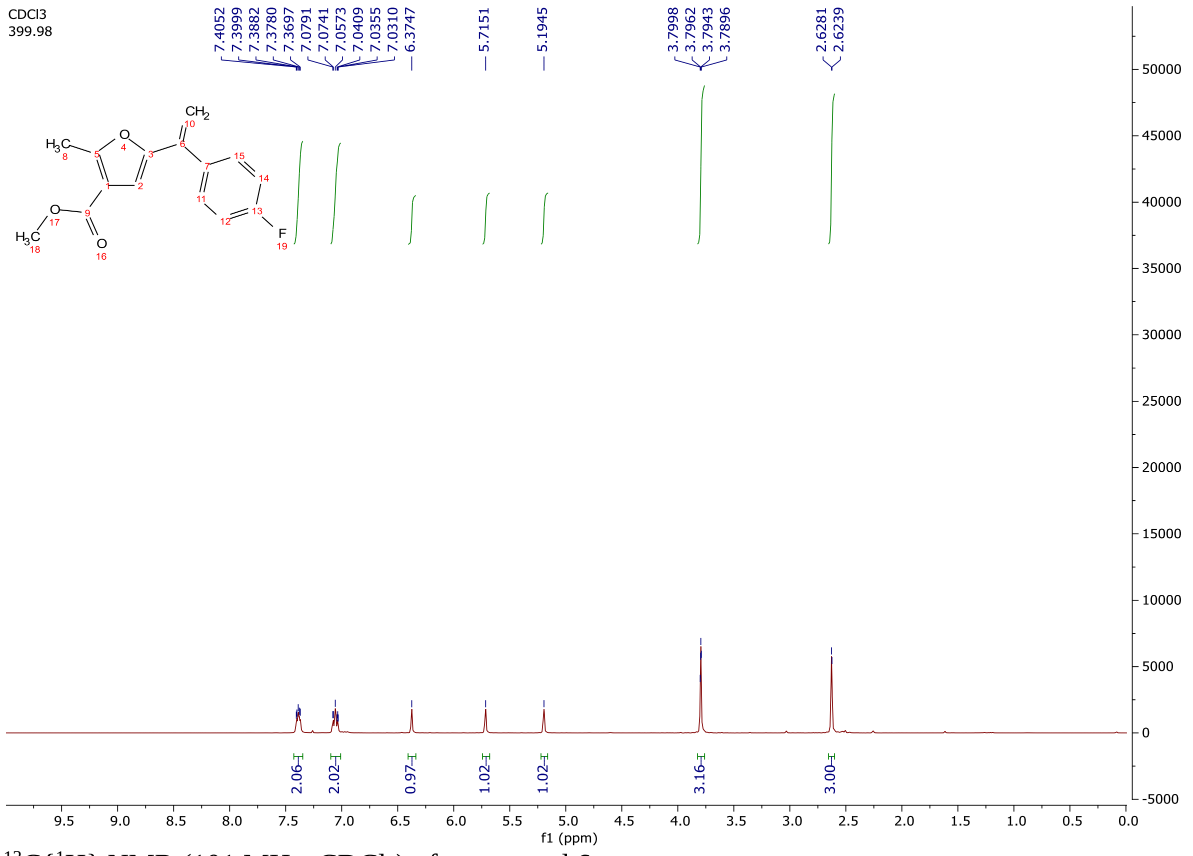

$^{13}C\{^{1}H\}$ NMR (101 MHz, $CDCl_3$) of compound **6**

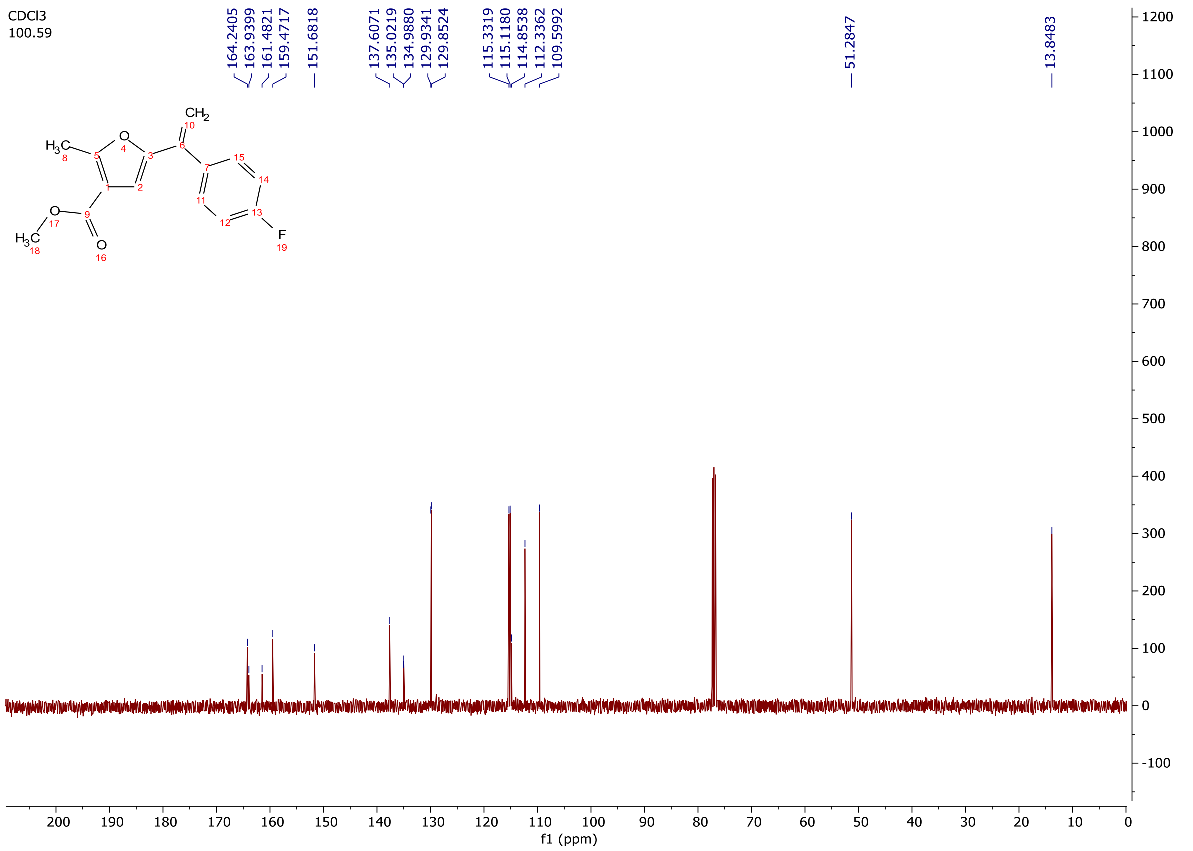

^{1}H NMR (500 MHz, $CDCl_3$) of compound **7**

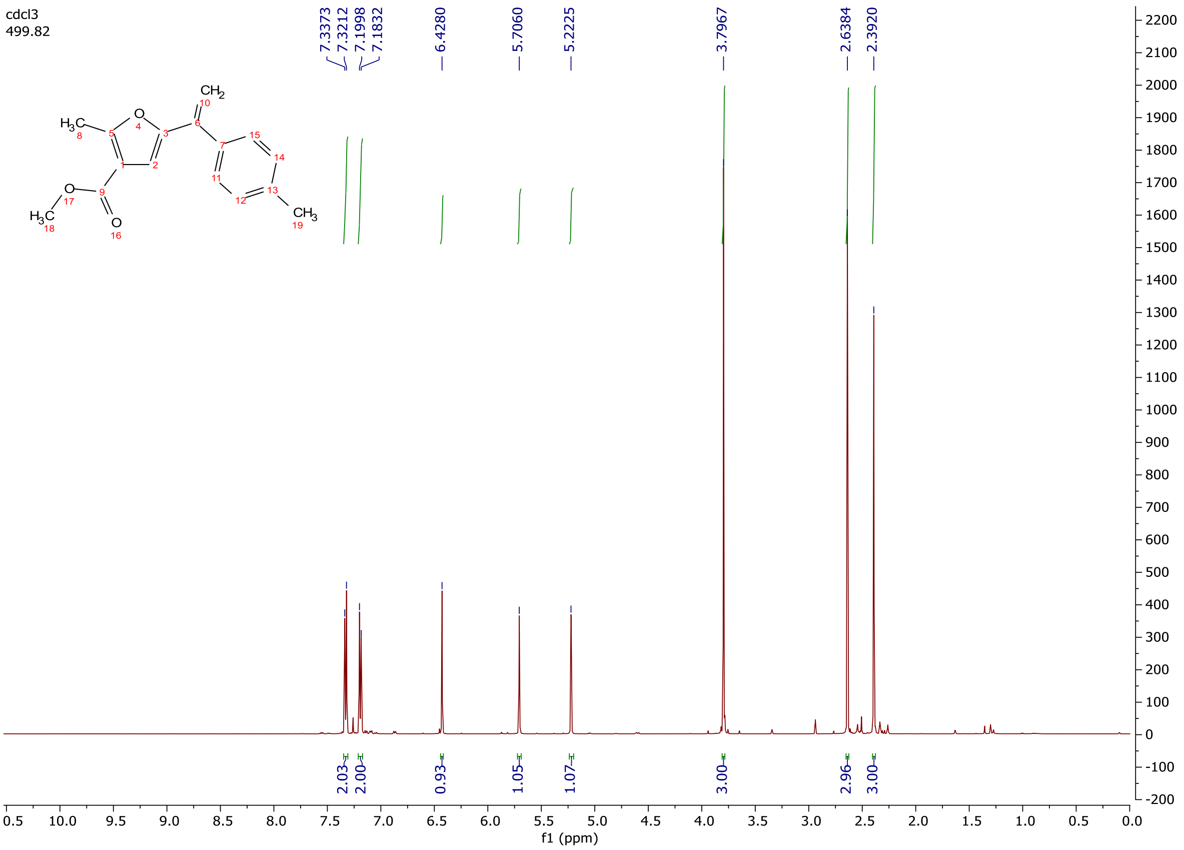

$^{13}C\{^1H\}$ NMR (126 MHz, $CDCl_3$) of compound **7**

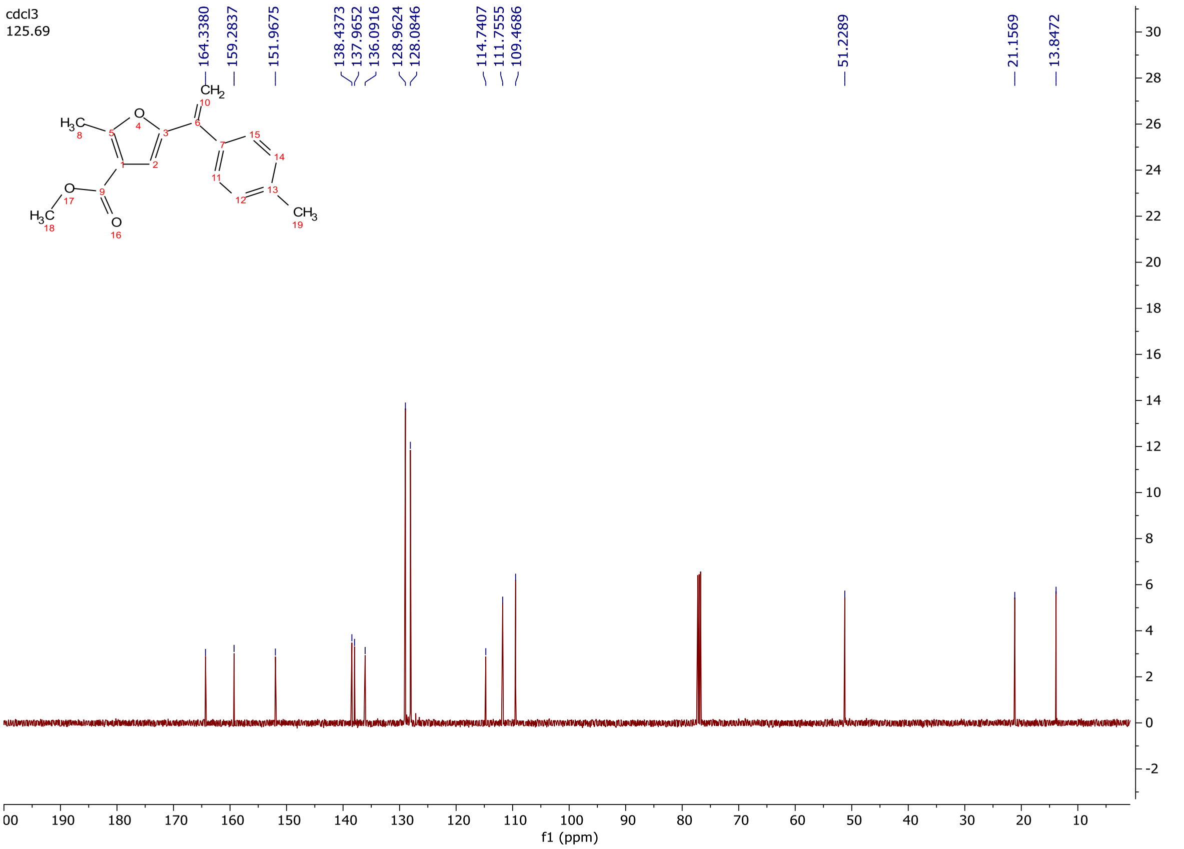

^{1}H NMR (500 MHz, $CDCl_3$) of compound **8**

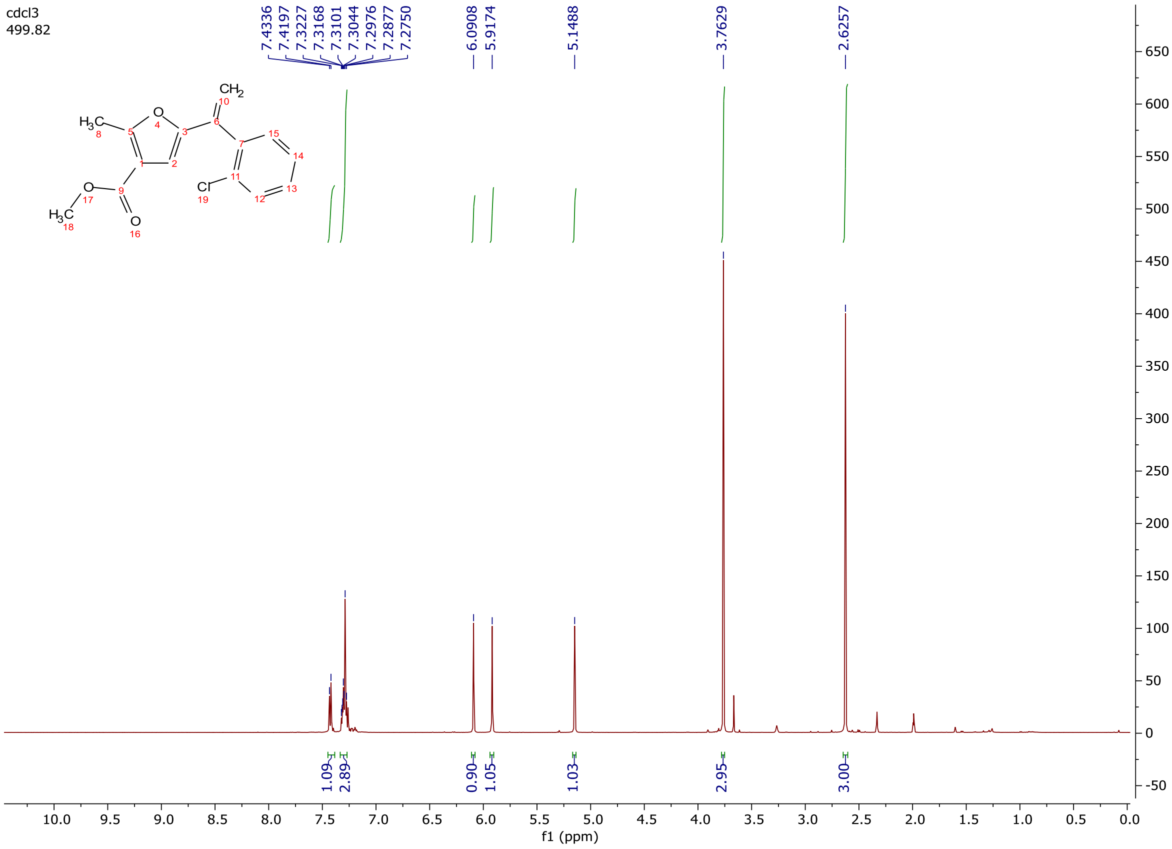

$^{13}C\{^{1}H\}$ NMR (126 MHz, $CDCl_3$) of compound **8**

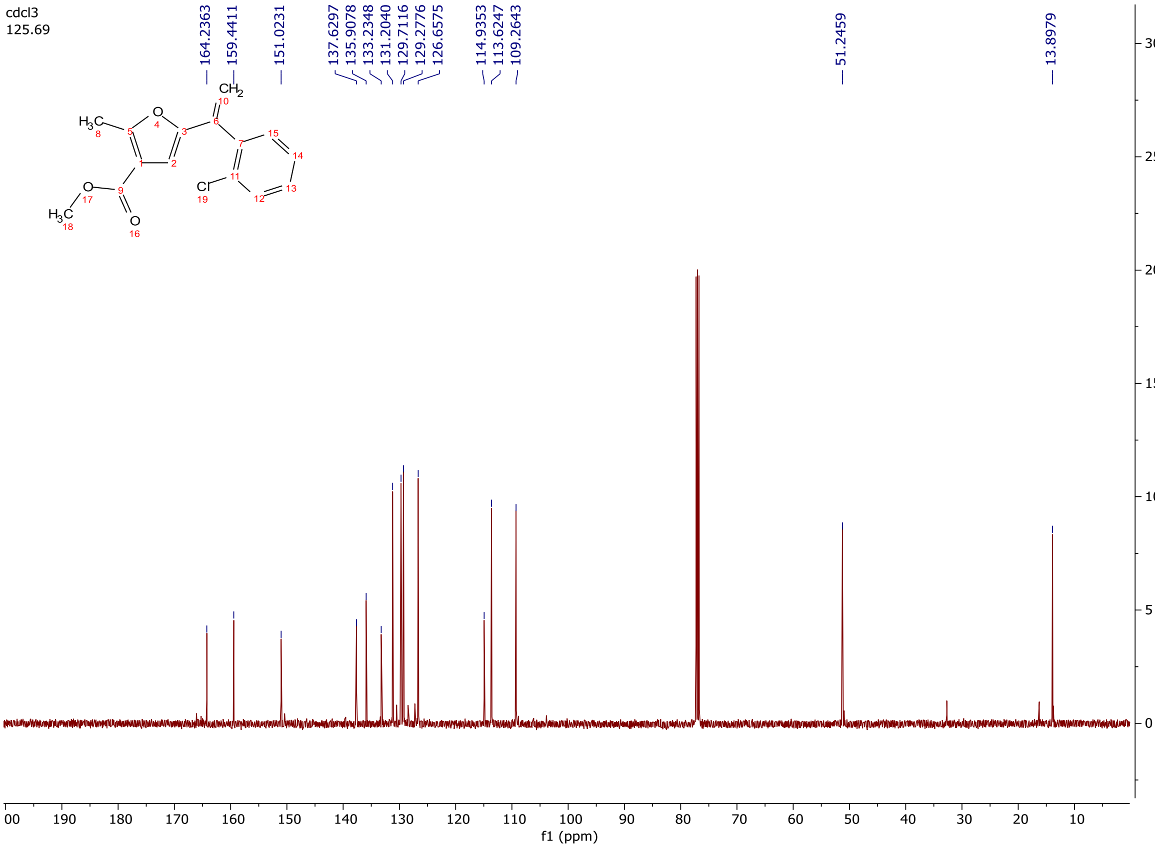

^{1}H NMR (400 MHz, $CDCl_3$) of compound **9**

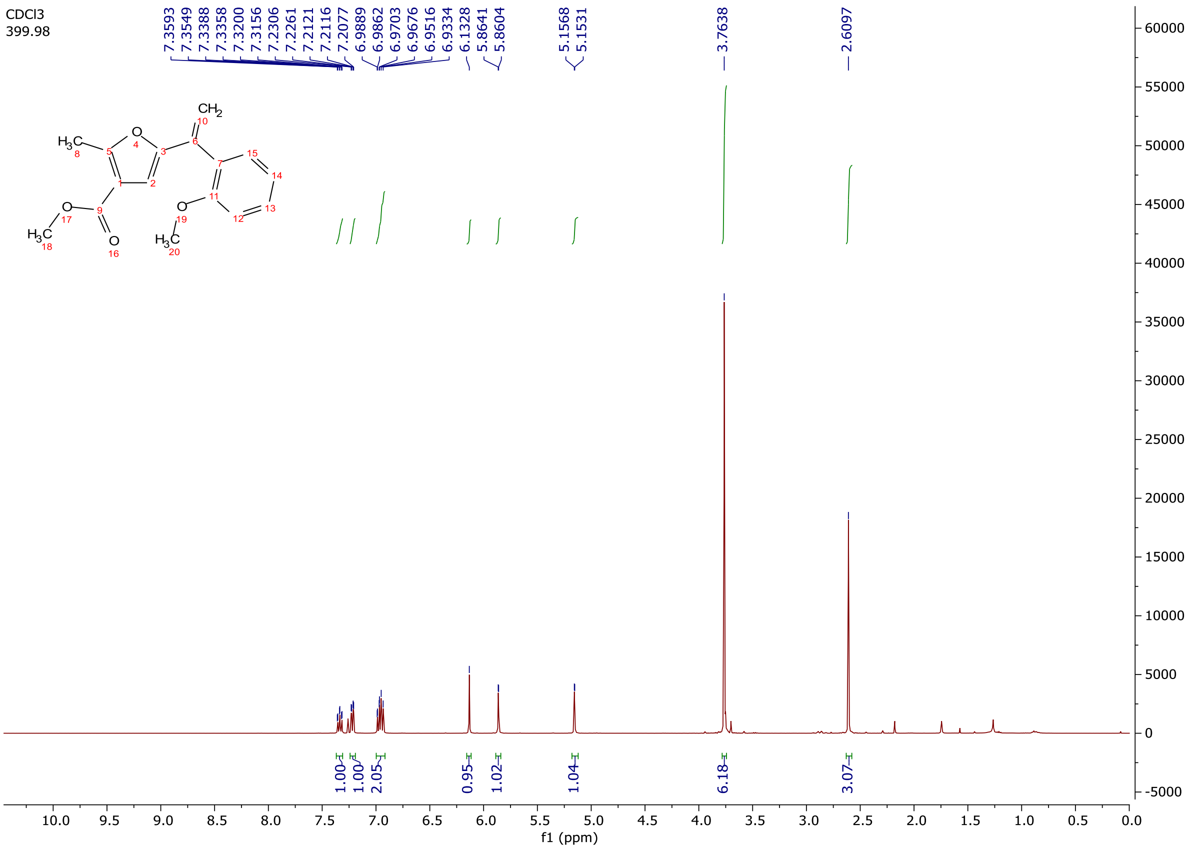

^{13}C{^{1}H} NMR (101 MHz, $CDCl_3$) of compound **9**

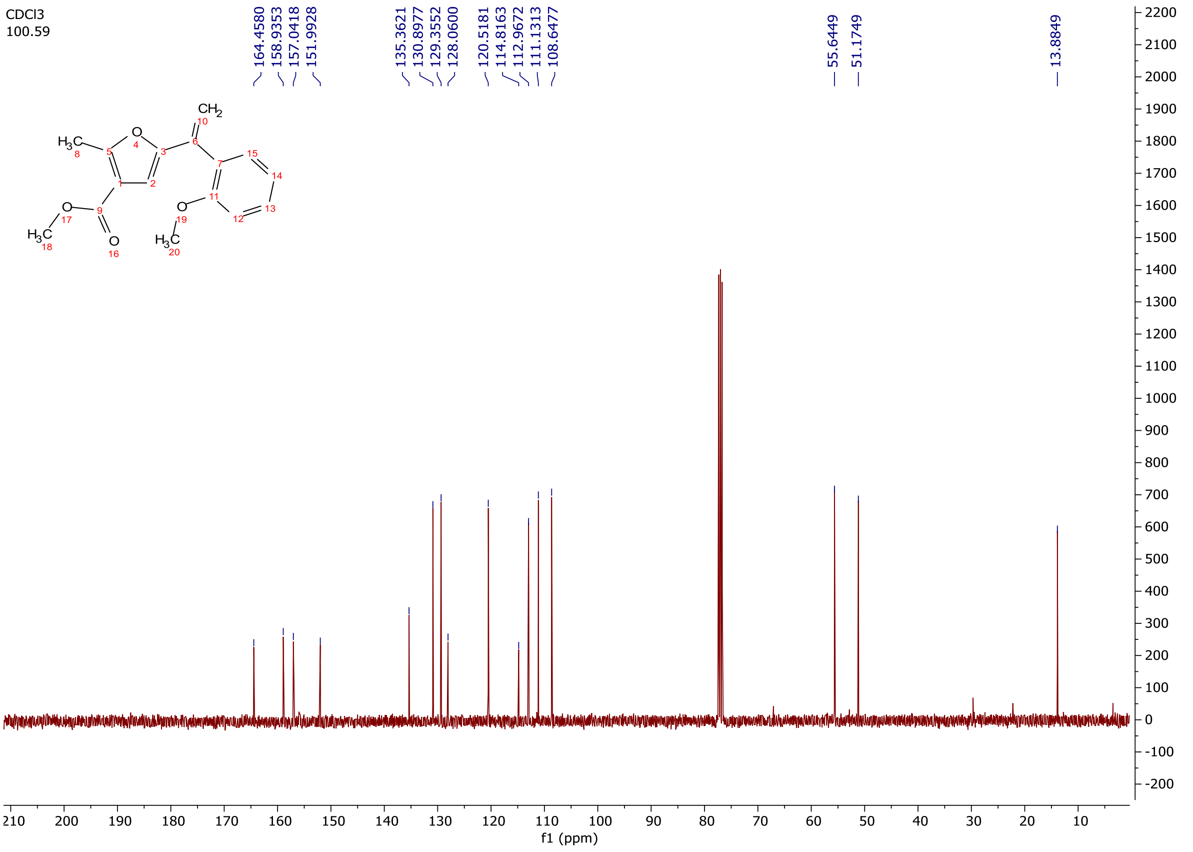

^{1}H NMR (500 MHz, $CDCl_3$) of compound **10**

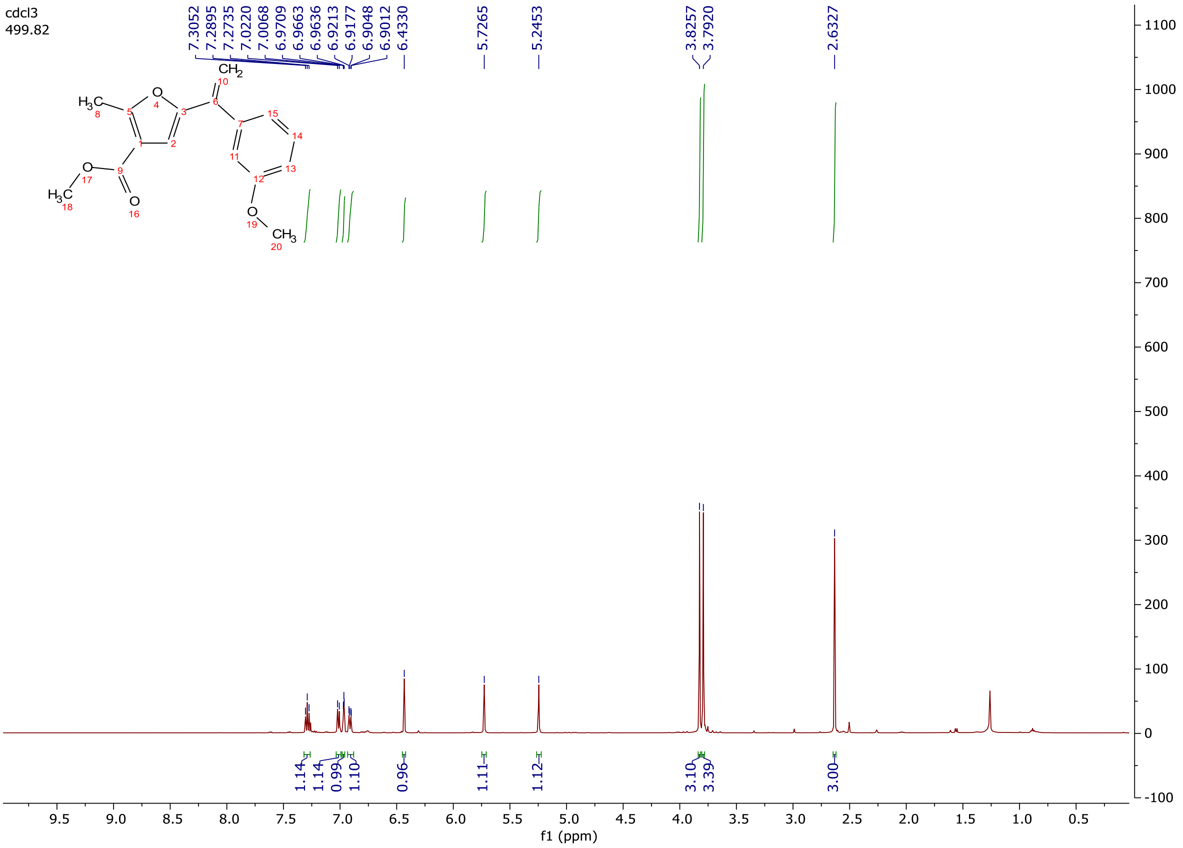

^{13}C{^{1}H} NMR (126 MHz, $CDCl_3$) of compound **10**

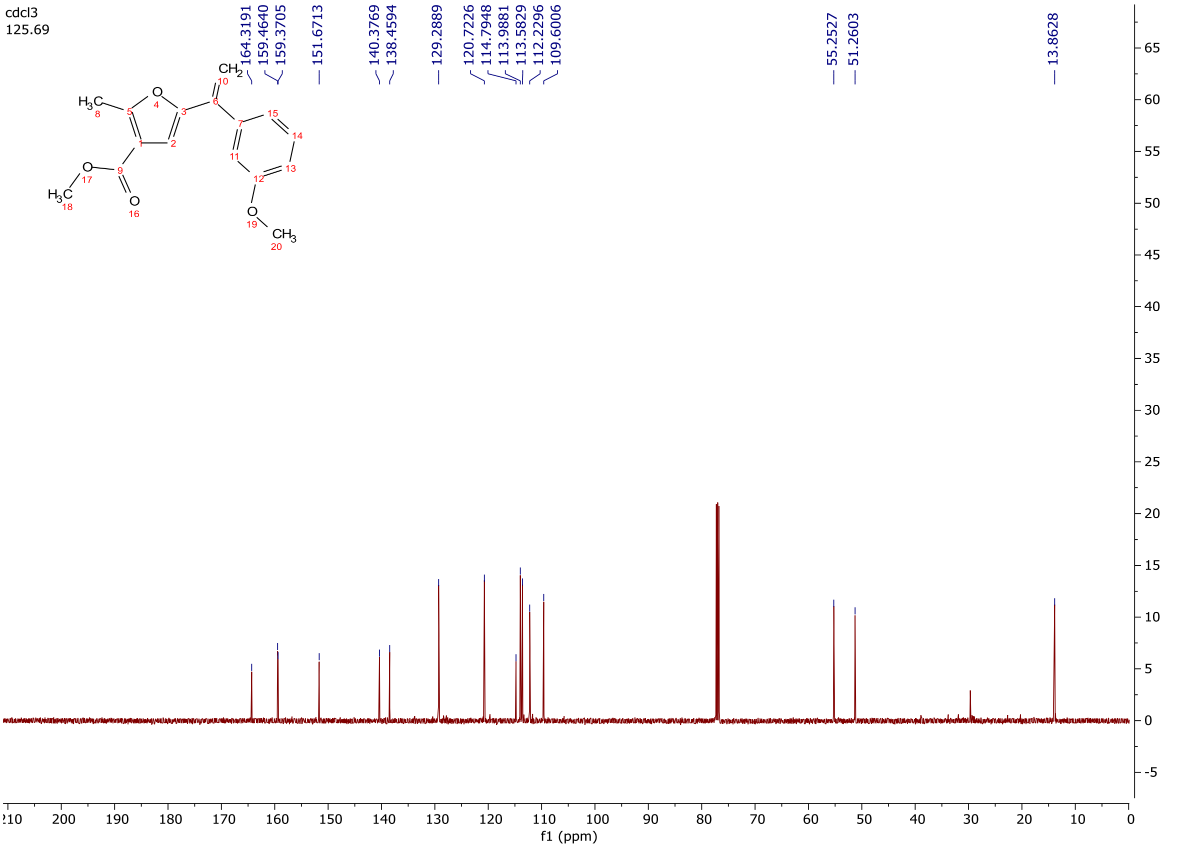

^{1}H NMR (400 MHz, $CDCl_3$) of compound **11**

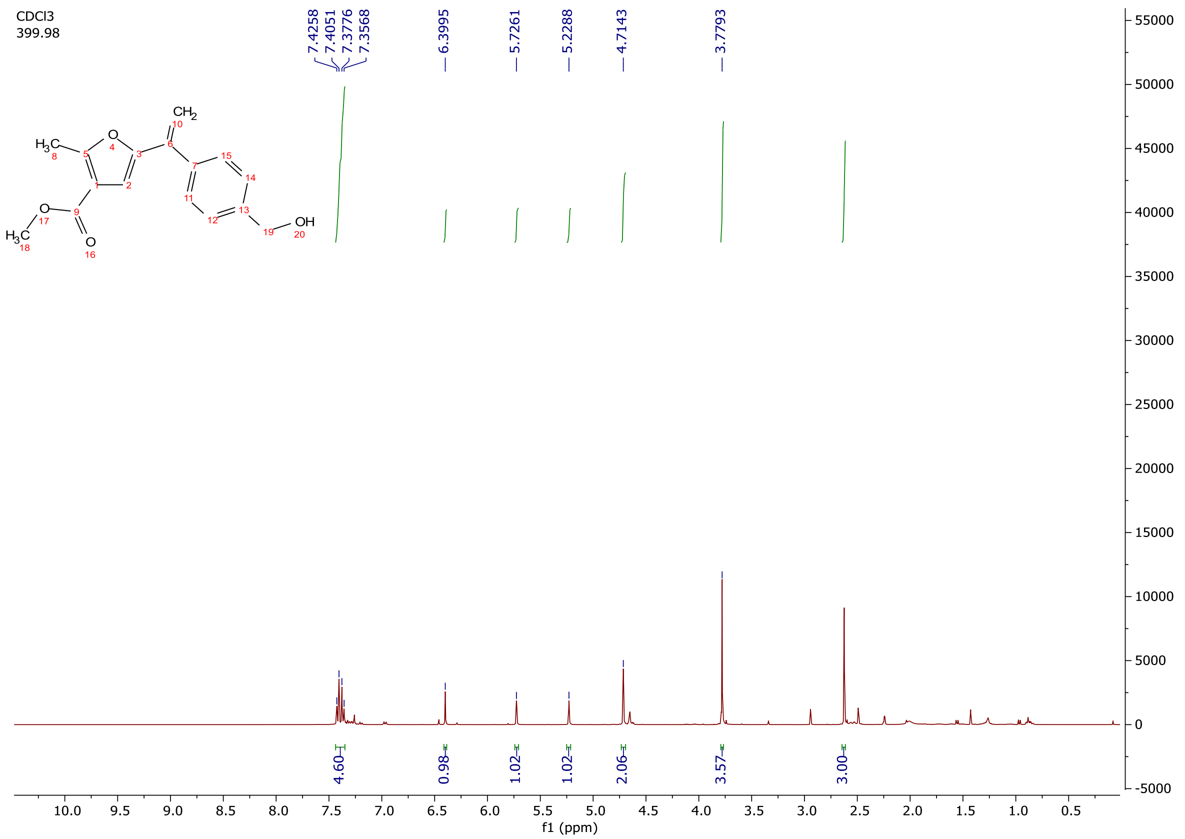

$^{13}C\{^{1}H\}$ NMR (101 MHz, $CDCl_3$) of compound **11**

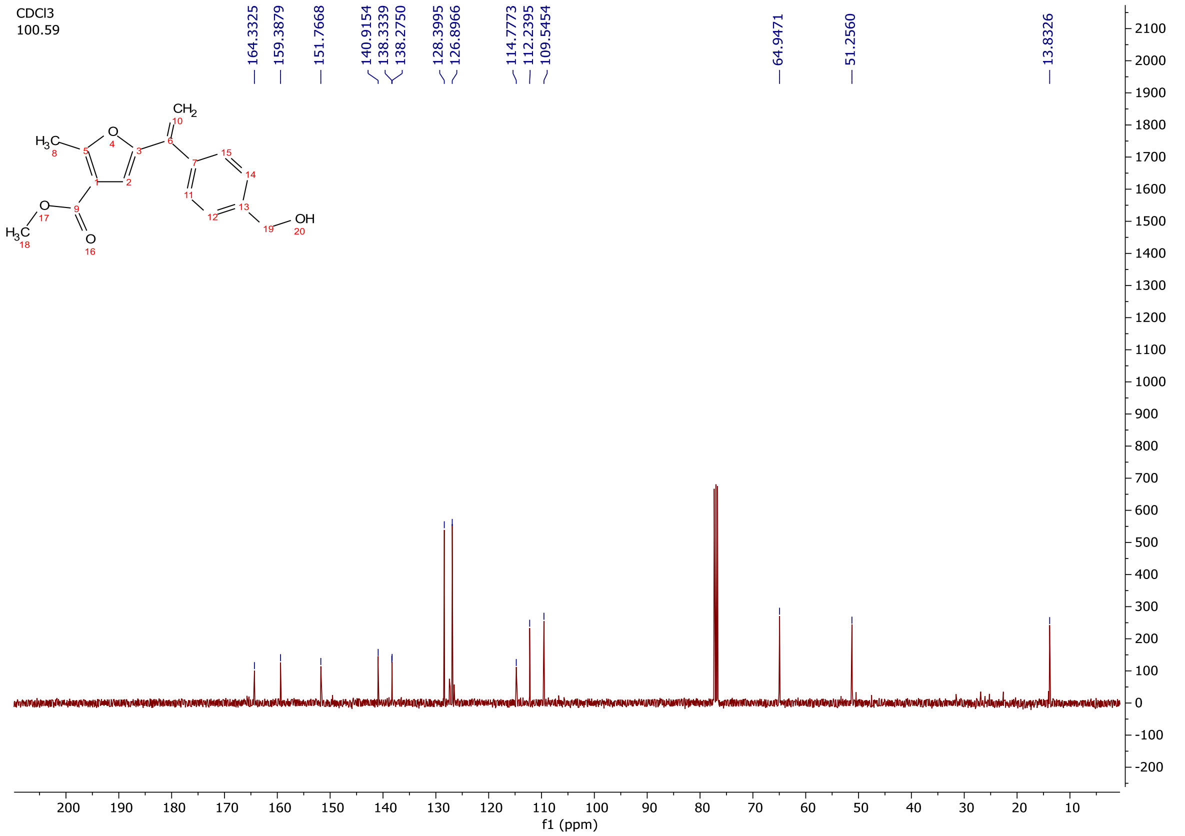

^{1}H NMR (400 MHz, CDCl$_3$) of compound **12**

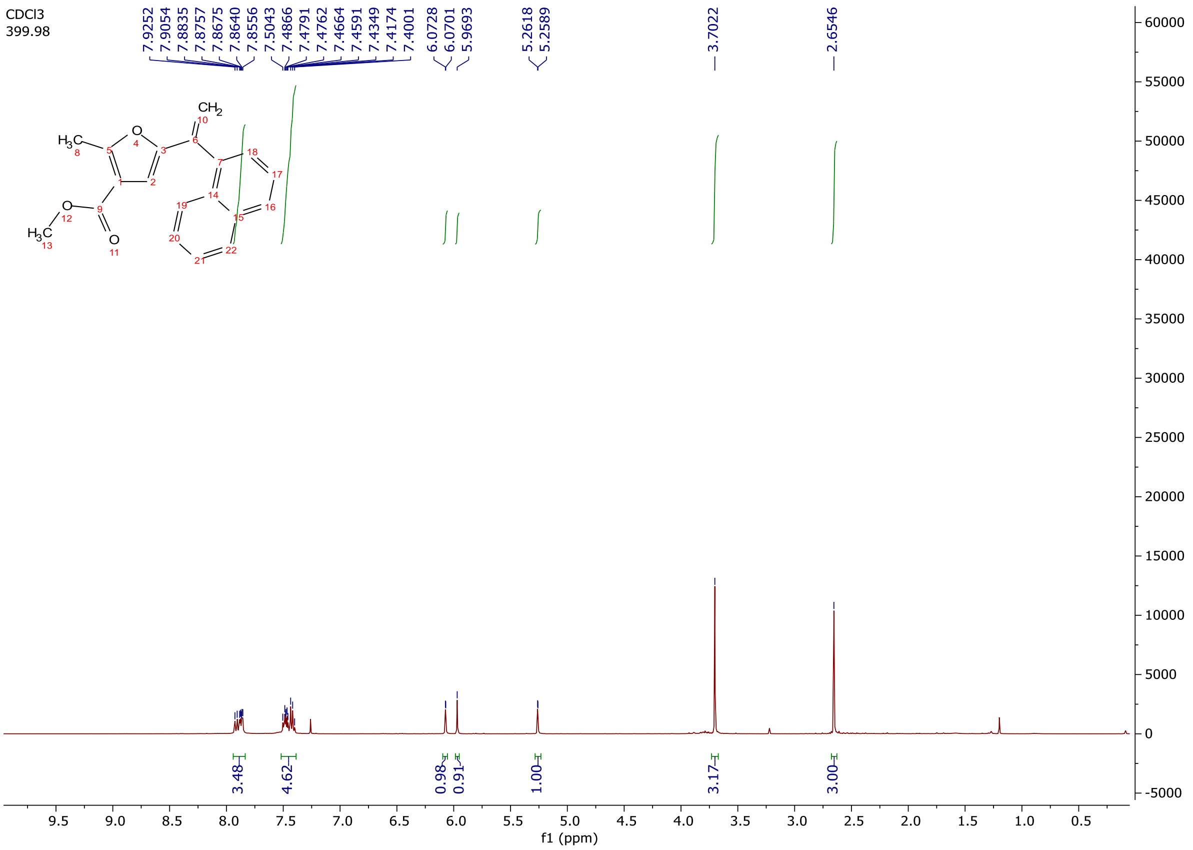

^{13}C{^{1}H} NMR (101 MHz, CDCl$_3$) of compound **12**

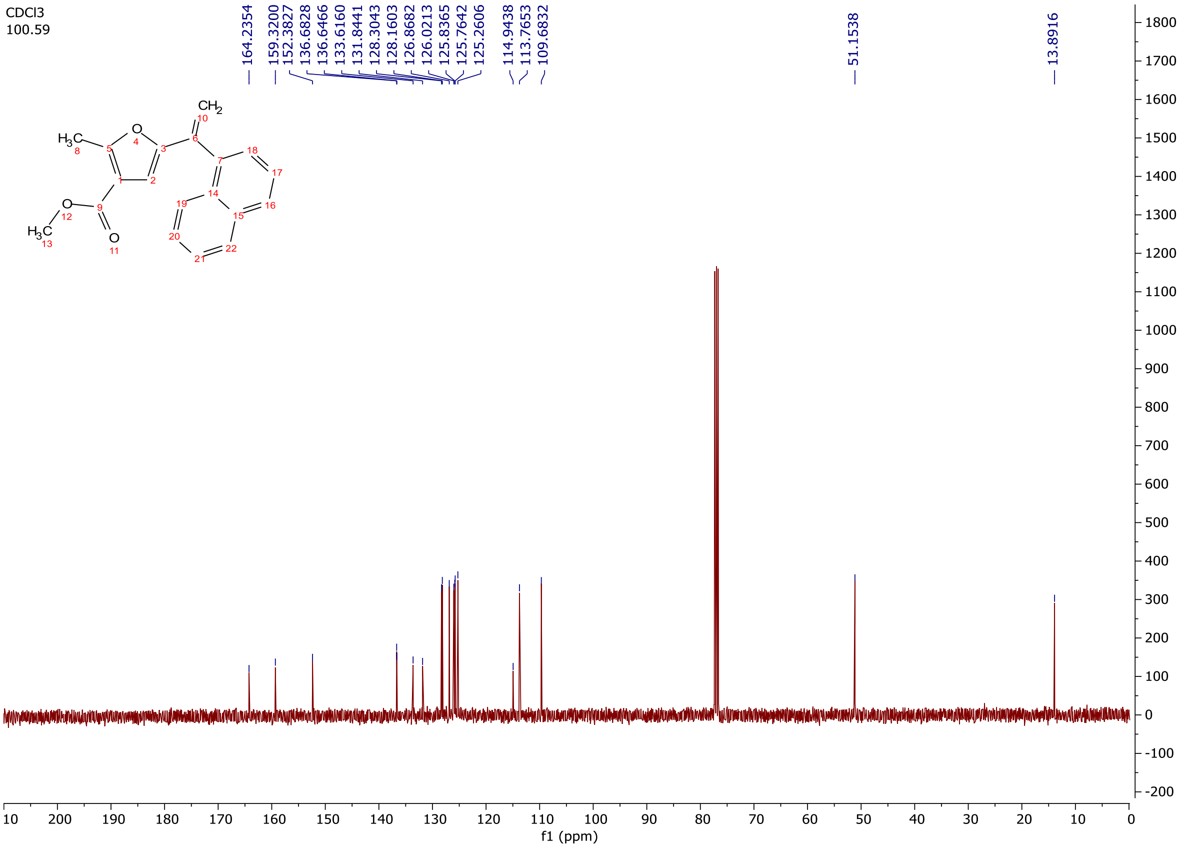

^{1}H NMR (400 MHz, $CDCl_3$) of compound **13**

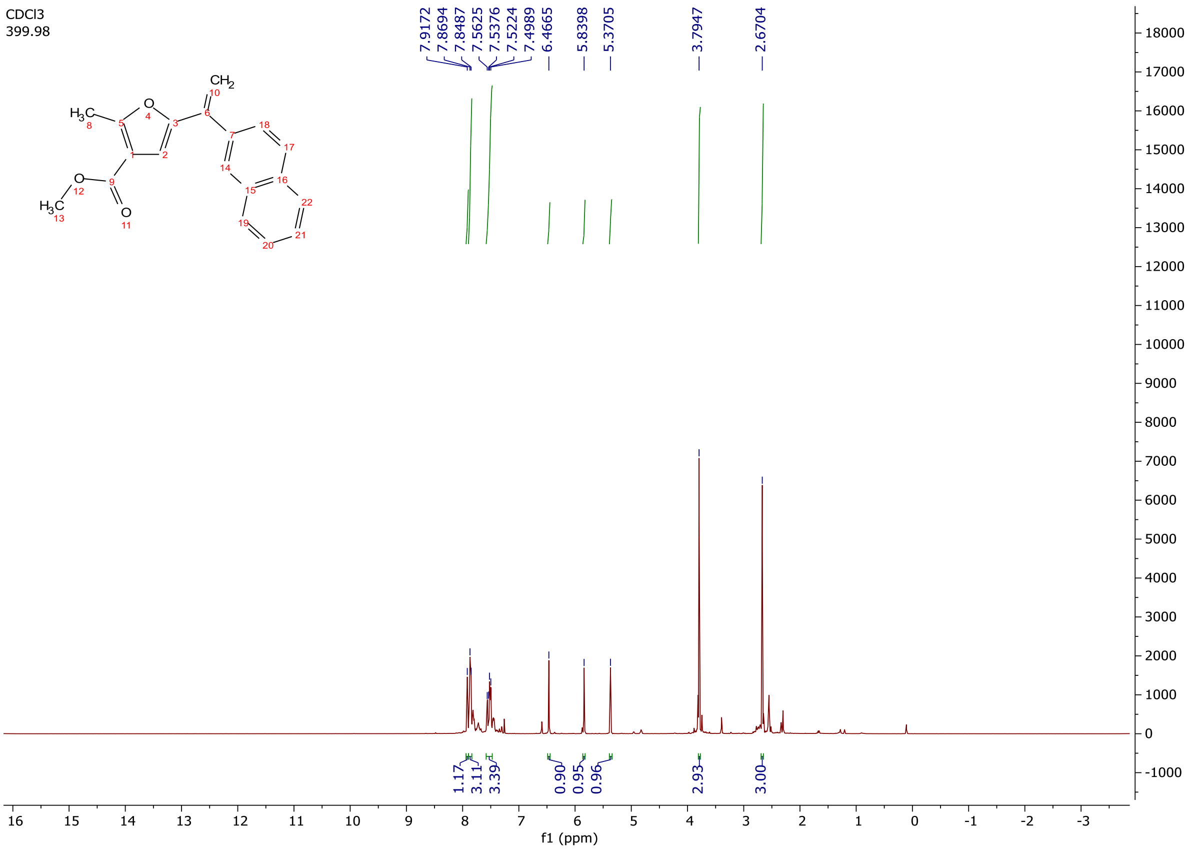

$^{13}C\{^{1}H\}$ NMR (101 MHz, $CDCl_3$) of compound **13**

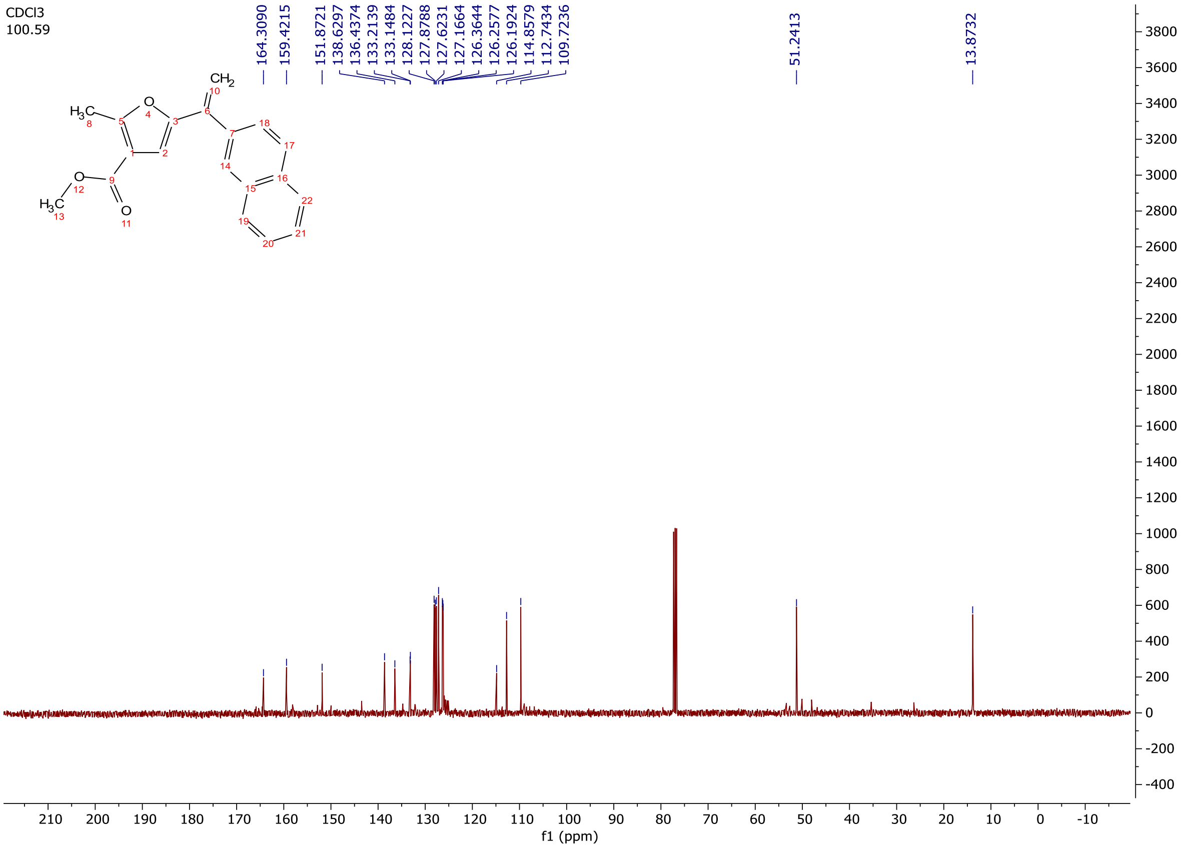

^{1}H NMR (400 MHz, $CDCl_3$) of compound **14**

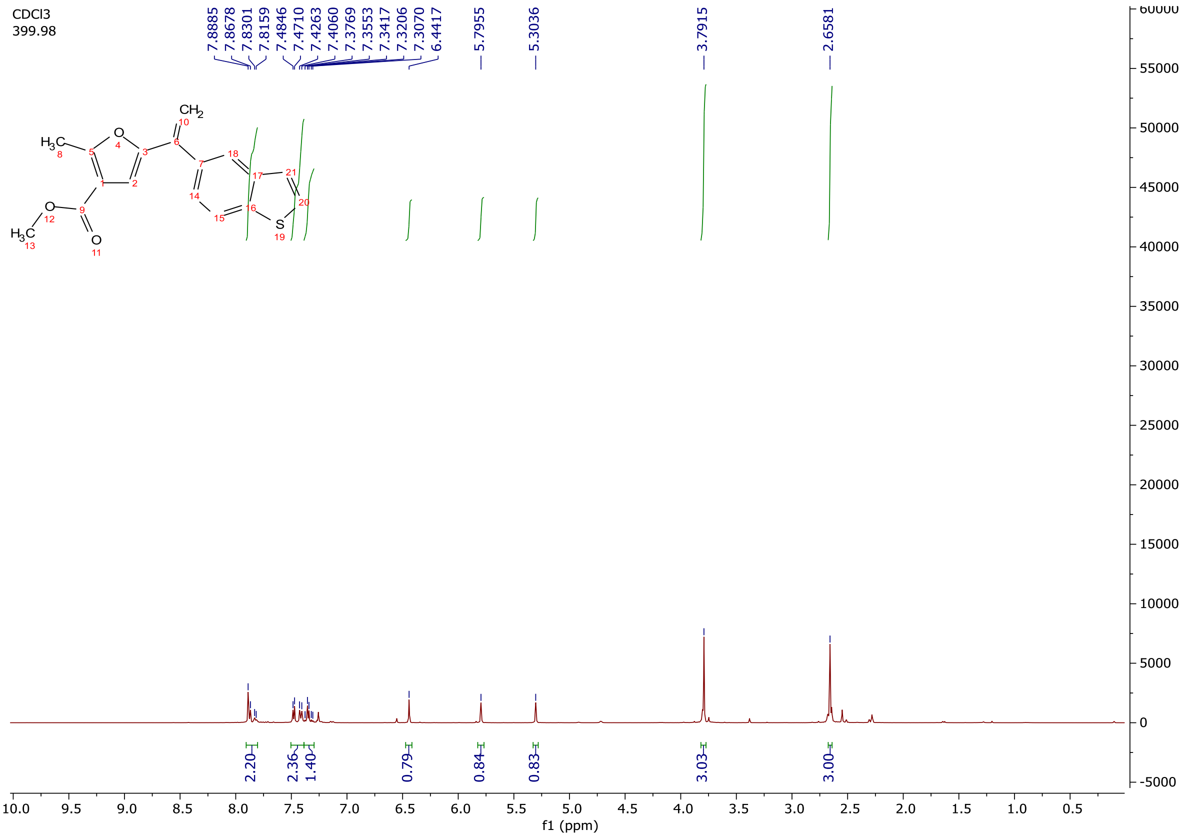

$^{13}C\{^{1}H\}$ NMR (101 MHz, $CDCl_3$) of compound **14**

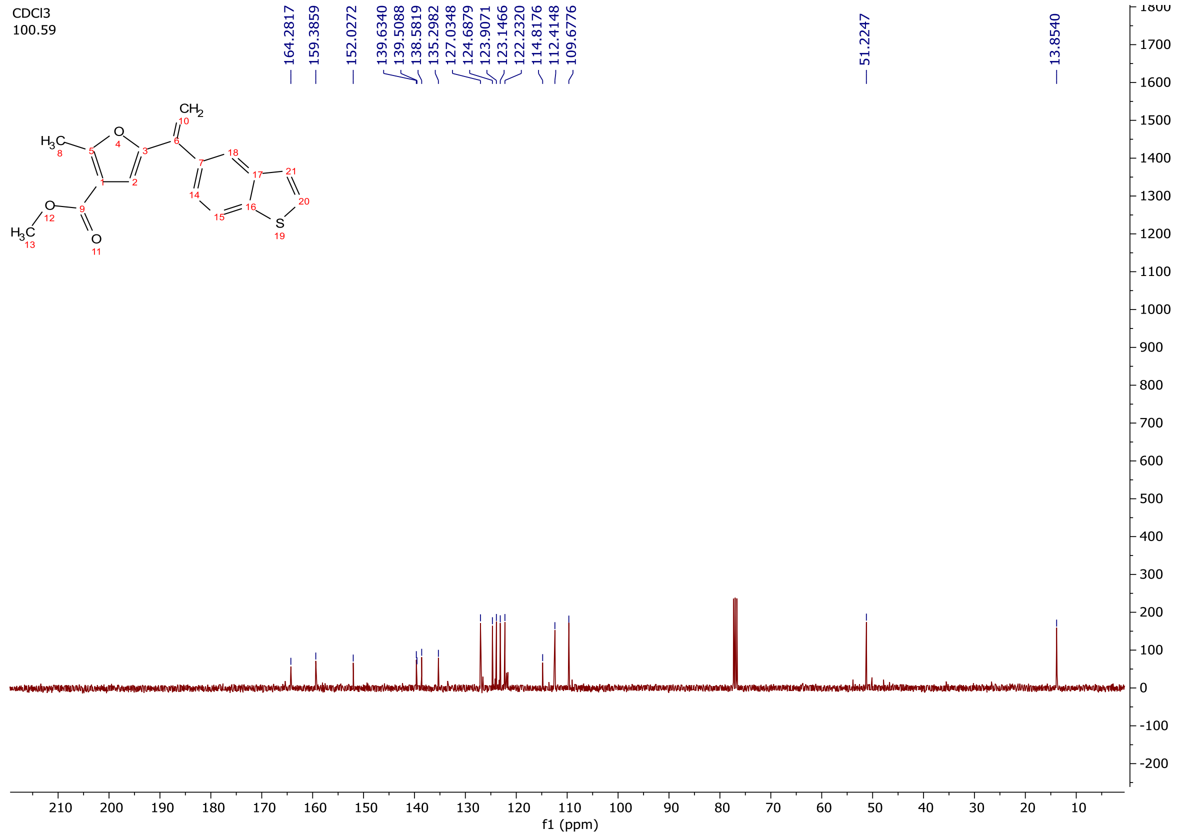

^{1}H NMR (400 MHz, $CDCl_3$) of compound **15**

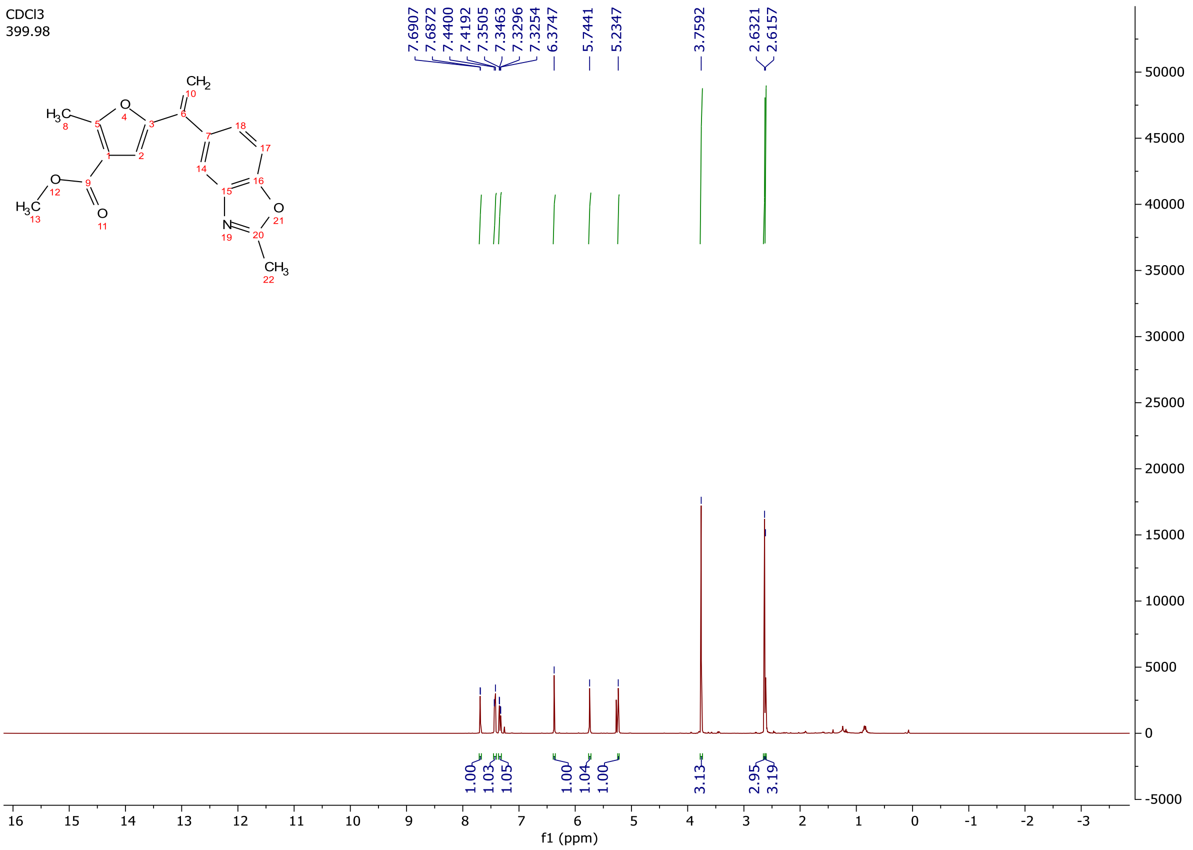

$^{13}C\{^{1}H\}$ NMR (101 MHz, $CDCl_3$) of compound **15**

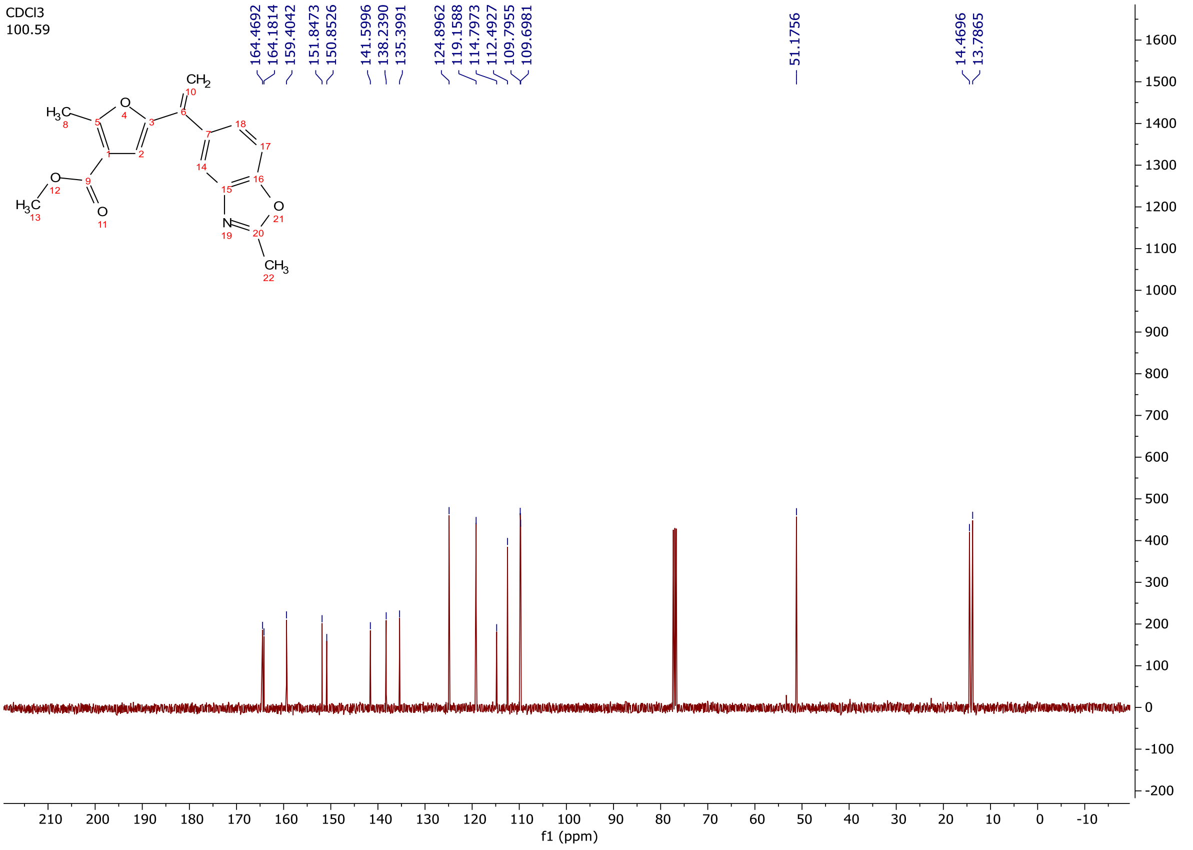

^{1}H NMR (400 MHz, $CDCl_3$) of compound **16**

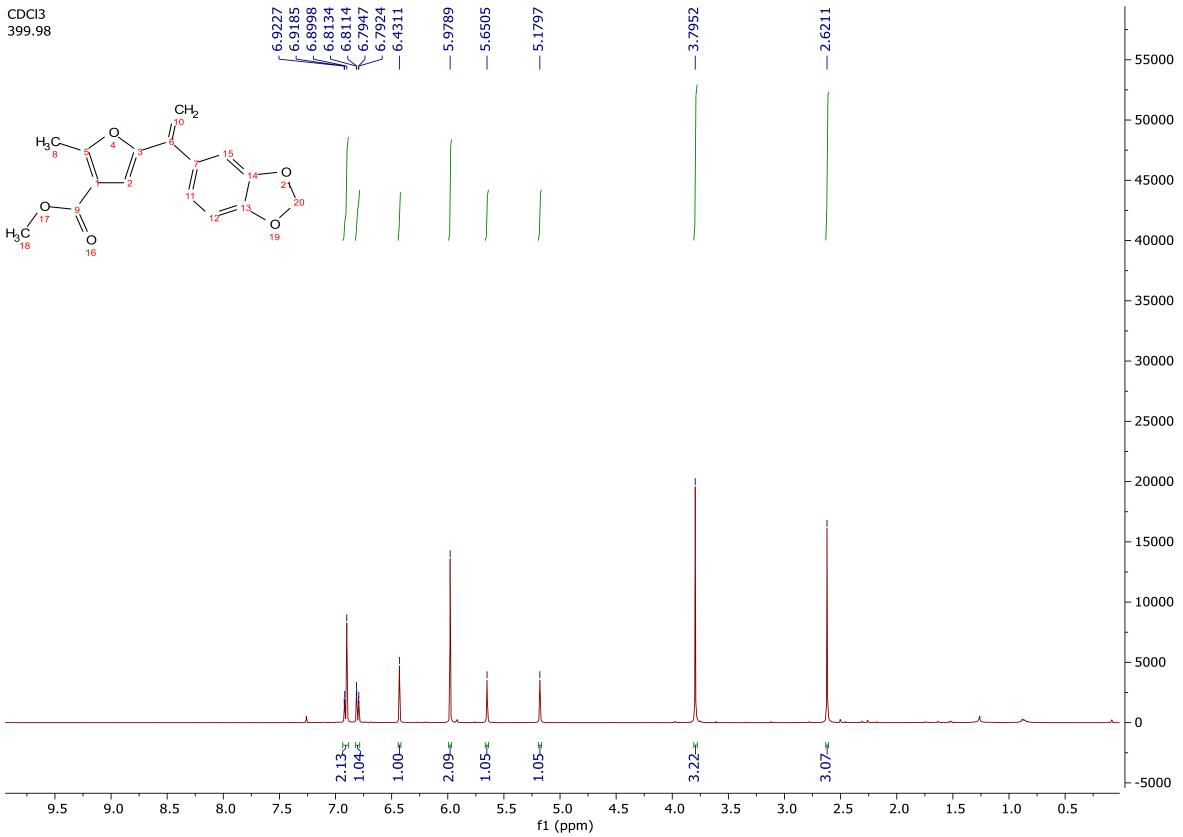

^{13}C{^{1}H} NMR (101 MHz, $CDCl_3$) of compound **16**

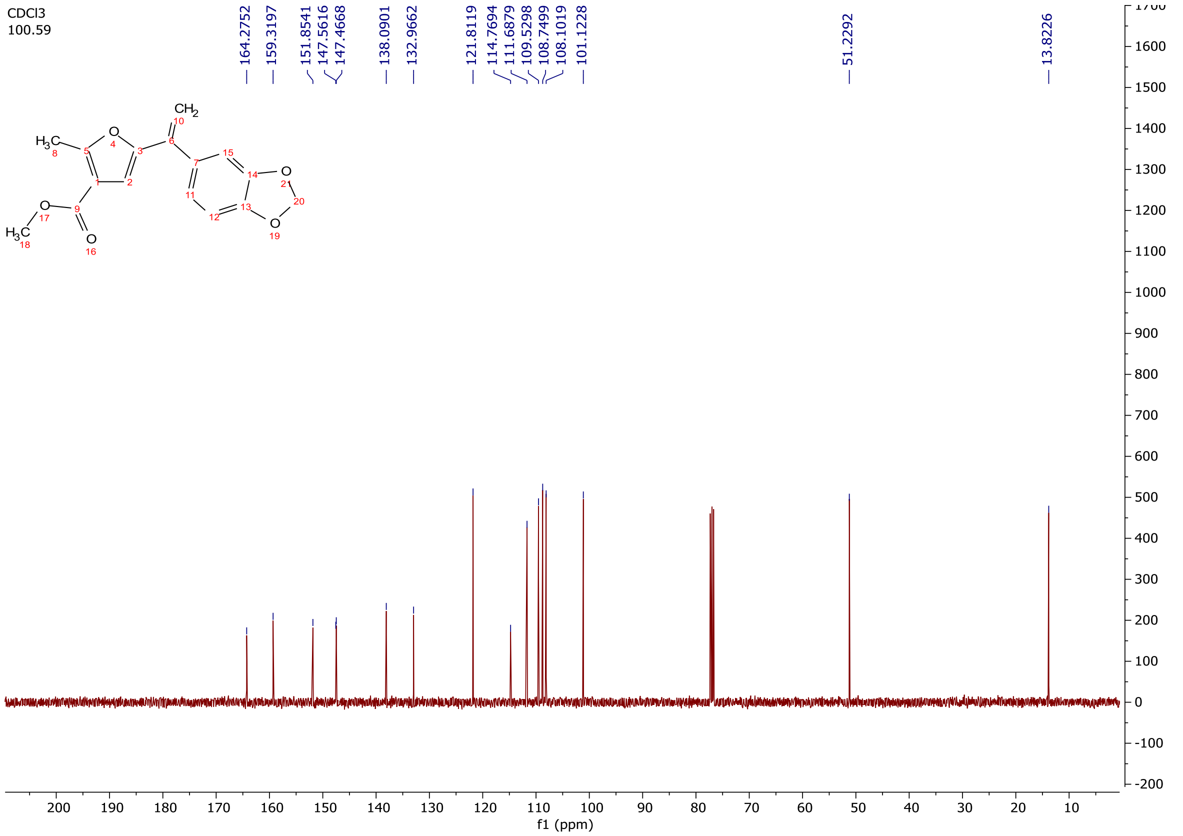

^{1}H NMR (500 MHz, $CDCl_3$) of compound **17**

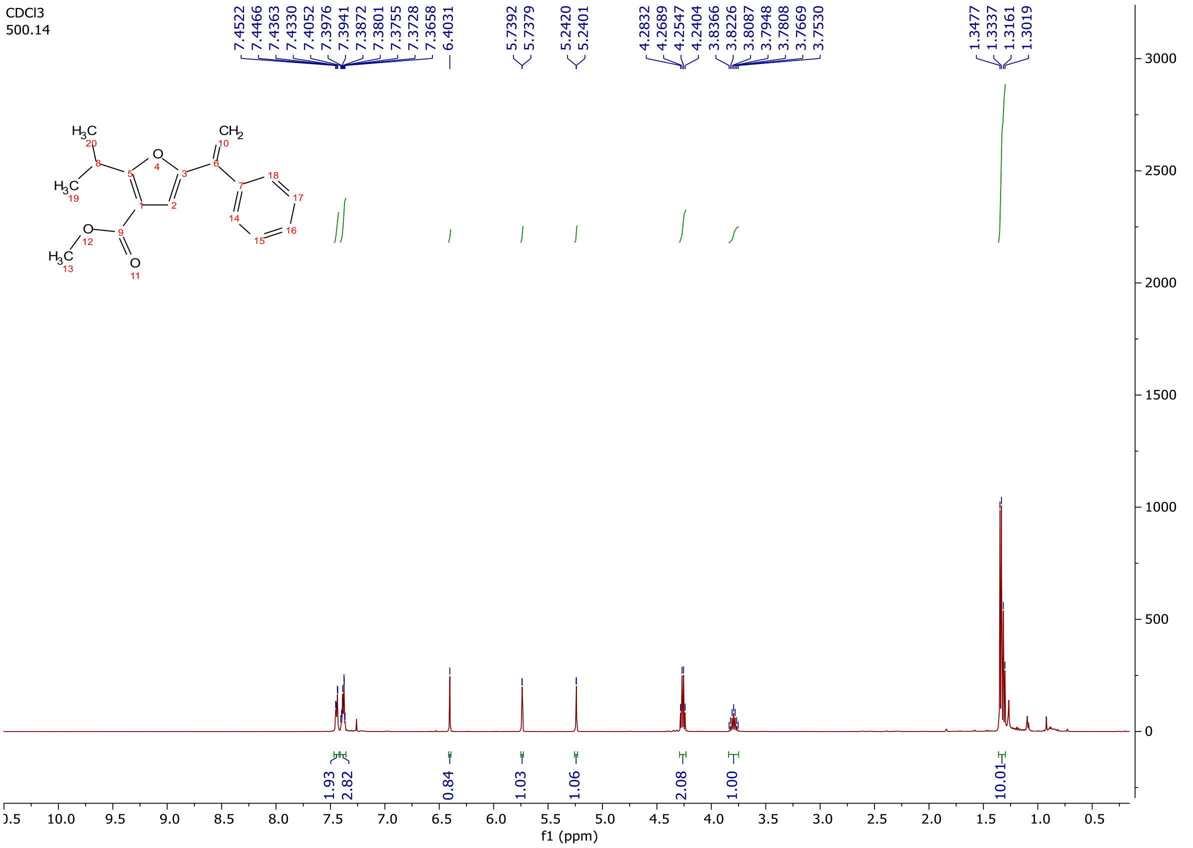

^{13}C{^{1}H} NMR (126 MHz, $CDCl_3$) of compound **17**

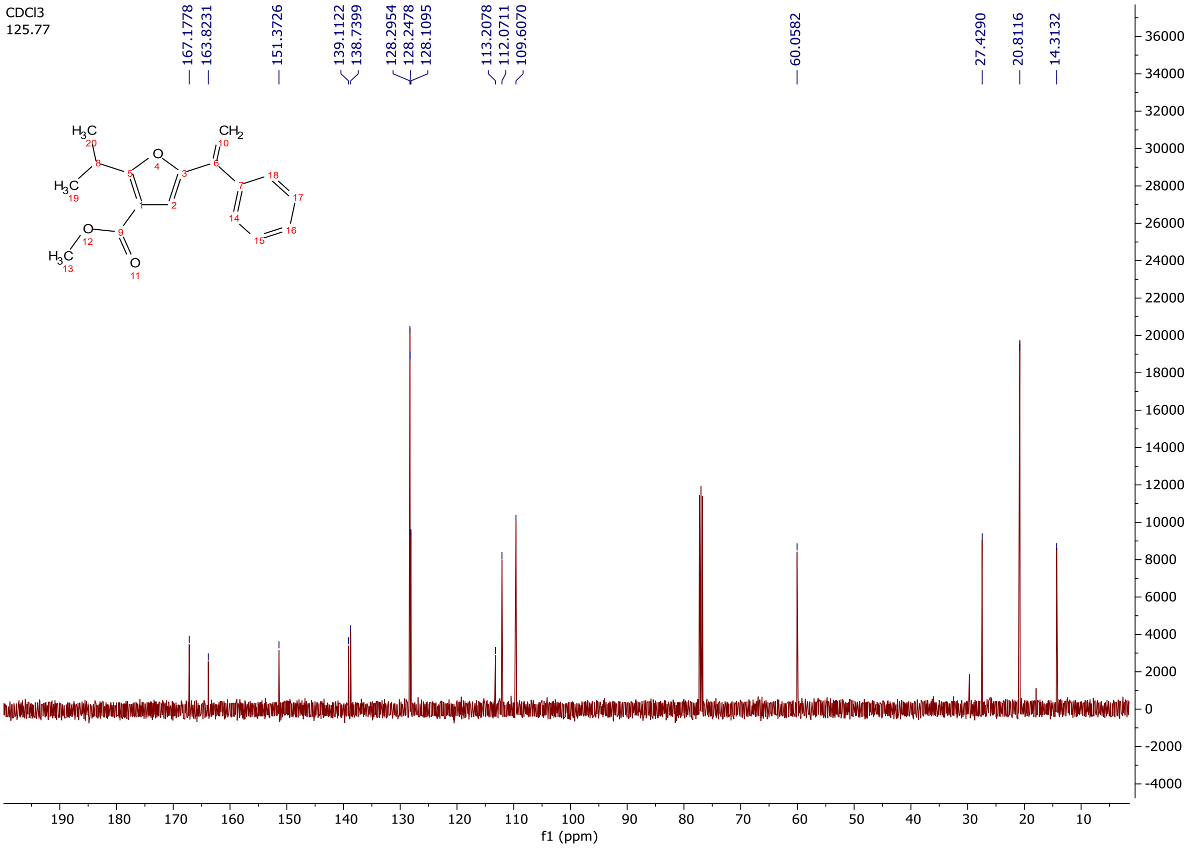

^{1}H NMR (500 MHz, $CDCl_3$) of compound **18**

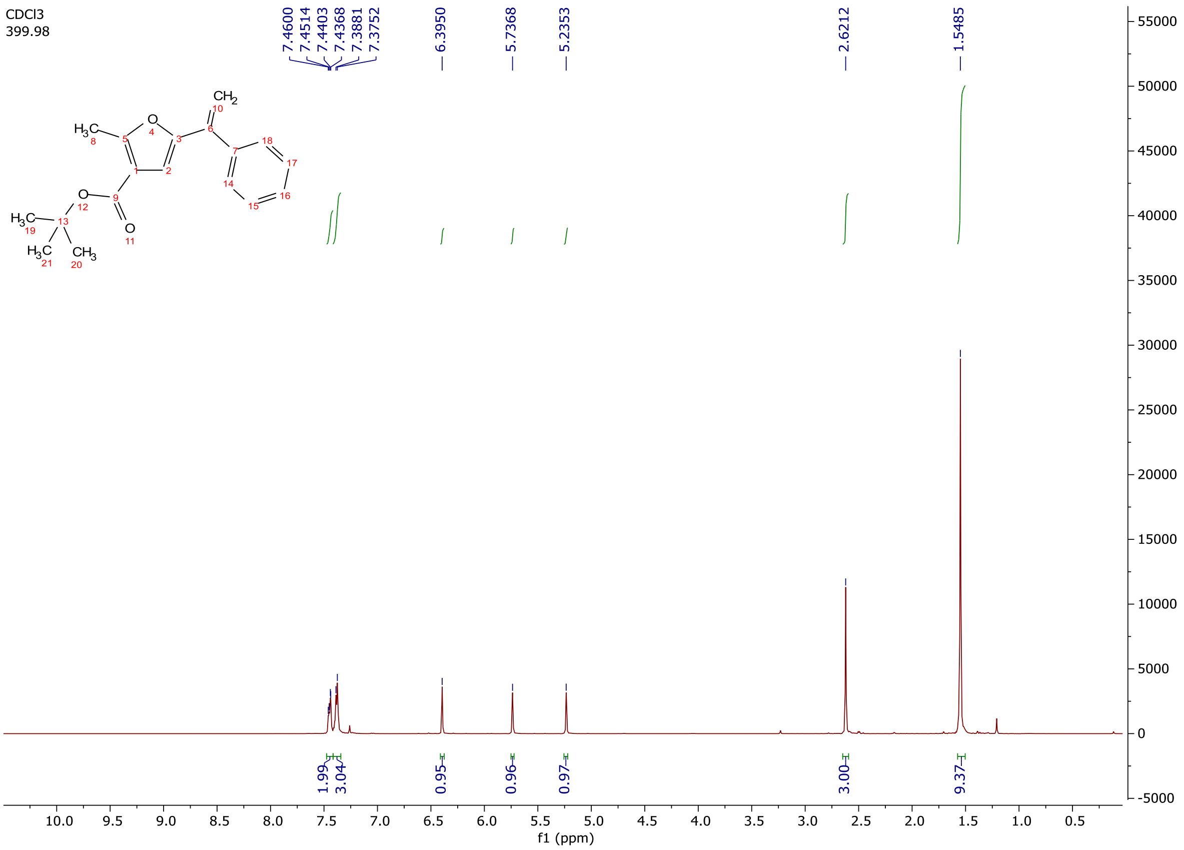

$^{13}C\{^{1}H\}$ NMR (101 MHz, $CDCl_3$) of compound **18**

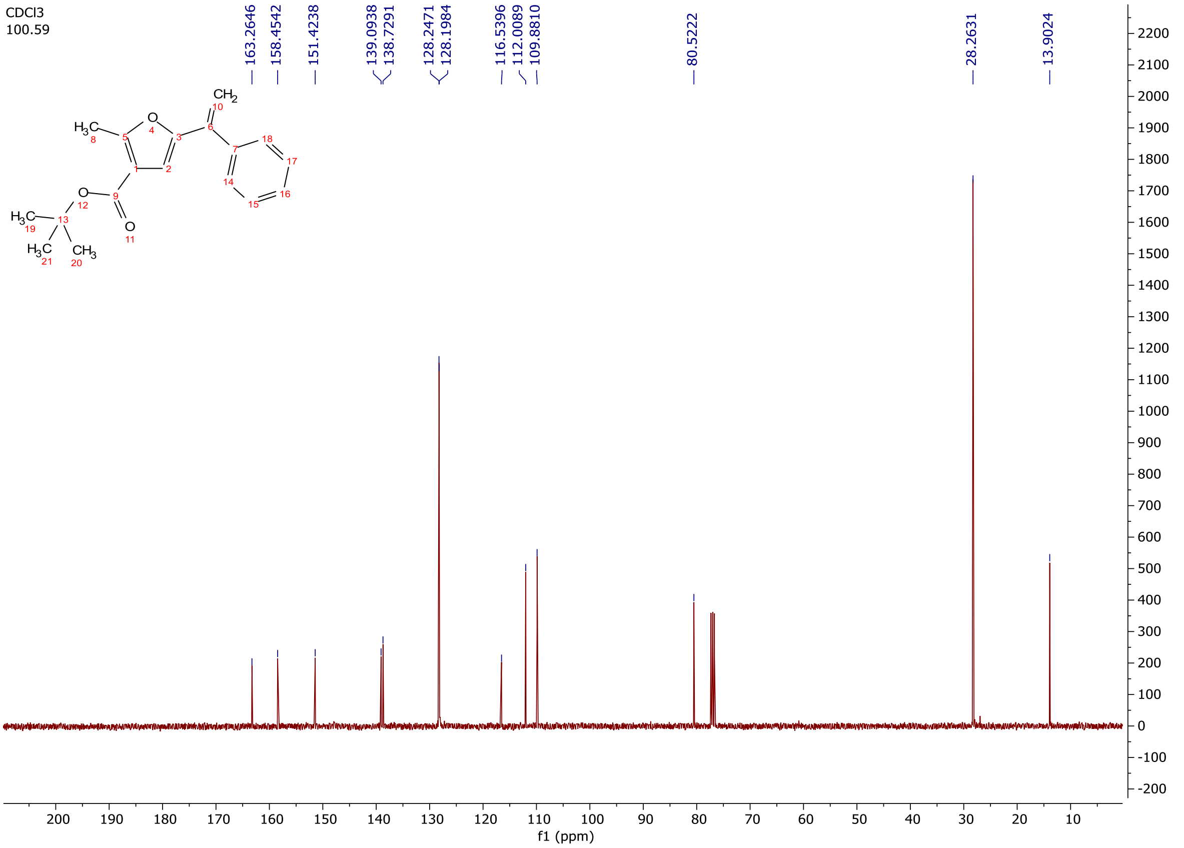

^{1}H NMR (400 MHz, $CDCl_3$) of compound **19**

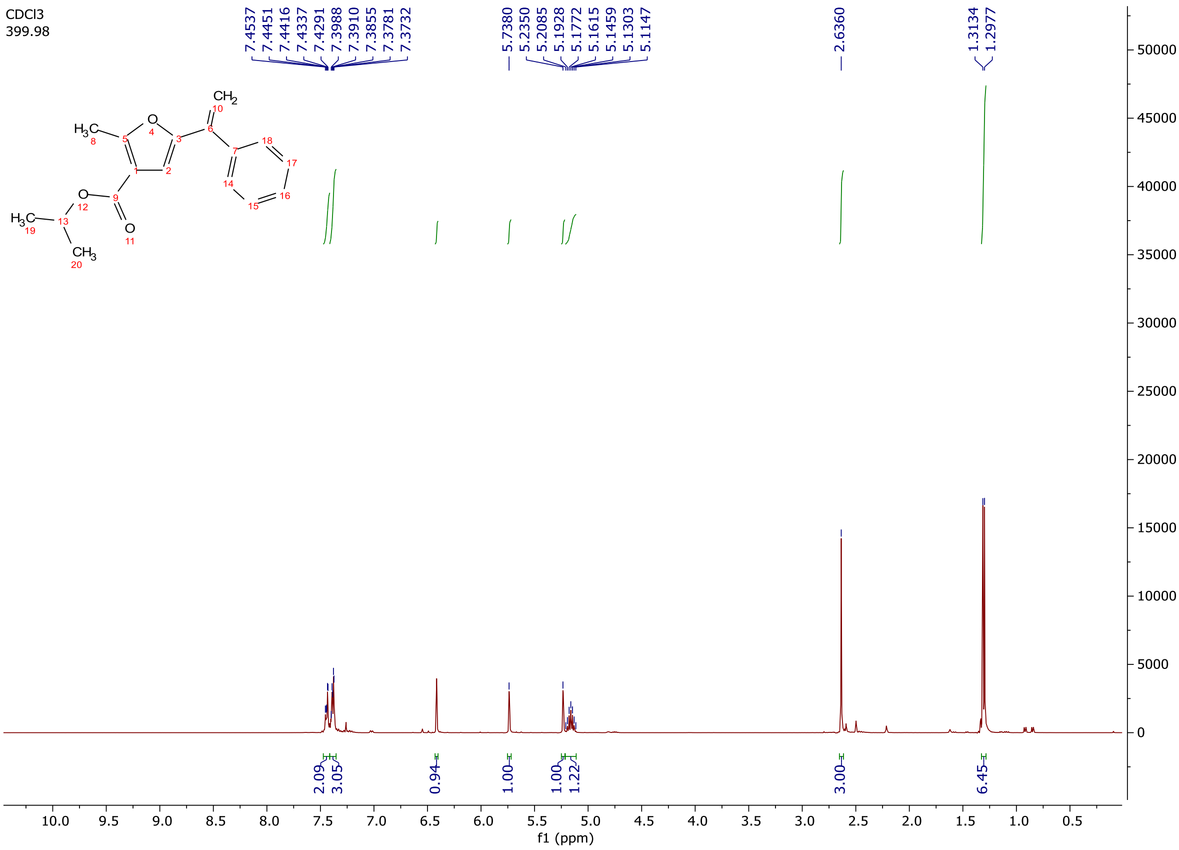

$^{13}C\{^{1}H\}$ NMR (101 MHz, $CDCl_3$) of compound **19**

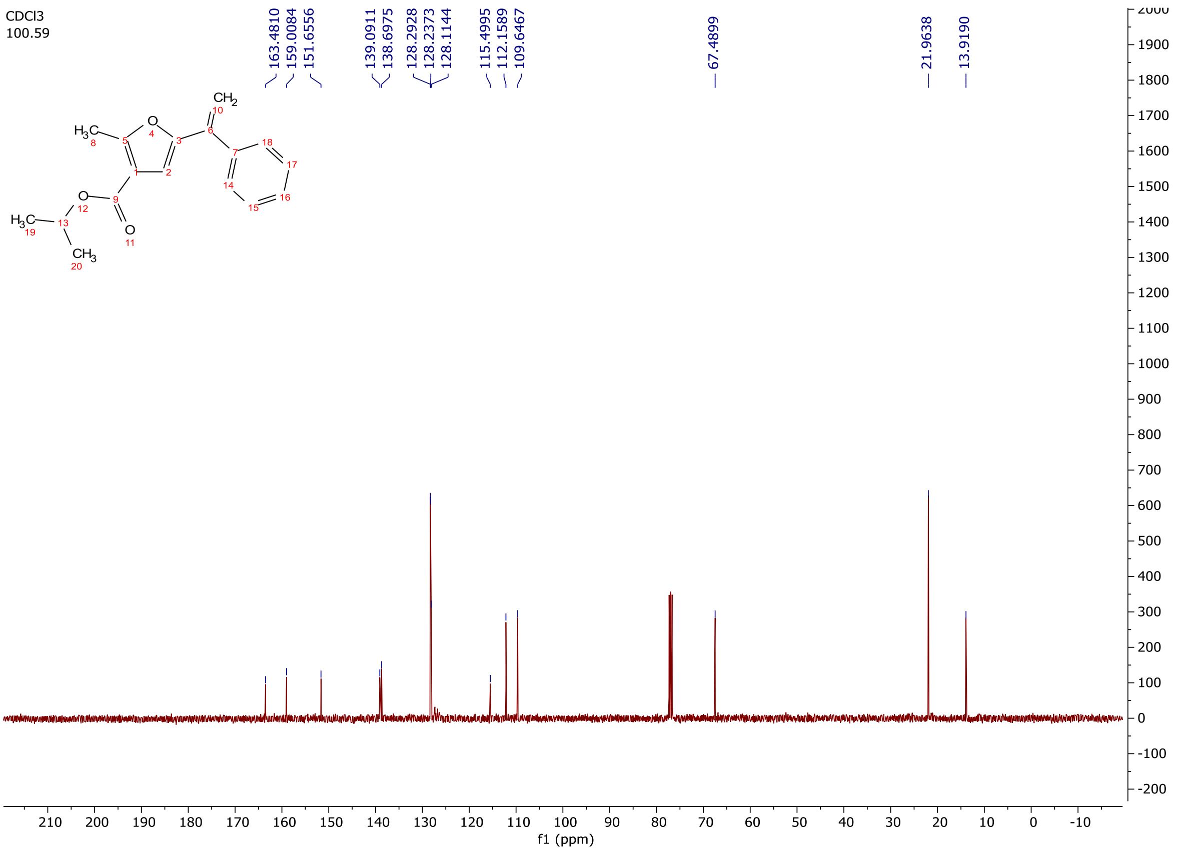

^{1}H NMR (500 MHz, $CDCl_3$) of compound **20**

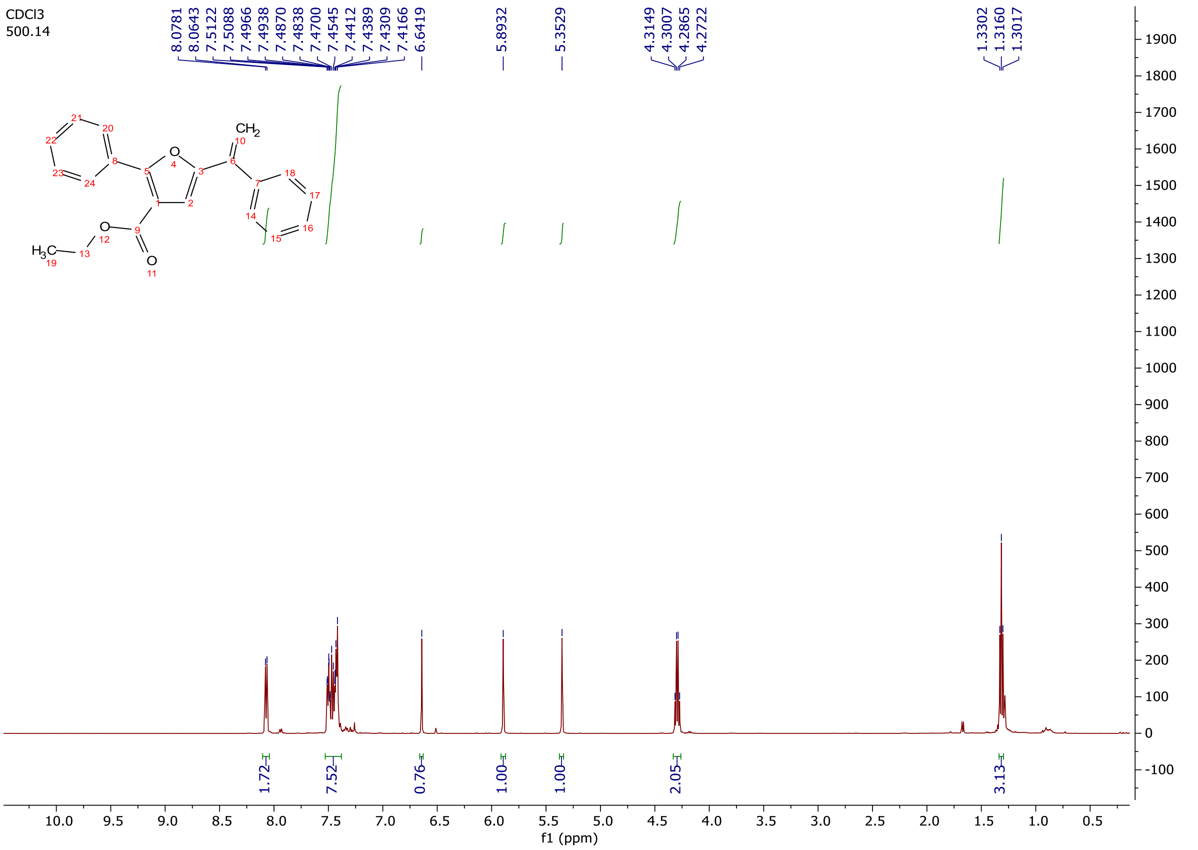

$^{13}C\{^{1}H\}$ NMR (126 MHz, $CDCl_3$) of compound **20**

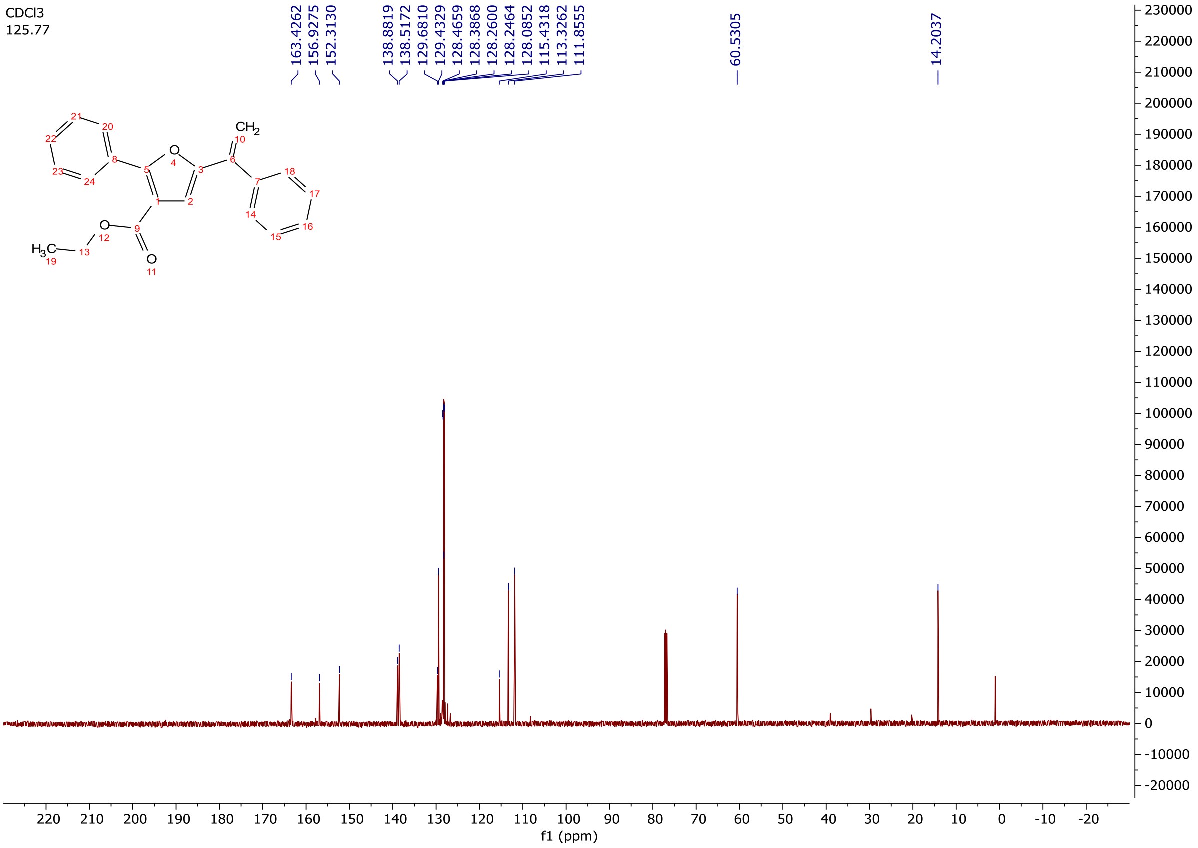

^{1}H NMR (400 MHz, $CDCl_3$) of compound **21a**

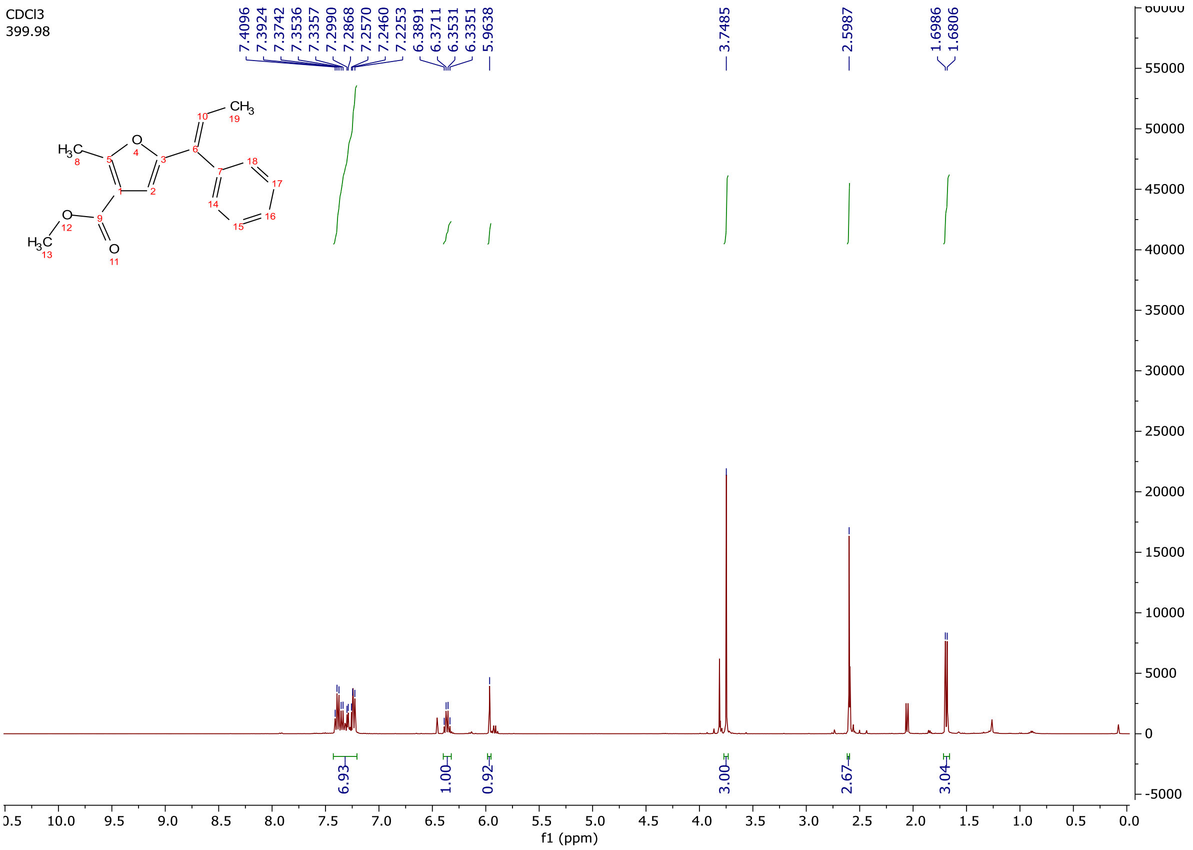

^{13}C{^{1}H} NMR (101 MHz, $CDCl_3$) of compound **21a**

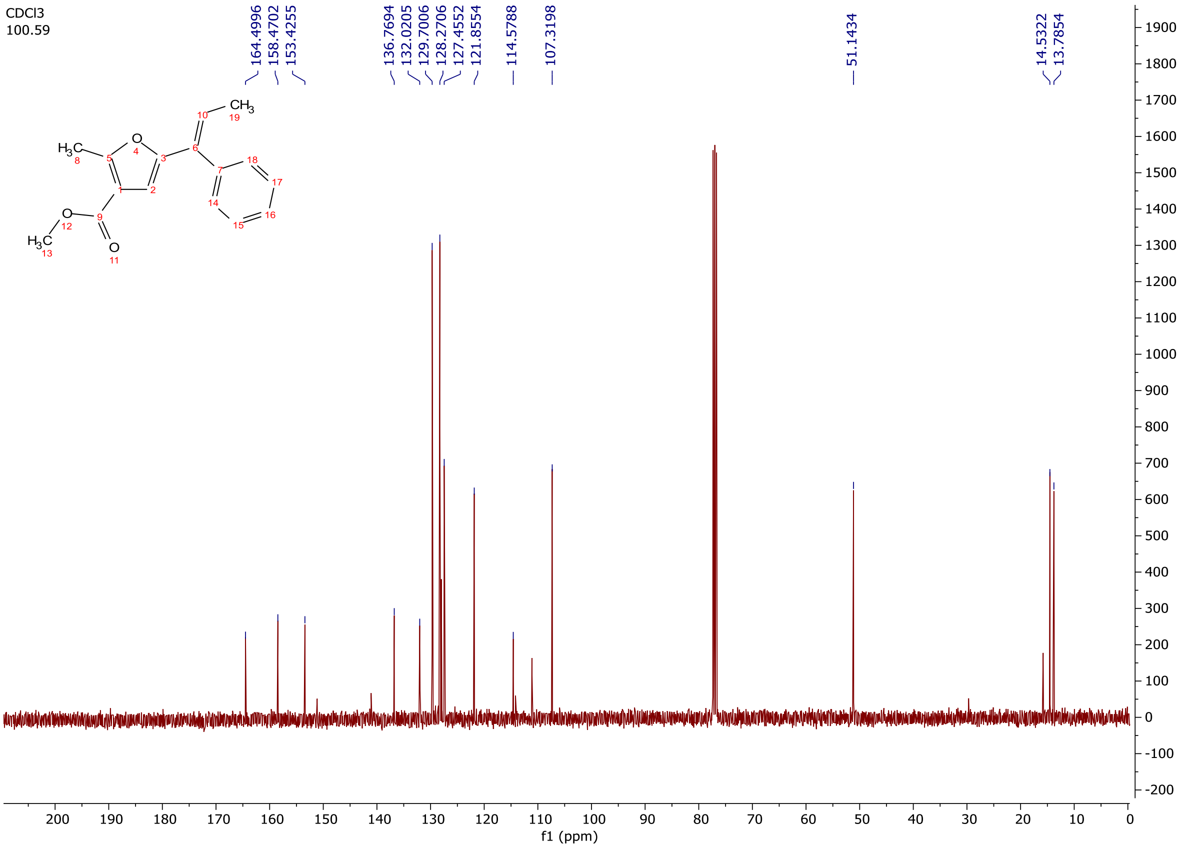

^{1}H NMR (400 MHz, $CDCl_3$) of compound **21b**

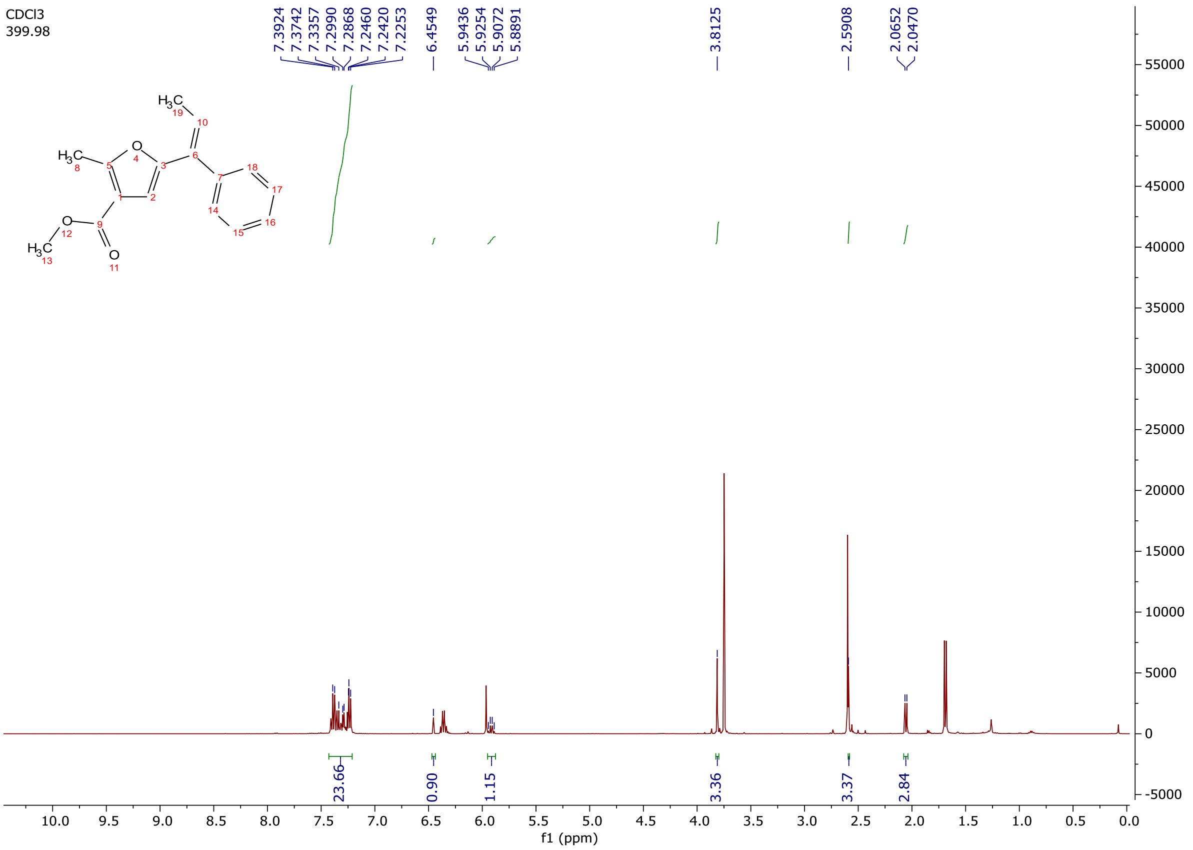

$^{13}C\{^{1}H\}$ NMR (101 MHz, $CDCl_3$) of compound **21b**

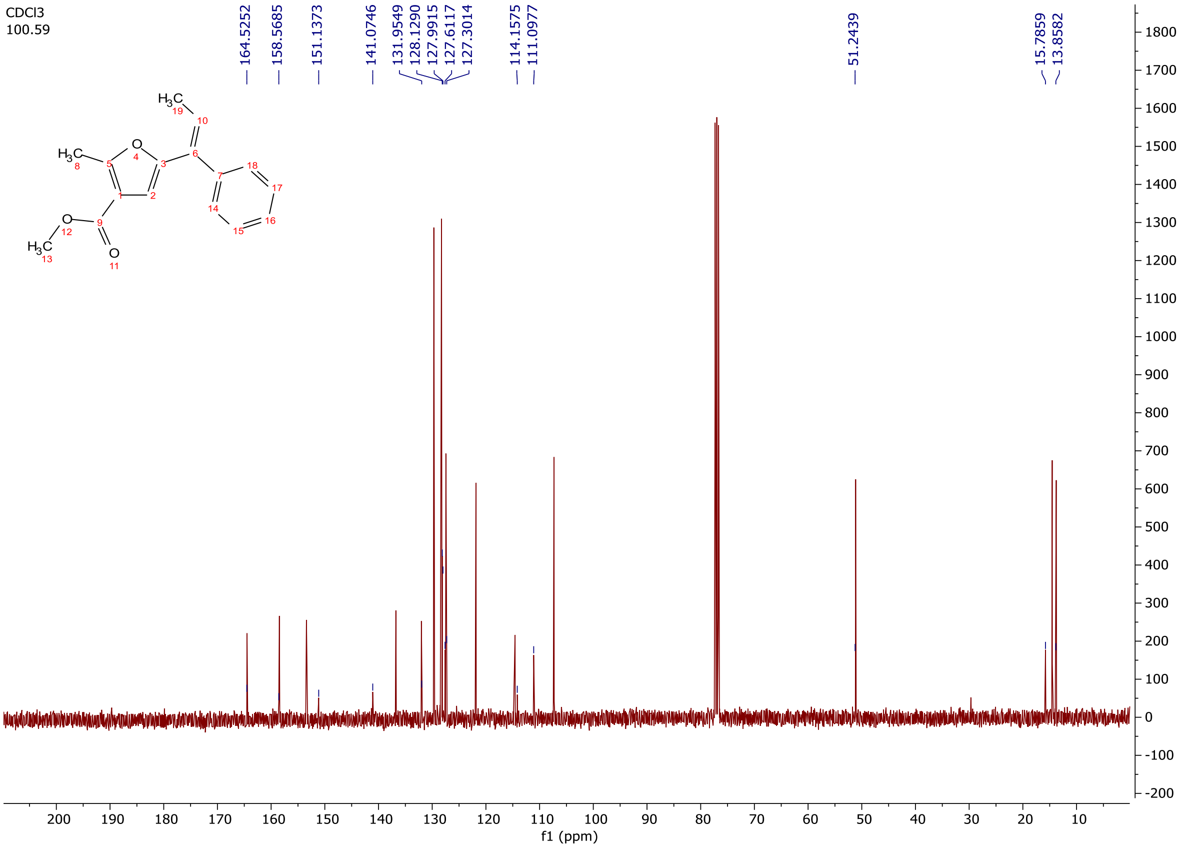

^{1}H NMR (500 MHz, $CDCl_3$) of compound **22a**

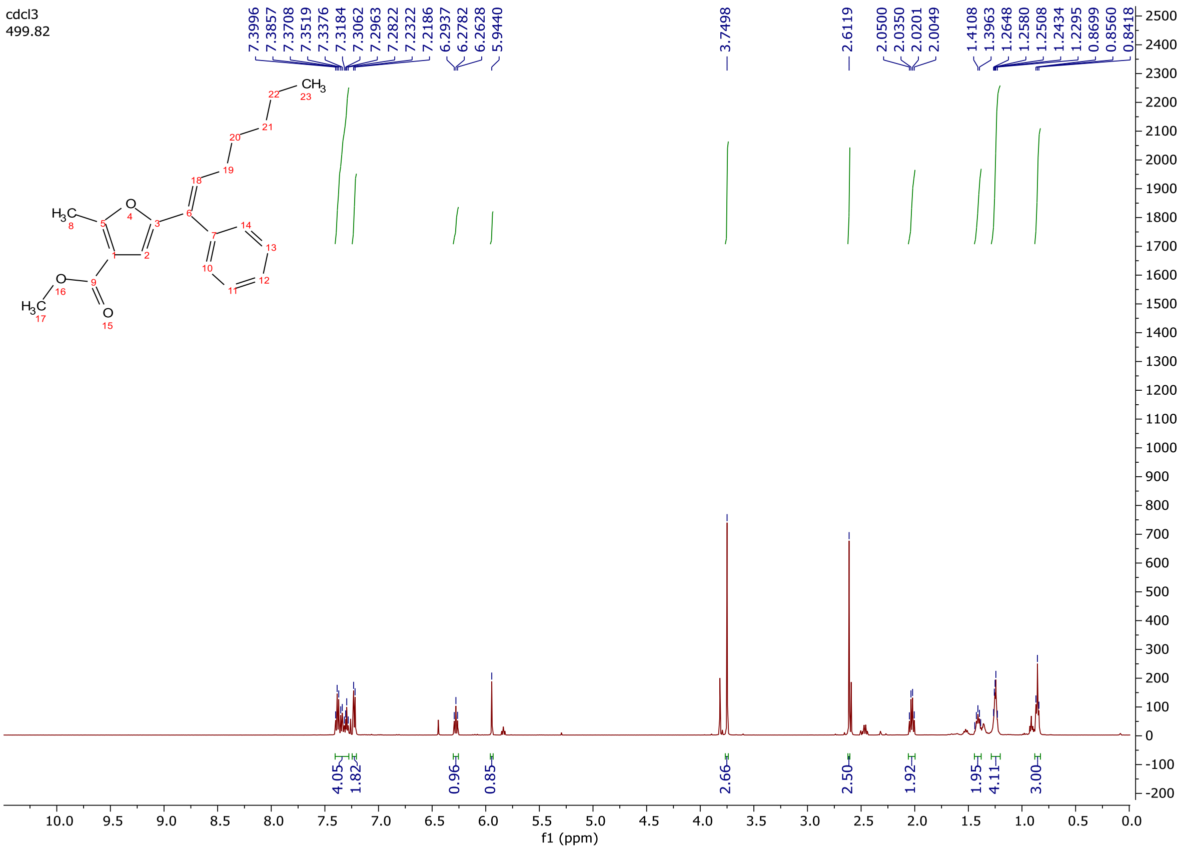

^{13}C{^{1}H} NMR (126 MHz, $CDCl_3$) of compound **22a**

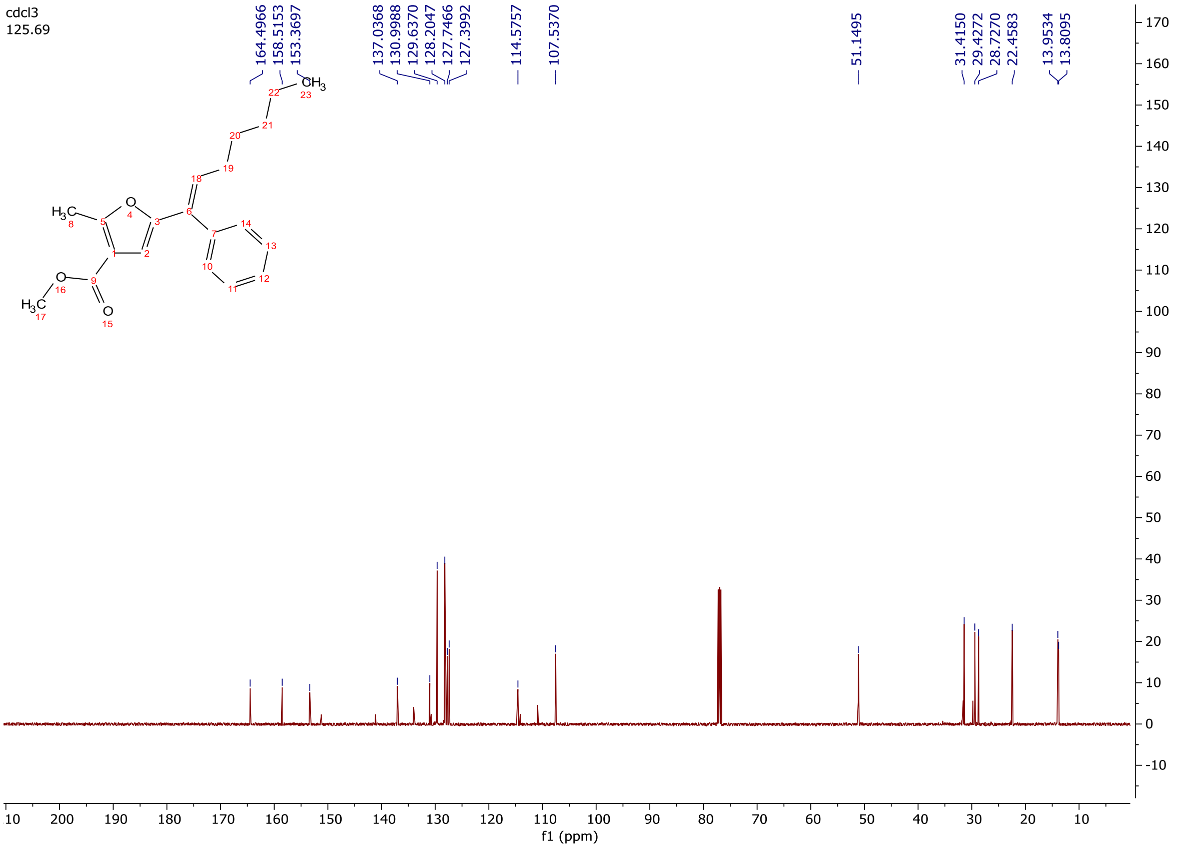

^{1}H NMR (500 MHz, $CDCl_3$) of compound **22b**

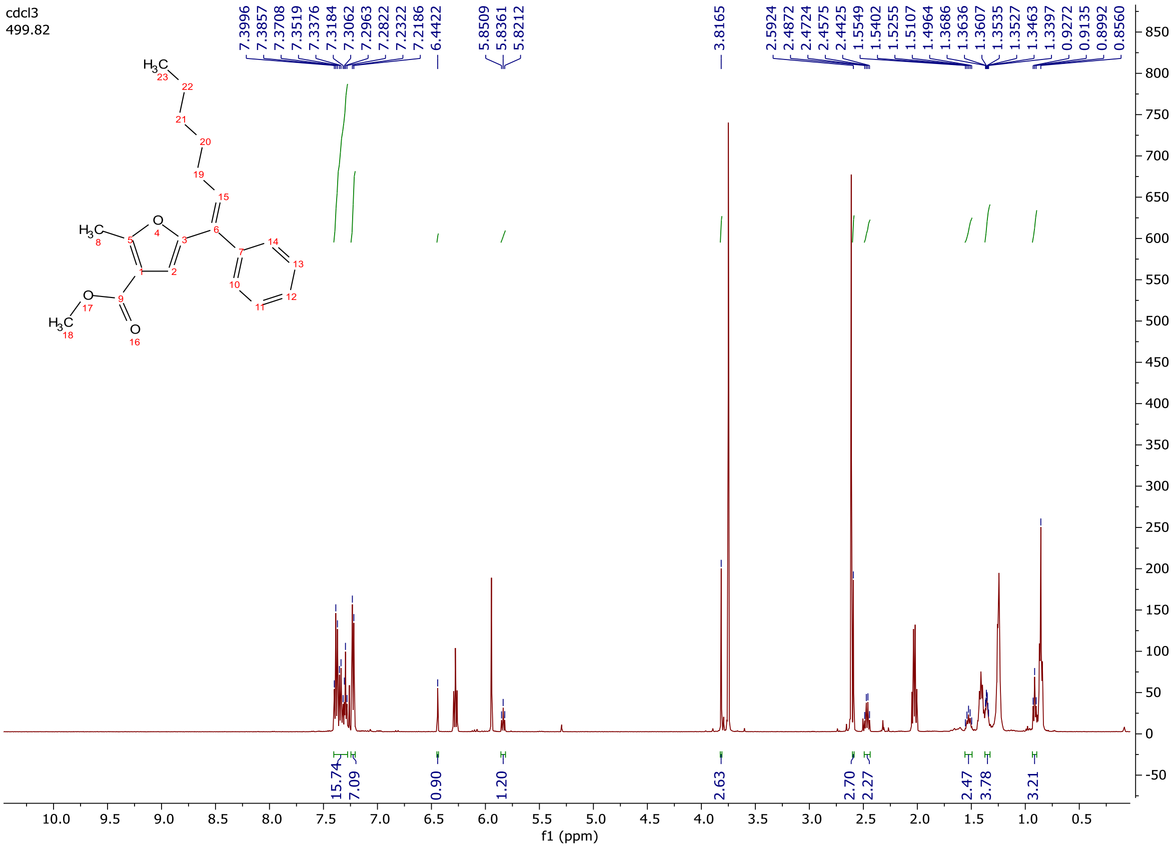

^{13}C{^{1}H} NMR (126 MHz, $CDCl_3$) of compound **22b**

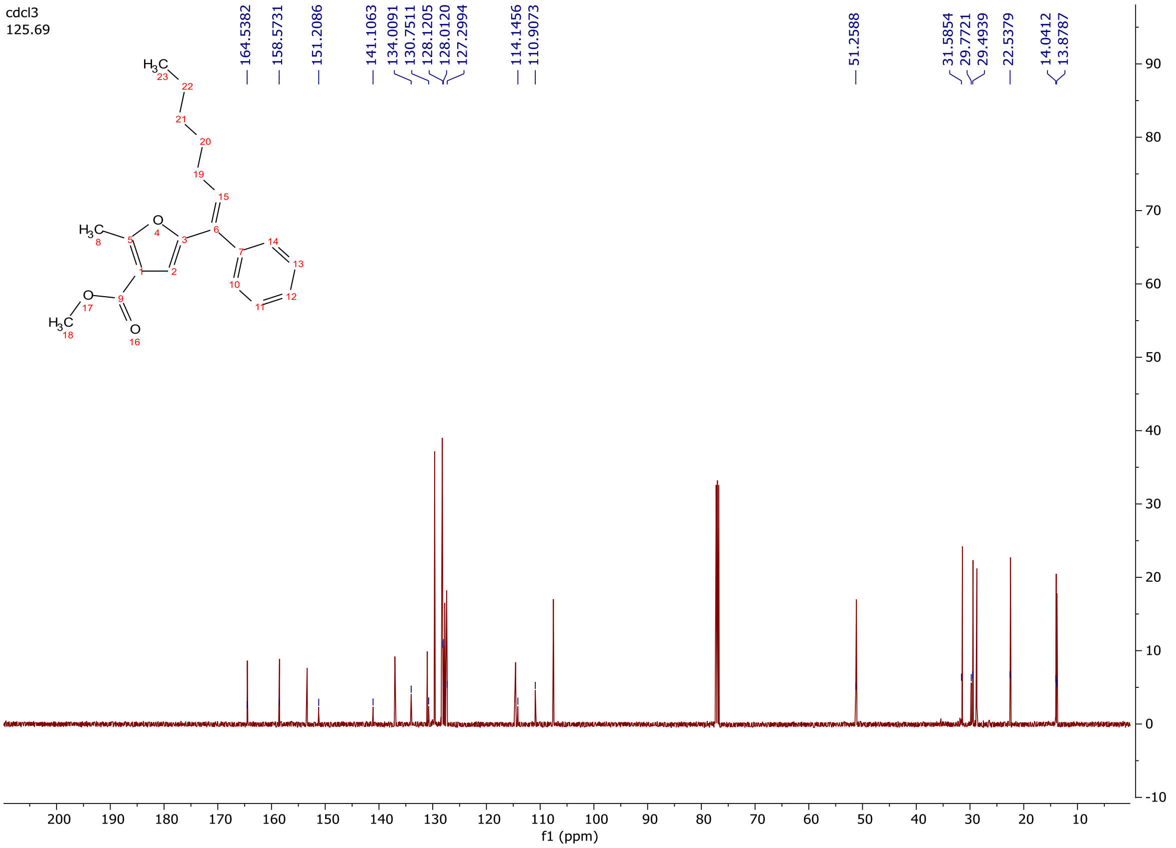

^{1}H NMR (400 MHz, $CDCl_3$) of compound **23**

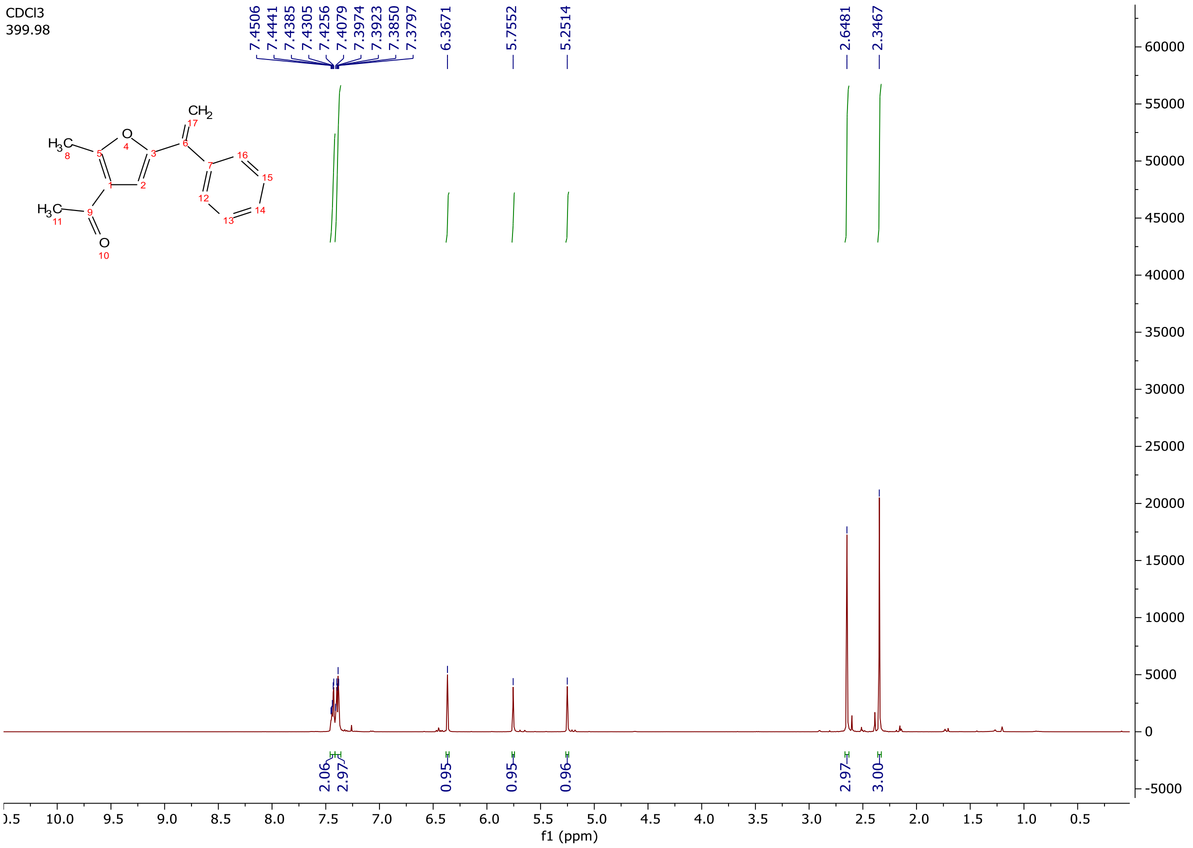

^{13}C{^{1}H} NMR (101 MHz, $CDCl_3$) of compound **23**

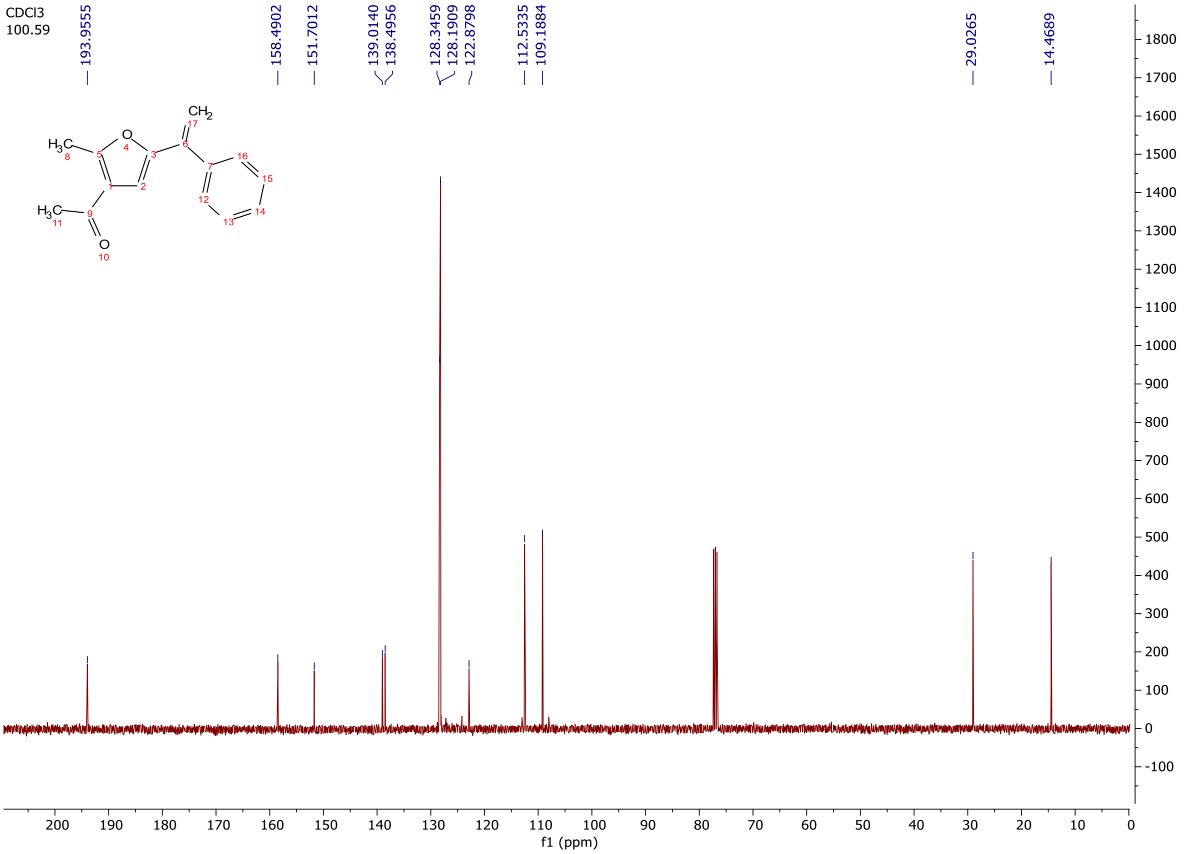

^{1}H NMR (400 MHz, $CDCl_3$) of compound **24**

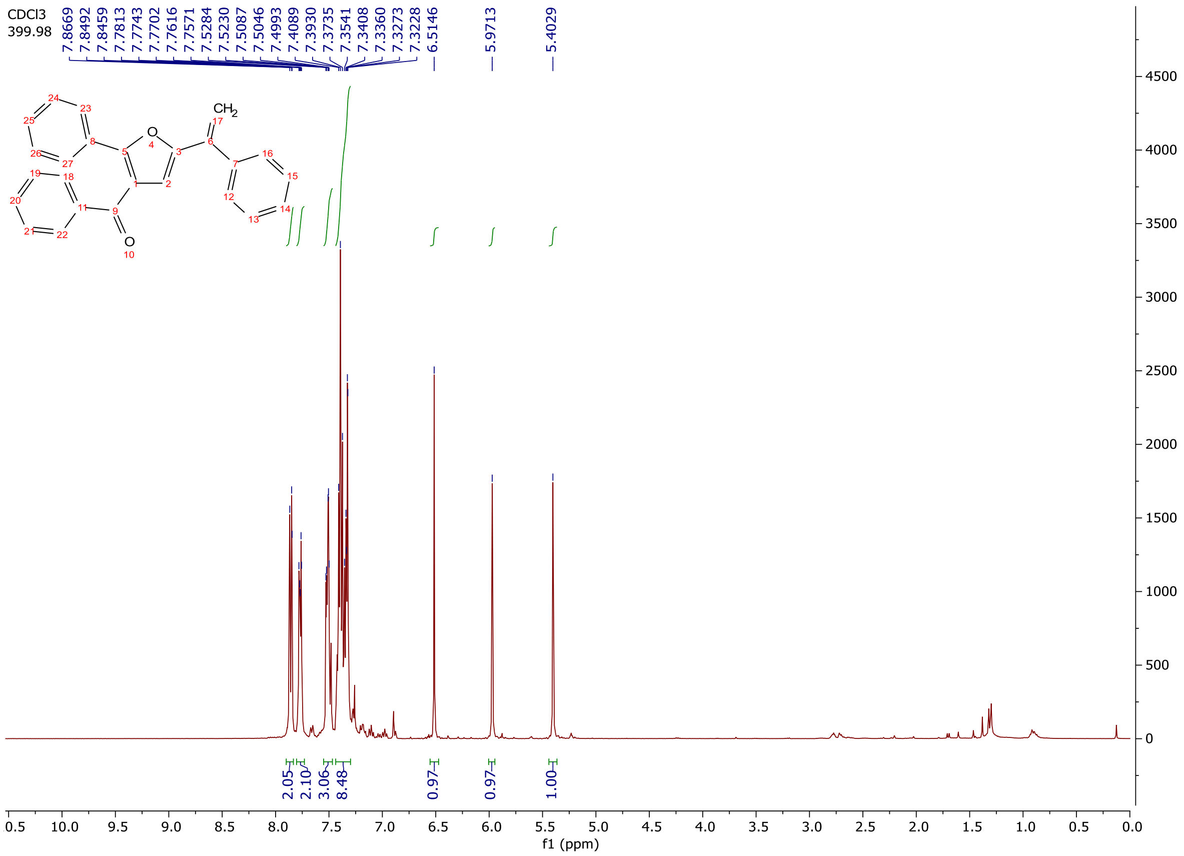

^{13}C{^{1}H} NMR (101 MHz, $CDCl_3$) of compound **24**

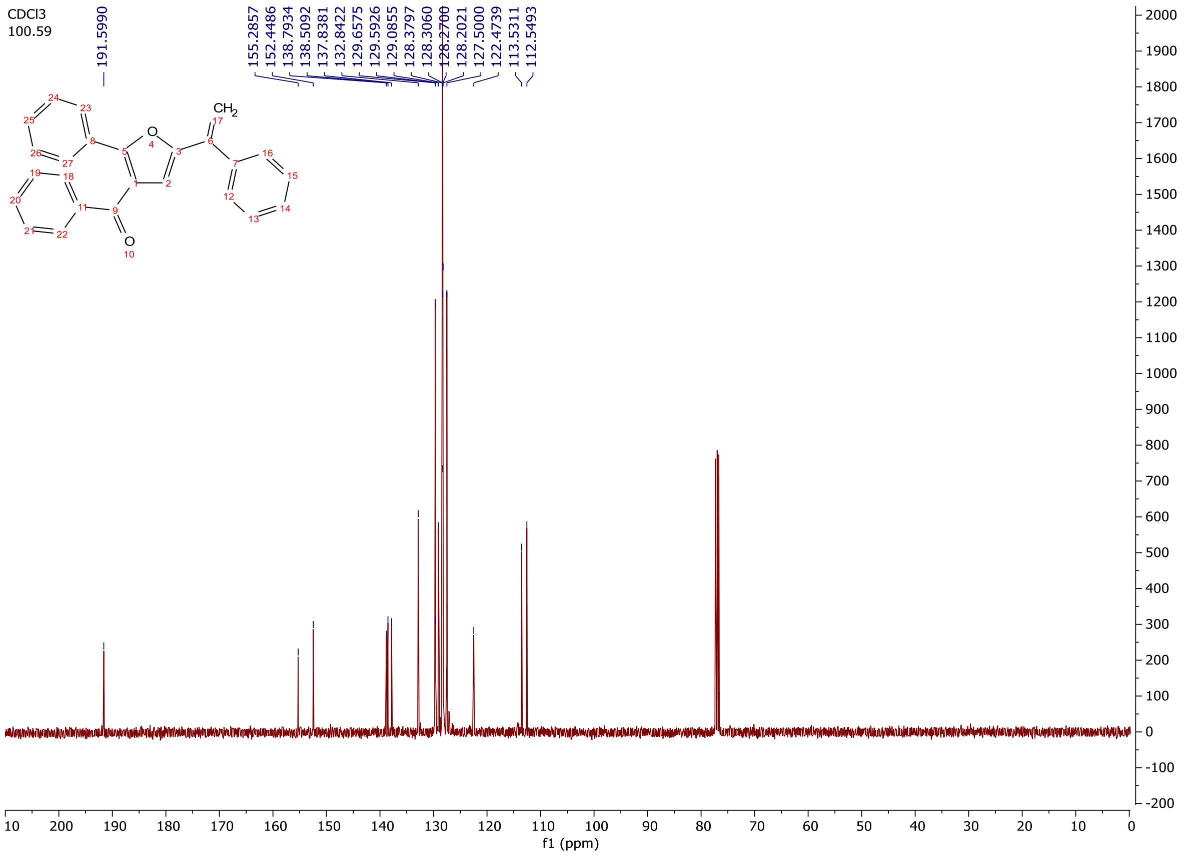

^{1}H NMR (500 MHz, $CDCl_3$) of compound **25**

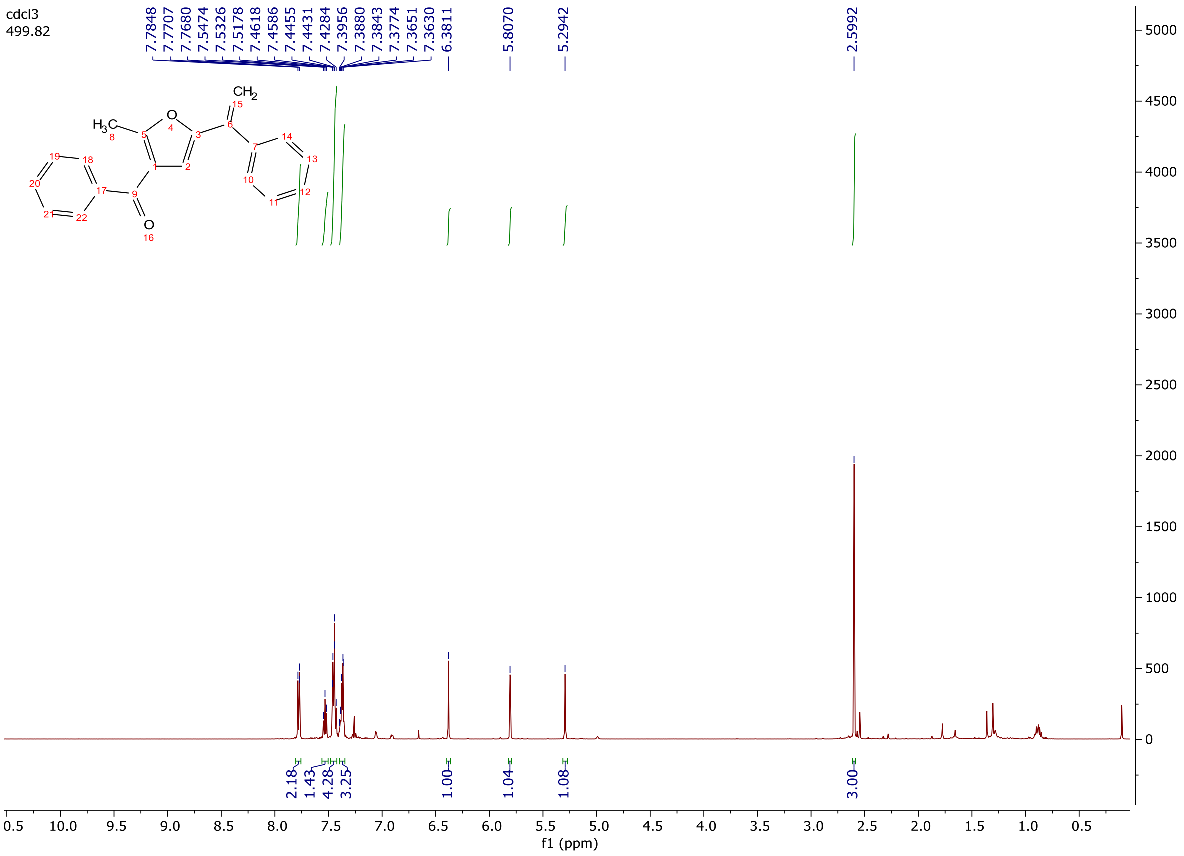

^{13}C{^{1}H} NMR (126 MHz, $CDCl_3$) of compound **25**

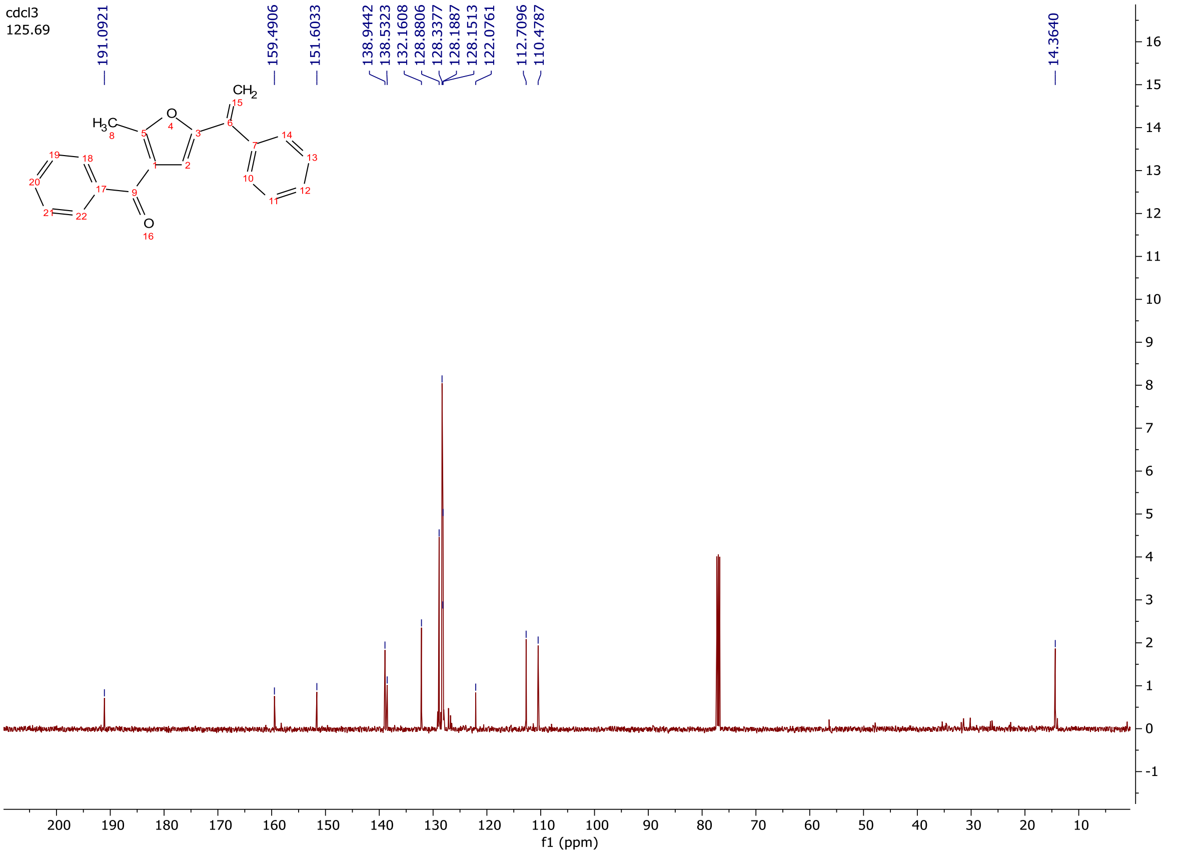

^{1}H NMR (400 MHz, $CDCl_3$) of compound **26**

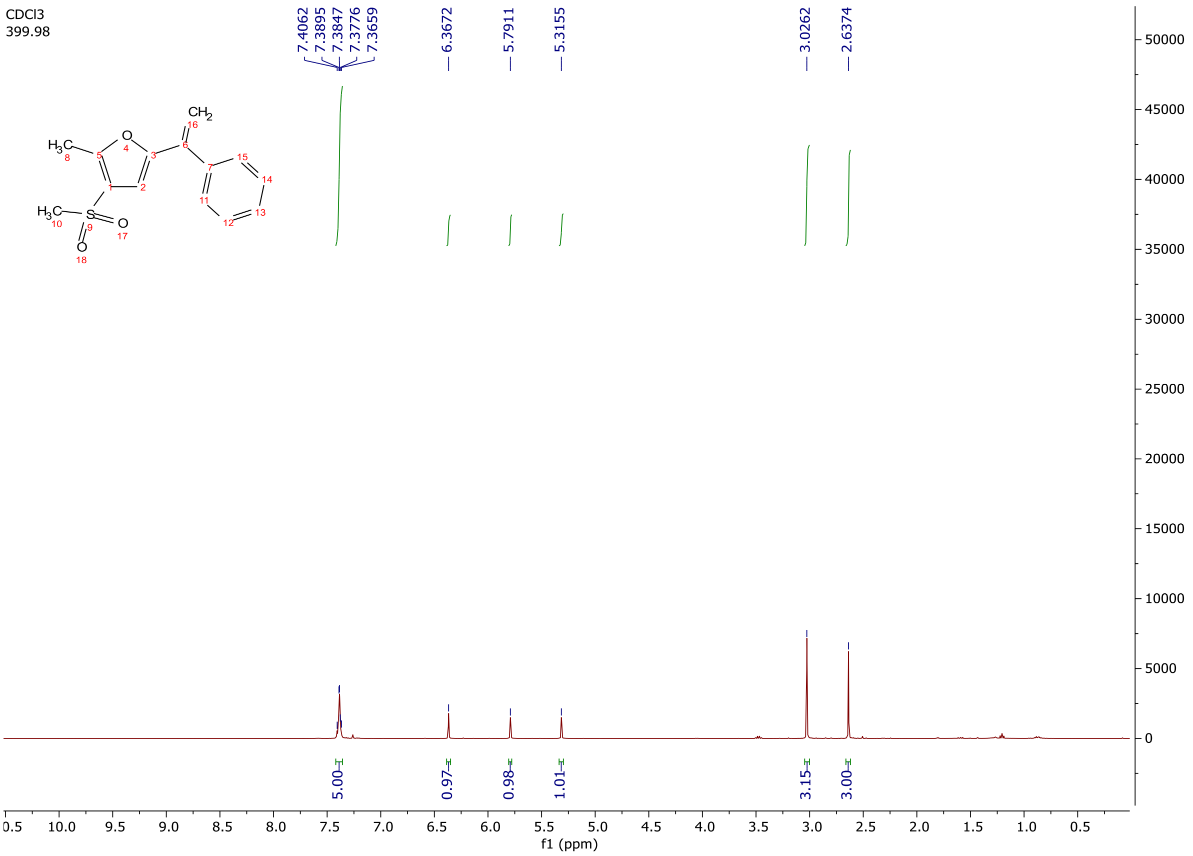

^{13}C{^{1}H} NMR (101 MHz, $CDCl_3$) of compound **26**

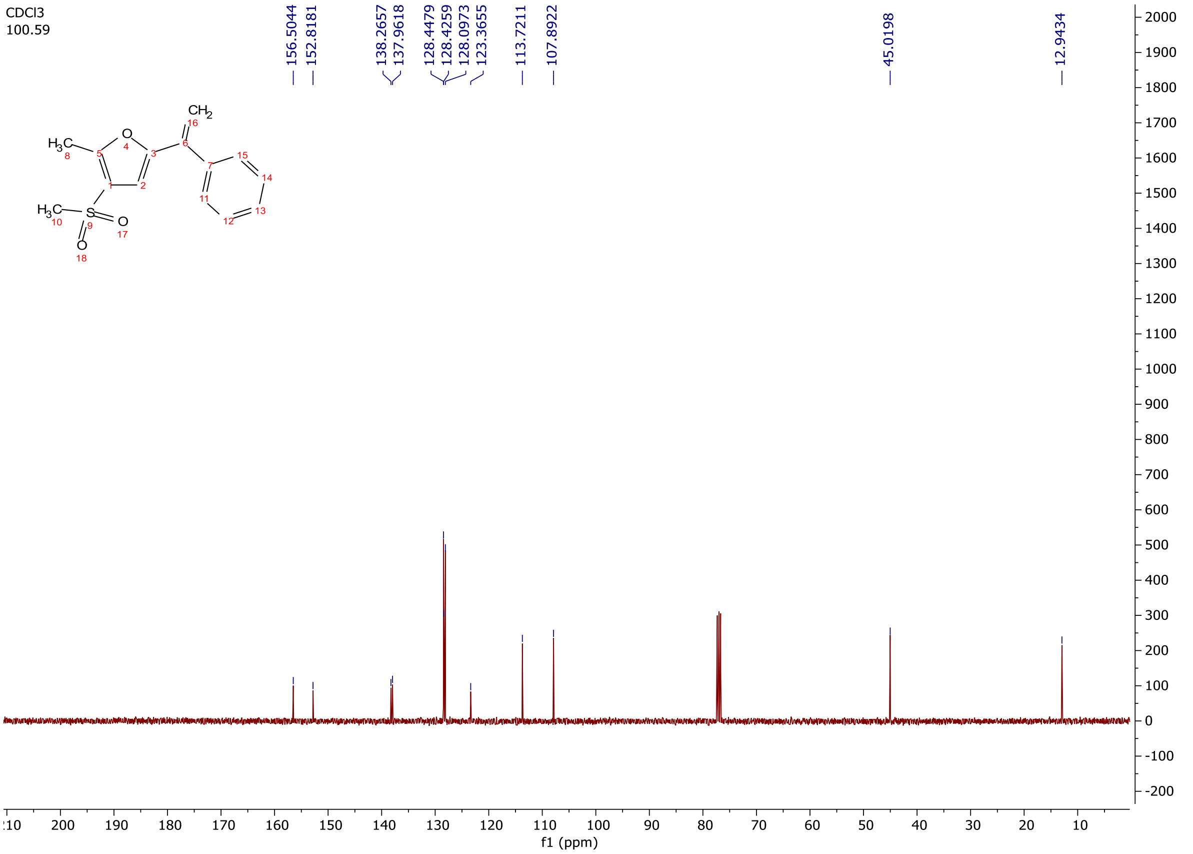

^{1}H NMR (400 MHz, $CDCl_3$) of compound **27**

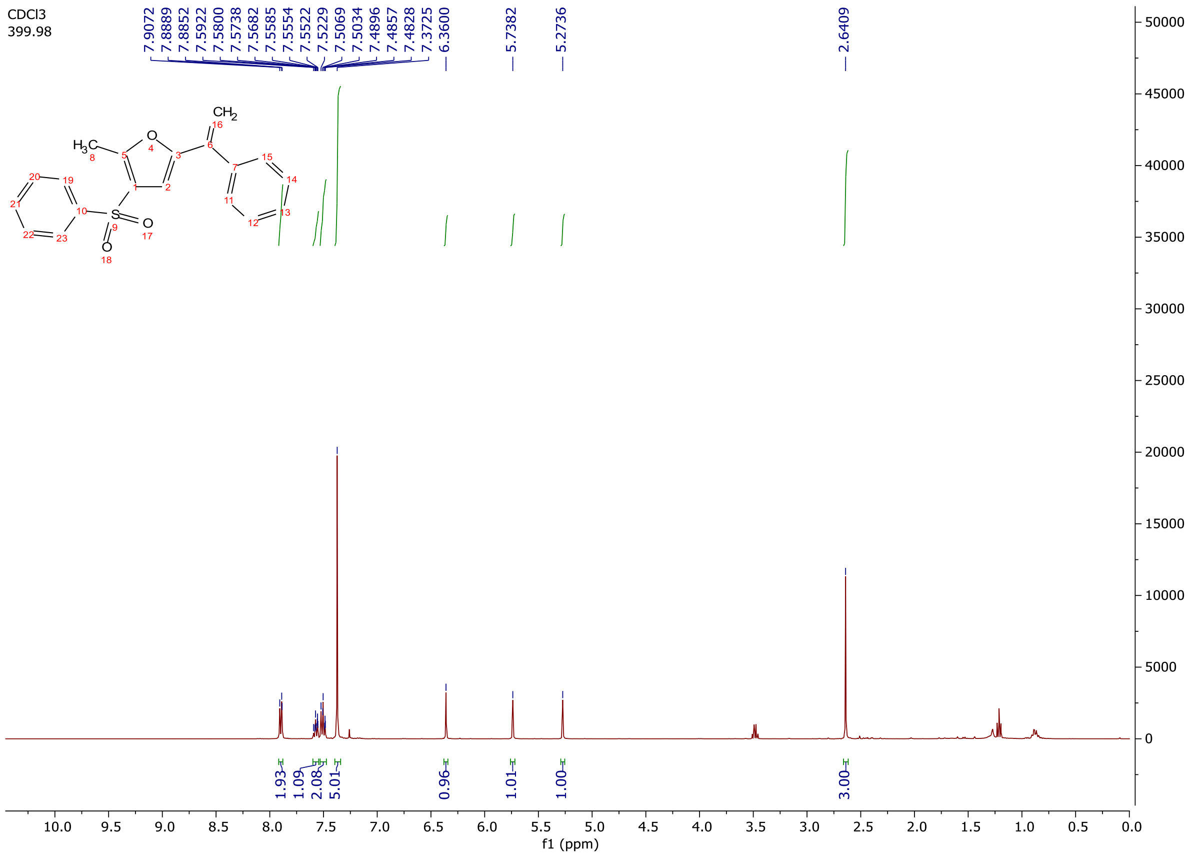

$^{13}C\{^{1}H\}$ NMR (101 MHz, $CDCl_3$) of compound **27**

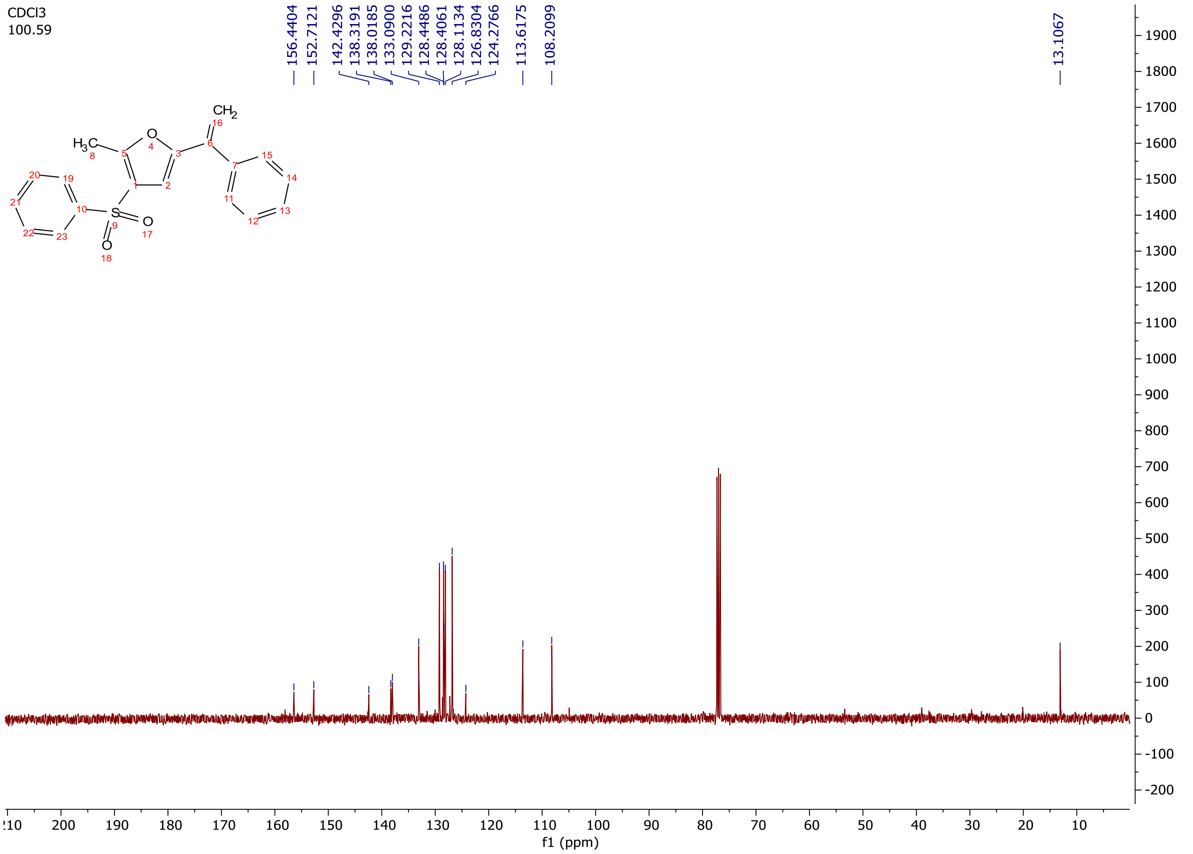

^{1}H NMR (600 MHz, $CDCl_3$) of compound **28**

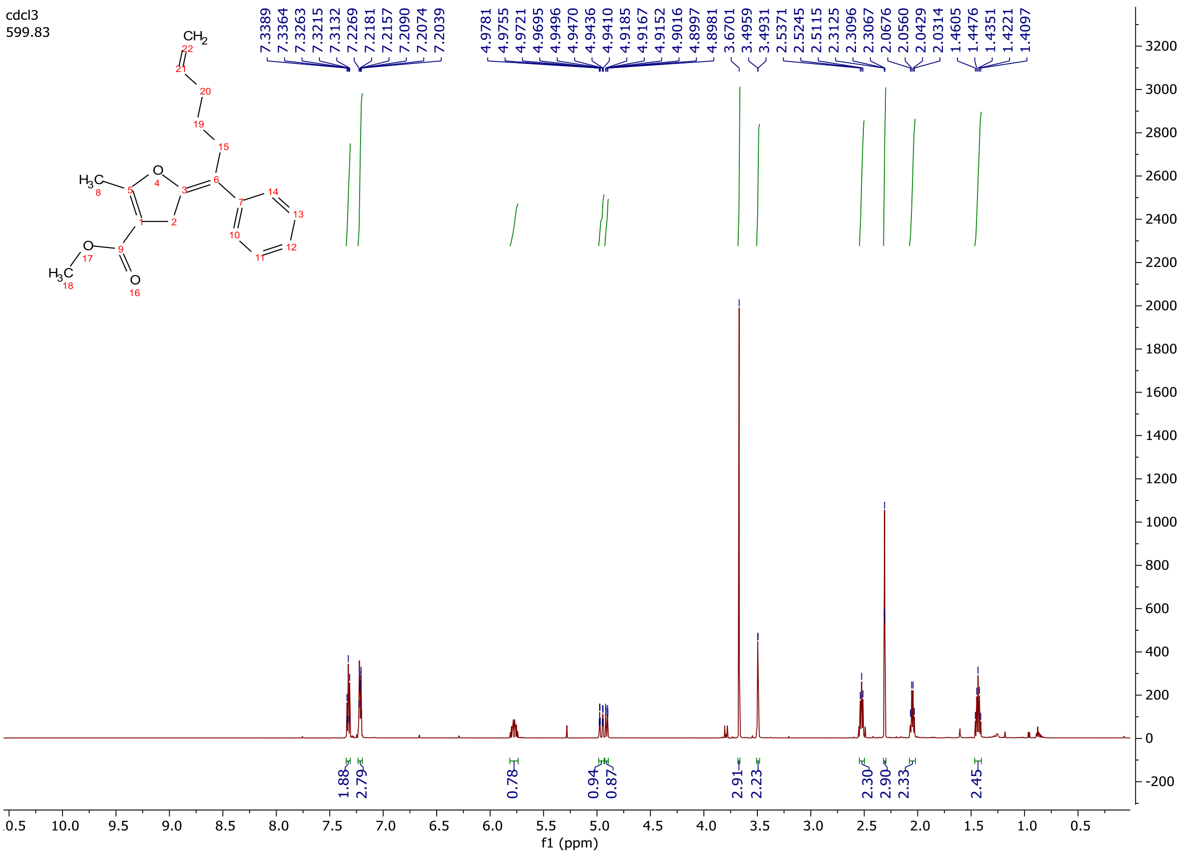

^{13}C{^{1}H} NMR (151 MHz, $CDCl_3$) of compound **28**

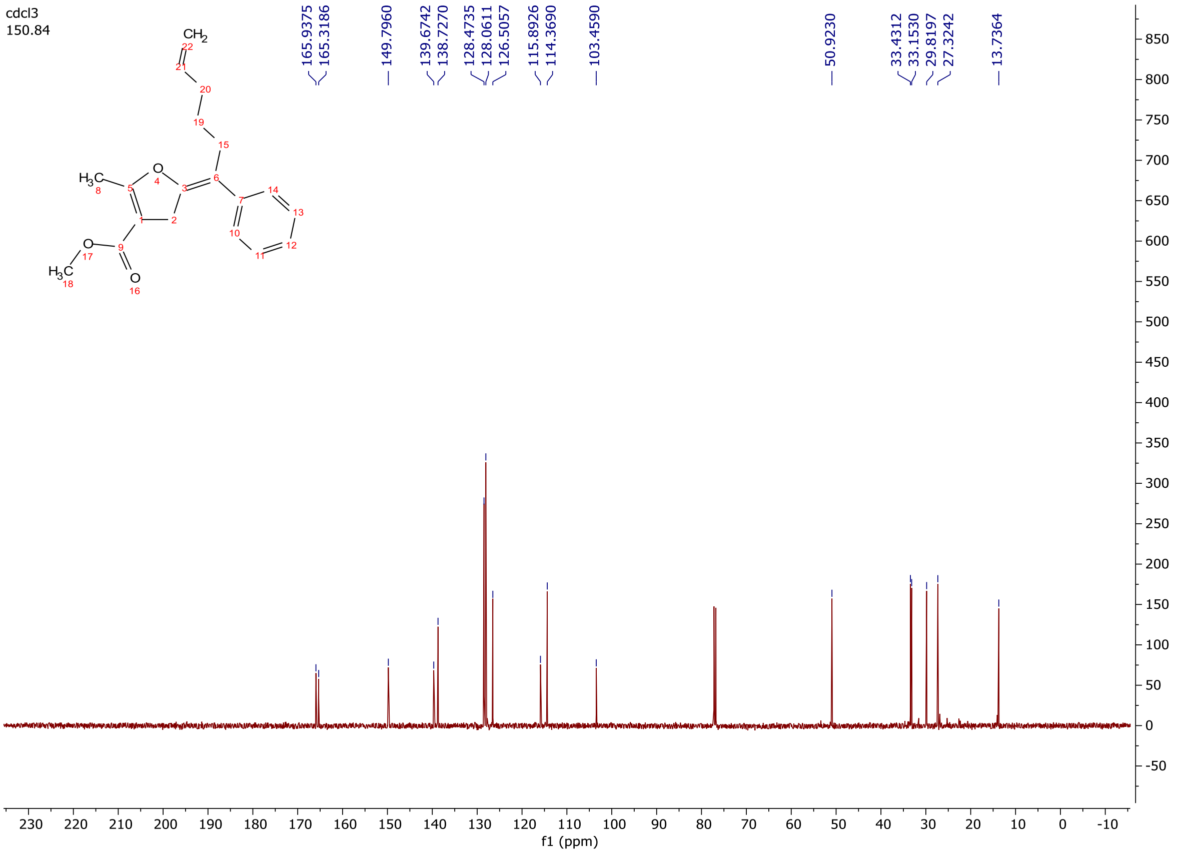

^{1}H NMR (500 MHz, $CDCl_3$) of compound **29a**

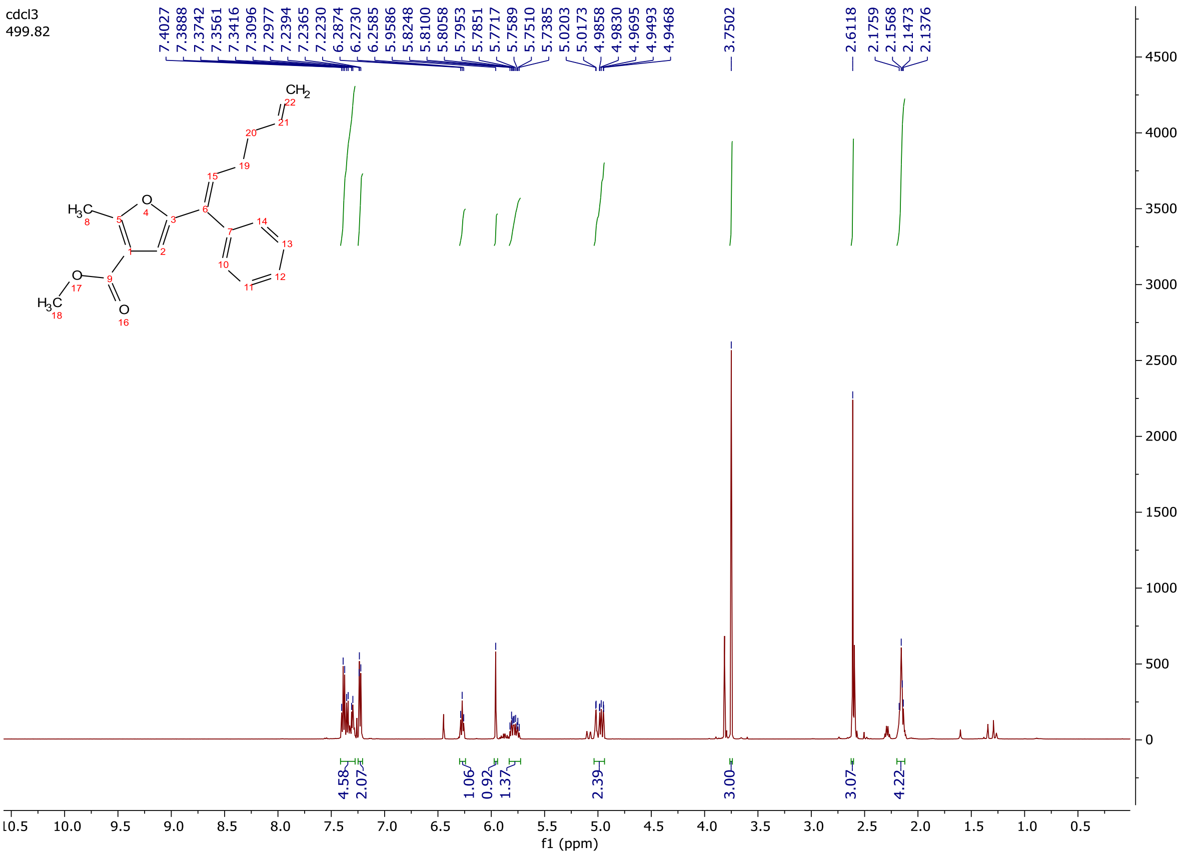

^{13}C{^{1}H} NMR (126 MHz, $CDCl_3$) of compound **29a**

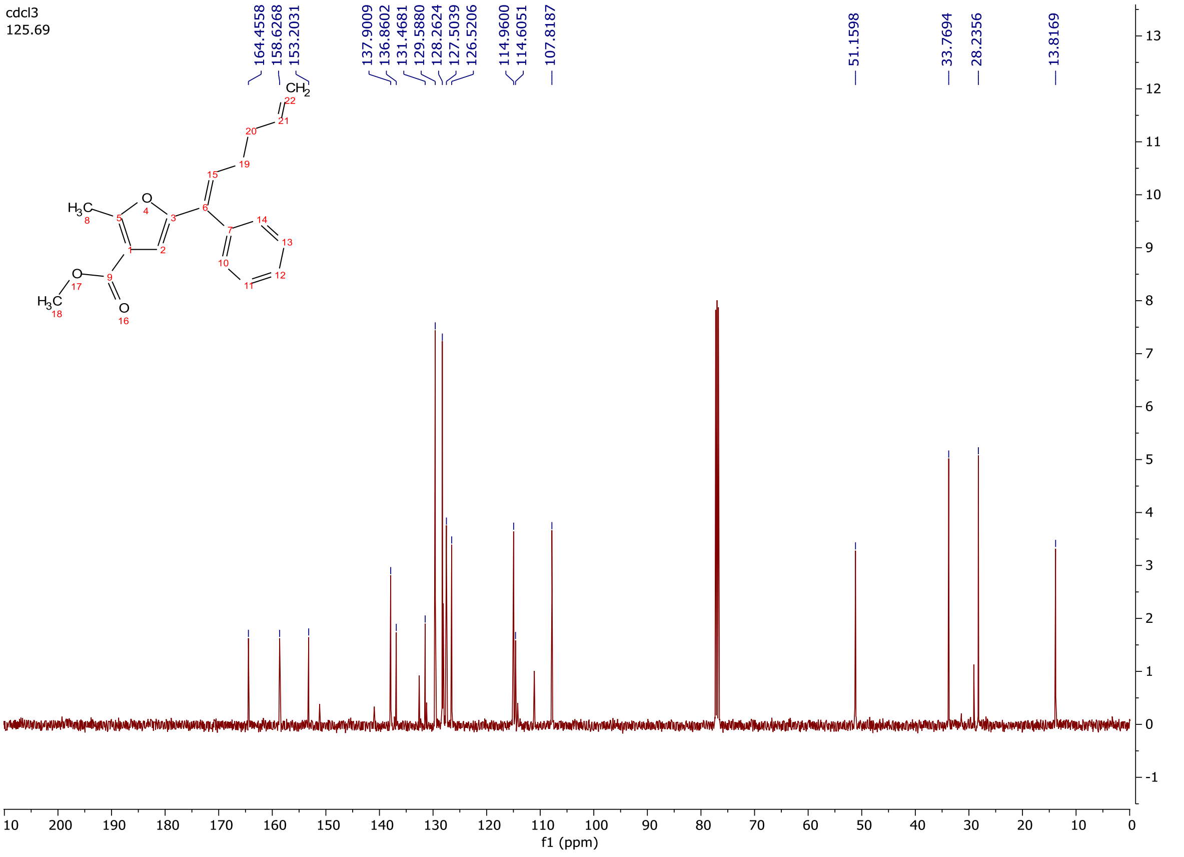

^{1}H NMR (500 MHz, $CDCl_3$) of compound **29b**

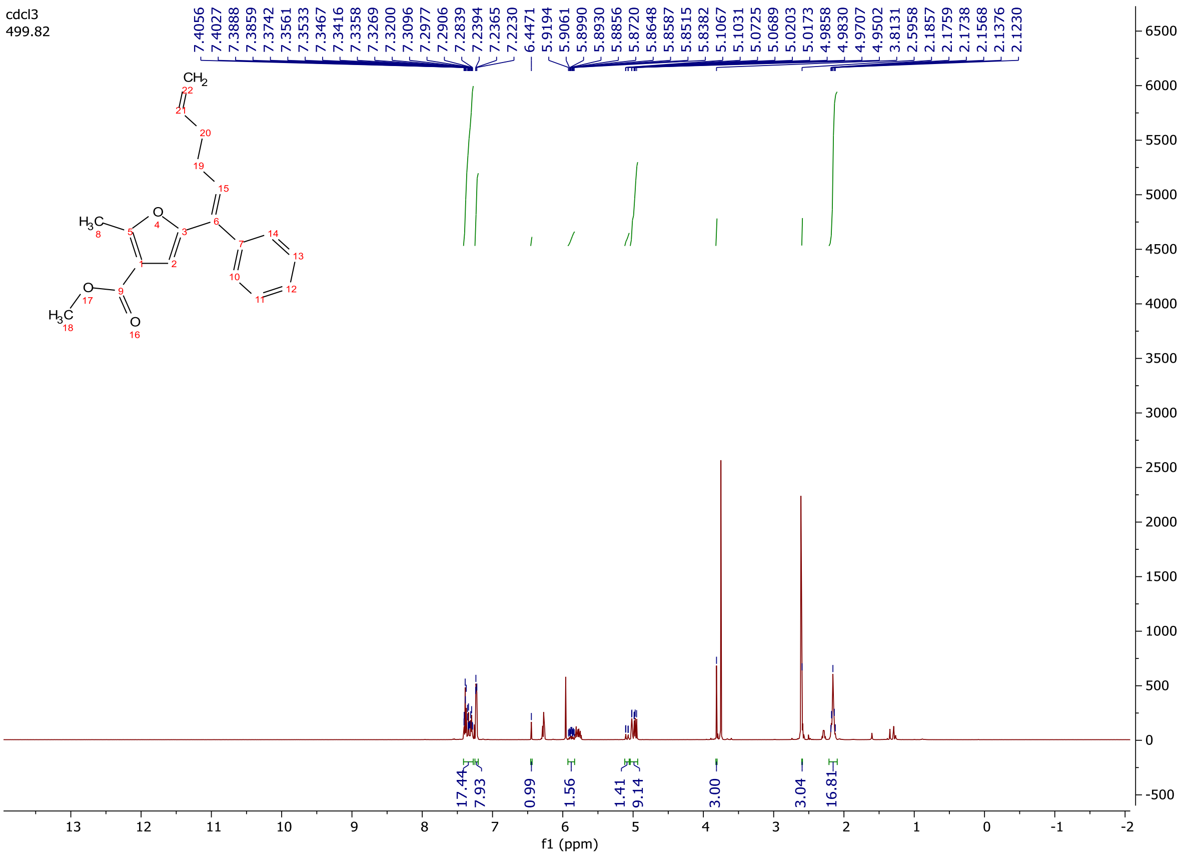

$^{13}C\{^{1}H\}$ NMR (126 MHz, $CDCl_3$) of compound **29b**

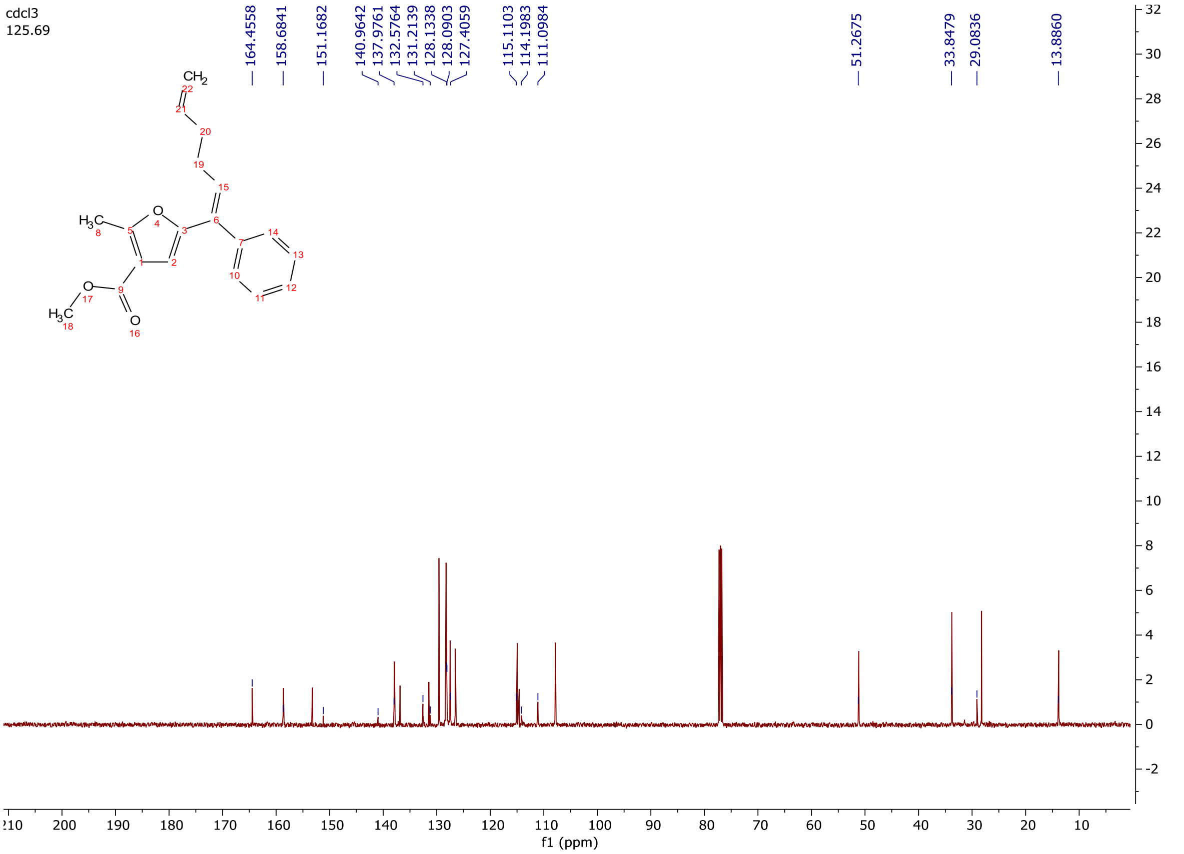

^{1}H NMR (500 MHz, $CDCl_3$) of compound **31**

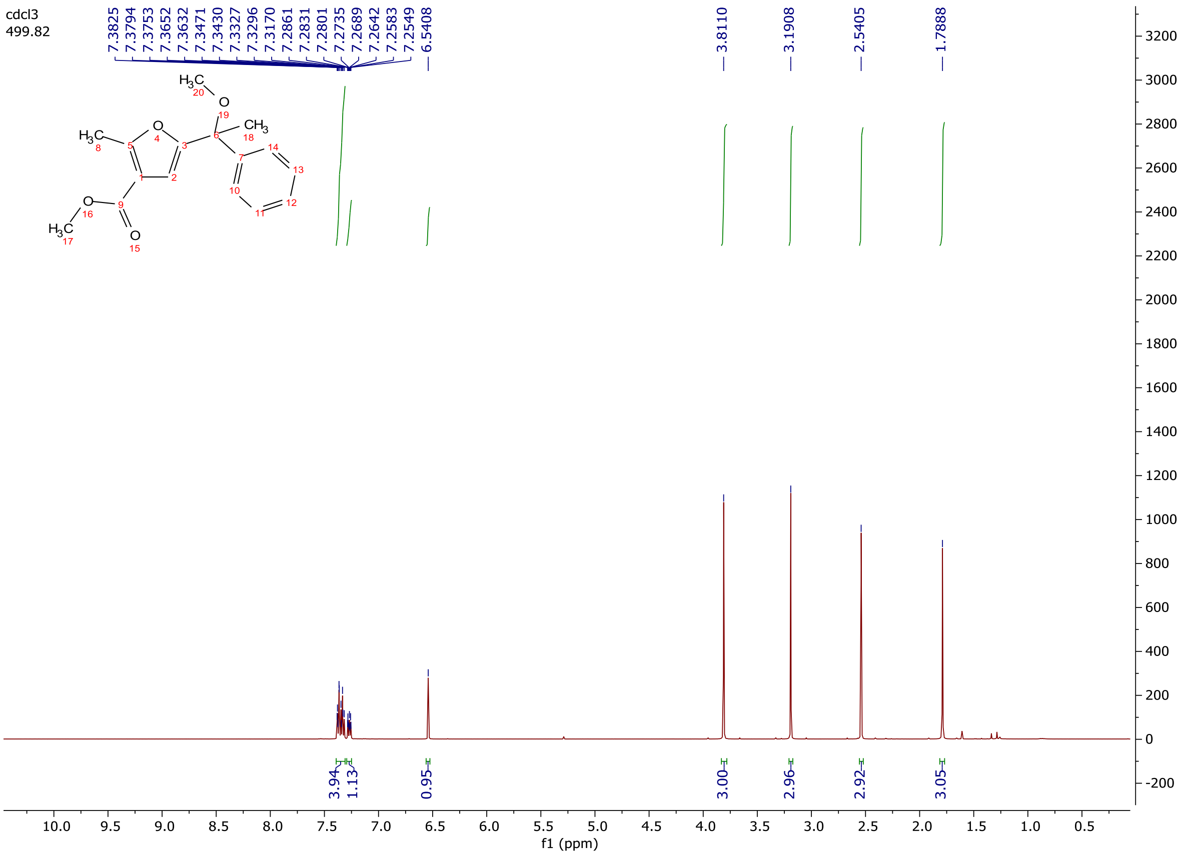

^{13}C{^{1}H} NMR (126 MHz, $CDCl_3$) of compound **31**

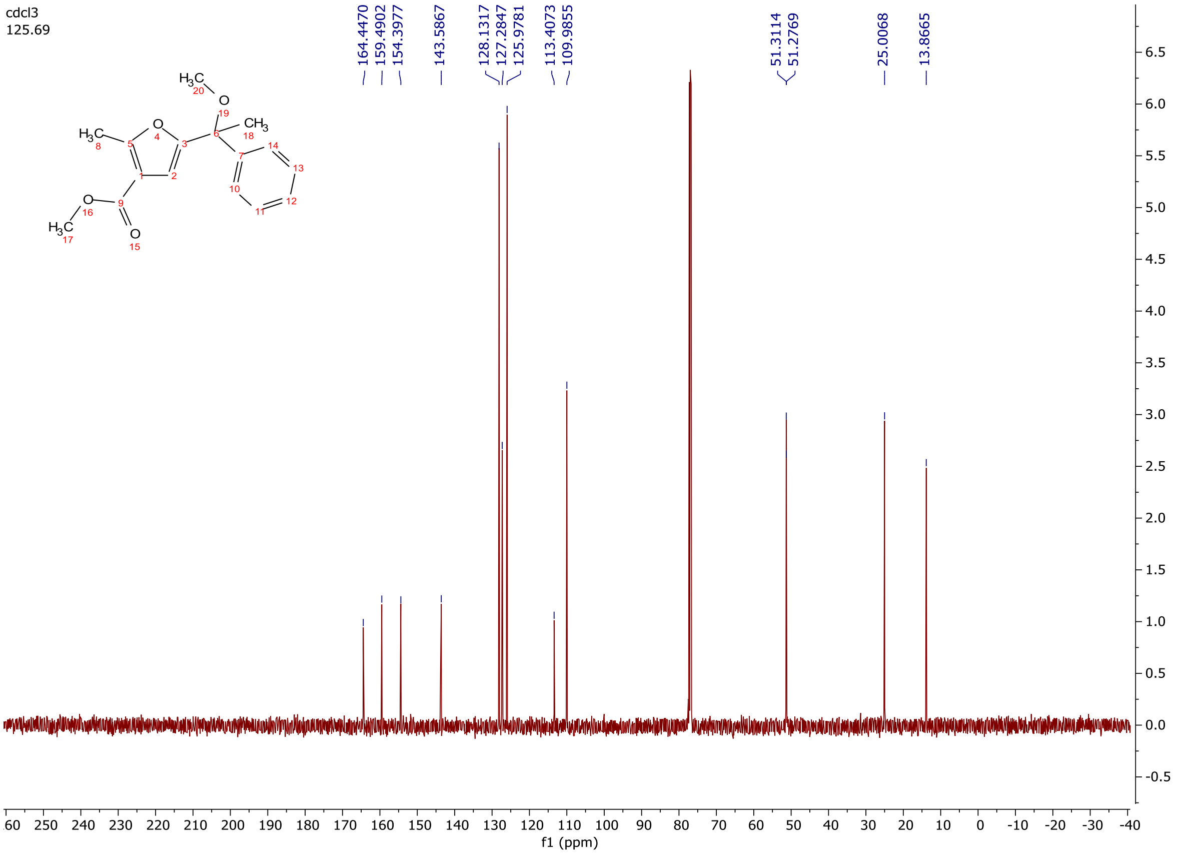

^{1}H NMR (500 MHz, CDCl$_3$) of compound **32**

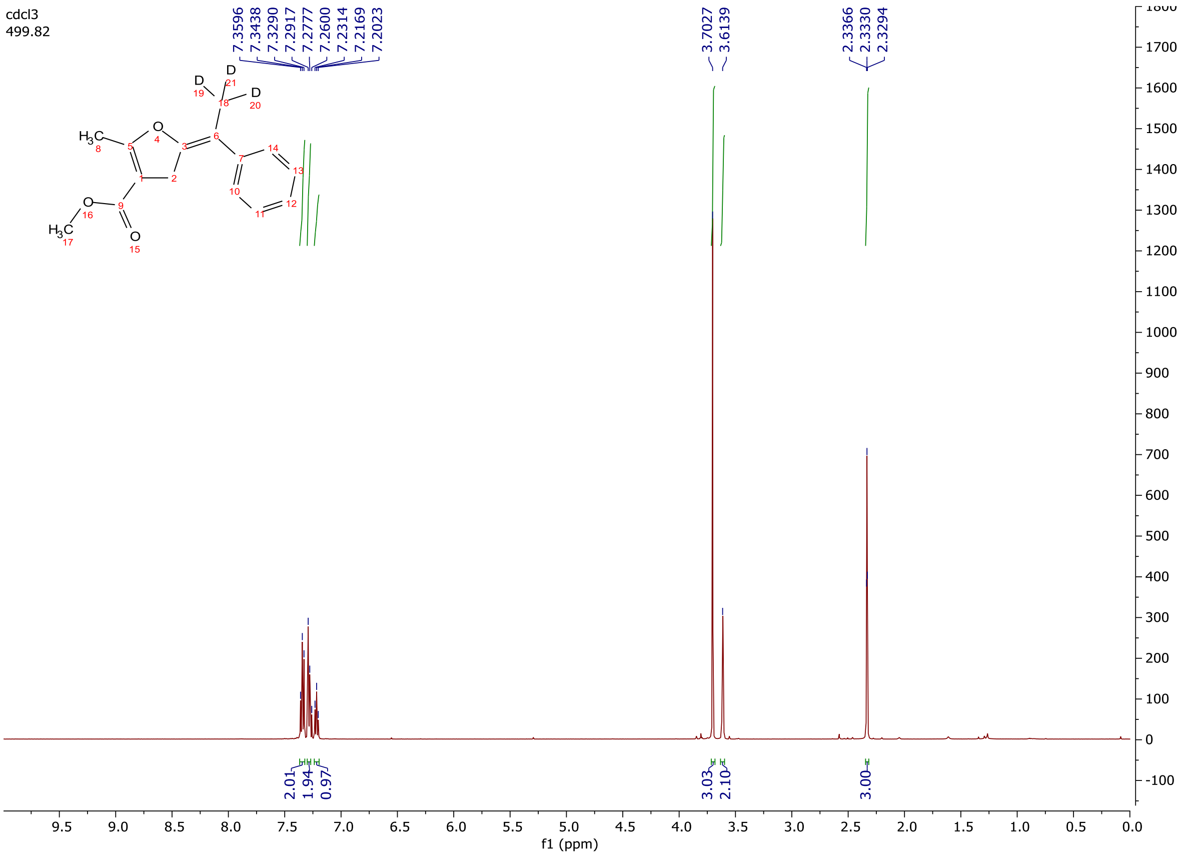

^{13}C{^{1}H} NMR (126 MHz, CDCl$_3$) of compound **32**

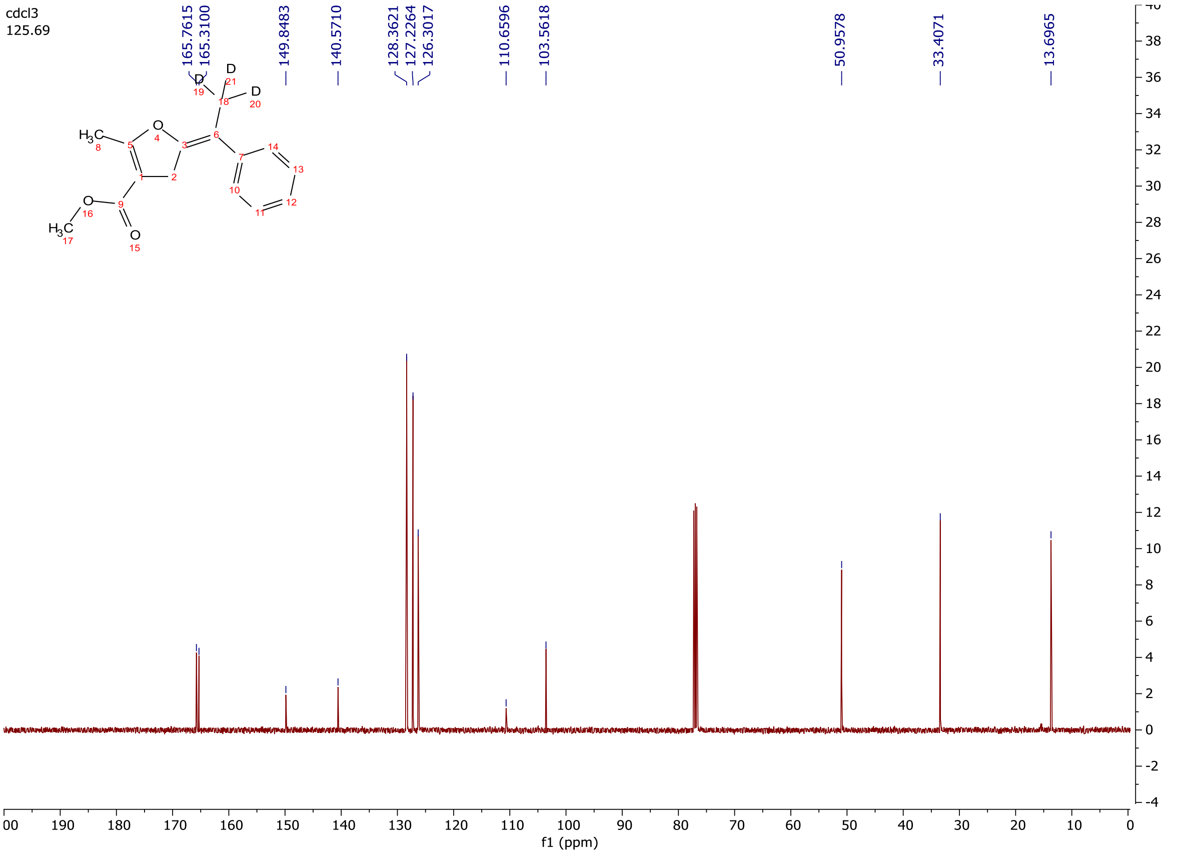

^{1}H NMR (400 MHz, $CDCl_3$) of compound **33**

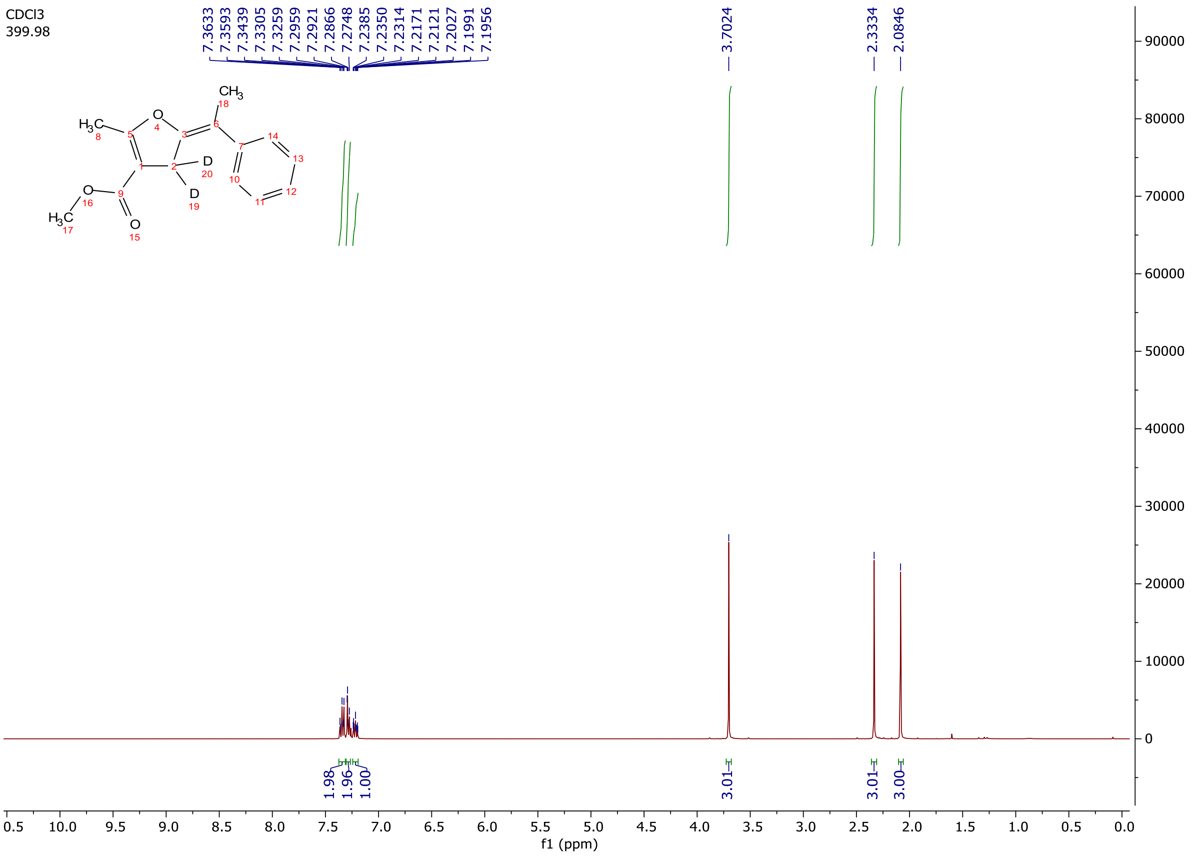

$^{13}C\{^{1}H\}$ NMR (101 MHz, $CDCl_3$) of compound **33**

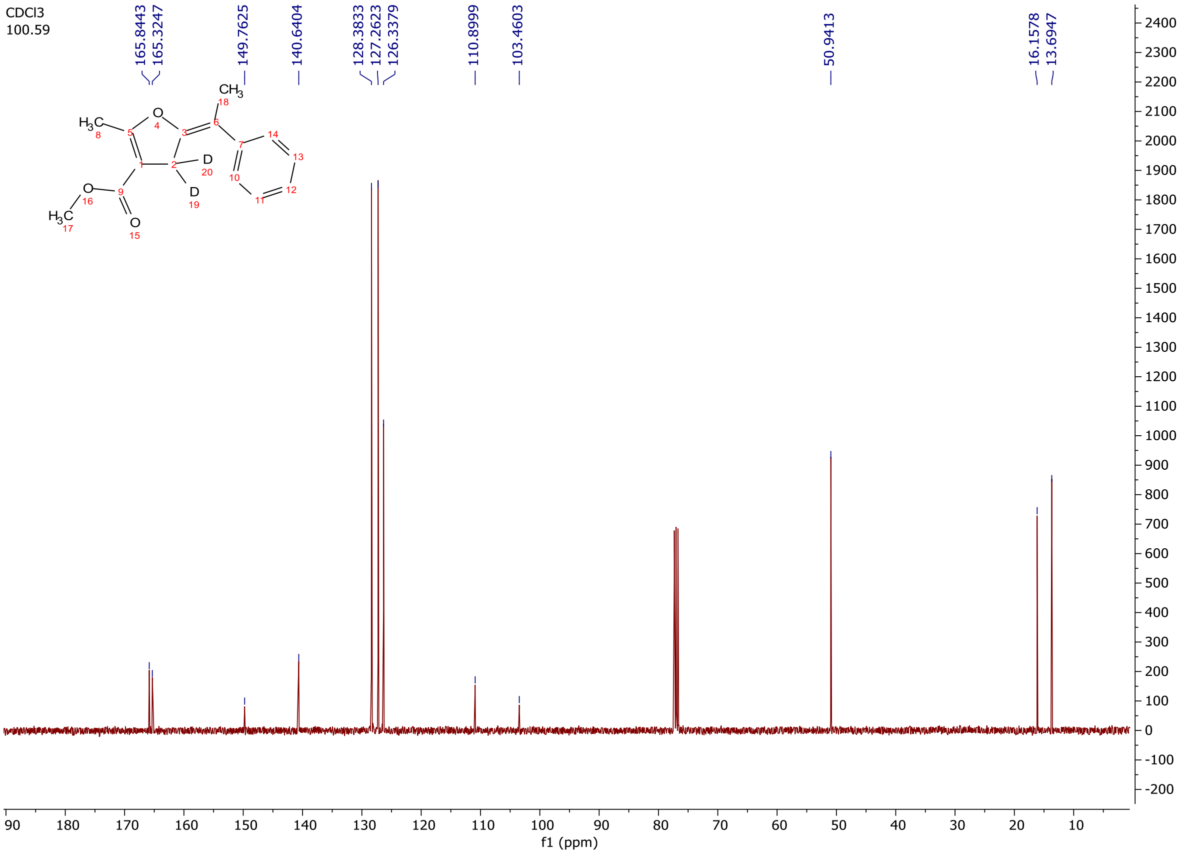

^{1}H NMR (500 MHz, $CDCl_3$) of compound **36**

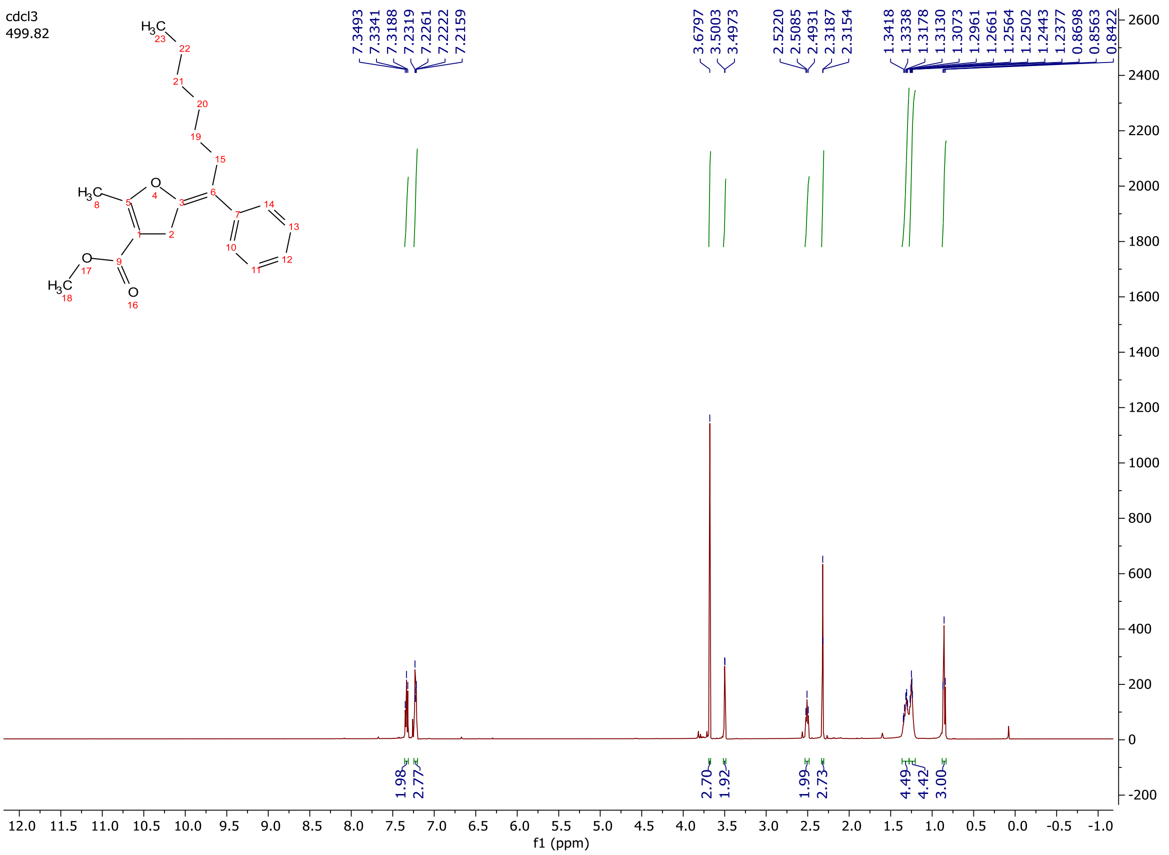

$^{13}C\{^{1}H\}$ NMR (126 MHz, $CDCl_3$) of compound **36**

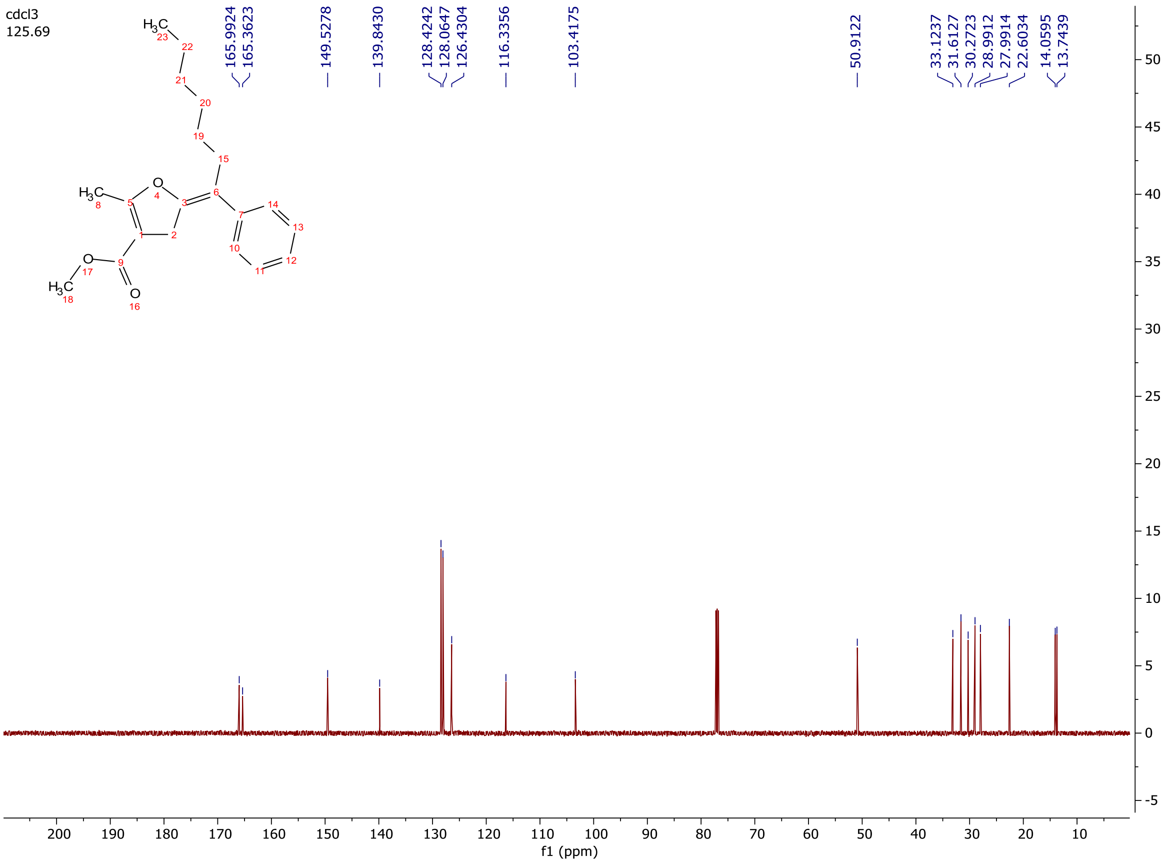

^{1}H NMR (500 MHz, $CDCl_3$) of methyl 2-acetylhex-4-ynoate-6,6,6-d3

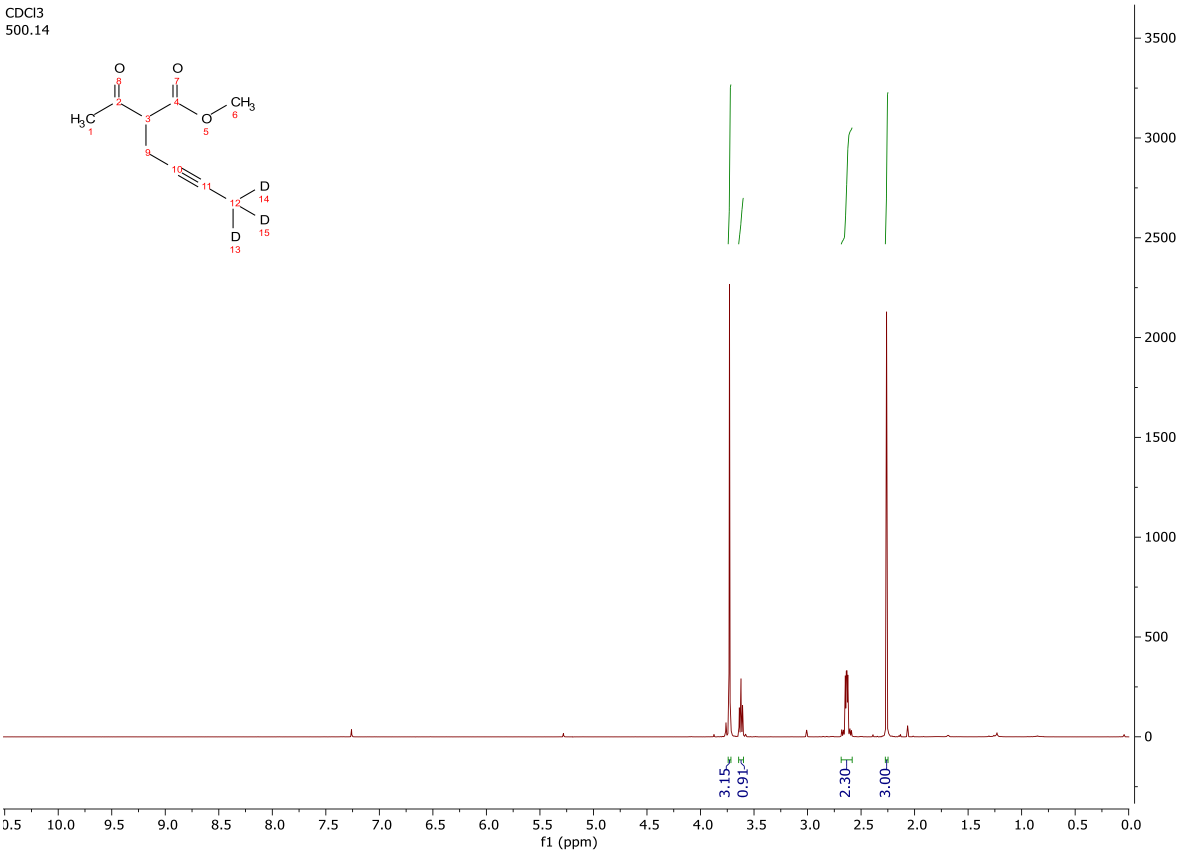

$^{13}C\{^{1}H\}$ NMR (126 MHz, $CDCl_3$) of methyl 2-acetylhex-4-ynoate-6,6,6-d3

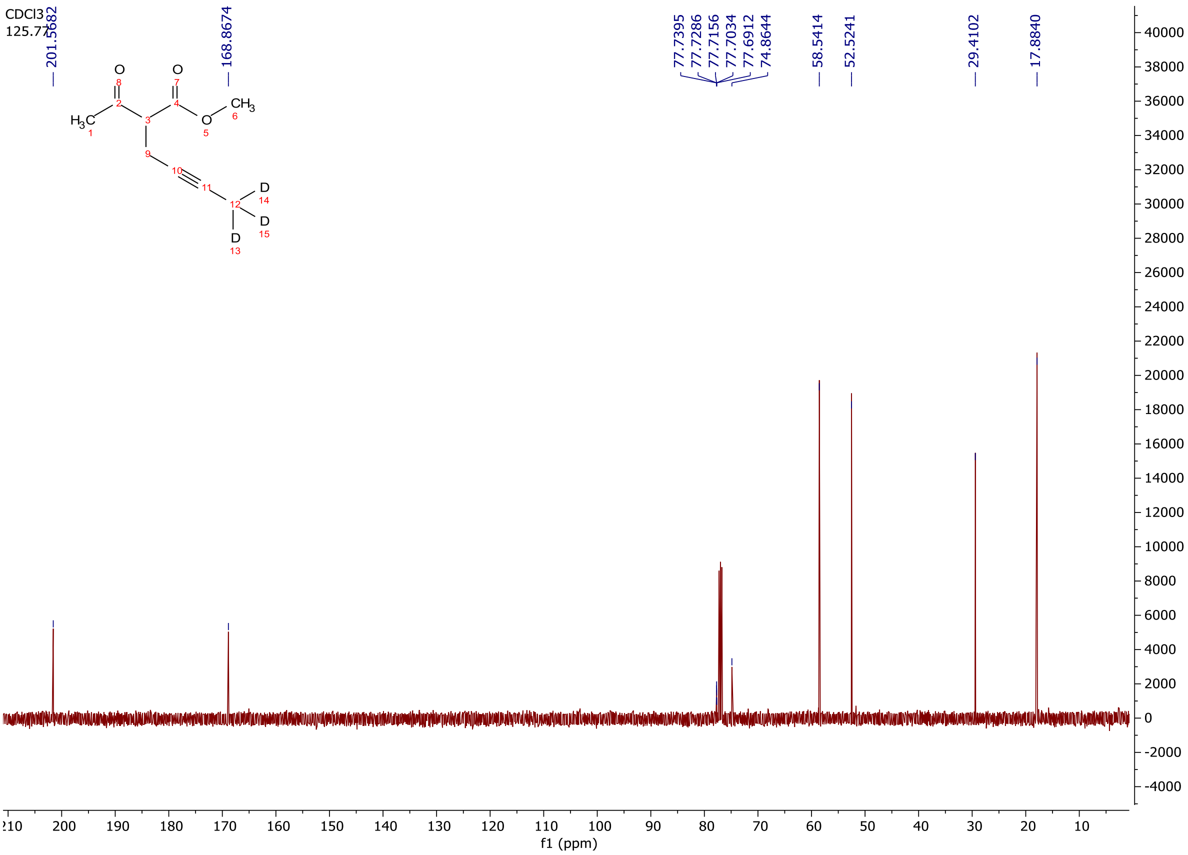

^{1}H NMR (400 MHz, $CDCl_3$) of methyl 2-acetylhex-4-ynoate-3,3-d2

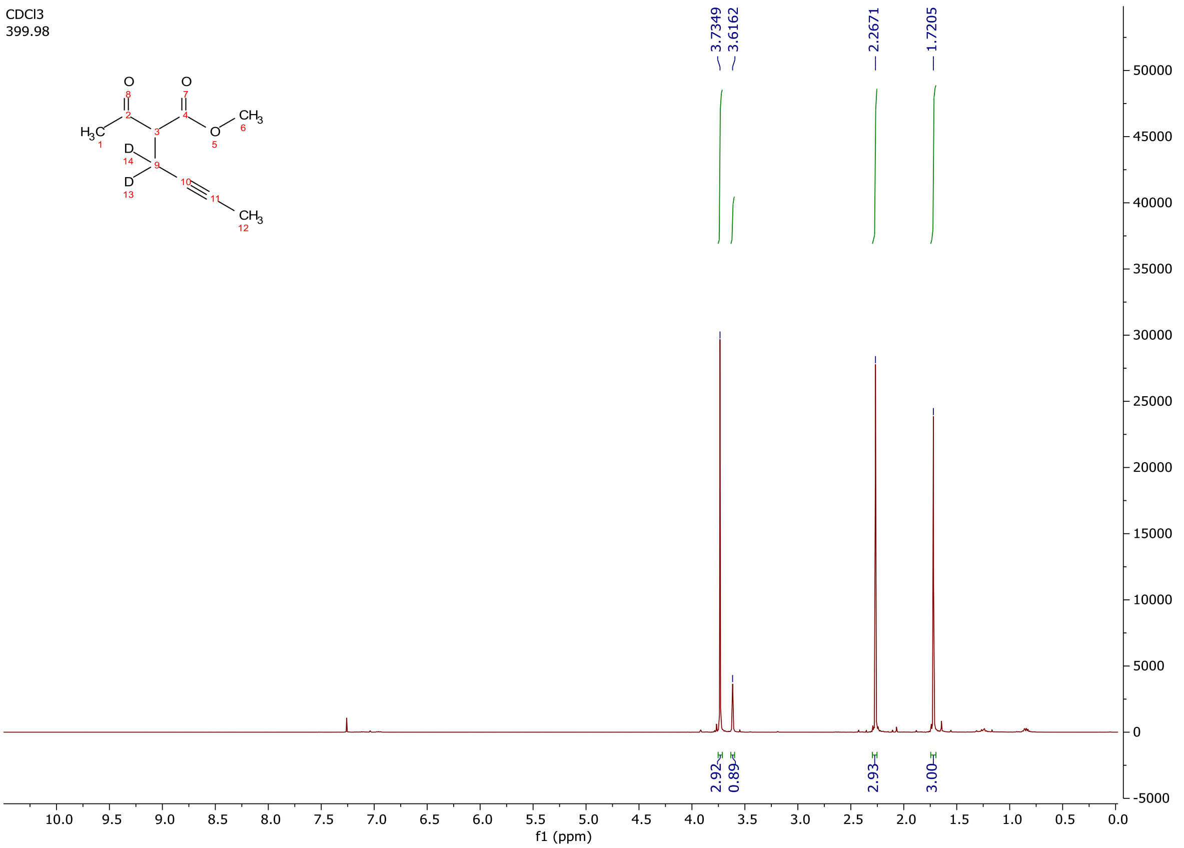

$^{13}C\{^{1}H\}$ NMR (101 MHz, $CDCl_3$) of methyl 2-acetylhex-4-ynoate-3,3-d2

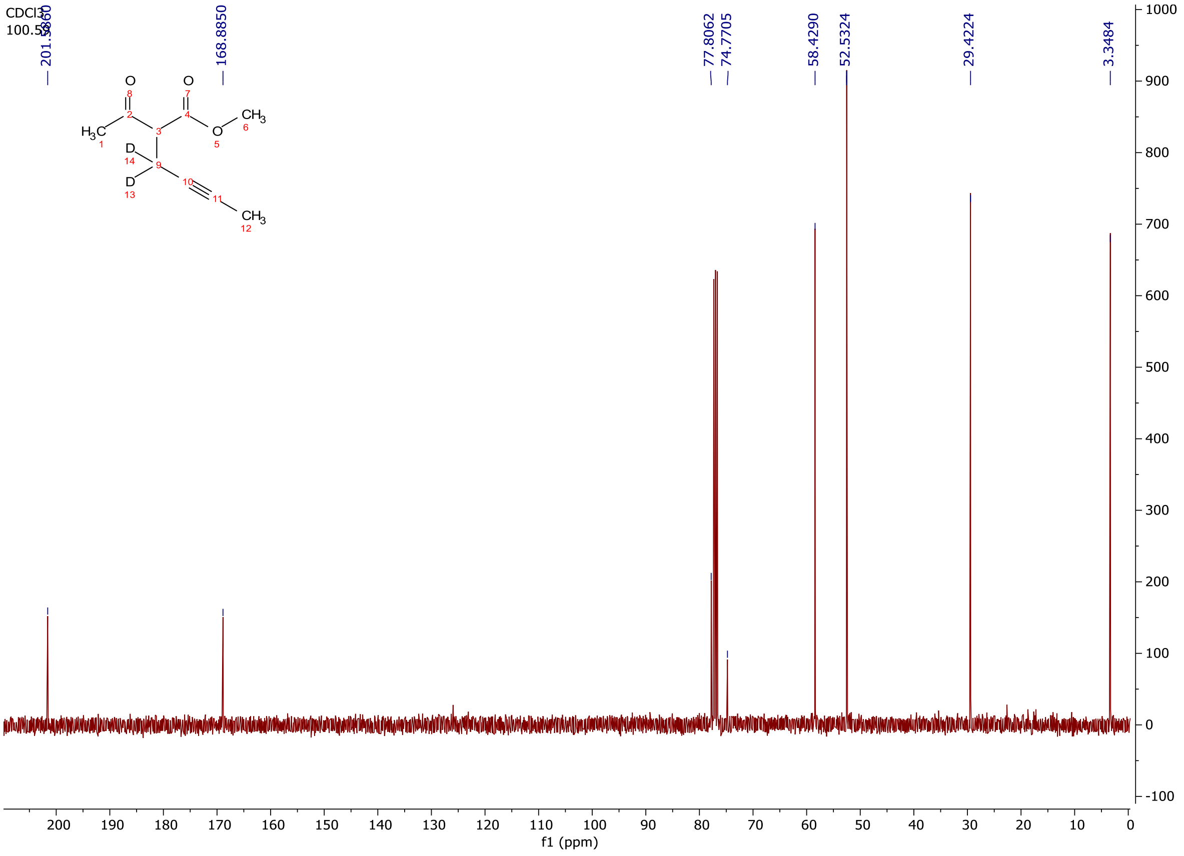

^{1}H NMR (600 MHz, $CDCl_3$) of methyl 2-acetylundec-4-ynoate

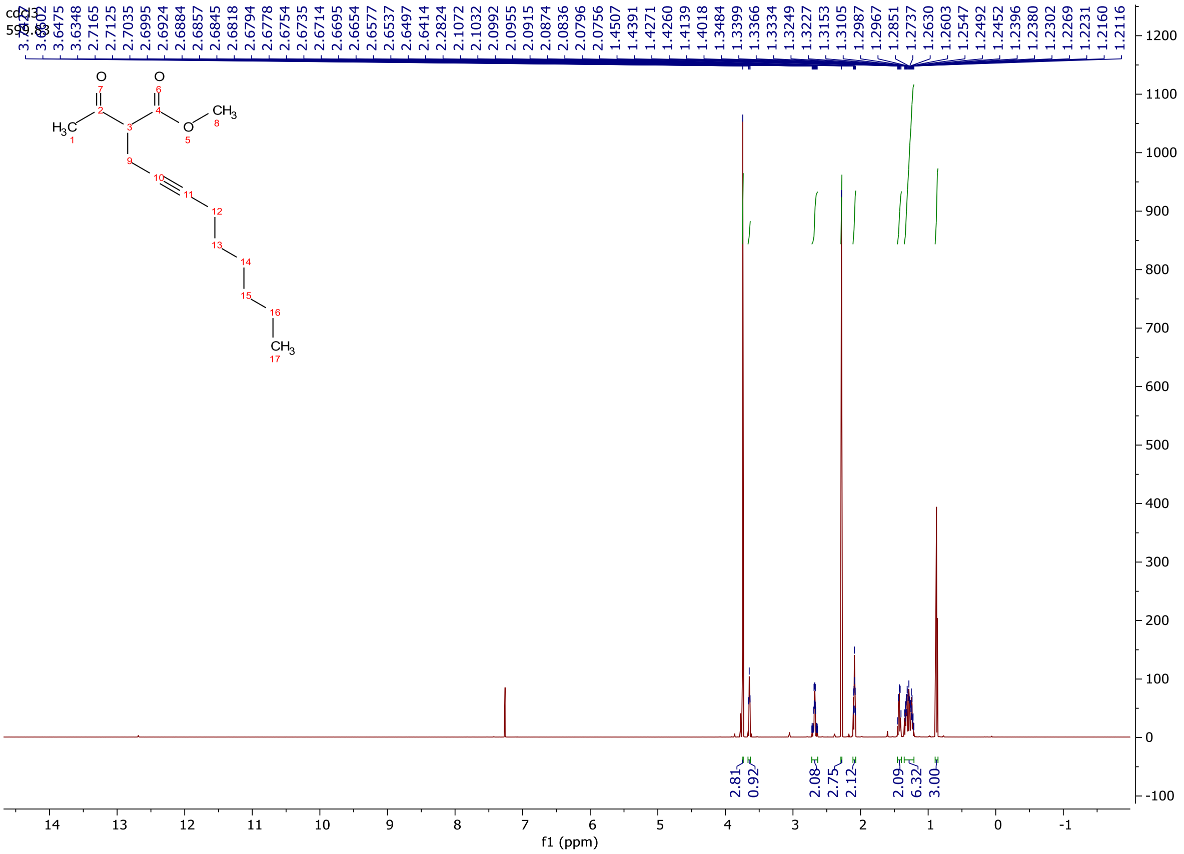

$^{13}C\{^{1}H\}$ NMR (151 MHz, $CDCl_3$) of methyl 2-acetylundec-4-ynoate

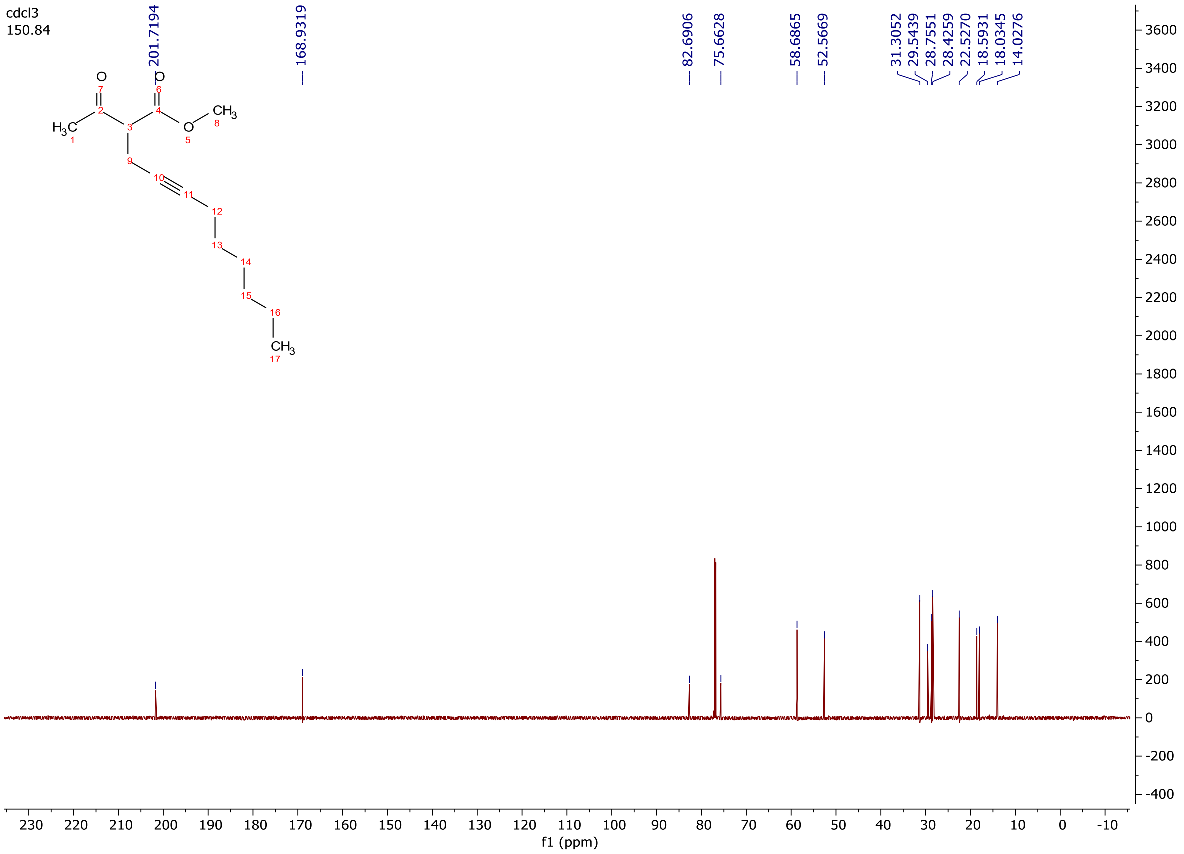

^{1}H NMR (500 MHz, $CDCl_3$) of methyl 2-acetyldec-9-en-4-ynoate

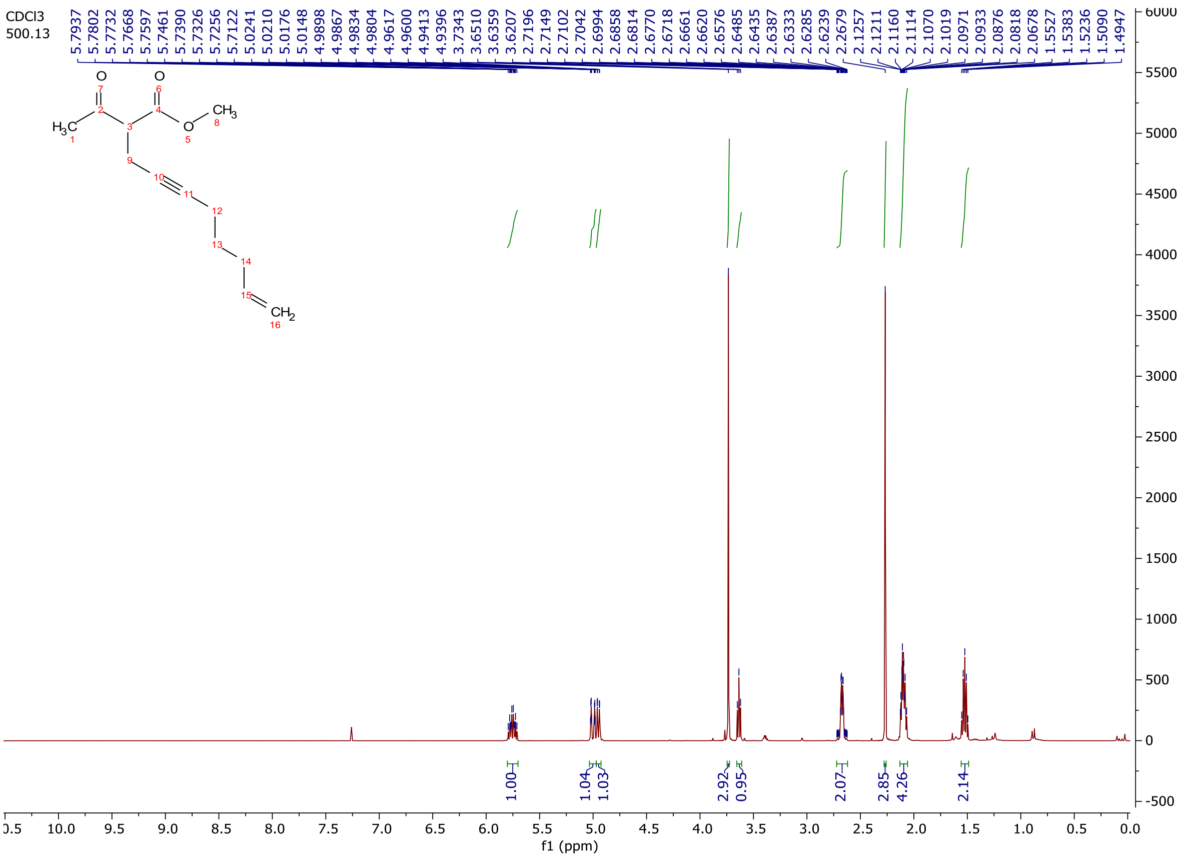

^{13}C{^{1}H} NMR (126 MHz, $CDCl_3$) of methyl 2-acetyldec-9-en-4-ynoate

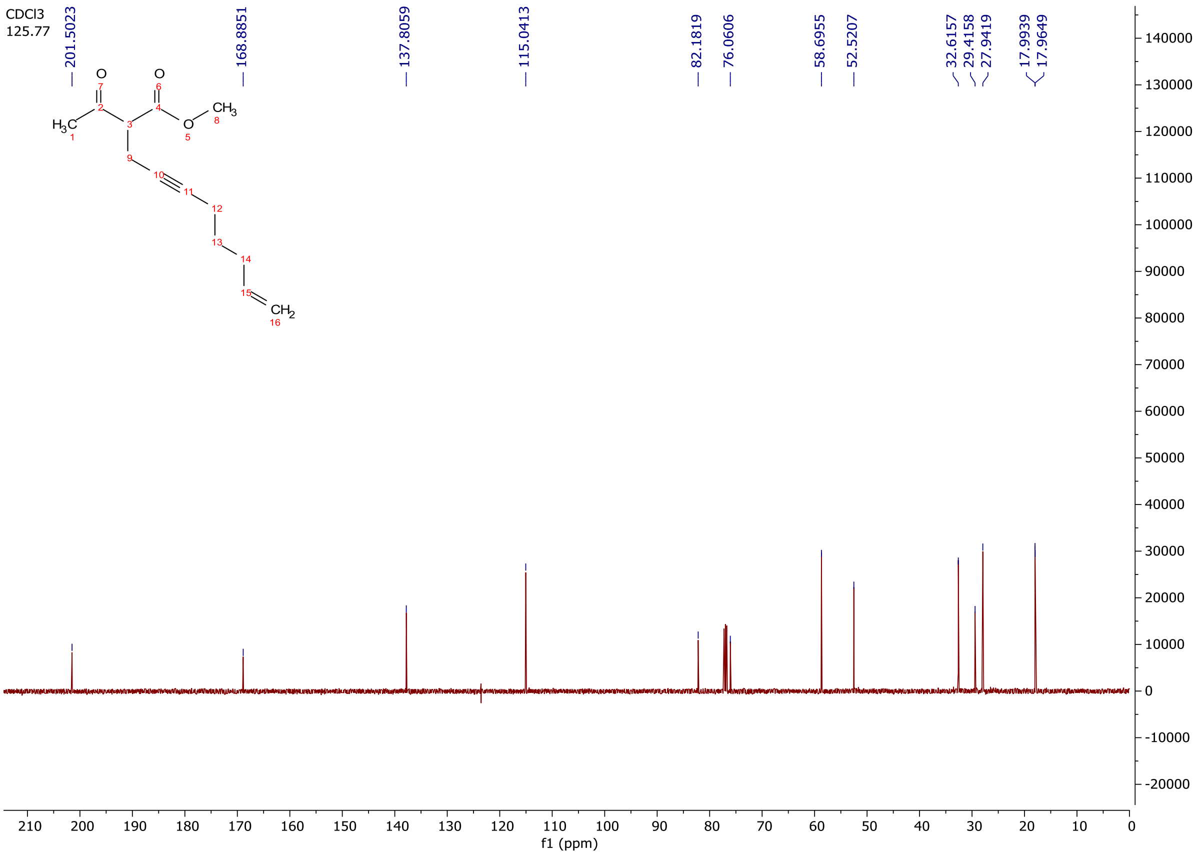

^{1}H NMR (400 MHz, $CDCl_3$) of methyl 2-acetylundec-10-en-4-ynoate

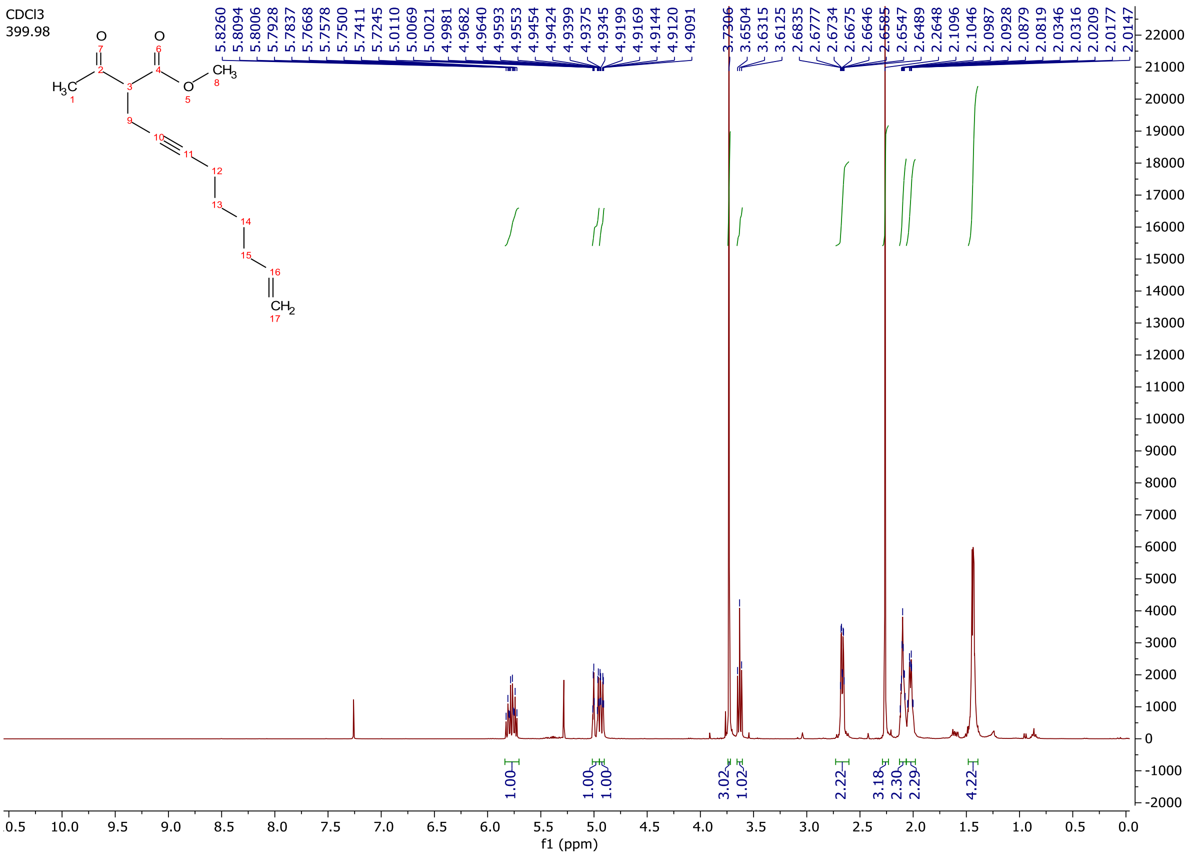

^{13}C{^{1}H} NMR (101 MHz, $CDCl_3$) of methyl 2-acetylundec-10-en-4-ynoate

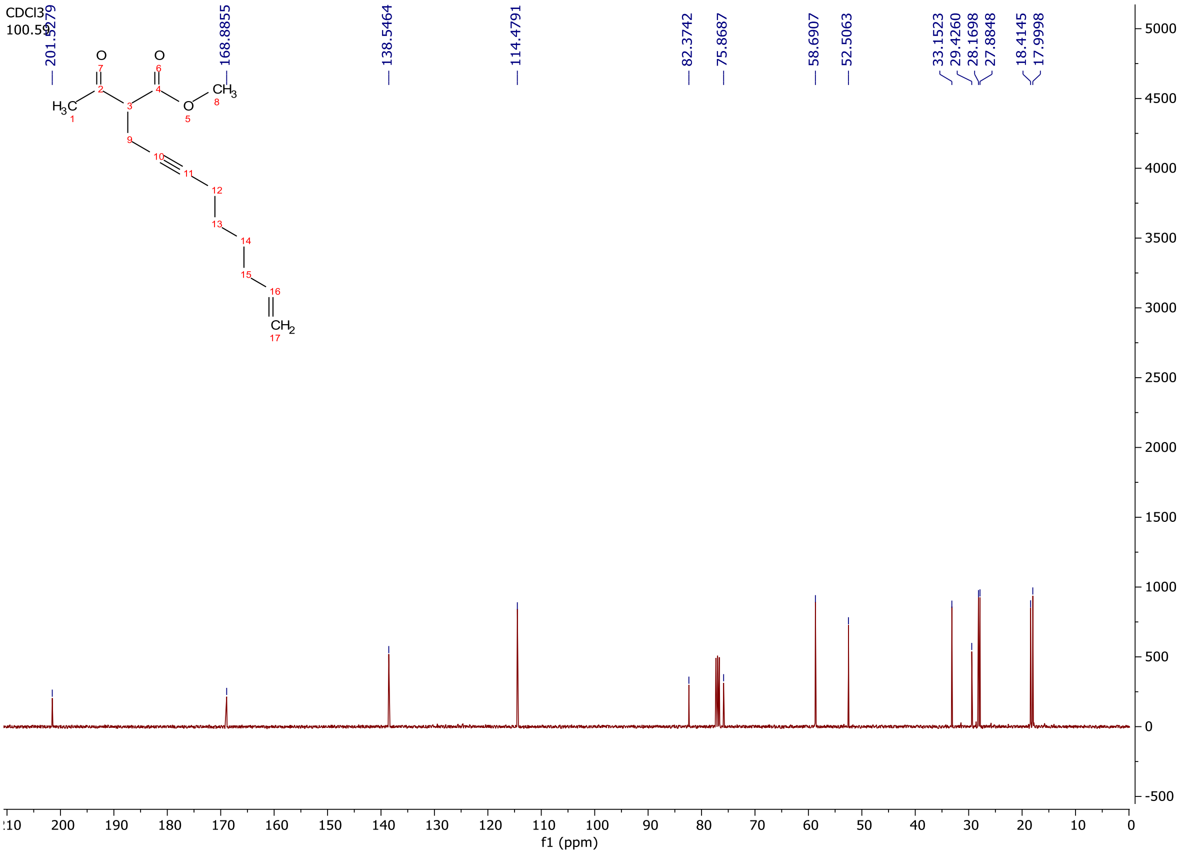

^{1}H NMR (400 MHz, $CDCl_3$) of methyl 2-acetylhex-4-ynoate

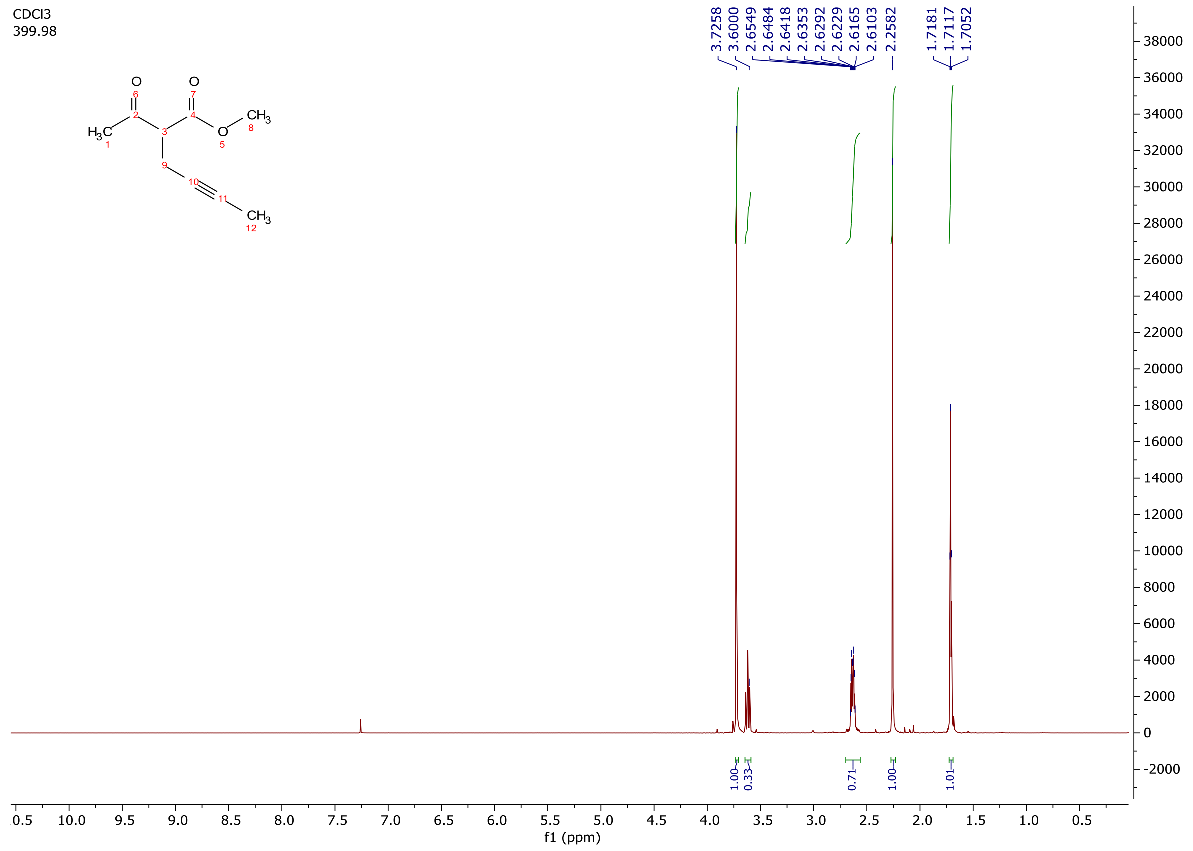

$^{13}C\{^{1}H\}$ NMR (101 MHz, $CDCl_3$) of methyl 2-acetylhex-4-ynoate

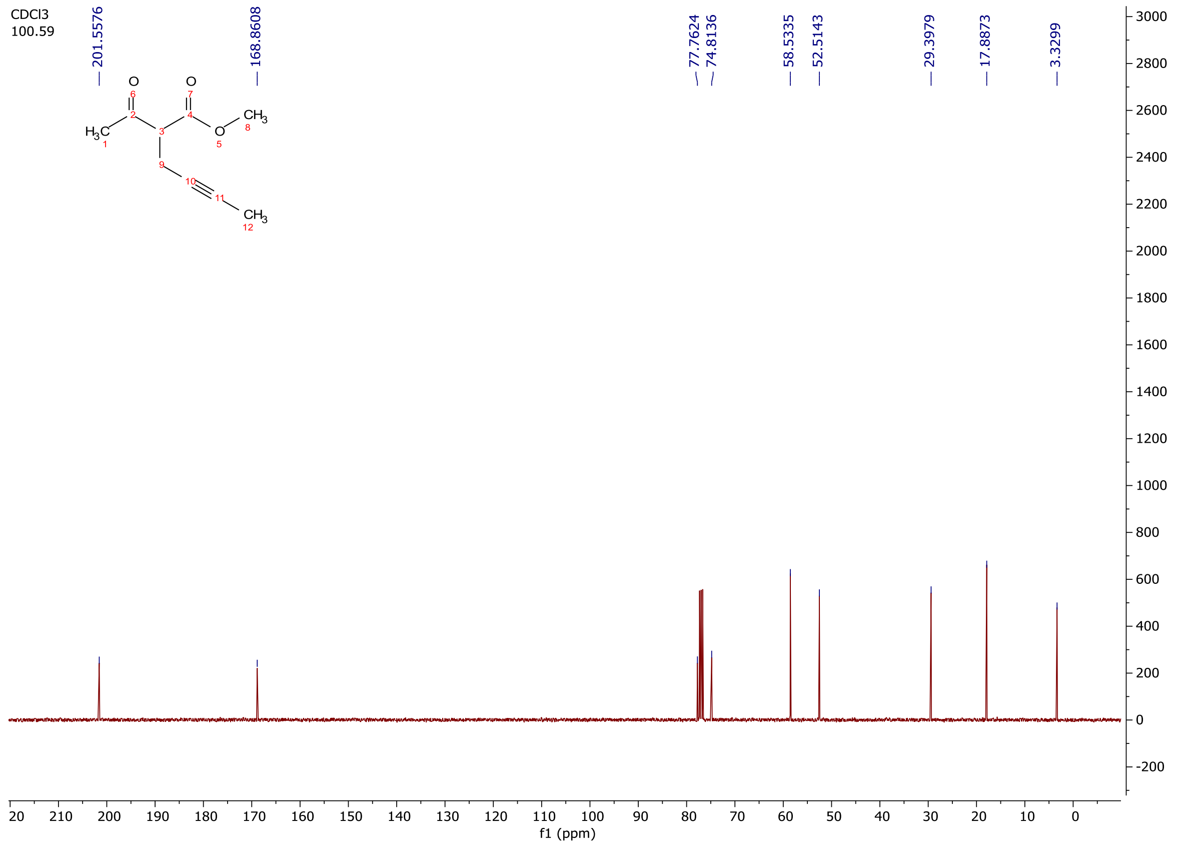

^{1}H NMR (400 MHz, CDCl$_3$) of isopropyl 2-acetylhex-4-ynoate

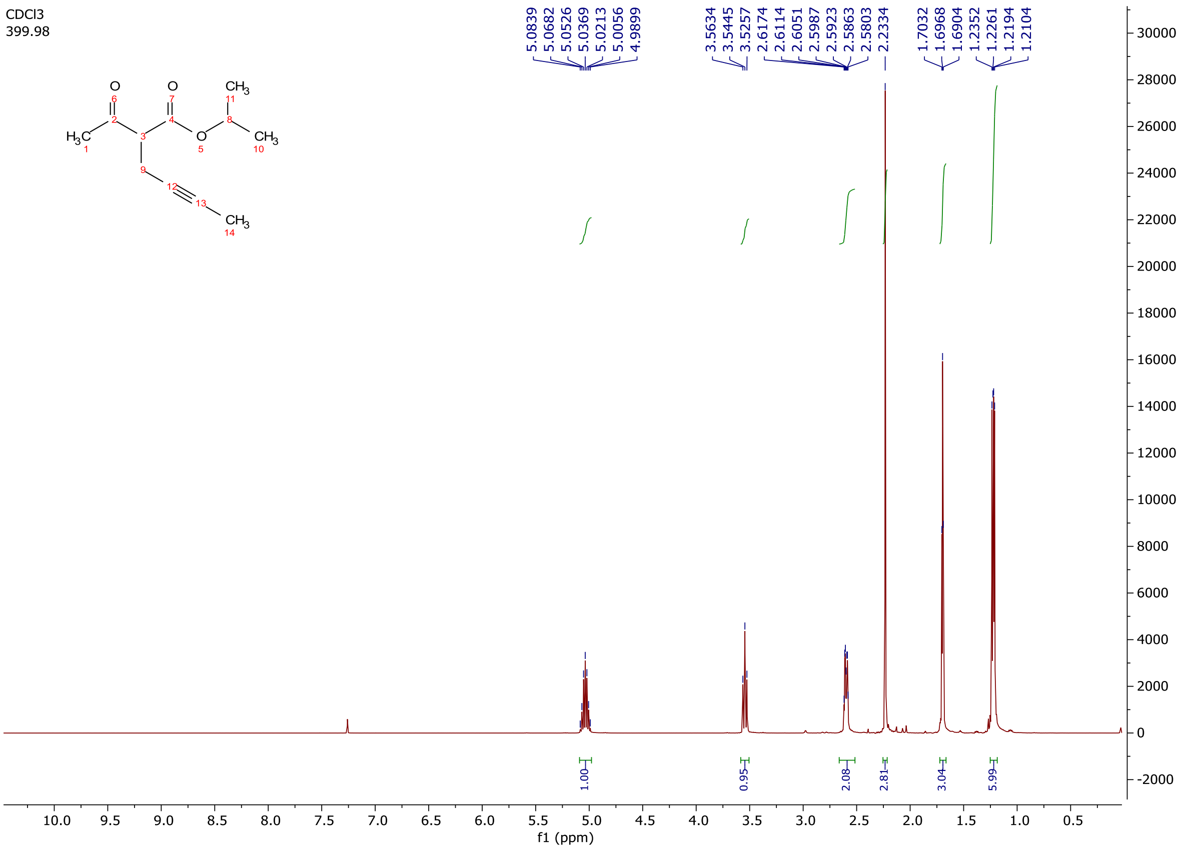

^{13}C{^{1}H} NMR (101 MHz, CDCl$_3$) of isopropyl 2-acetylhex-4-ynoate

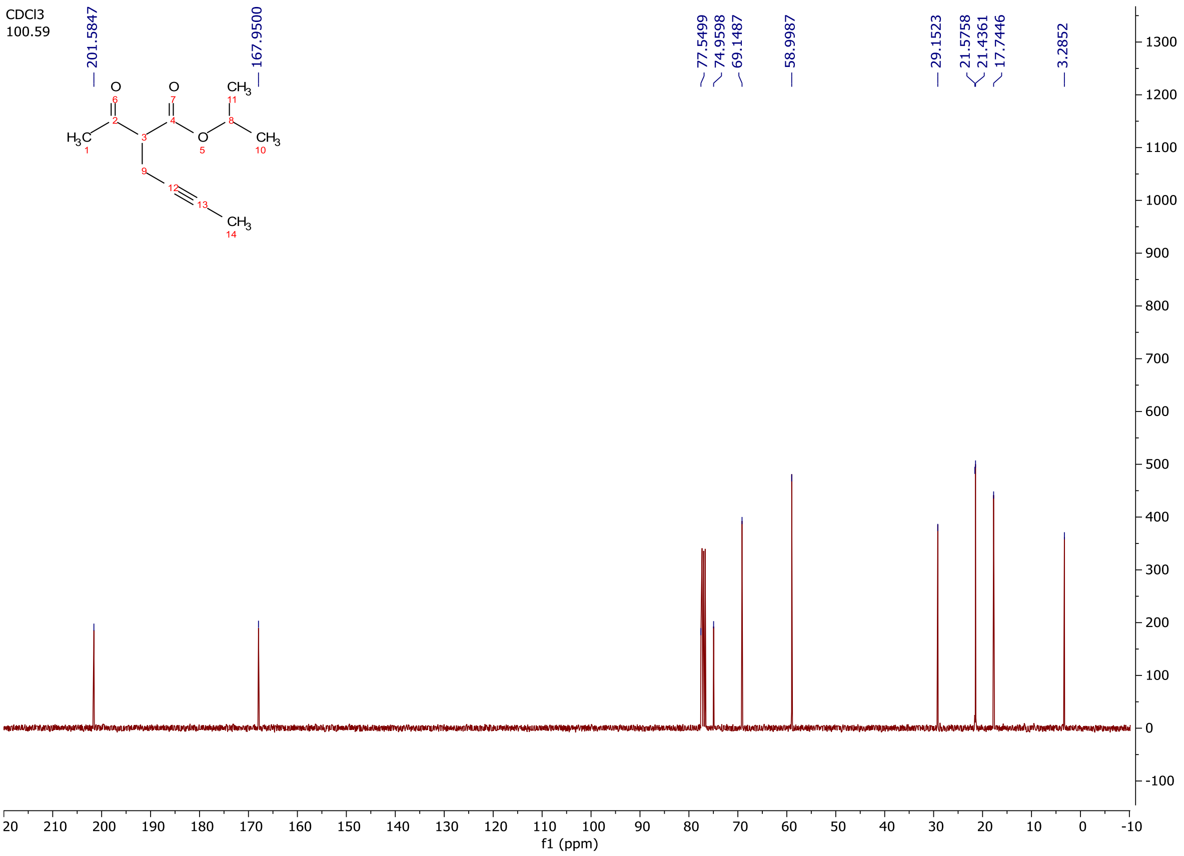

^{1}H NMR (400 MHz, CDCl$_3$) of tert-butyl 2-acetylhex-4-ynoate

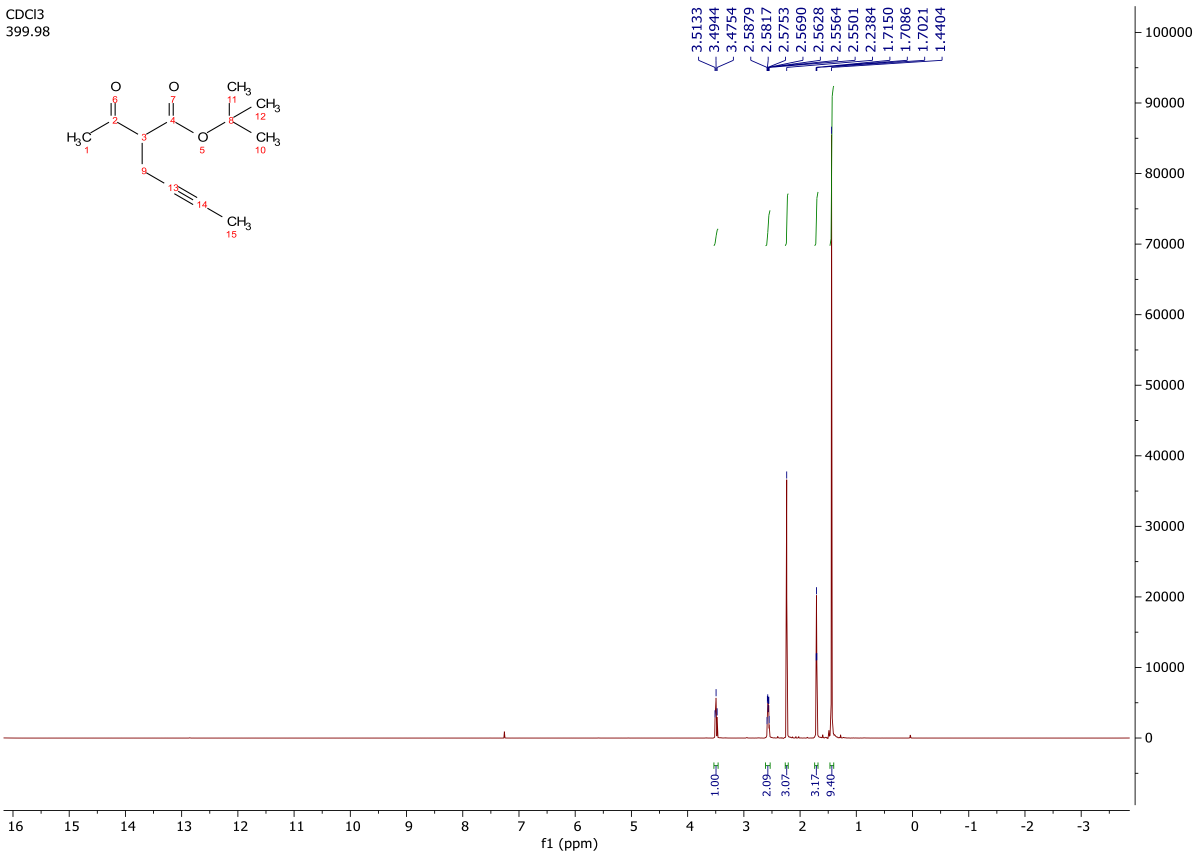

^{13}C{^{1}H} NMR (101 MHz, CDCl$_3$) of tert-butyl 2-acetylhex-4-ynoate

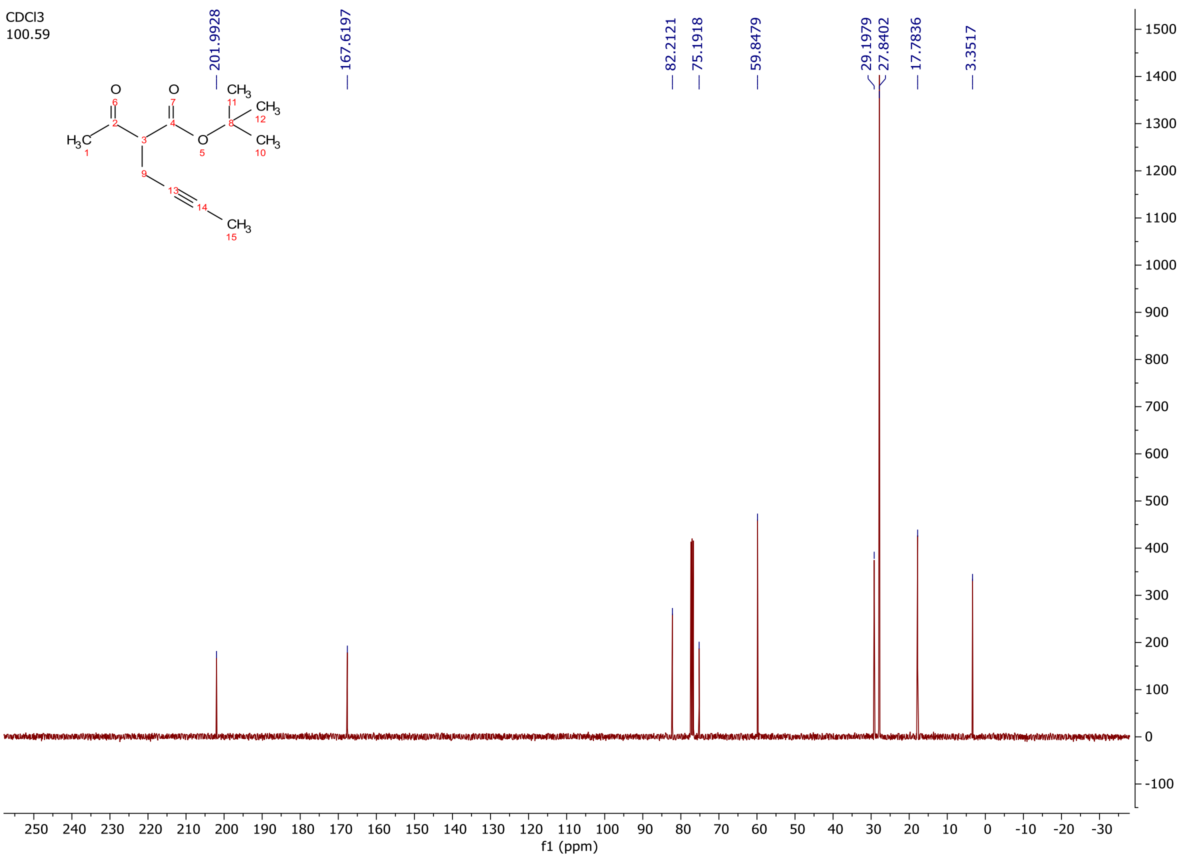

^{1}H NMR (400 MHz, $CDCl_3$) of ethyl 3-oxo-3-phenylpropanoate

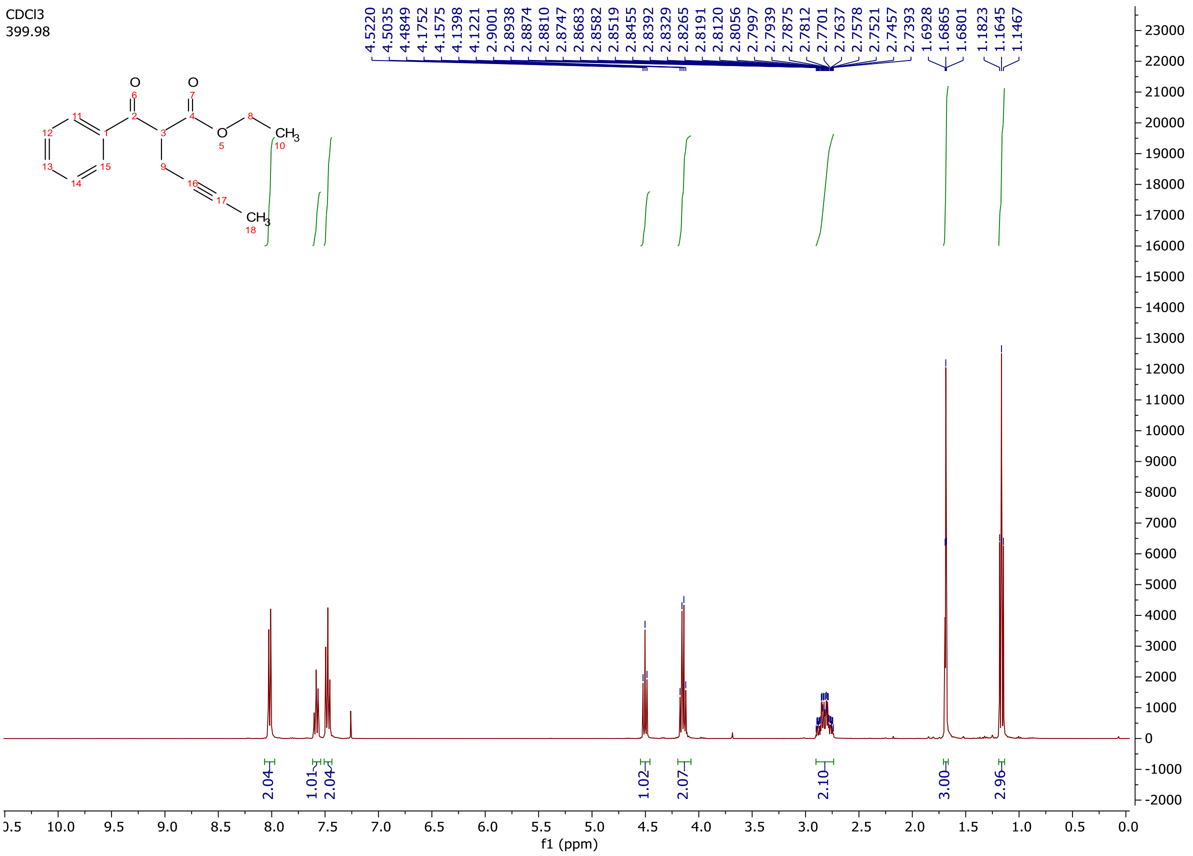

$^{13}C\{^{1}H\}$ NMR (101 MHz, $CDCl_3$) of ethyl 3-oxo-3-phenylpropanoate

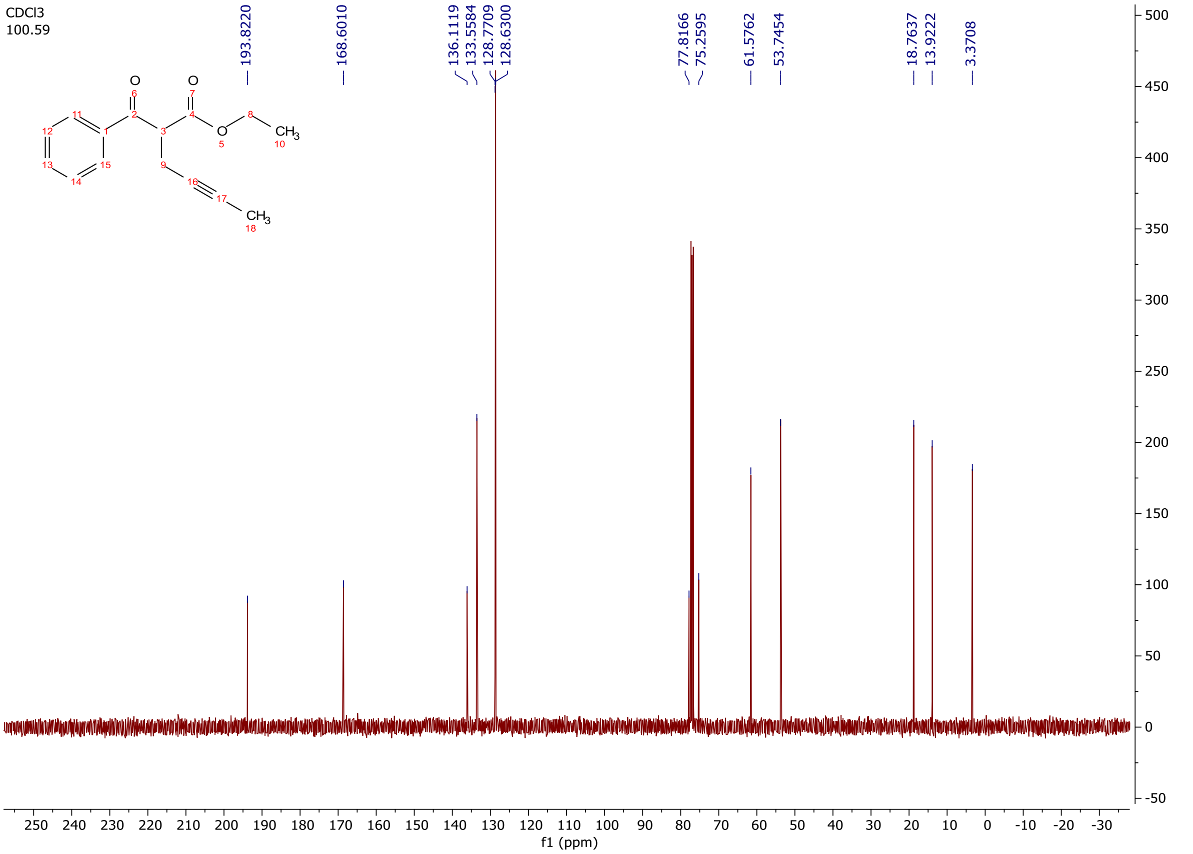

^{1}H NMR (400 MHz, $CDCl_3$) of ethyl 2-isobutyrylhex-4-ynoate

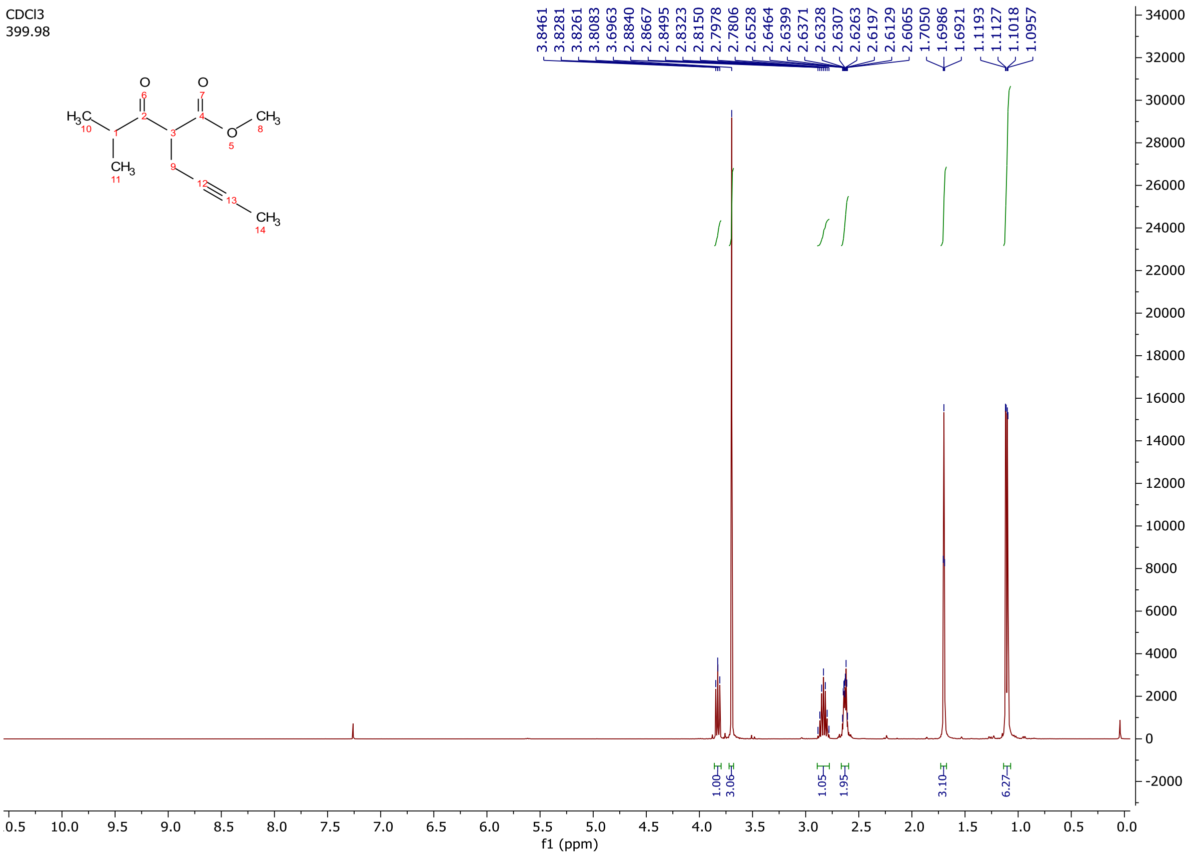

$^{13}C\{^{1}H\}$ NMR (101 MHz, $CDCl_3$) of ethyl 2-isobutyrylhex-4-ynoate

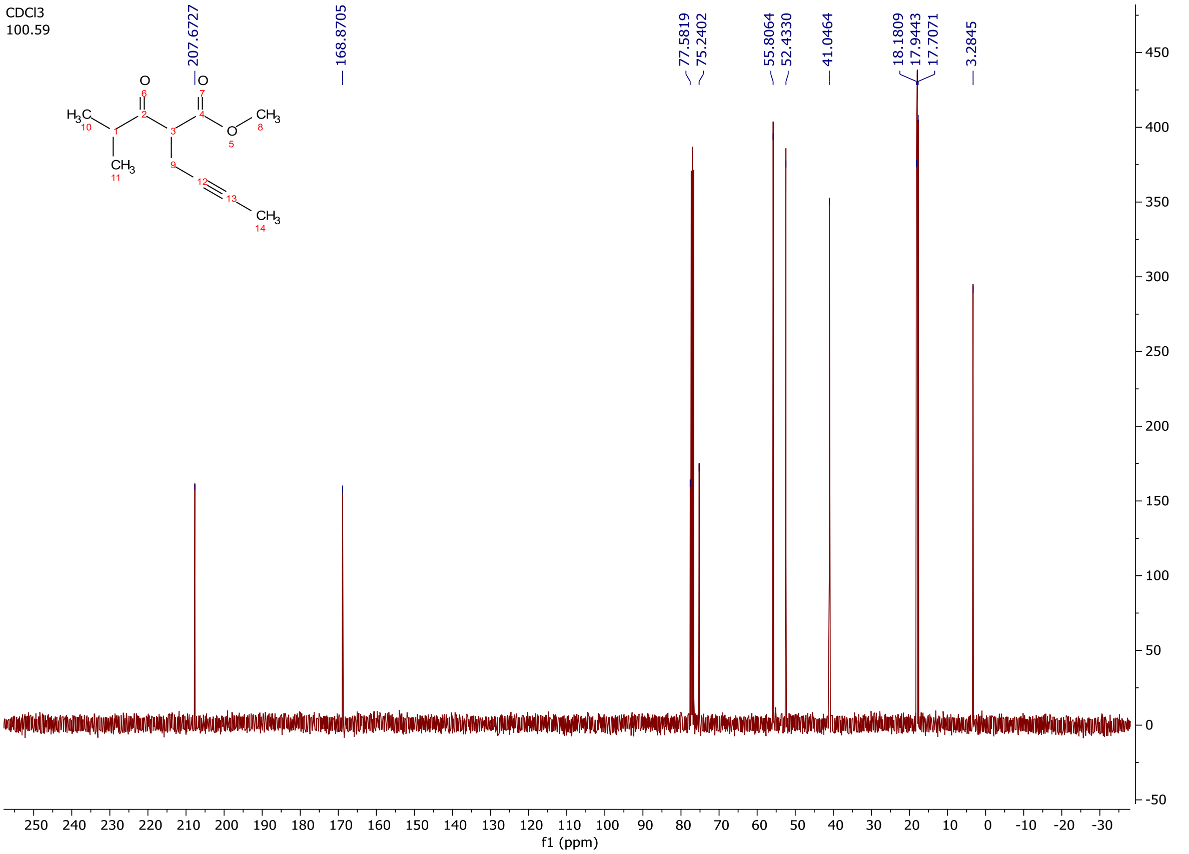

^{1}H NMR (400 MHz, $CDCl_3$) of methyl 2-acetylhept-4-ynoate

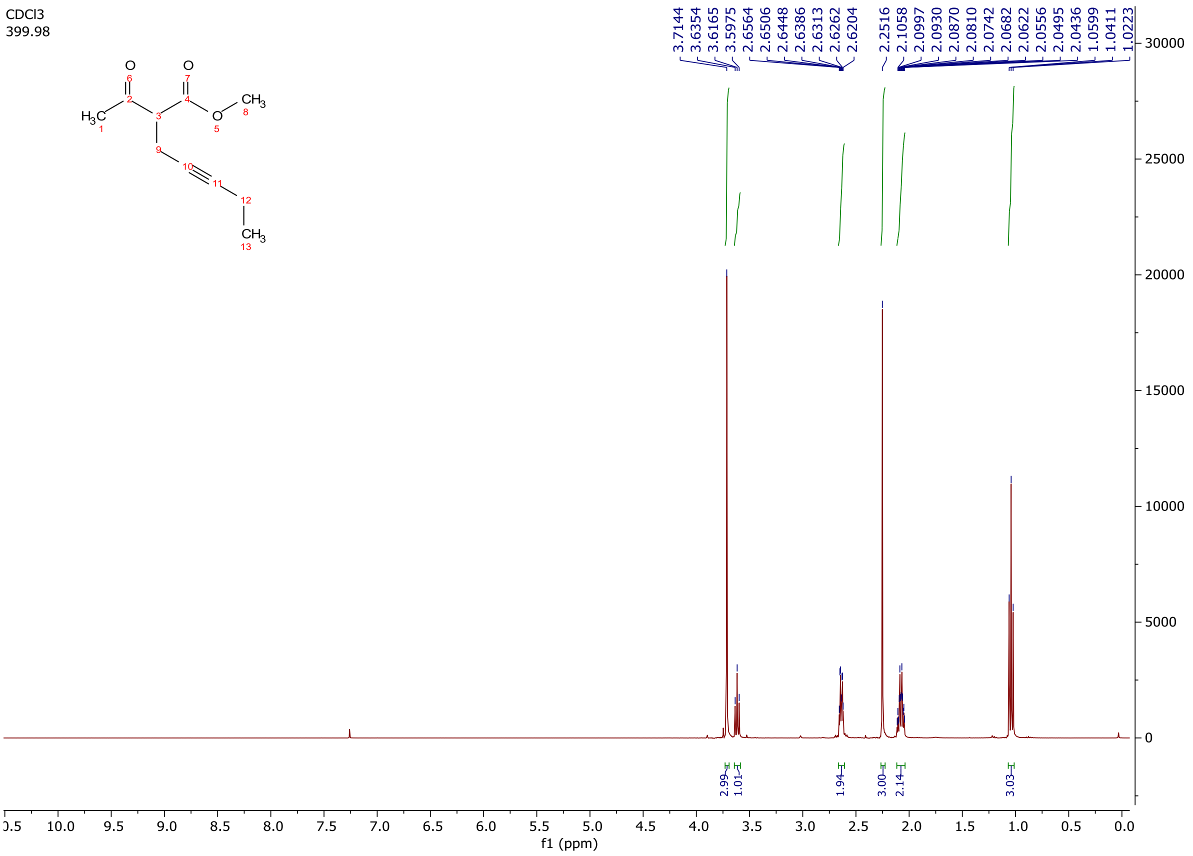

$^{13}C\{^{1}H\}$ NMR (101 MHz, $CDCl_3$) of methyl 2-acetylhept-4-ynoate

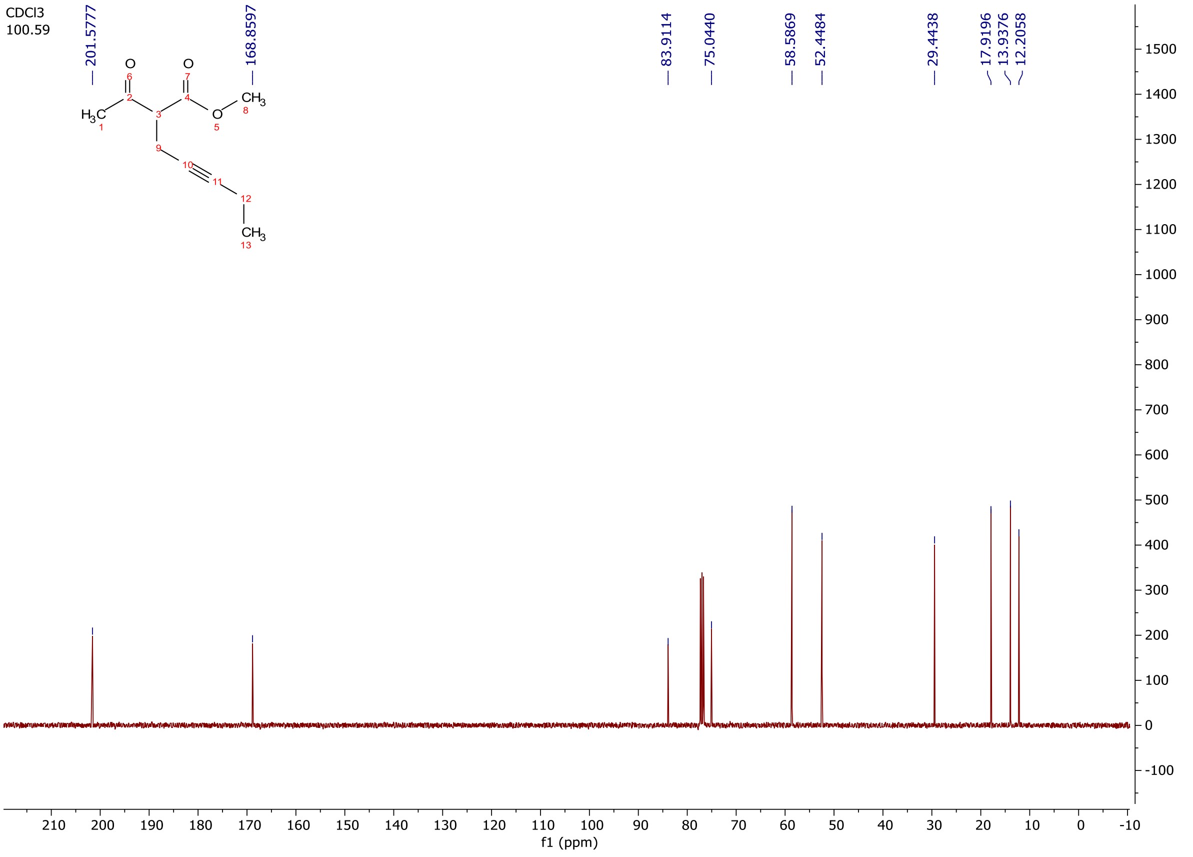

^1H NMR (400 MHz, $CDCl_3$) of 3-(but-2-yn-1-yl)pentane-2,4-dione

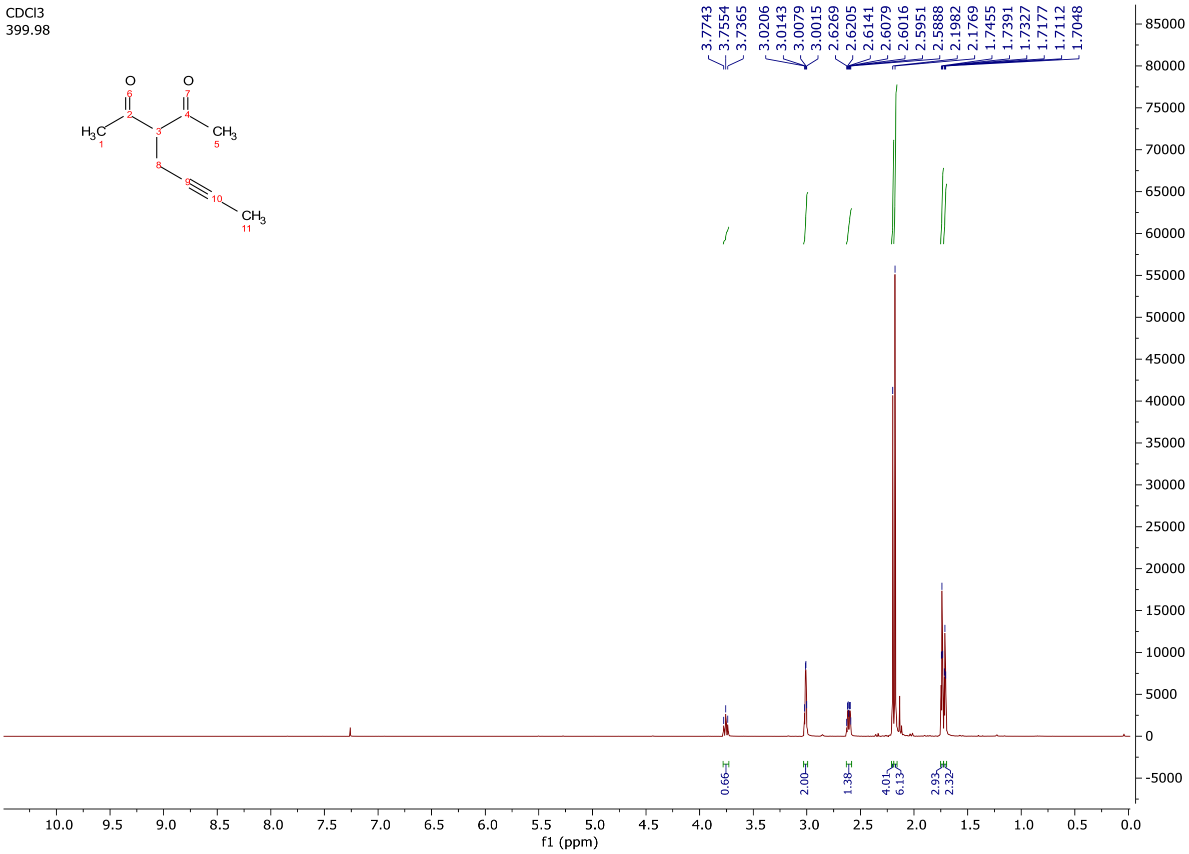

$^{13}C\{^1H\}$ NMR (101 MHz, $CDCl_3$) of 3-(but-2-yn-1-yl)pentane-2,4-dione

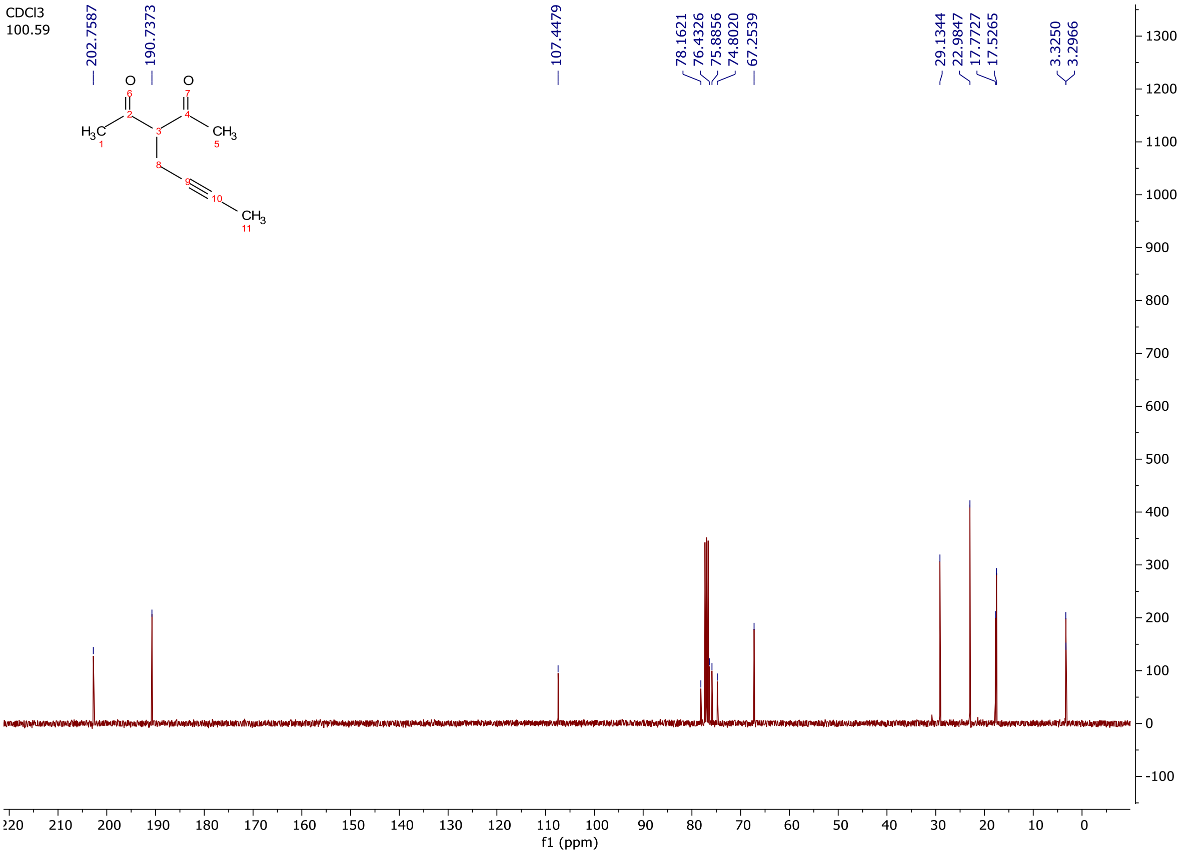

^{1}H NMR (400 MHz, $CDCl_3$) of 2-(but-2-yn-1-yl)-1-phenylbutane-1,3-dione

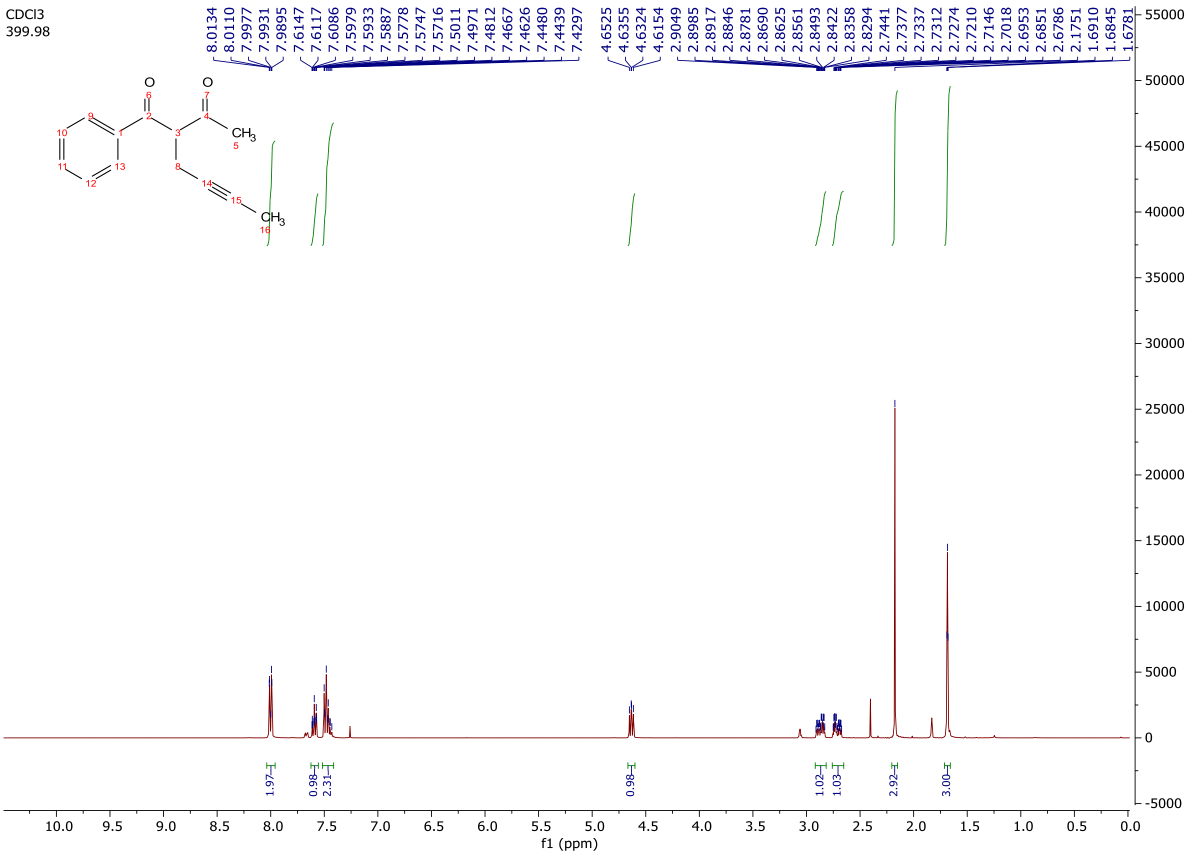

^{13}C{^{1}H} NMR (101 MHz, $CDCl_3$) of 2-(but-2-yn-1-yl)-1-phenylbutane-1,3-dione

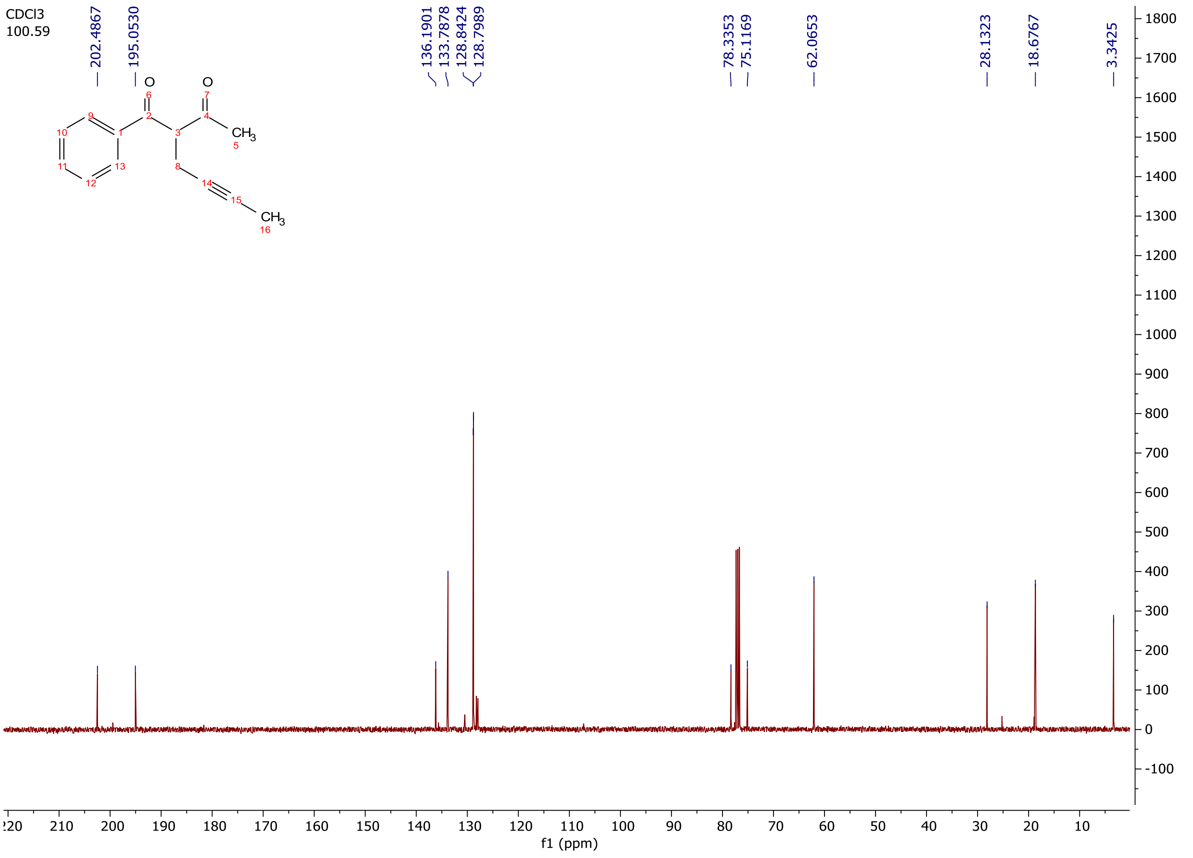

^{1}H NMR (400 MHz, $CDCl_3$) of 2-(but-2-yn-1-yl)-1,3-diphenylpropane-1,3-dione

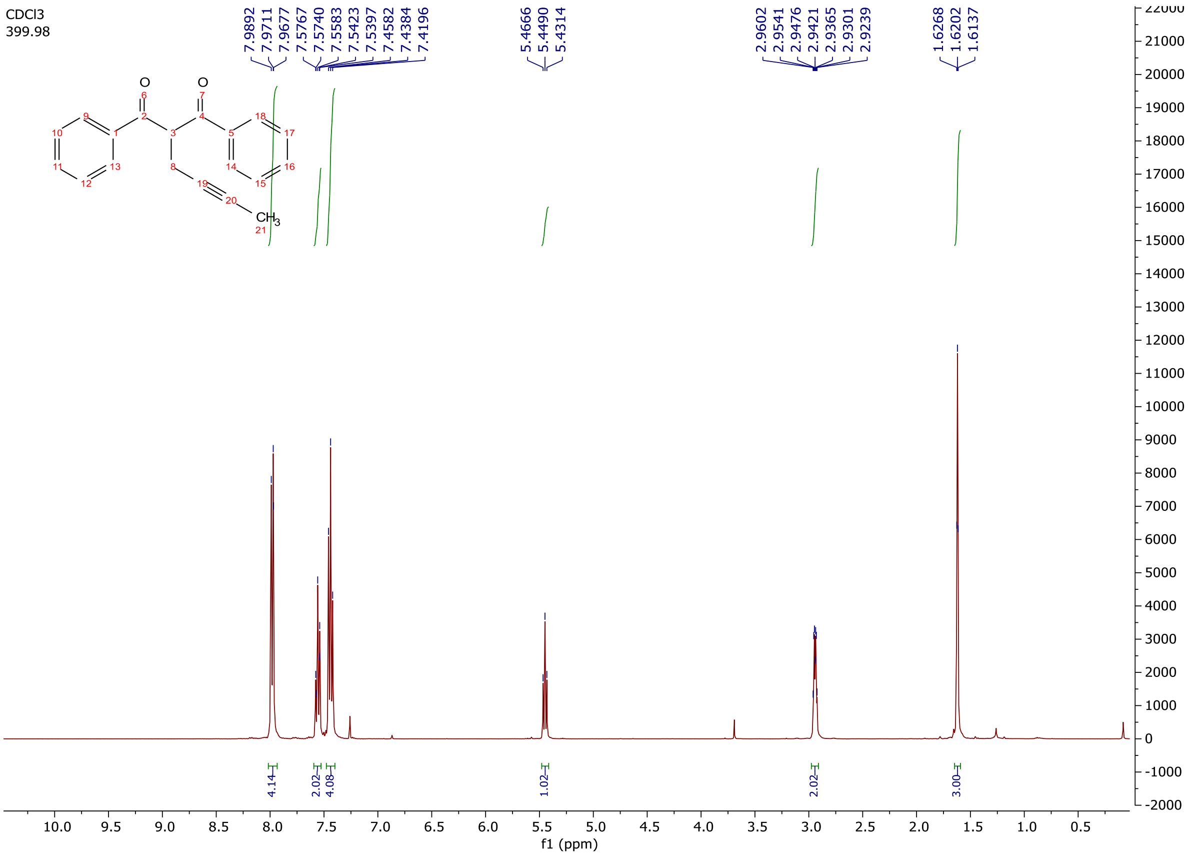

^{13}C{^{1}H} NMR (101 MHz, $CDCl_3$) of 2-(but-2-yn-1-yl)-1,3-diphenylpropane-1,3-dione

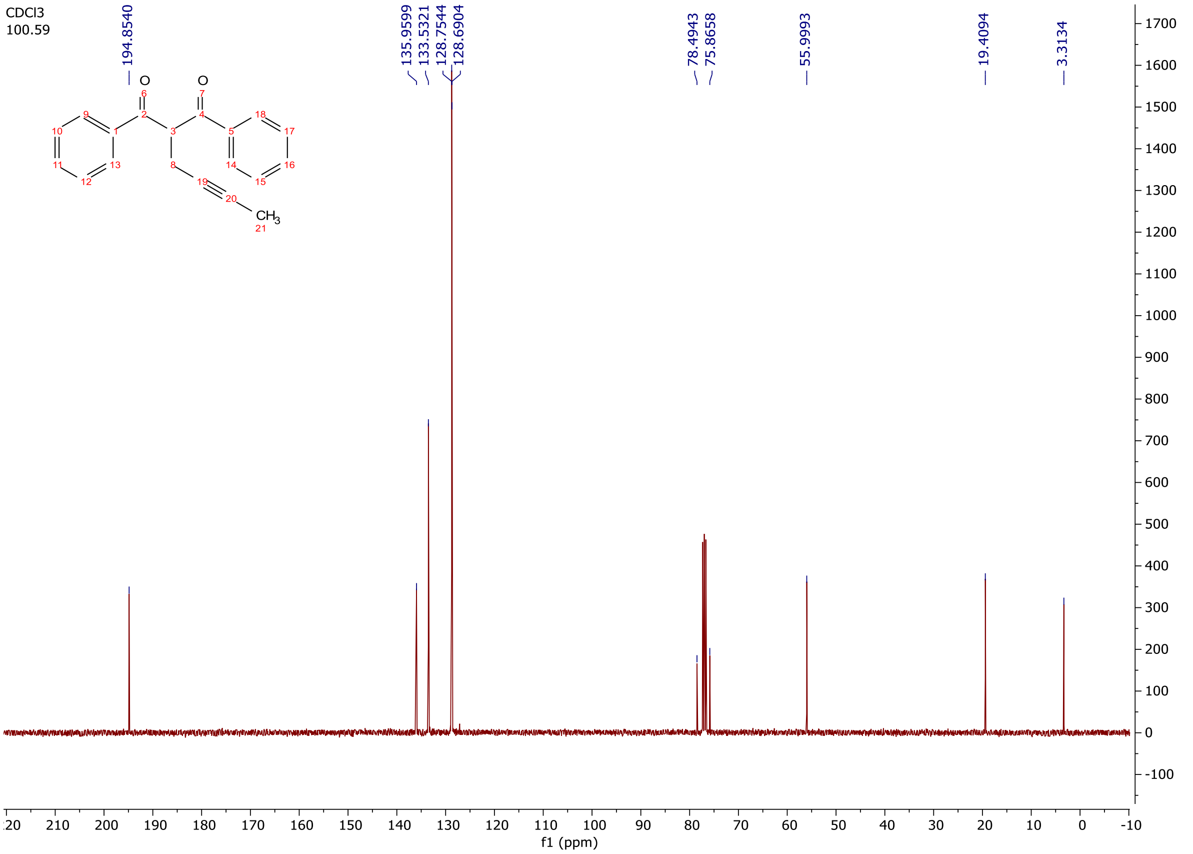

^{1}H NMR (400 MHz, $CDCl_3$) of 3-(phenylsulfonyl)hept-5-yn-2-one

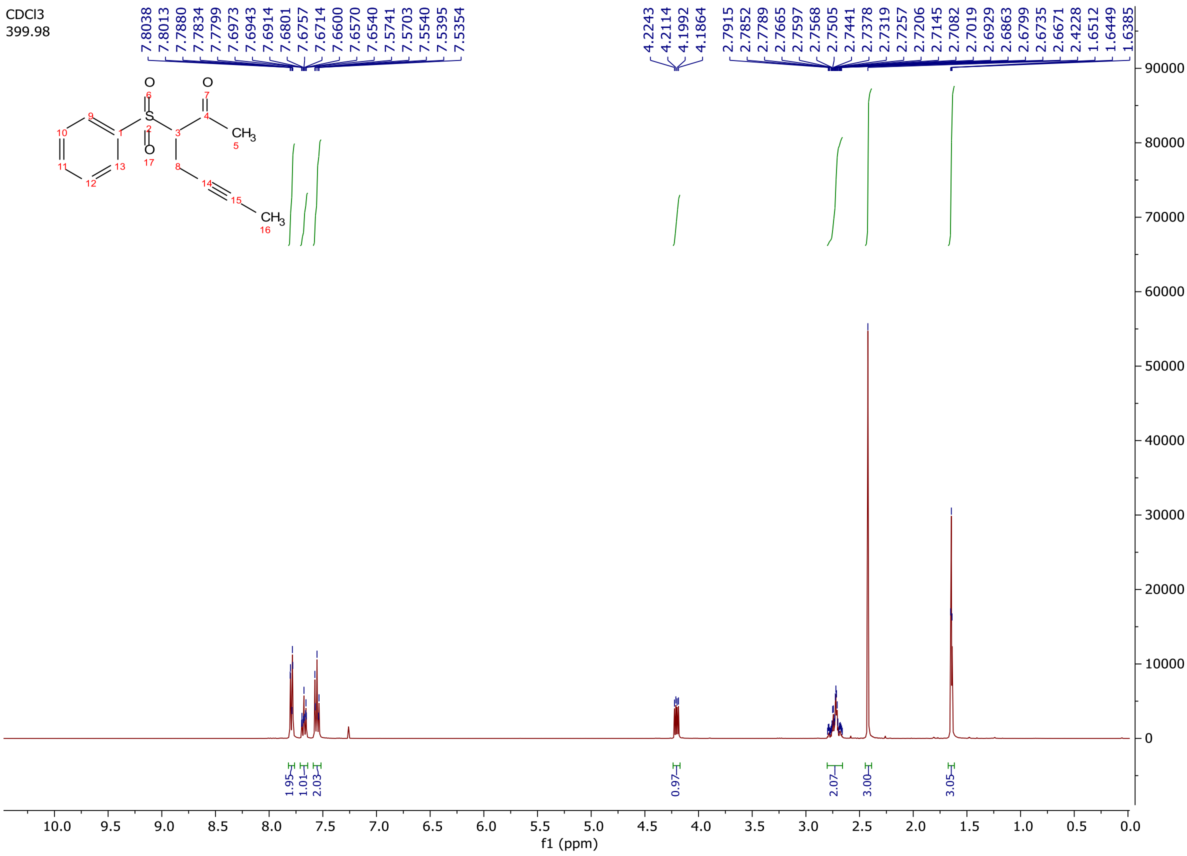

$^{13}C\{^{1}H\}$ NMR (101 MHz, $CDCl_3$) of 3-(phenylsulfonyl)hept-5-yn-2-one

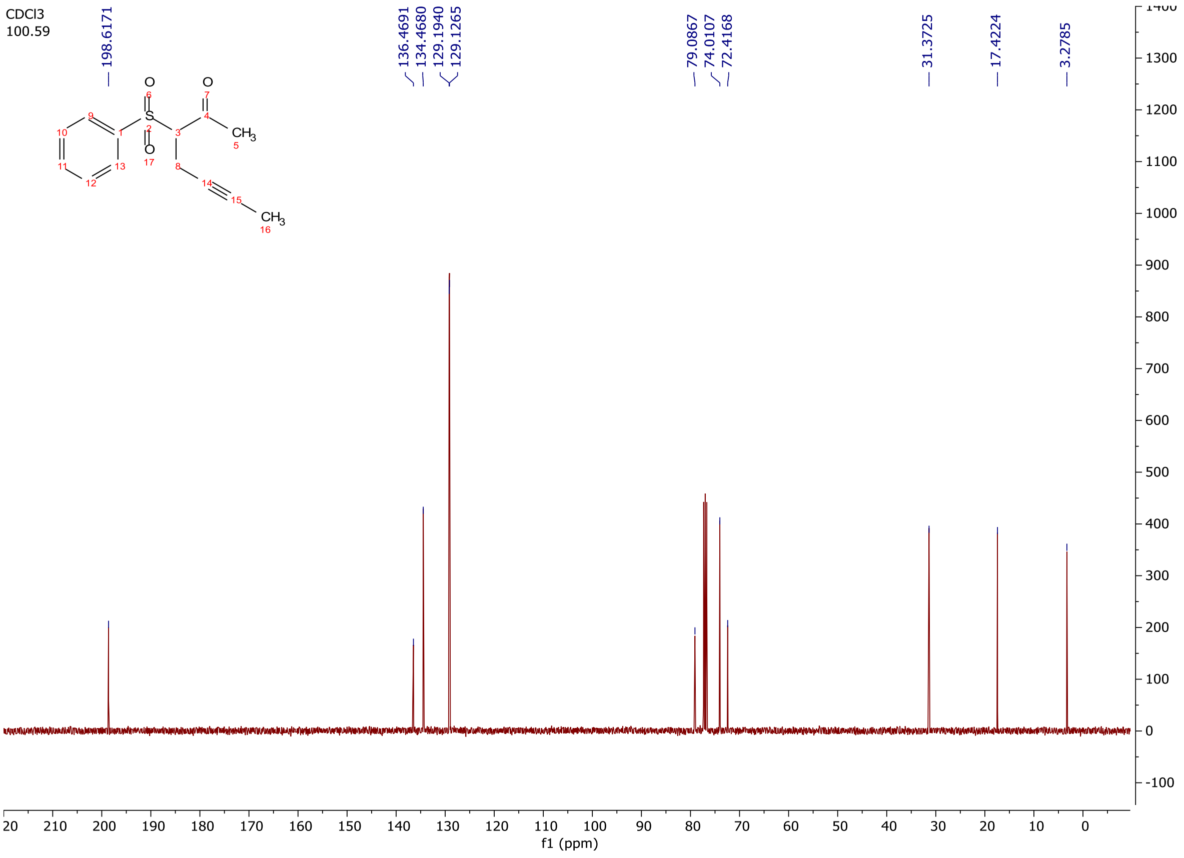

^{1}H NMR (400 MHz, $CDCl_3$) of 3-(methylsulfonyl)hept-5-yn-2-one

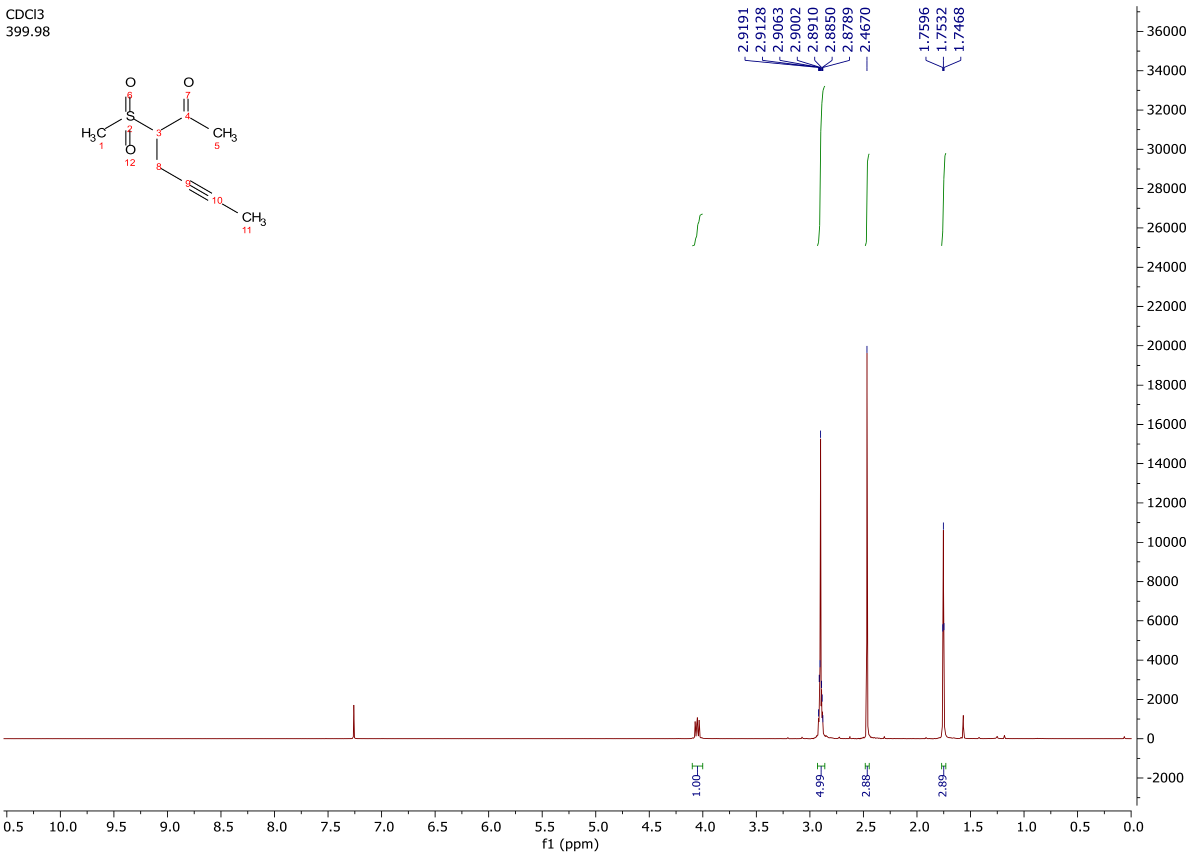

$^{13}C\{^{1}H\}$ NMR (101 MHz, $CDCl_3$) of 3-(methylsulfonyl)hept-5-yn-2-one

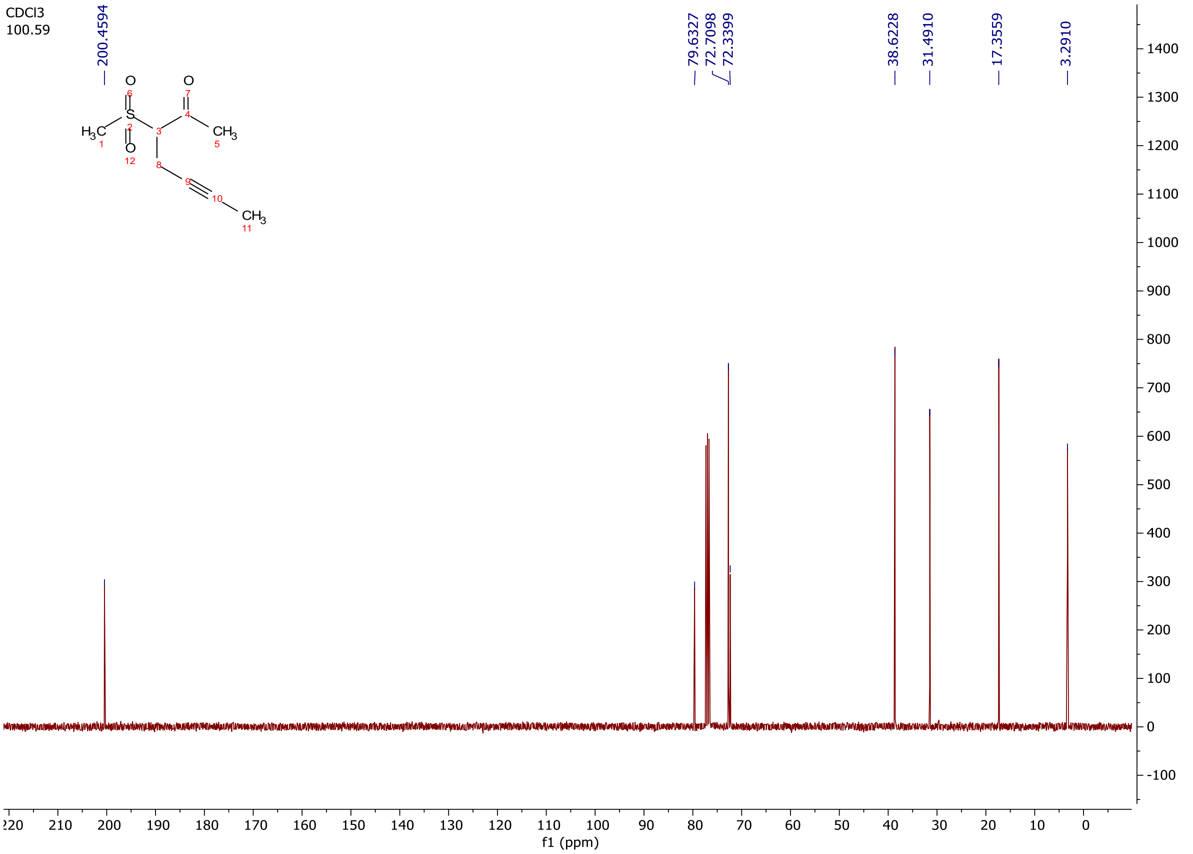

References

1 N. C. Bruno, M. T. Tudge and S. L. Buchwald, *Chem. Sci.*, 2013, **4**, 916–920.
2 J. Xiang, X.-X. Jin, Q.-Q. Su, S.-C. Cheng, C.-C. Ko, W.-L. Man, M. Xue, L. Wu, C.-M. Che and T.-C. Lau, *Commun. Chem.*, 2019, **2**, 40.
3 B. Bisek and W. Chaładaj, *Adv. Synth. Catal.*, 2022, **364**, 4281–4288.
4 R. J. Armstrong-Chong, K. Matthews and J. M. Chong, *Tetrahedron*, 2004, **60**, 10239–10244.
5 Gaussian 16, Revision C.01, M. J. Frisch, G. W. Trucks, H. B. Schlegel, G. E. Scuseria, M. A. Robb, J. R. Cheeseman, G. Scalmani, V. Barone, G. A. Petersson, H. Nakatsuji, X. Li, M. Cari-cato, A. V. Marenich, J. Bloino, B. G. Janesko, R. Gomperts, B. Mennucci, H. P. Hratchian, J. V. Ortiz, A. F. Izmaylov, J. L. Sonnenberg, D. Williams-Young, F. Ding, F. Lipparini, F. Egidi, J. Goings, B. Peng, A. Petrone, T. Henderson, D. Ranasinghe, V. G. Zakrzewski, J. Gao, N. Rega, G. Zheng, W. Liang, M. Hada, M. Ehara, K. Toyota, R. Fukuda, J. Hasegawa, M. Ishida, T. Nakajima, Y. Honda, O. Kitao, H. Nakai, T. Vreven, K. Throssell, J. A. Montgomery, Jr., J. E. Peralta, F. Ogliaro, M. J. Bearpark, J. J. Heyd, E. N. Brothers, K. N. Kudin, V. N. Staroverov, T. A. Keith, R. Kobayashi, J. Normand, K. Raghavachari, A. P. Rendell, J. C. Burant, S. S. Iyengar, J. Tomasi, M. Cossi, J. M. Millam, M. Klene, C. Adamo, R. Cammi, J. W. Ochterski, R. L. Mar-tin, K. Morokuma, O. Farkas, J. B. Foresman, and D. J. Fox, Gaussian, Inc., Wallingford CT, 2019.
6 Y. Zhao and D. G. Truhlar, *Theor. Chem. Acc.*, 2008, **120**, 215–241.
7 A. V. Marenich, C. J. Cramer and D. G. Truhlar, *J. Phys. Chem. B*, 2009, **113**, 6378–6396.
8 CYLview20; Legault, C. Y., Université de Sherbrooke, 2020 (http://www.cylview.org)
